# Supplementary figures and images for: TAK1-mediated phosphorylation of PLCE1 represses PIP2 hydrolysis to impede esophageal squamous cancer metastasis (part 1 of 2)
Source: eLife. 2025 Apr 23;13:RP97373. doi: 10.7554/eLife.97373 (PMC12017773; doi:10.7554/eLife.97373)

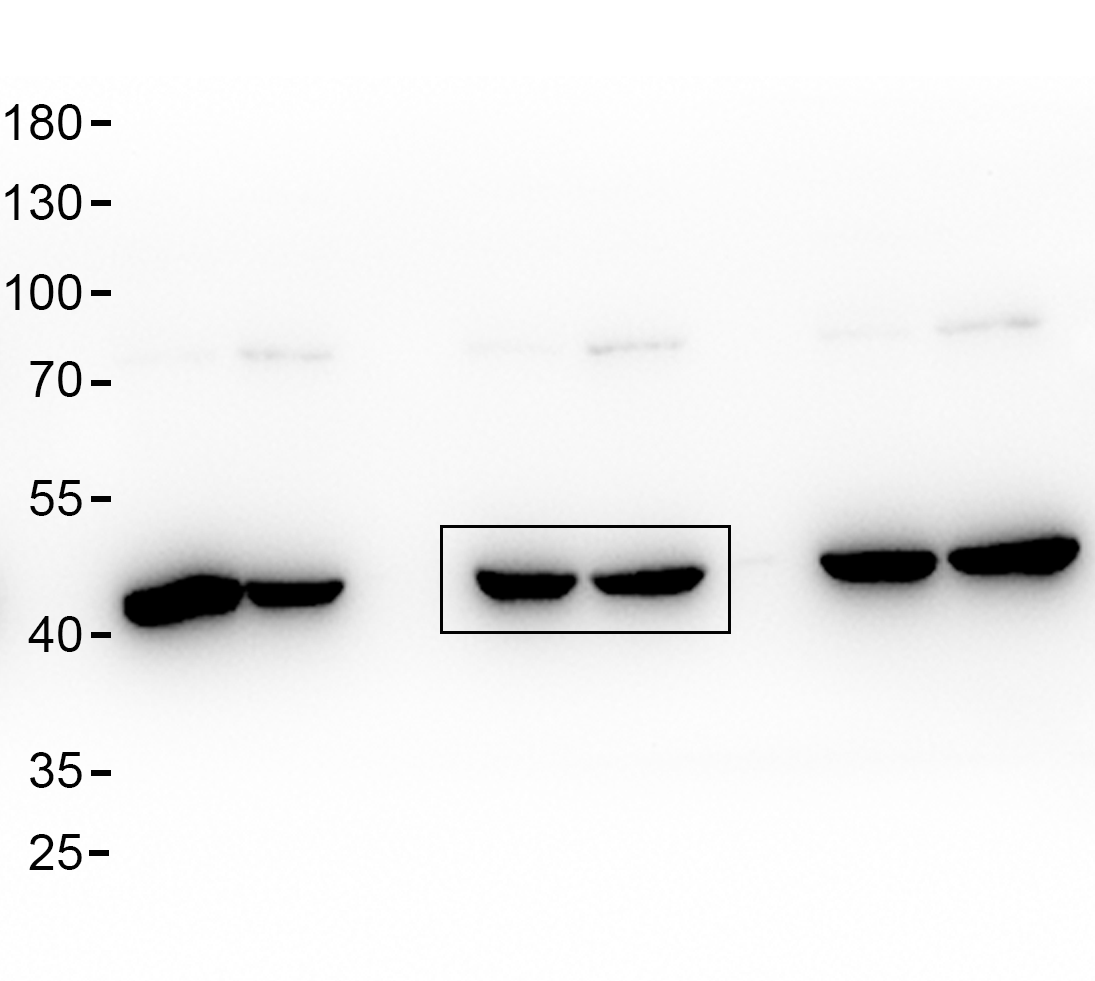

Supplement: Figure 1—source data 3. [file elife-97373-fig1-data3.zip › Figure 1-source data 2/Figure 1A/Actin.tif]

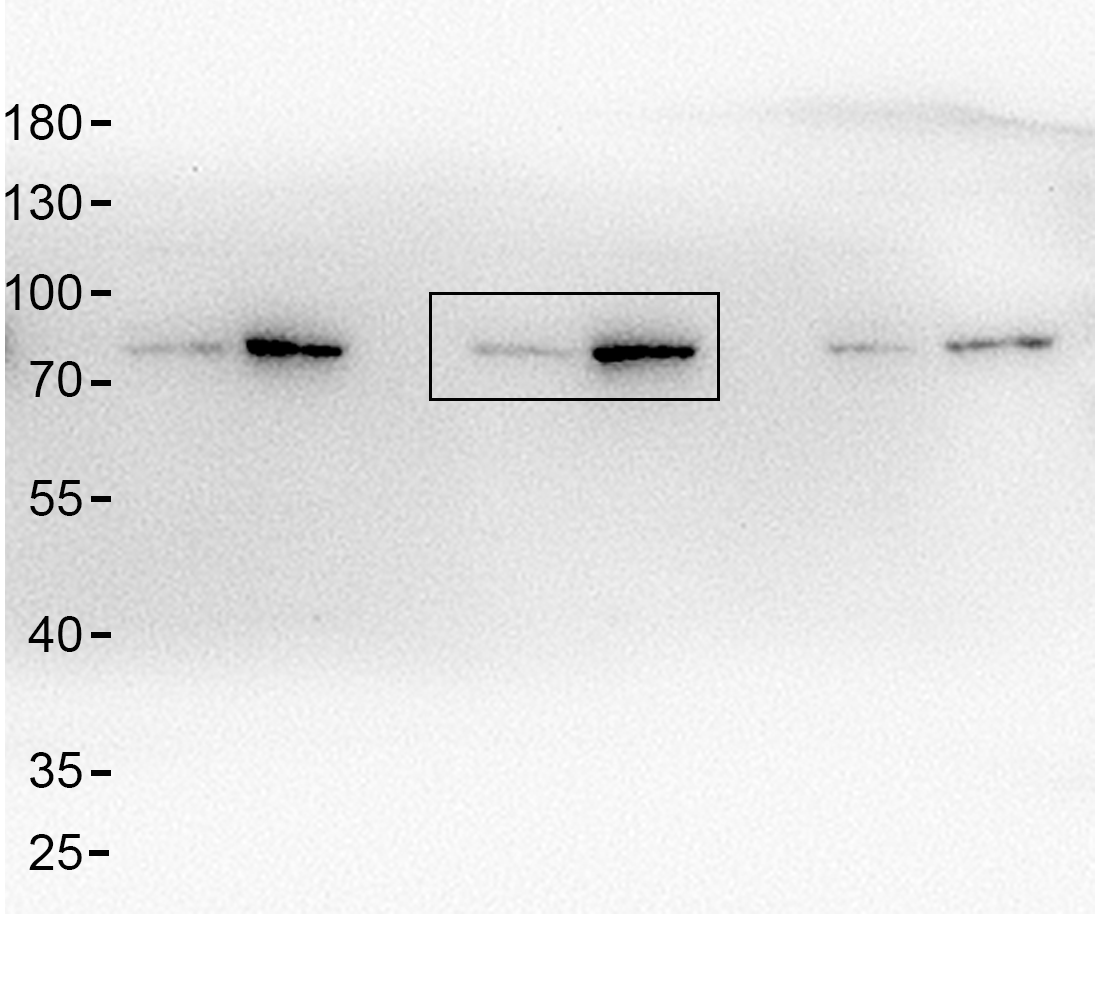

Supplement: Figure 1—source data 3. [file elife-97373-fig1-data3.zip › Figure 1-source data 2/Figure 1A/TAK1.tif]

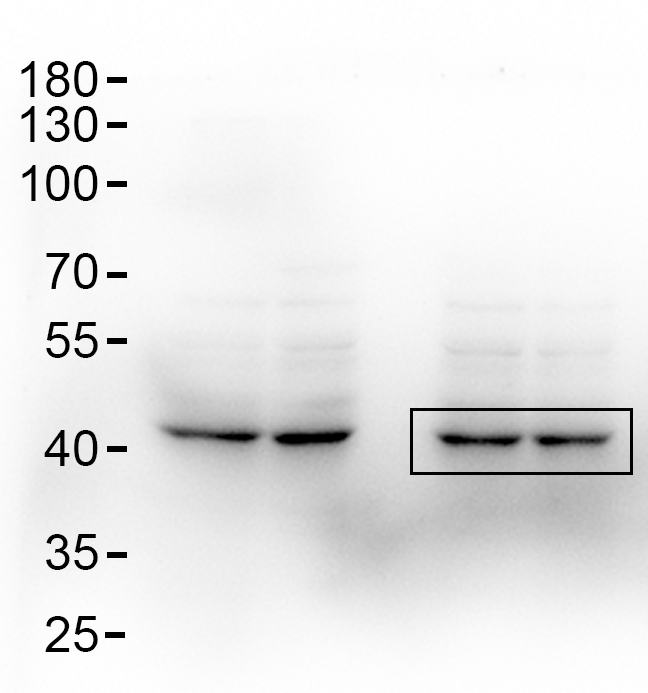

Supplement: Figure 1—source data 3. [file elife-97373-fig1-data3.zip › Figure 1-source data 2/Figure 1E/Actin.tif]

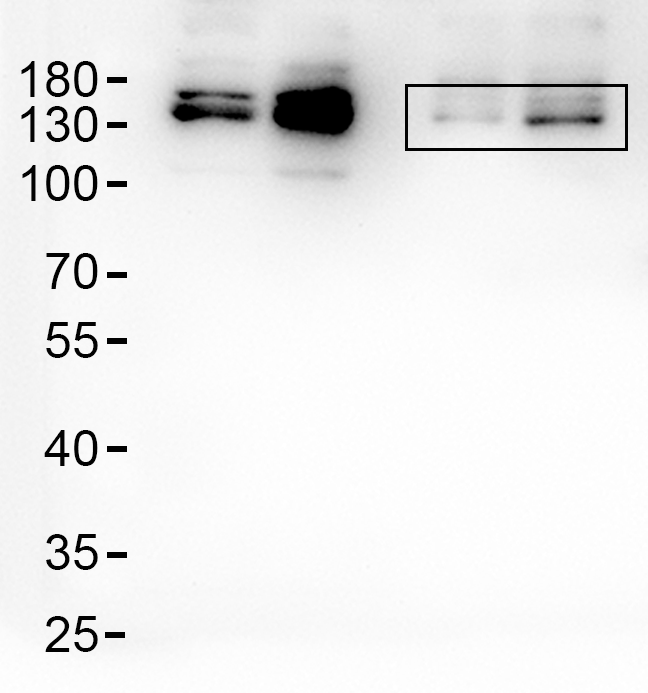

Supplement: Figure 1—source data 3. [file elife-97373-fig1-data3.zip › Figure 1-source data 2/Figure 1E/E-cadherin.tif]

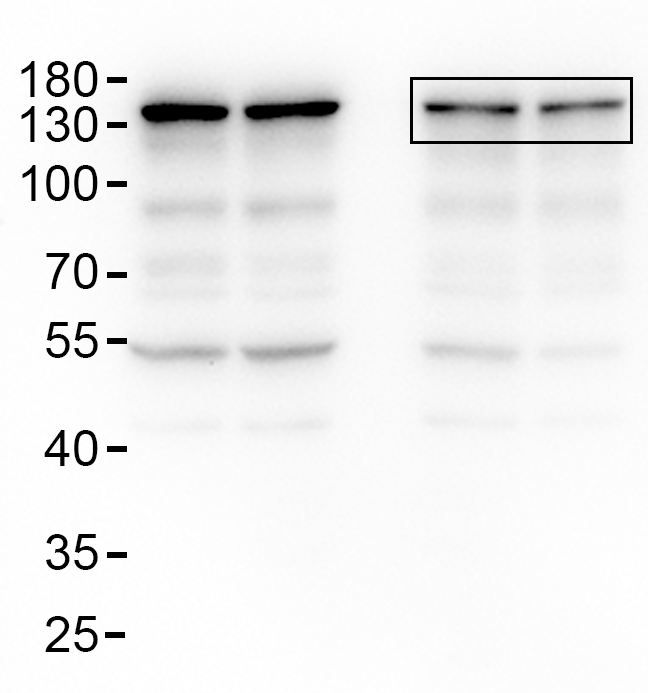

Supplement: Figure 1—source data 3. [file elife-97373-fig1-data3.zip › Figure 1-source data 2/Figure 1E/N-cadherin.tif]

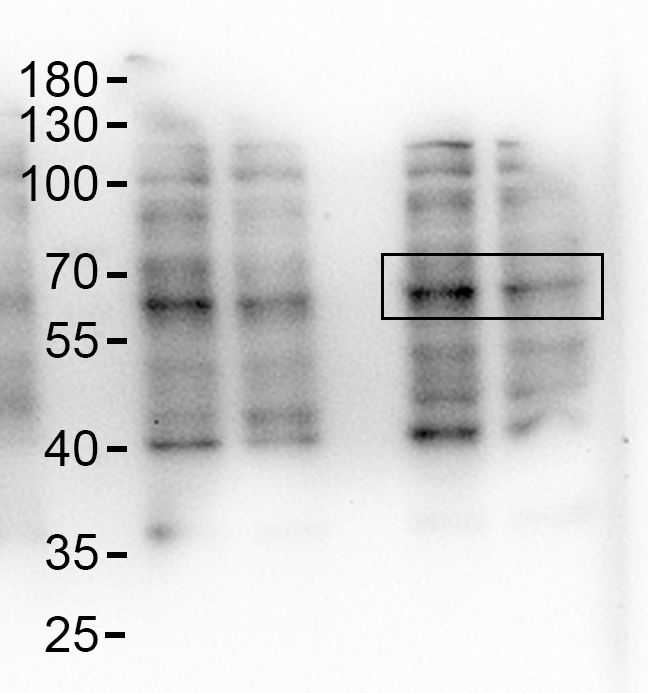

Supplement: Figure 1—source data 3. [file elife-97373-fig1-data3.zip › Figure 1-source data 2/Figure 1E/Vimentin.tif]

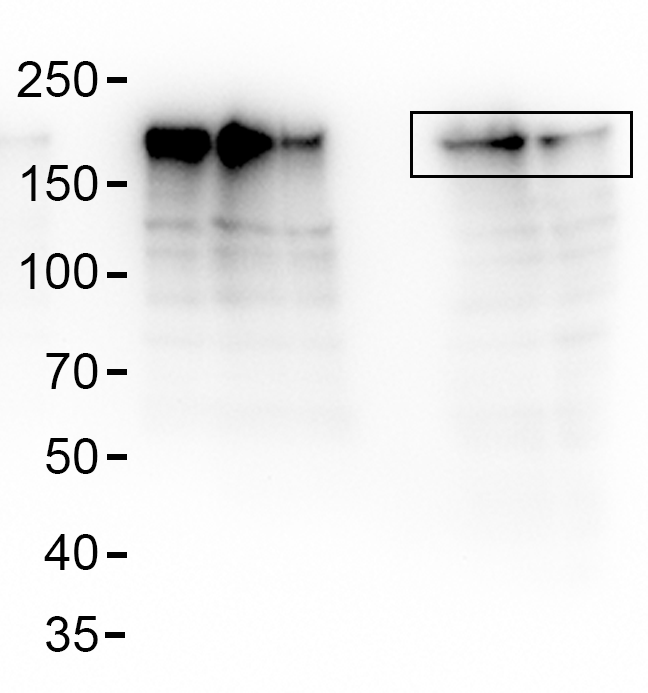

Supplement: Figure 1—source data 3. [file elife-97373-fig1-data3.zip › Figure 1-source data 2/Figure 1E/ZEB1.tif]

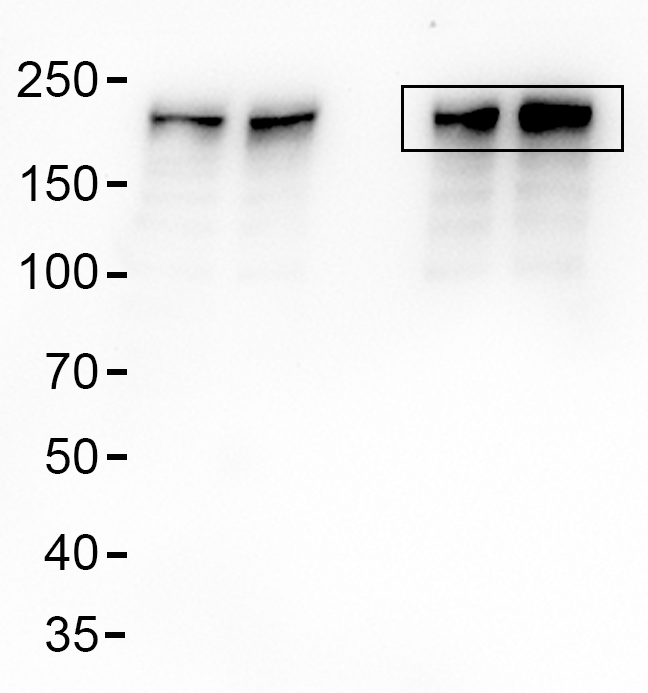

Supplement: Figure 1—source data 3. [file elife-97373-fig1-data3.zip › Figure 1-source data 2/Figure 1E/ZO-1.tif]

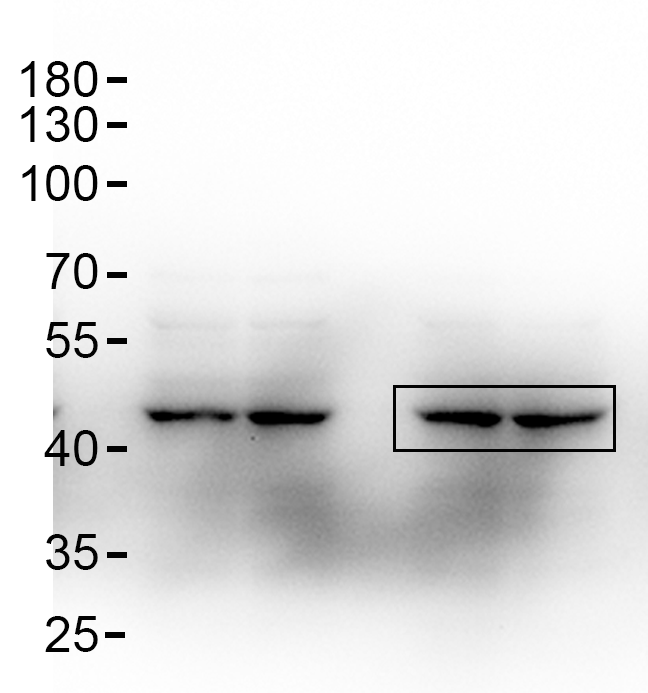

Supplement: Figure 1—source data 3. [file elife-97373-fig1-data3.zip › Figure 1-source data 2/Figure 1I/Actin.tif]

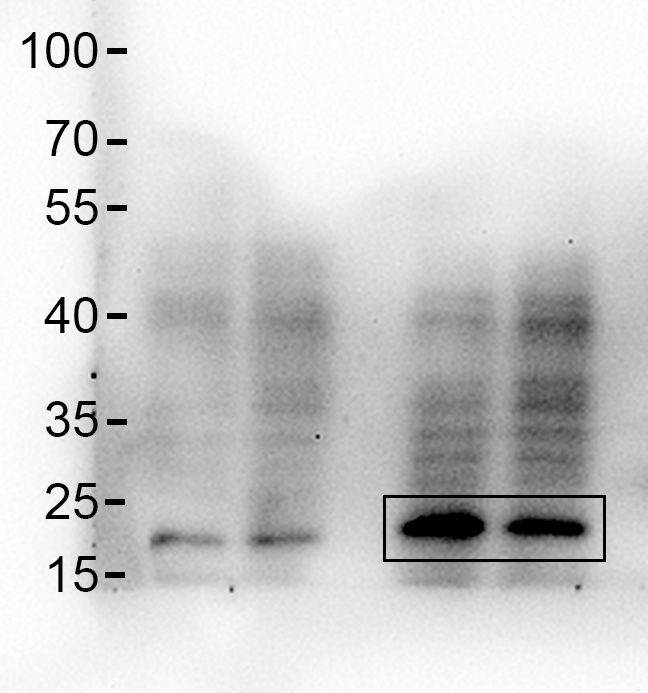

Supplement: Figure 1—source data 3. [file elife-97373-fig1-data3.zip › Figure 1-source data 2/Figure 1I/Claudin-1.tif]

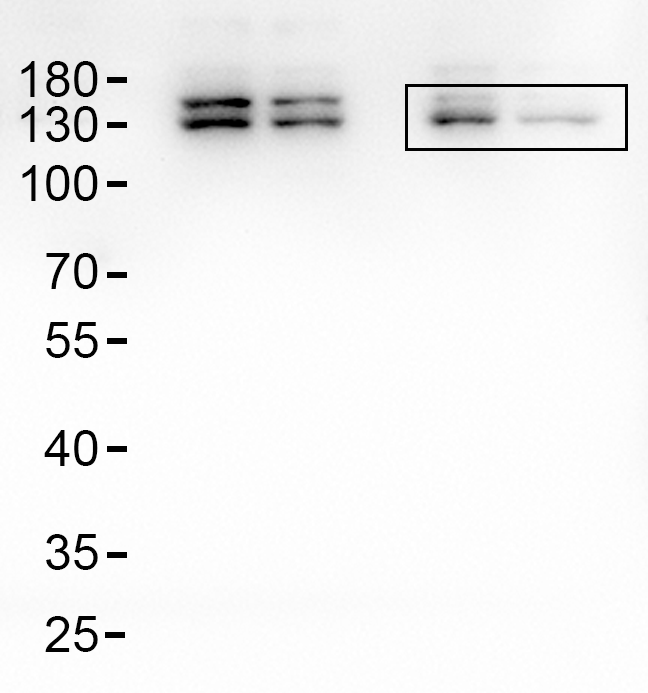

Supplement: Figure 1—source data 3. [file elife-97373-fig1-data3.zip › Figure 1-source data 2/Figure 1I/E-cadherin.tif]

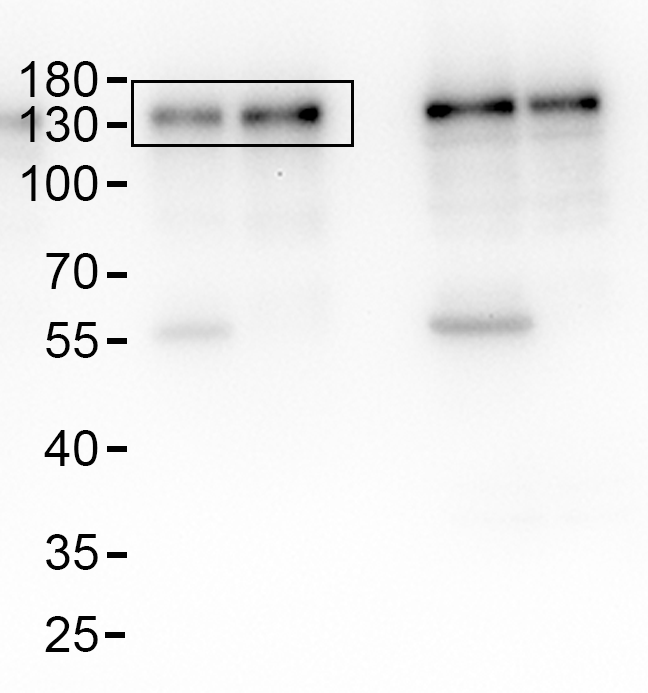

Supplement: Figure 1—source data 3. [file elife-97373-fig1-data3.zip › Figure 1-source data 2/Figure 1I/N-cadherin.tif]

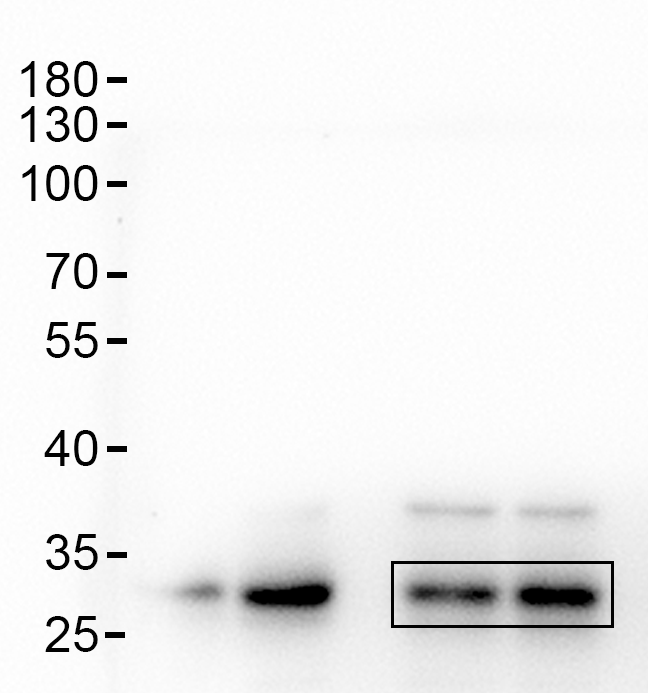

Supplement: Figure 1—source data 3. [file elife-97373-fig1-data3.zip › Figure 1-source data 2/Figure 1I/Slug.tif]

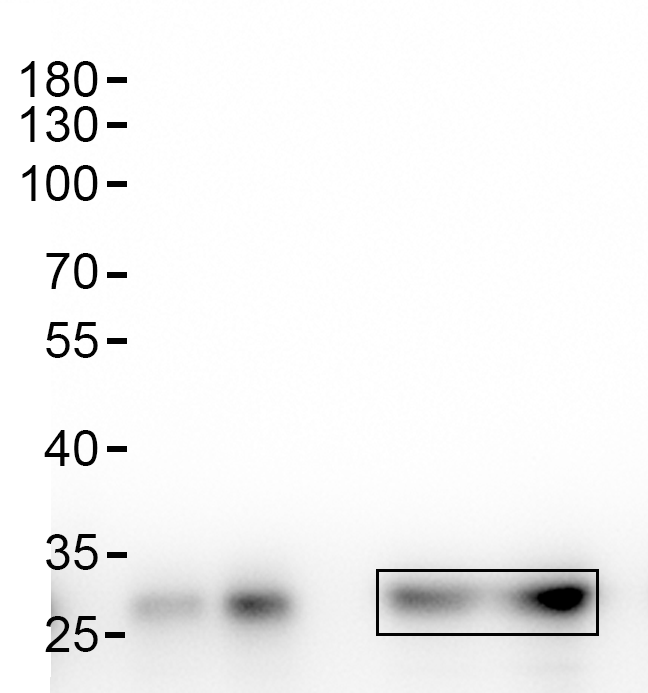

Supplement: Figure 1—source data 3. [file elife-97373-fig1-data3.zip › Figure 1-source data 2/Figure 1I/Snail.tif]

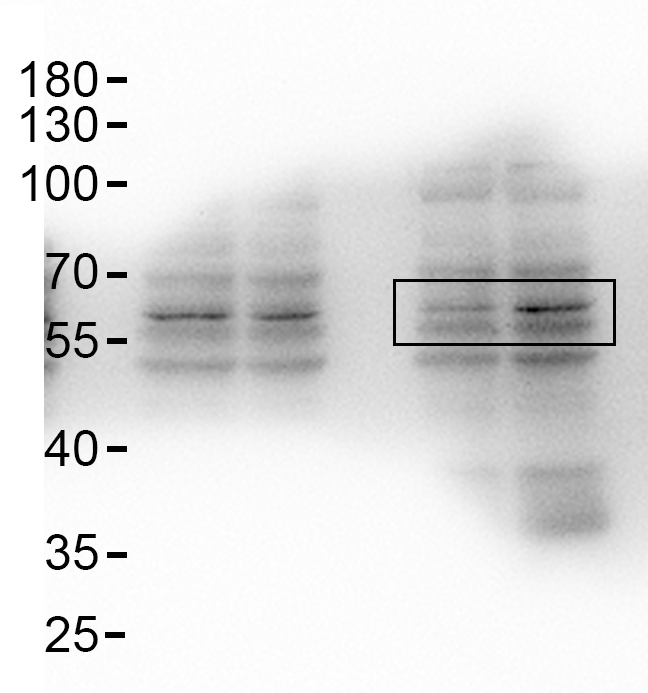

Supplement: Figure 1—source data 3. [file elife-97373-fig1-data3.zip › Figure 1-source data 2/Figure 1I/Vimentin.tif]

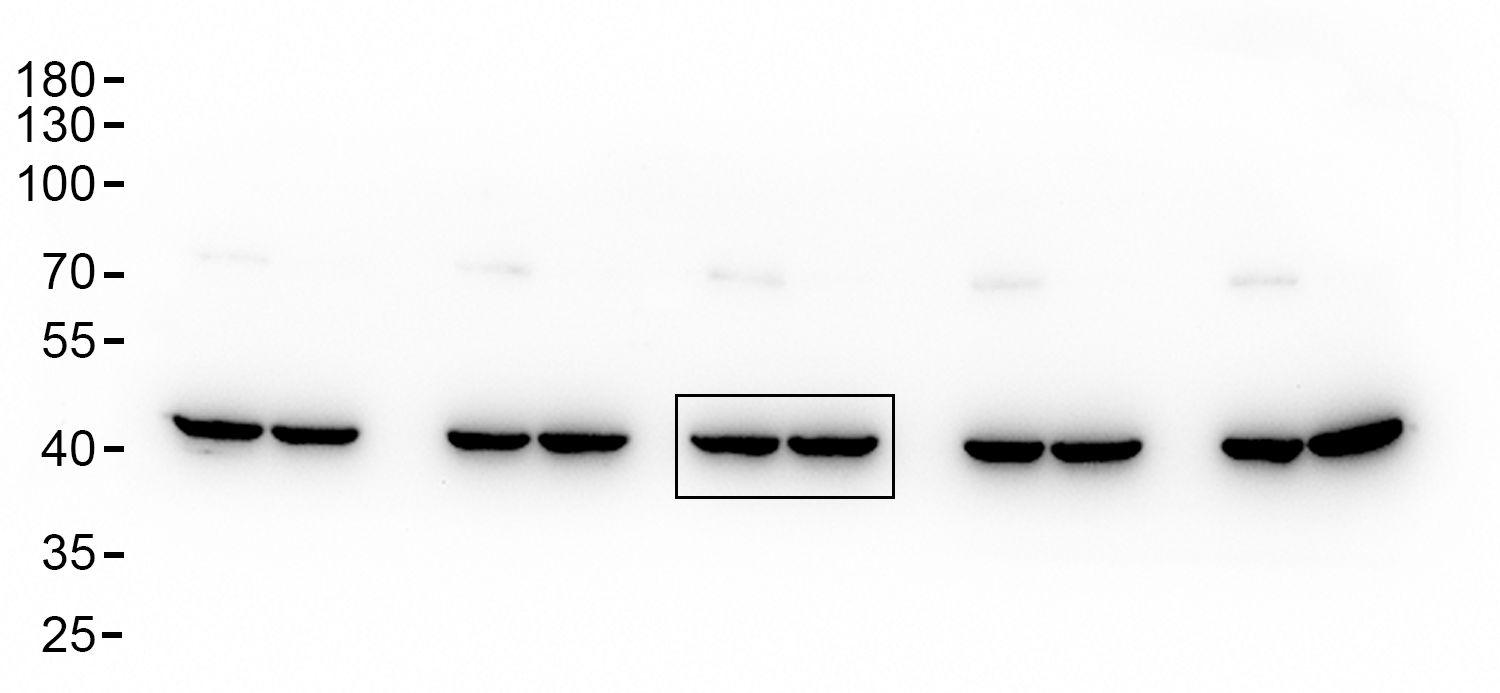

Supplement: Figure 1—figure supplement 3—source data 3. [file elife-97373-fig1-figsupp3-data3.zip › Figure 1-figure supplement 3-source data 2/Figure 1-figure supplement 3B/Actin.tif]

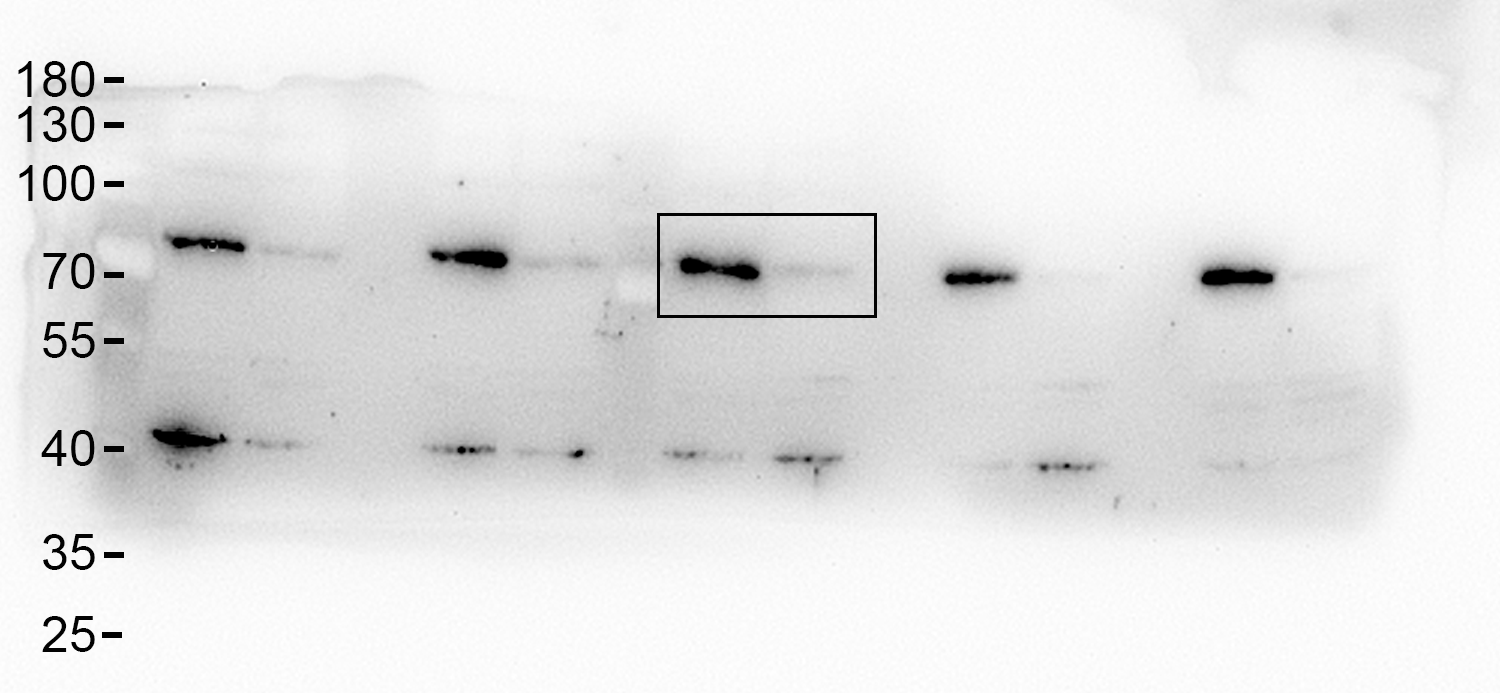

Supplement: Figure 1—figure supplement 3—source data 3. [file elife-97373-fig1-figsupp3-data3.zip › Figure 1-figure supplement 3-source data 2/Figure 1-figure supplement 3B/TAK1.tif]

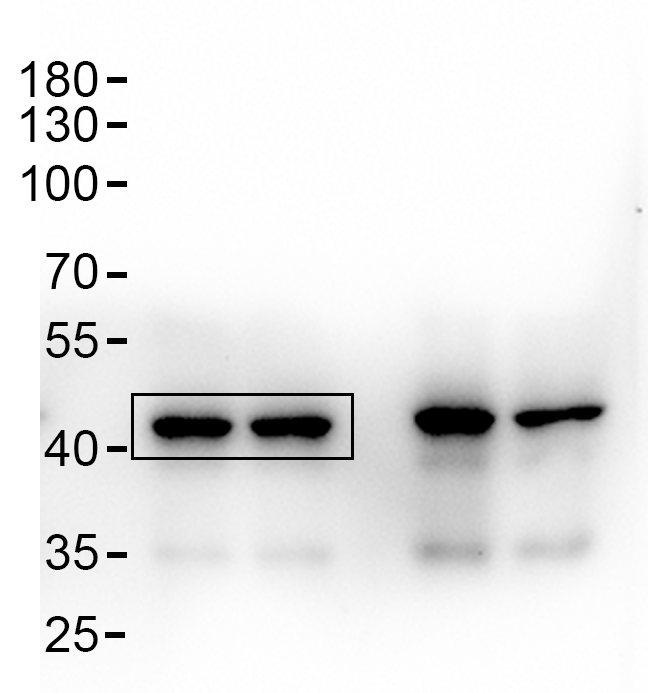

Supplement: Figure 1—figure supplement 3—source data 3. [file elife-97373-fig1-figsupp3-data3.zip › Figure 1-figure supplement 3-source data 2/Figure 1-figure supplement 3F/Actin.tif]

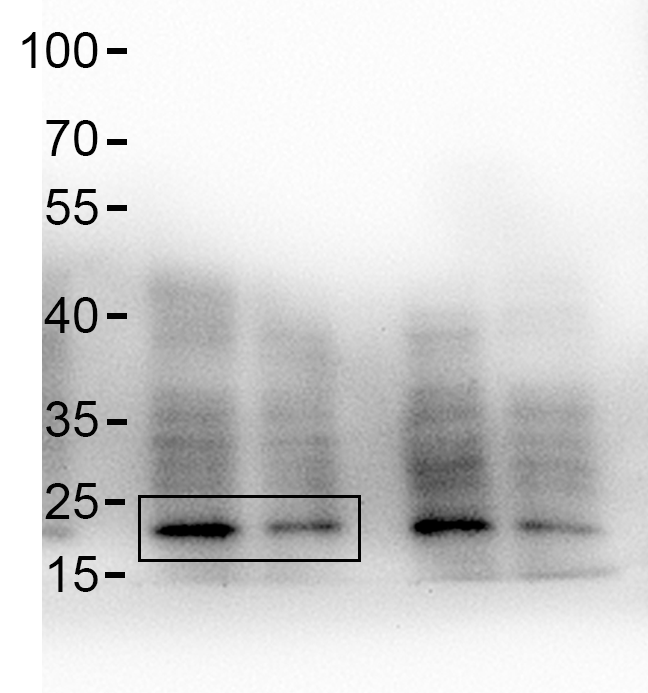

Supplement: Figure 1—figure supplement 3—source data 3. [file elife-97373-fig1-figsupp3-data3.zip › Figure 1-figure supplement 3-source data 2/Figure 1-figure supplement 3F/Claudin-1.tif]

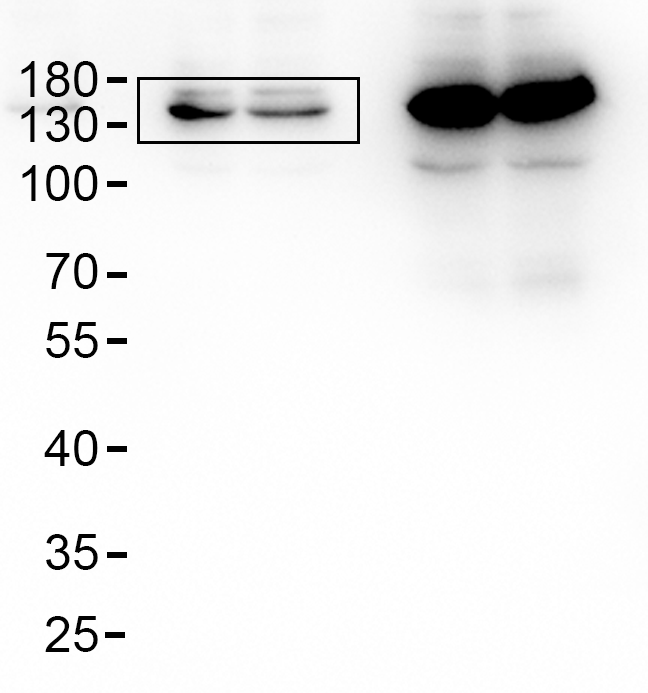

Supplement: Figure 1—figure supplement 3—source data 3. [file elife-97373-fig1-figsupp3-data3.zip › Figure 1-figure supplement 3-source data 2/Figure 1-figure supplement 3F/E-cadherin.tif]

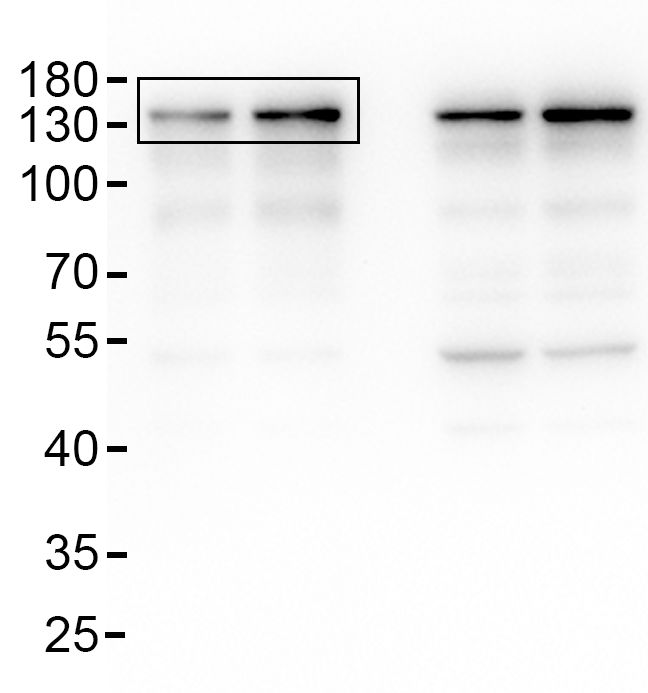

Supplement: Figure 1—figure supplement 3—source data 3. [file elife-97373-fig1-figsupp3-data3.zip › Figure 1-figure supplement 3-source data 2/Figure 1-figure supplement 3F/N-cadherin.tif]

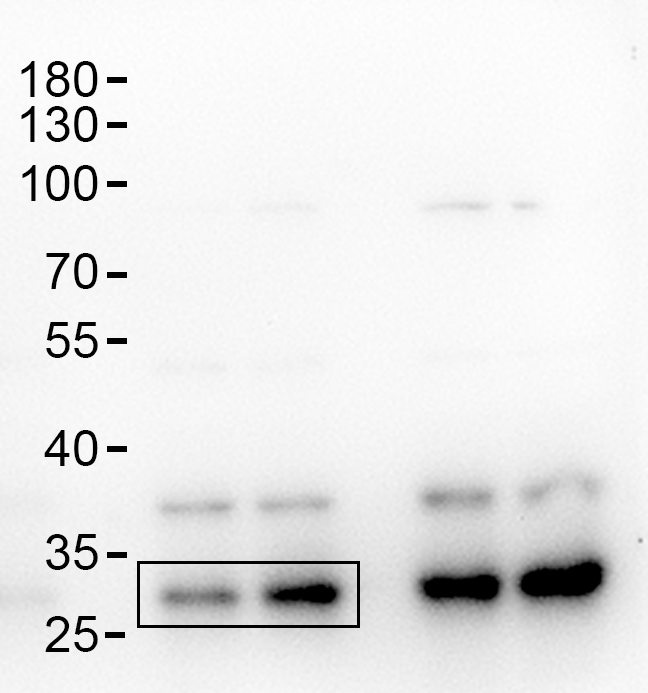

Supplement: Figure 1—figure supplement 3—source data 3. [file elife-97373-fig1-figsupp3-data3.zip › Figure 1-figure supplement 3-source data 2/Figure 1-figure supplement 3F/Slug.tif]

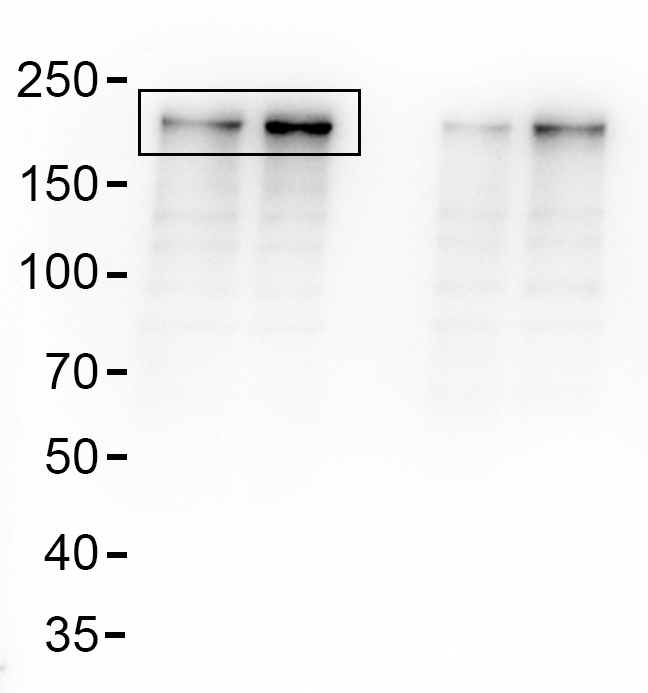

Supplement: Figure 1—figure supplement 3—source data 3. [file elife-97373-fig1-figsupp3-data3.zip › Figure 1-figure supplement 3-source data 2/Figure 1-figure supplement 3F/ZEB1.tif]

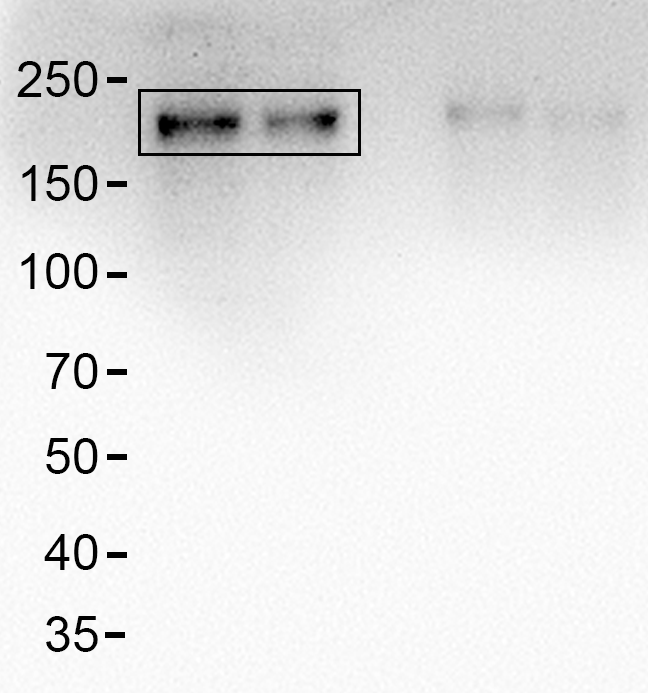

Supplement: Figure 1—figure supplement 3—source data 3. [file elife-97373-fig1-figsupp3-data3.zip › Figure 1-figure supplement 3-source data 2/Figure 1-figure supplement 3F/ZO-1.tif]

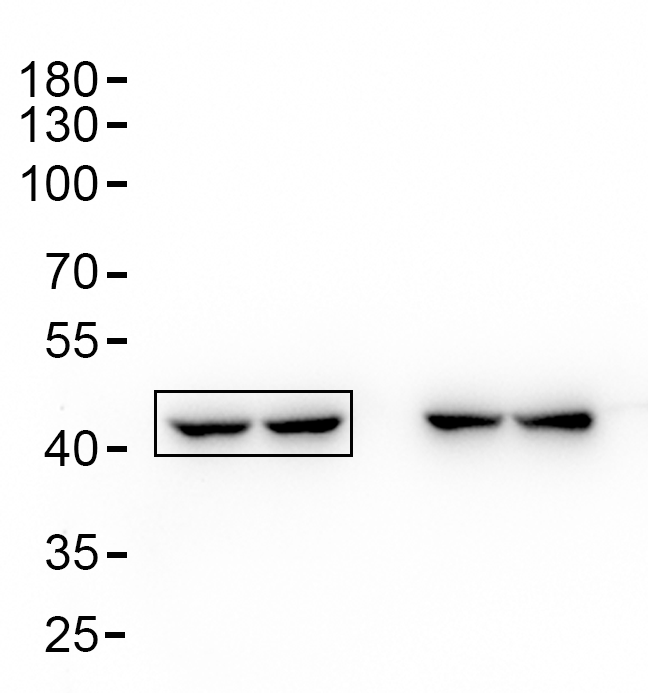

Supplement: Figure 1—figure supplement 4—source data 3. [file elife-97373-fig1-figsupp4-data3.zip › Figure 1-figure supplement 4-source data 2/Figure 1-figure supplement 4A/Actin.tif]

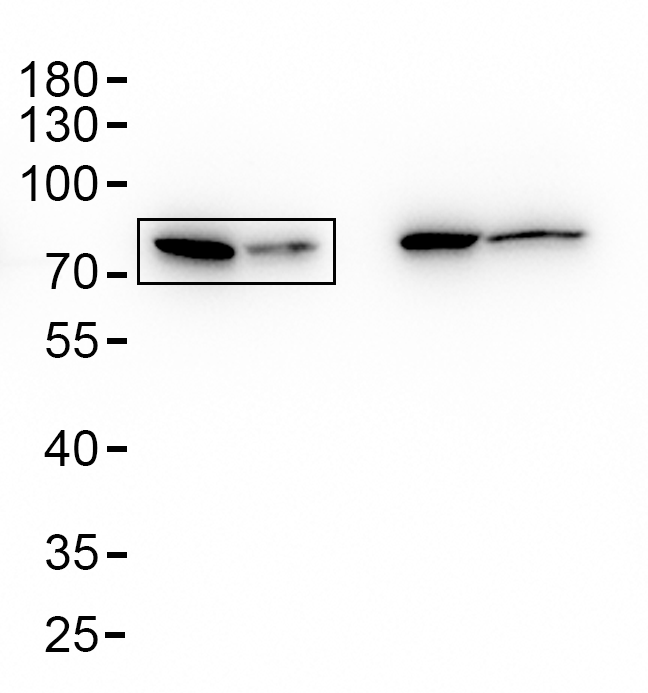

Supplement: Figure 1—figure supplement 4—source data 3. [file elife-97373-fig1-figsupp4-data3.zip › Figure 1-figure supplement 4-source data 2/Figure 1-figure supplement 4A/TAK1.tif]

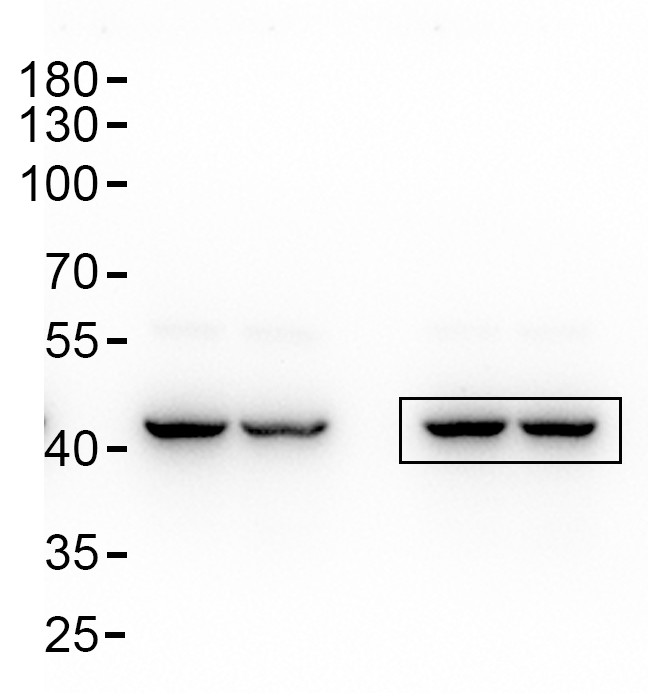

Supplement: Figure 1—figure supplement 4—source data 3. [file elife-97373-fig1-figsupp4-data3.zip › Figure 1-figure supplement 4-source data 2/Figure 1-figure supplement 4E/Actin.tif]

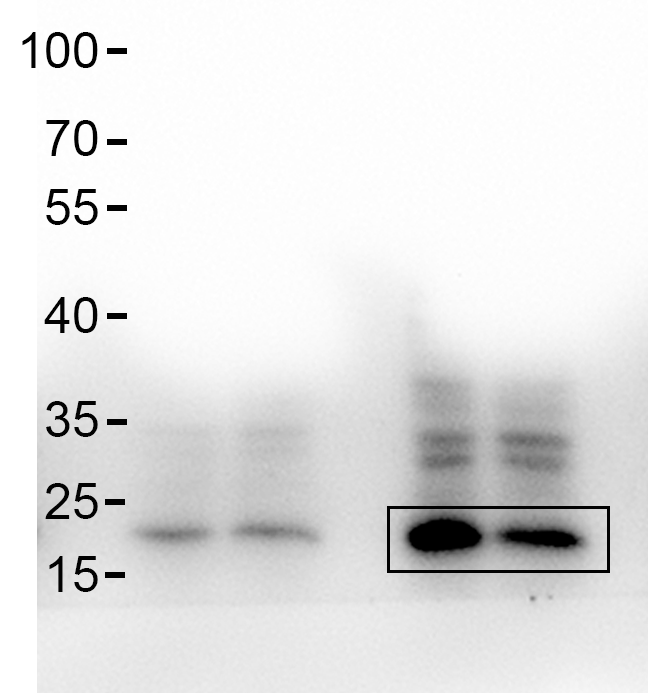

Supplement: Figure 1—figure supplement 4—source data 3. [file elife-97373-fig1-figsupp4-data3.zip › Figure 1-figure supplement 4-source data 2/Figure 1-figure supplement 4E/Claudin-1.tif]

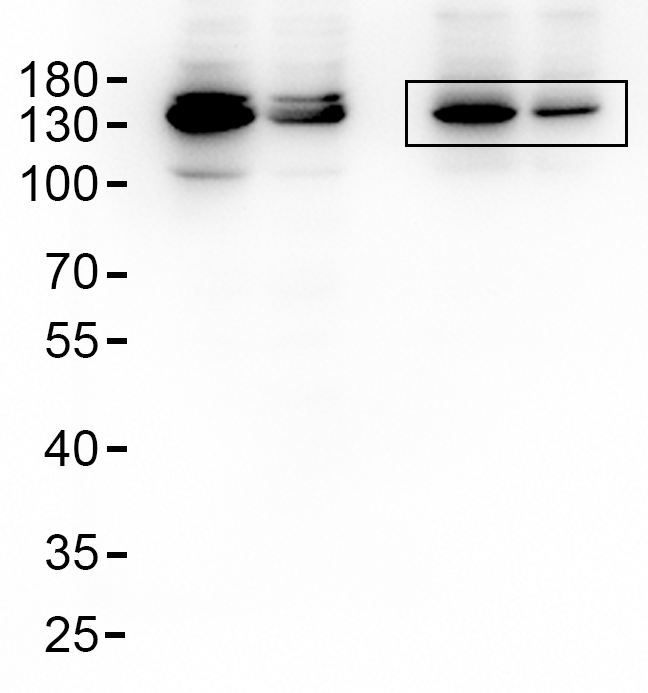

Supplement: Figure 1—figure supplement 4—source data 3. [file elife-97373-fig1-figsupp4-data3.zip › Figure 1-figure supplement 4-source data 2/Figure 1-figure supplement 4E/E-cadherin.tif]

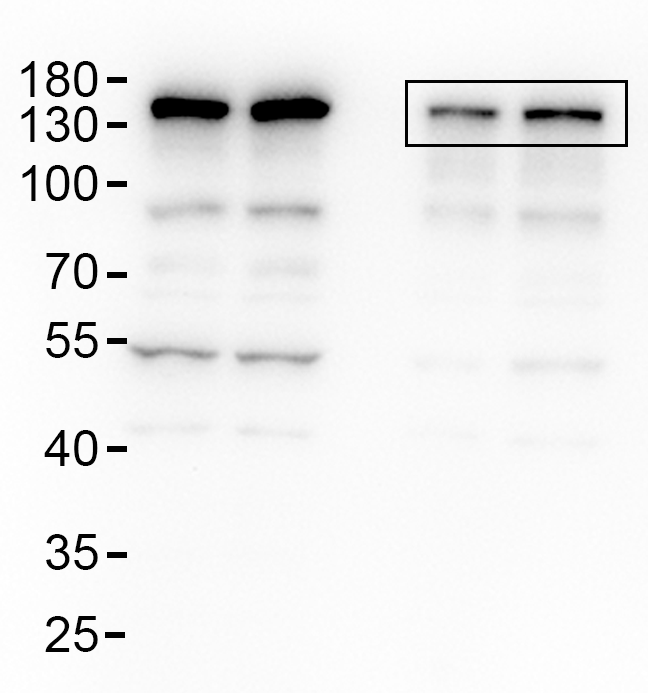

Supplement: Figure 1—figure supplement 4—source data 3. [file elife-97373-fig1-figsupp4-data3.zip › Figure 1-figure supplement 4-source data 2/Figure 1-figure supplement 4E/N-cadherin.tif]

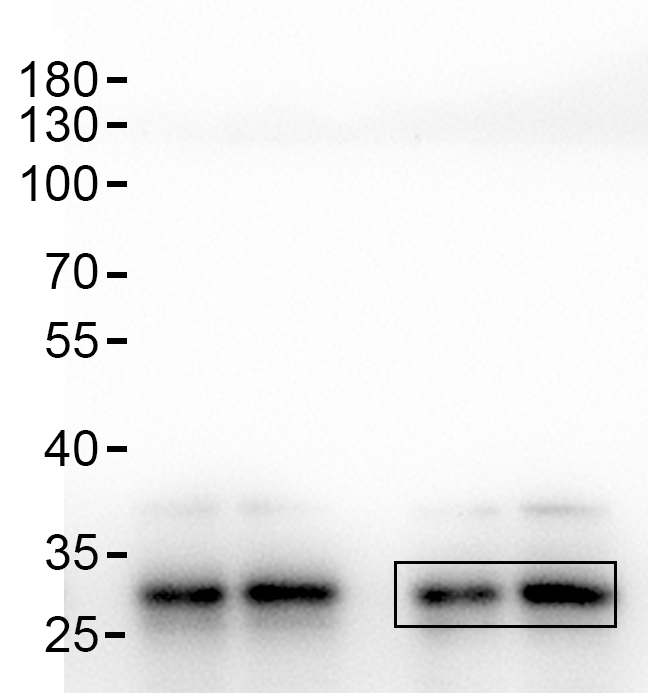

Supplement: Figure 1—figure supplement 4—source data 3. [file elife-97373-fig1-figsupp4-data3.zip › Figure 1-figure supplement 4-source data 2/Figure 1-figure supplement 4E/Slug.tif]

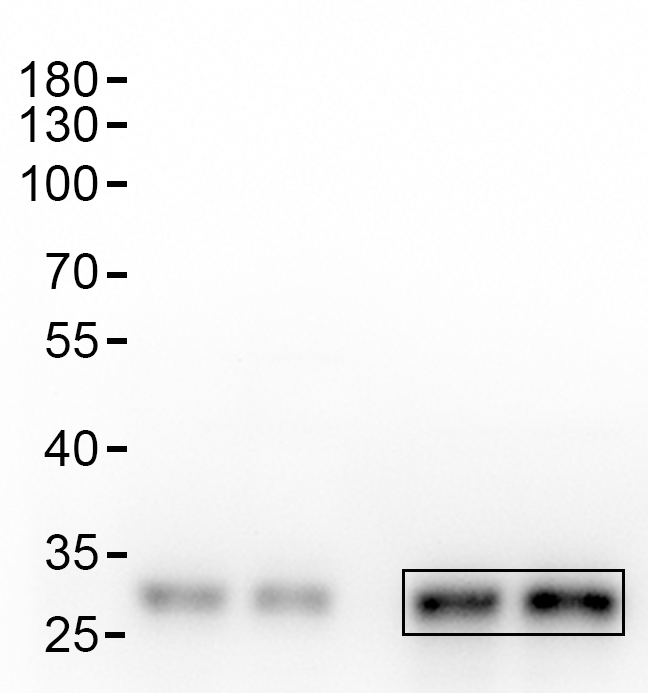

Supplement: Figure 1—figure supplement 4—source data 3. [file elife-97373-fig1-figsupp4-data3.zip › Figure 1-figure supplement 4-source data 2/Figure 1-figure supplement 4E/Snail.tif]

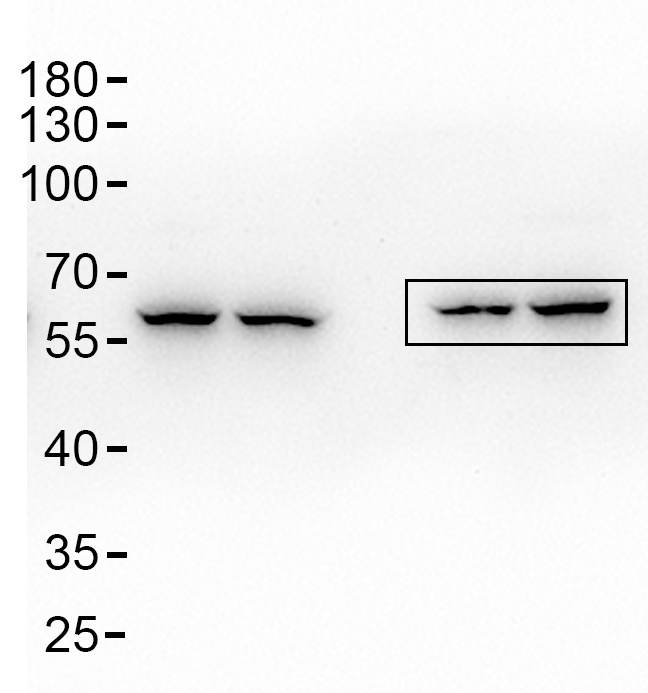

Supplement: Figure 1—figure supplement 4—source data 3. [file elife-97373-fig1-figsupp4-data3.zip › Figure 1-figure supplement 4-source data 2/Figure 1-figure supplement 4E/Vimentin.tif]

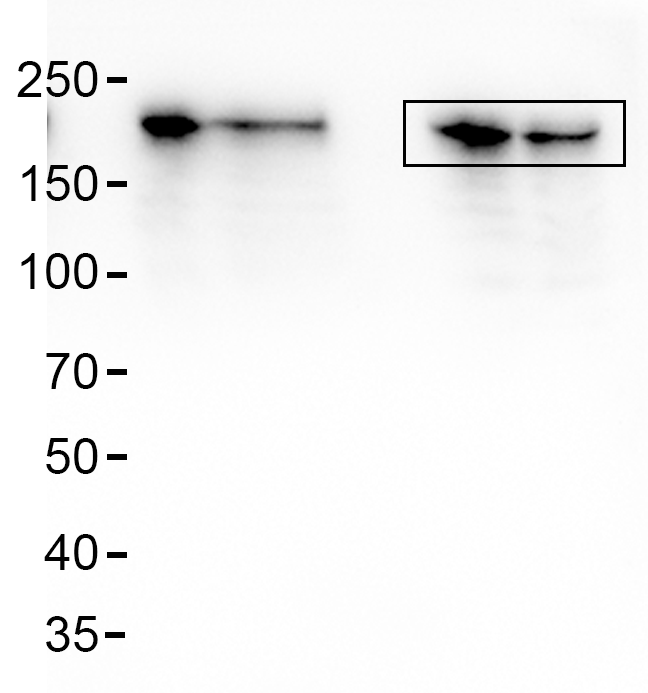

Supplement: Figure 1—figure supplement 4—source data 3. [file elife-97373-fig1-figsupp4-data3.zip › Figure 1-figure supplement 4-source data 2/Figure 1-figure supplement 4E/ZO-1.tif]

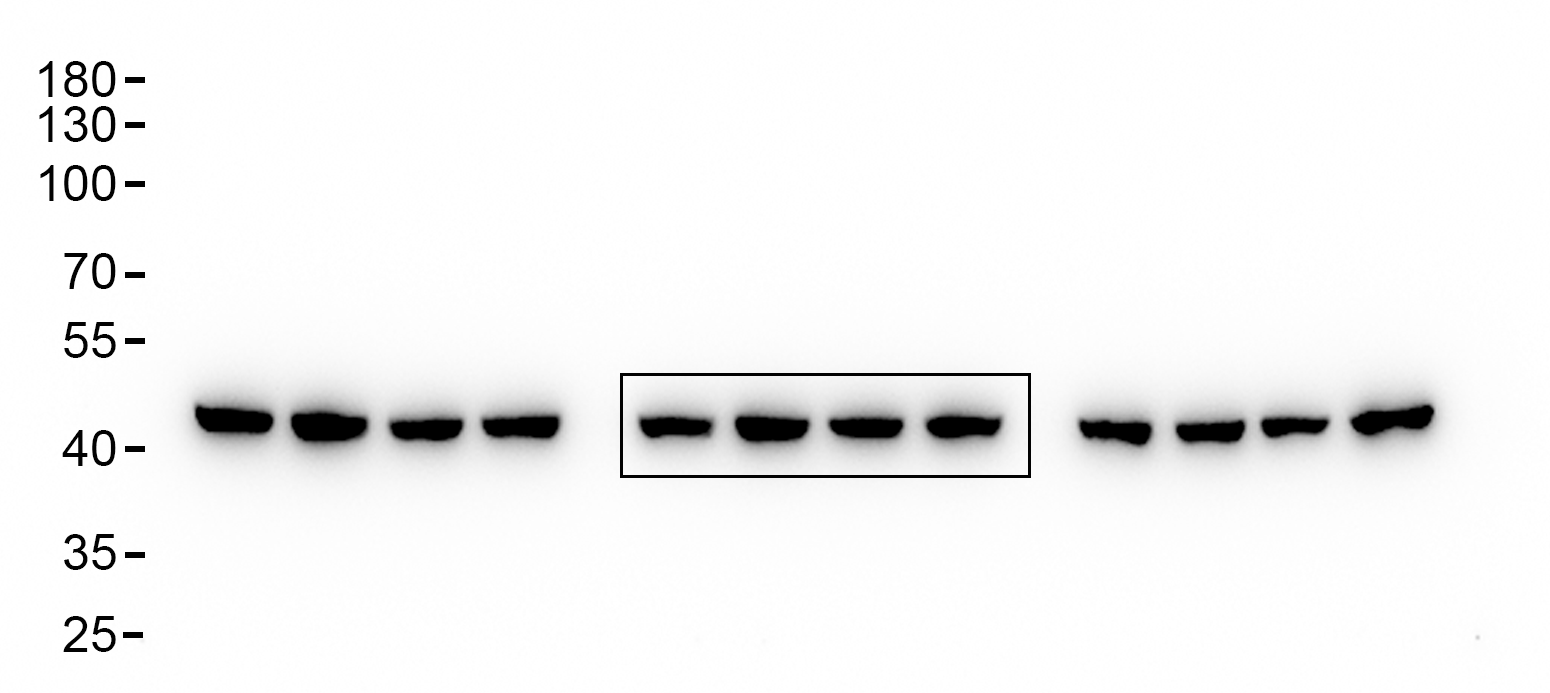

Supplement: Figure 2—source data 3. [file elife-97373-fig2-data3.zip › Figure 2-source data 2/Figure 2B/actin.tif]

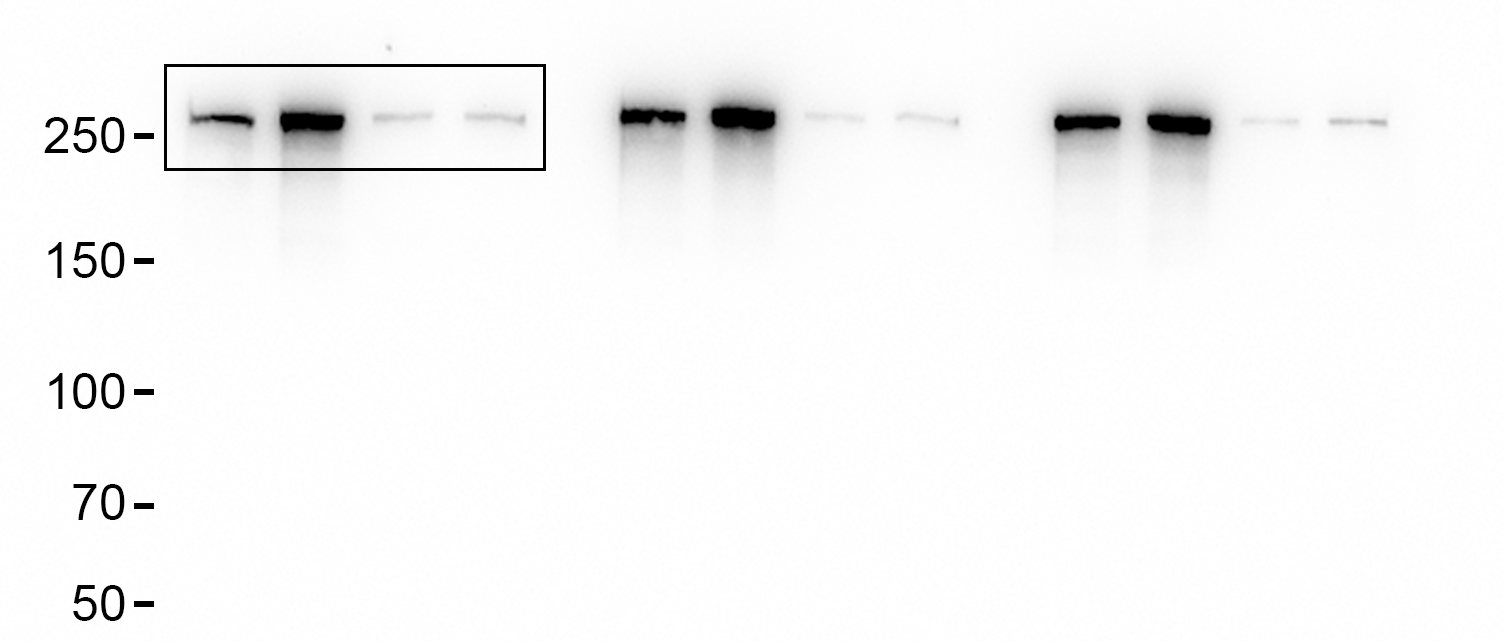

Supplement: Figure 2—source data 3. [file elife-97373-fig2-data3.zip › Figure 2-source data 2/Figure 2B/p-plce1.tif]

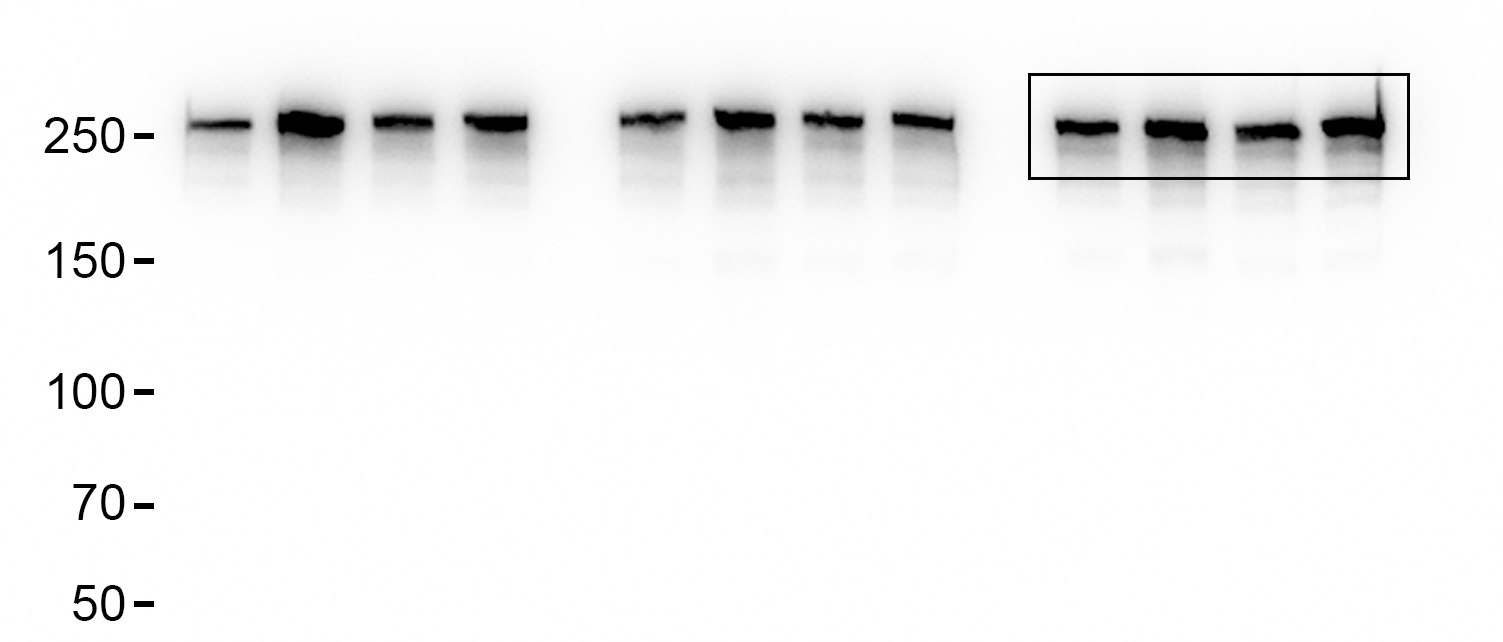

Supplement: Figure 2—source data 3. [file elife-97373-fig2-data3.zip › Figure 2-source data 2/Figure 2B/plce1.tif]

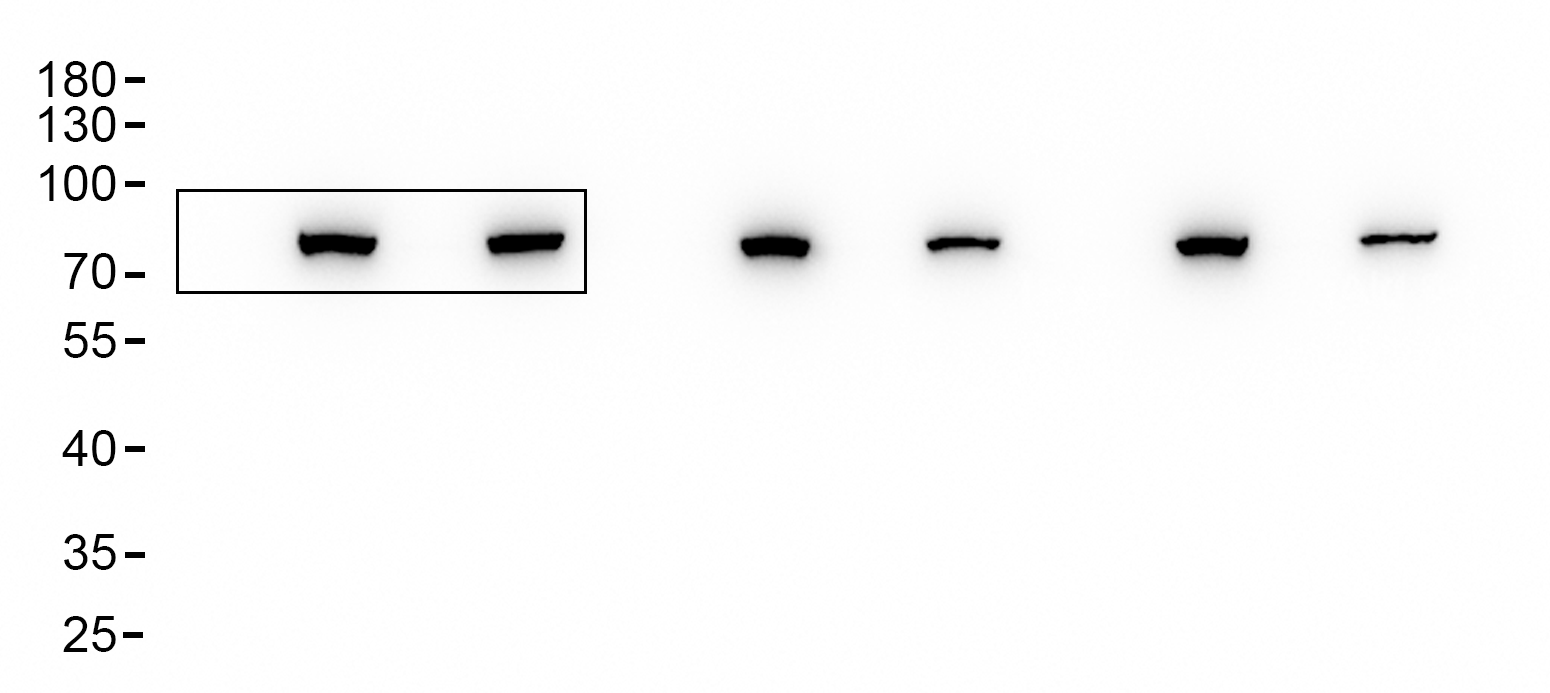

Supplement: Figure 2—source data 3. [file elife-97373-fig2-data3.zip › Figure 2-source data 2/Figure 2B/tak1.tif]

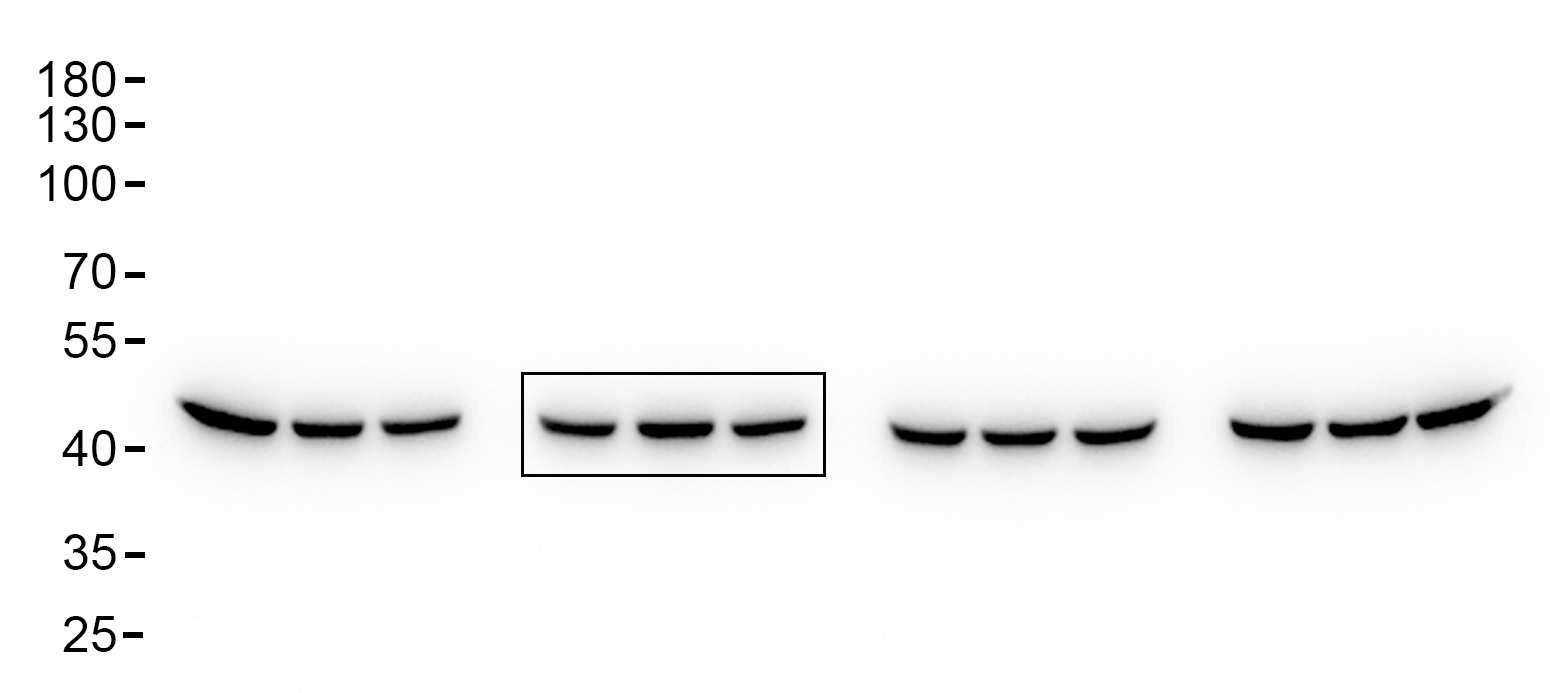

Supplement: Figure 2—source data 3. [file elife-97373-fig2-data3.zip › Figure 2-source data 2/Figure 2C/actin.tif]

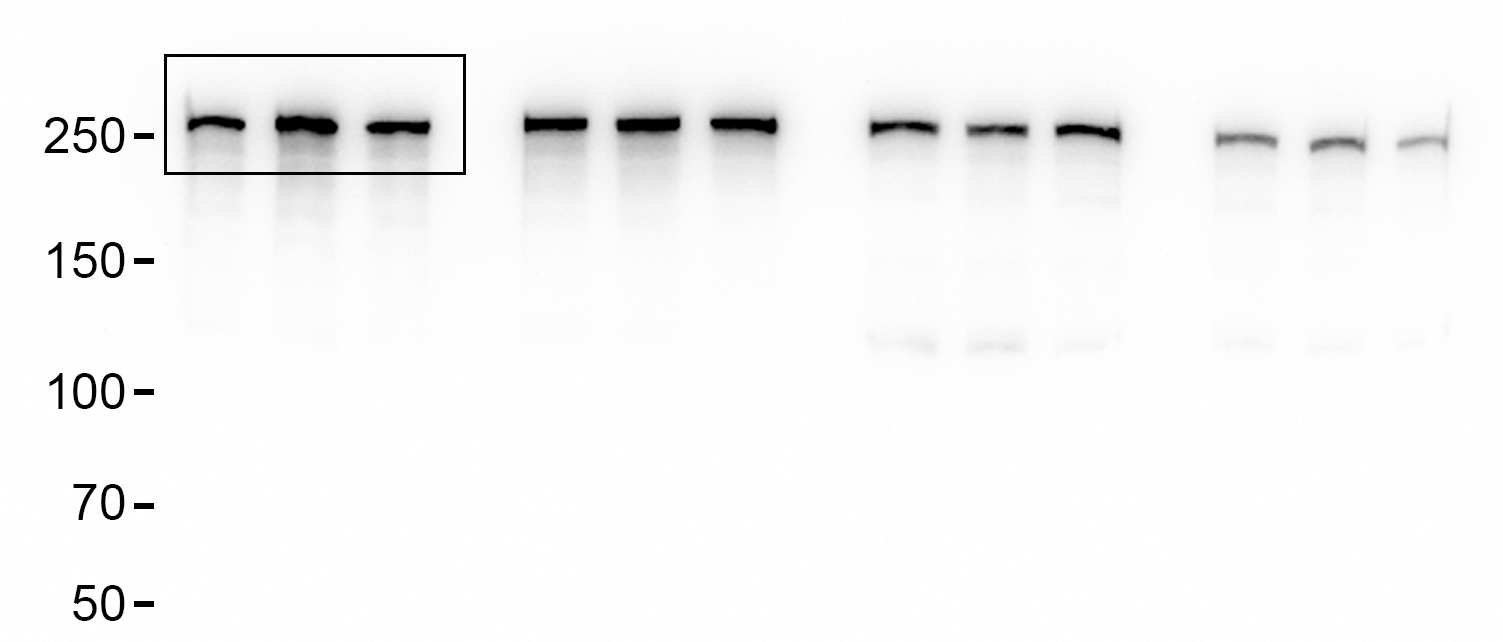

Supplement: Figure 2—source data 3. [file elife-97373-fig2-data3.zip › Figure 2-source data 2/Figure 2C/p-plce1.tif]

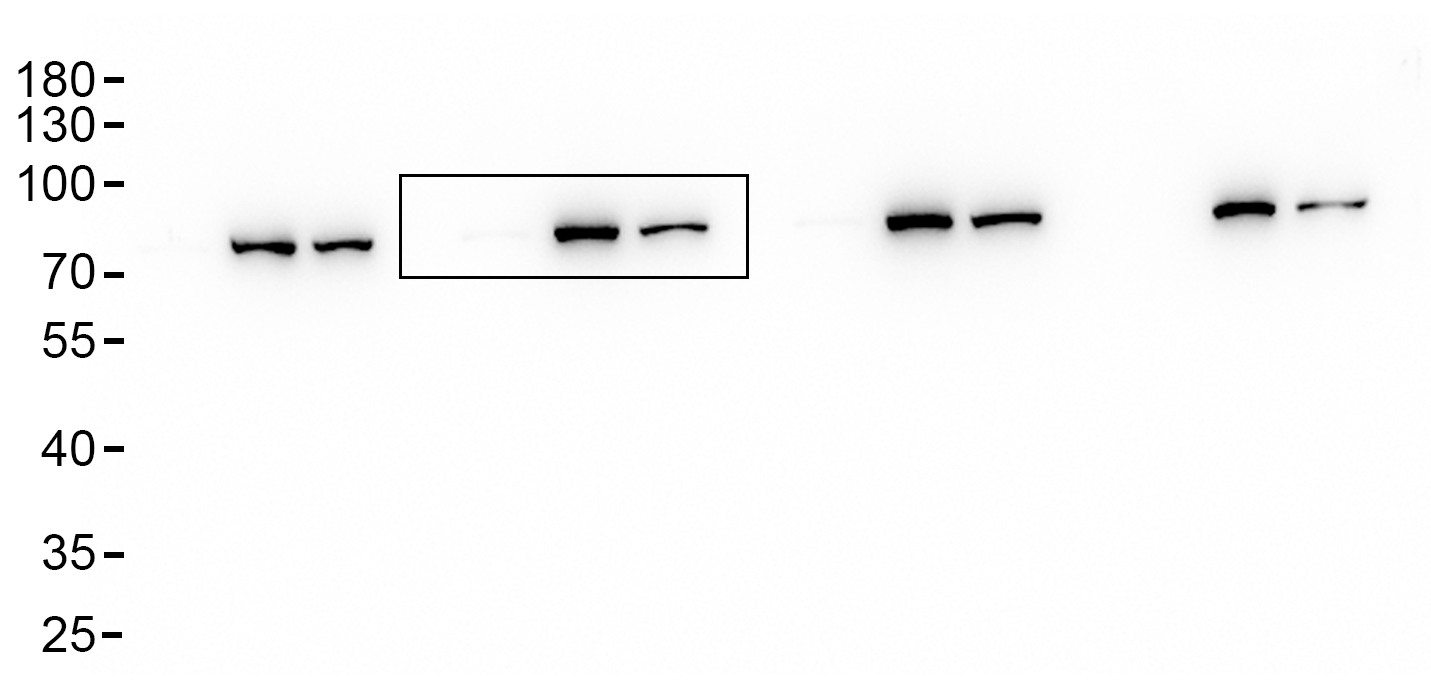

Supplement: Figure 2—source data 3. [file elife-97373-fig2-data3.zip › Figure 2-source data 2/Figure 2C/P-TAK1.tif]

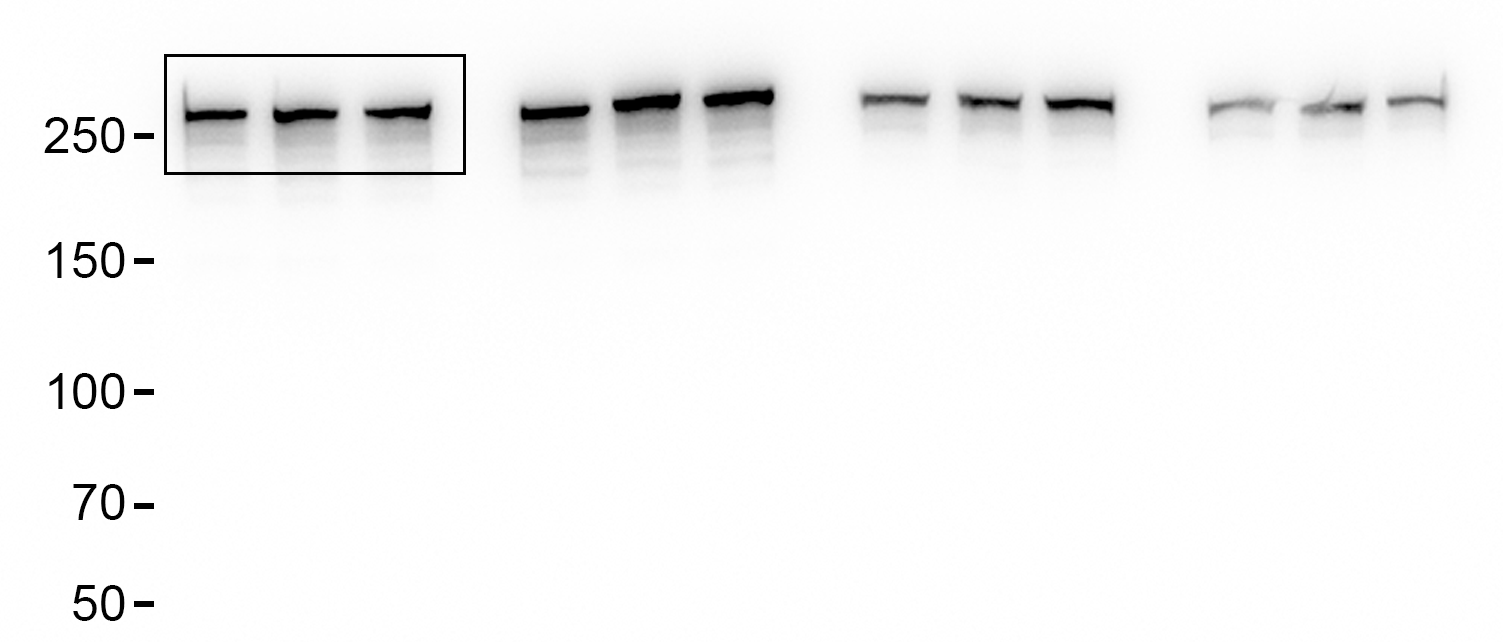

Supplement: Figure 2—source data 3. [file elife-97373-fig2-data3.zip › Figure 2-source data 2/Figure 2C/plce1.tif]

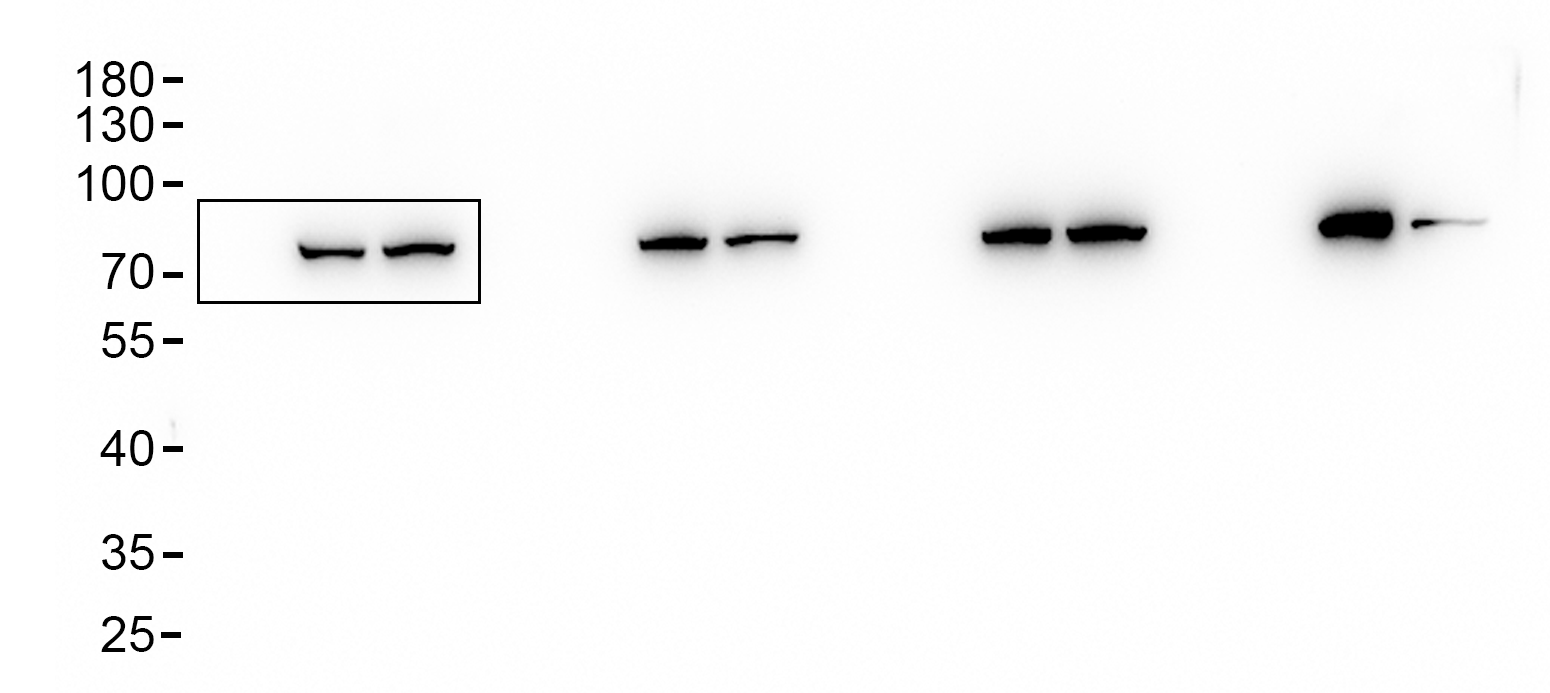

Supplement: Figure 2—source data 3. [file elife-97373-fig2-data3.zip › Figure 2-source data 2/Figure 2C/TAK1.tif]

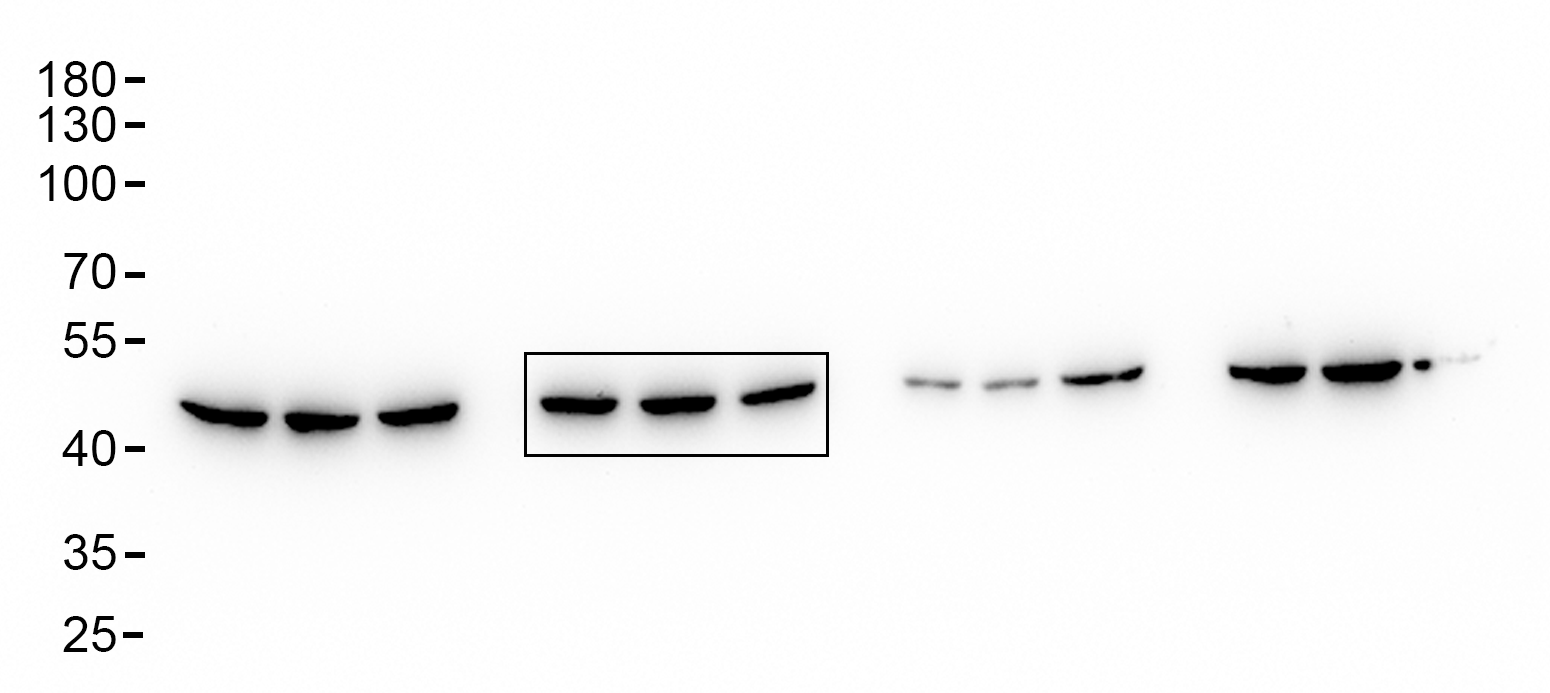

Supplement: Figure 2—source data 3. [file elife-97373-fig2-data3.zip › Figure 2-source data 2/Figure 2D/actin.tif]

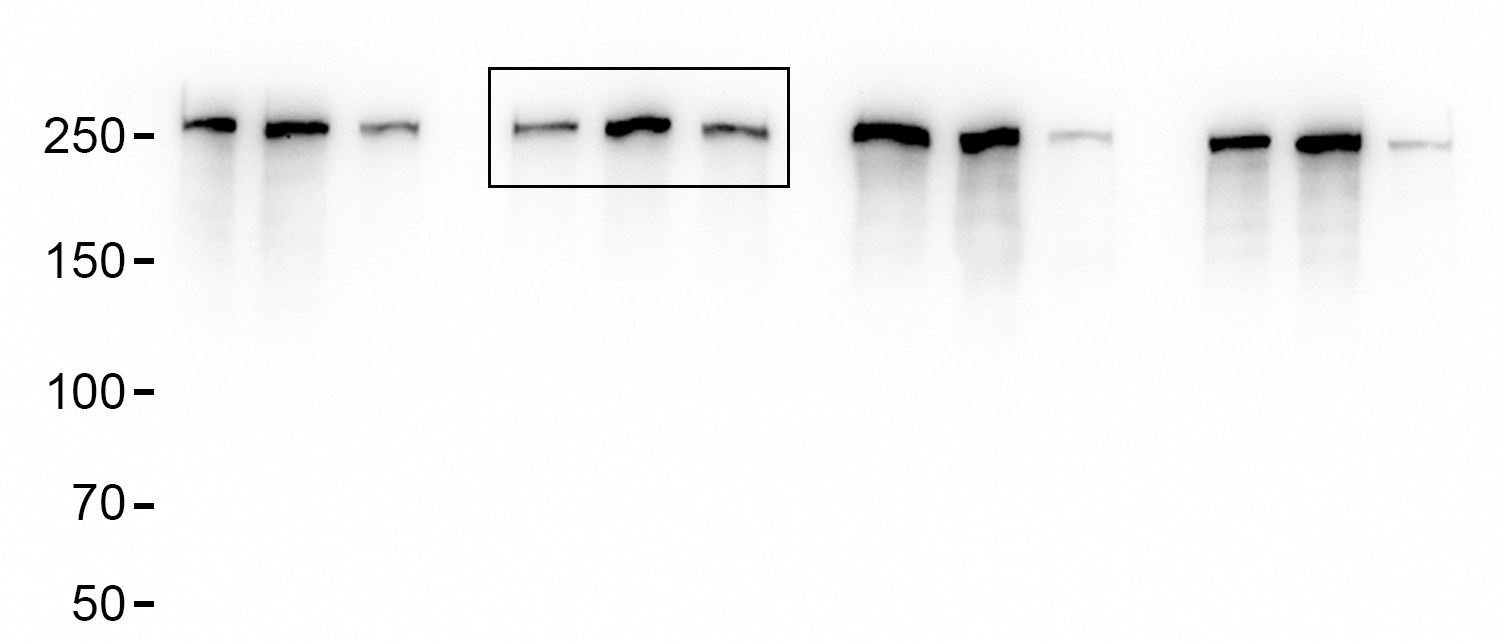

Supplement: Figure 2—source data 3. [file elife-97373-fig2-data3.zip › Figure 2-source data 2/Figure 2D/p-plce1.tif]

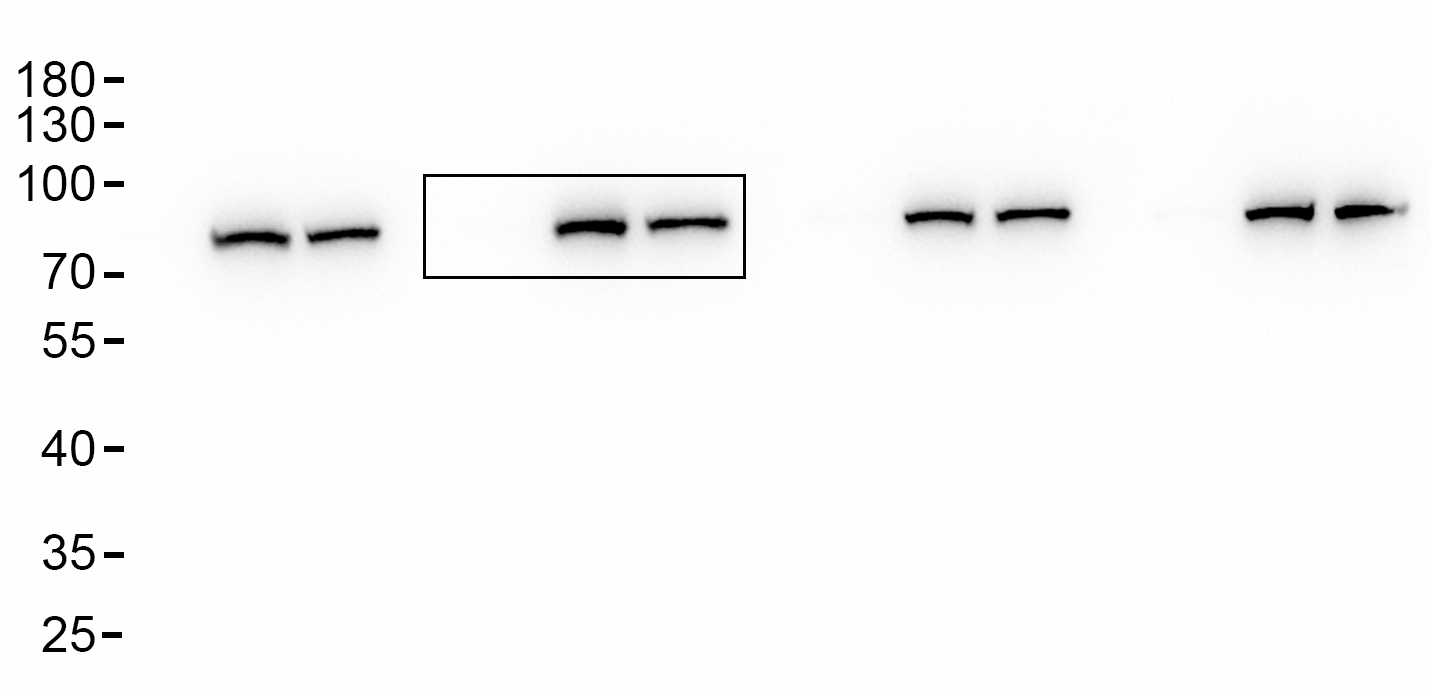

Supplement: Figure 2—source data 3. [file elife-97373-fig2-data3.zip › Figure 2-source data 2/Figure 2D/P-TAK1.tif]

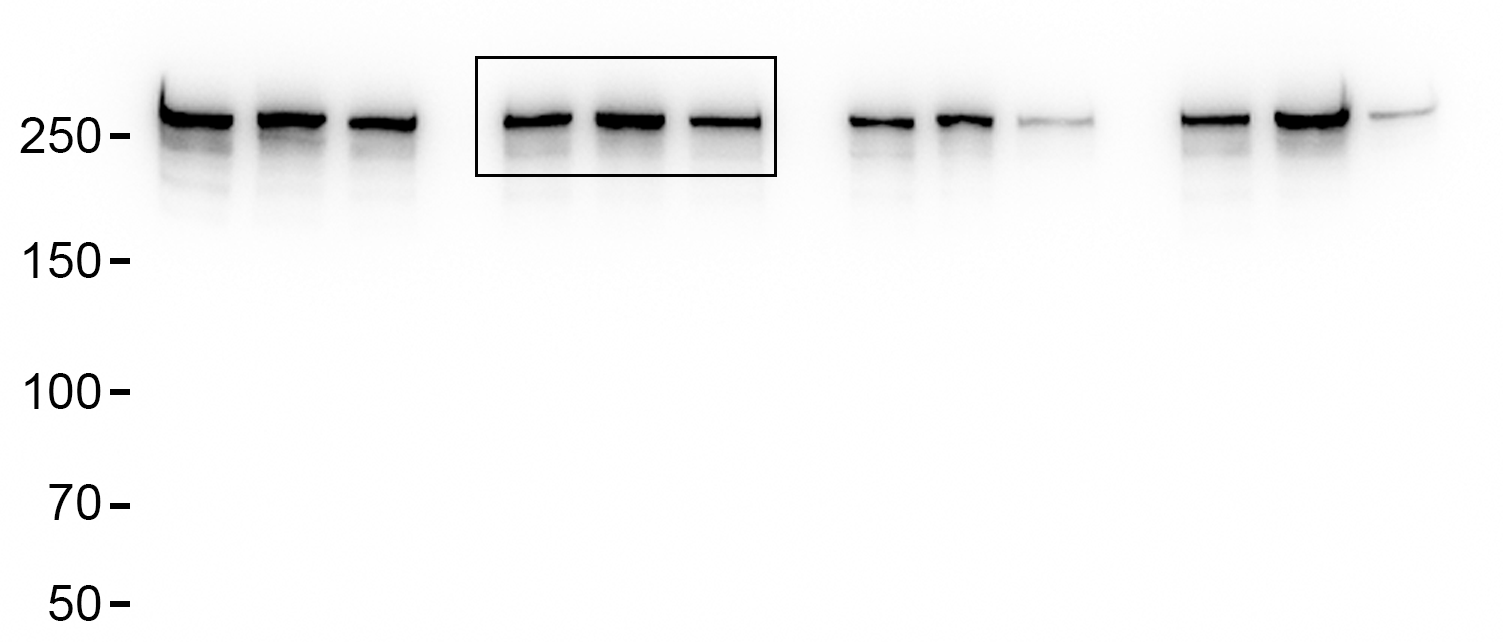

Supplement: Figure 2—source data 3. [file elife-97373-fig2-data3.zip › Figure 2-source data 2/Figure 2D/plce1.tif]

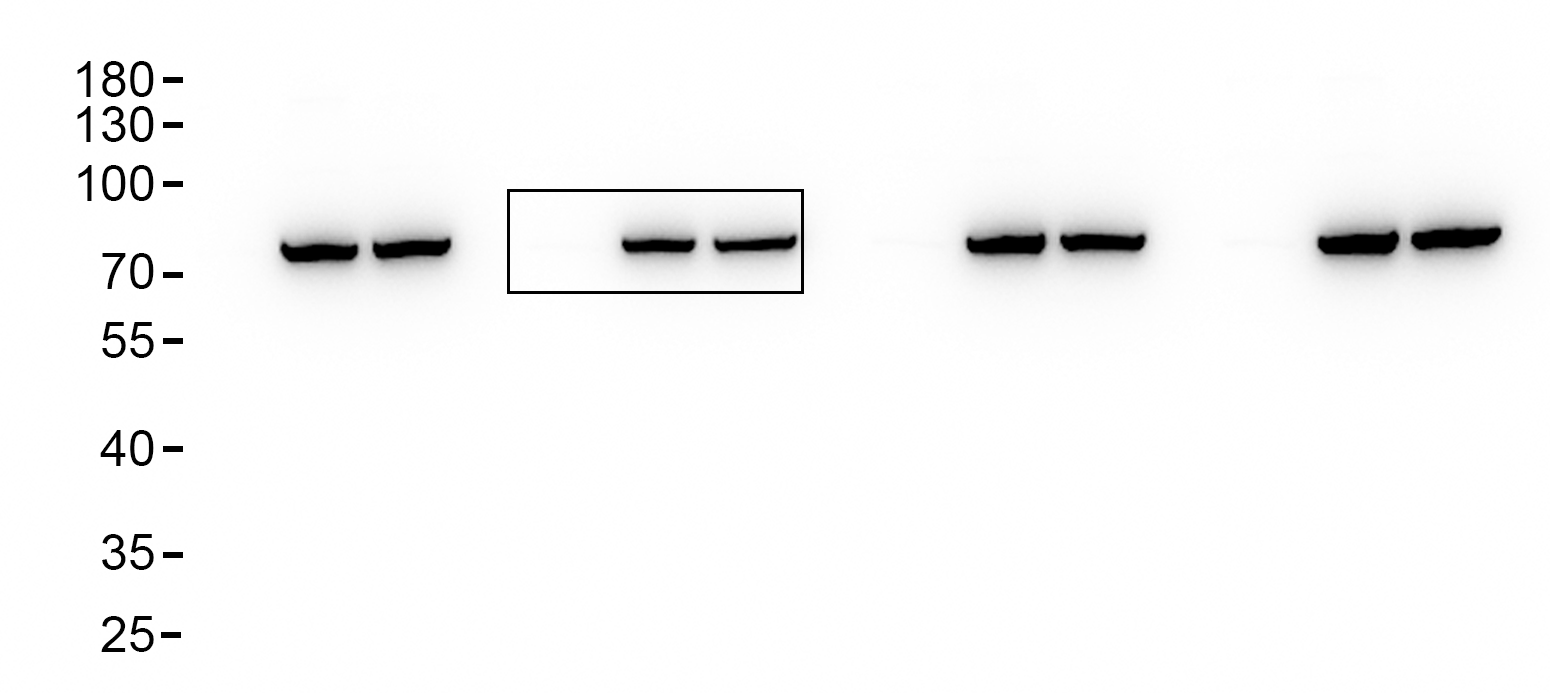

Supplement: Figure 2—source data 3. [file elife-97373-fig2-data3.zip › Figure 2-source data 2/Figure 2D/TAK1.tif]

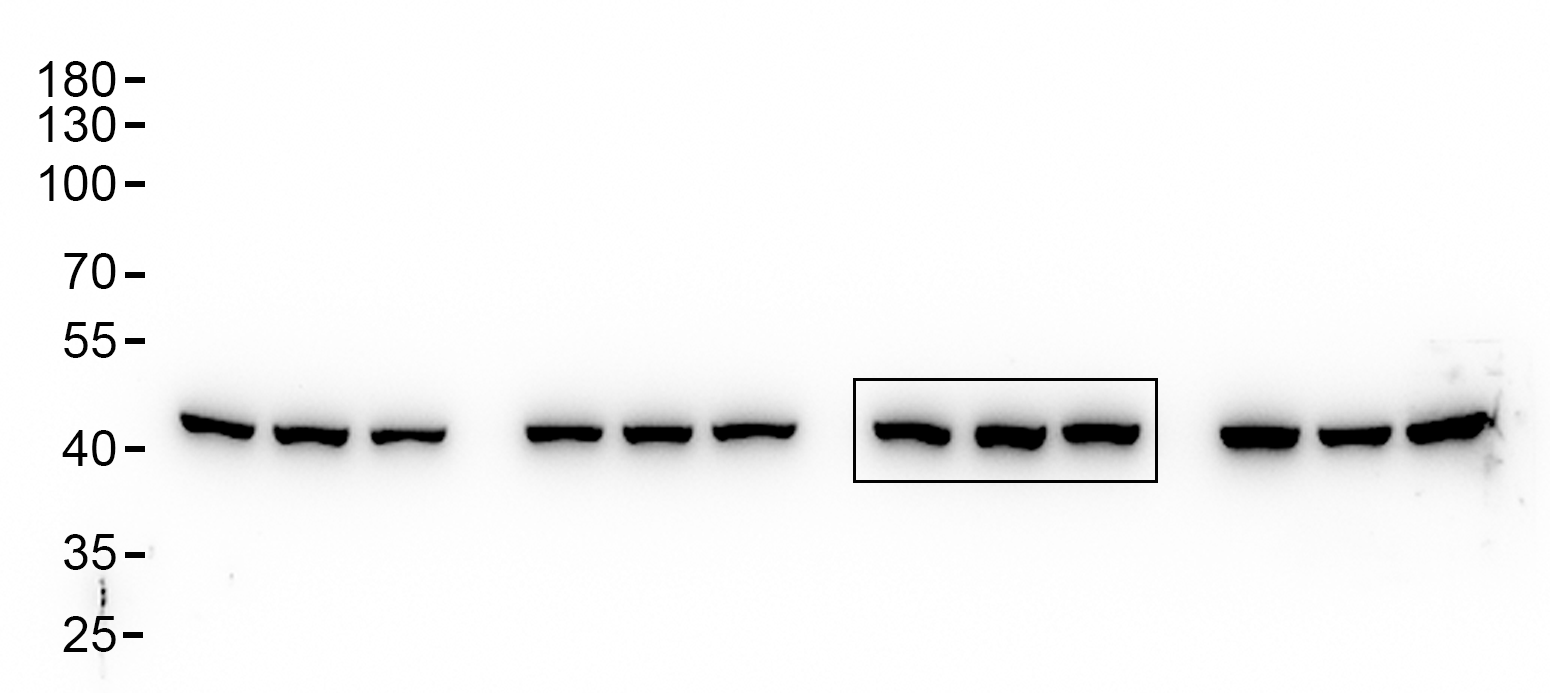

Supplement: Figure 2—source data 3. [file elife-97373-fig2-data3.zip › Figure 2-source data 2/Figure 2E/actin.tif]

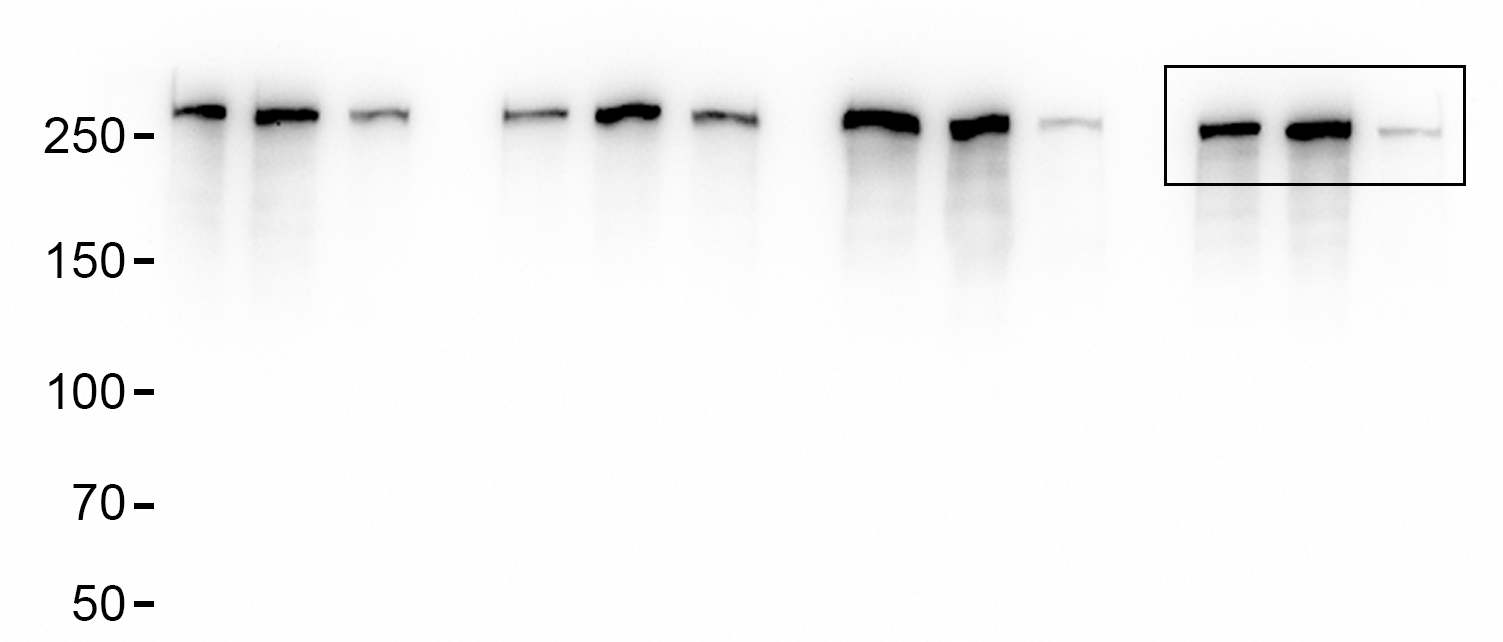

Supplement: Figure 2—source data 3. [file elife-97373-fig2-data3.zip › Figure 2-source data 2/Figure 2E/p-plce1.tif]

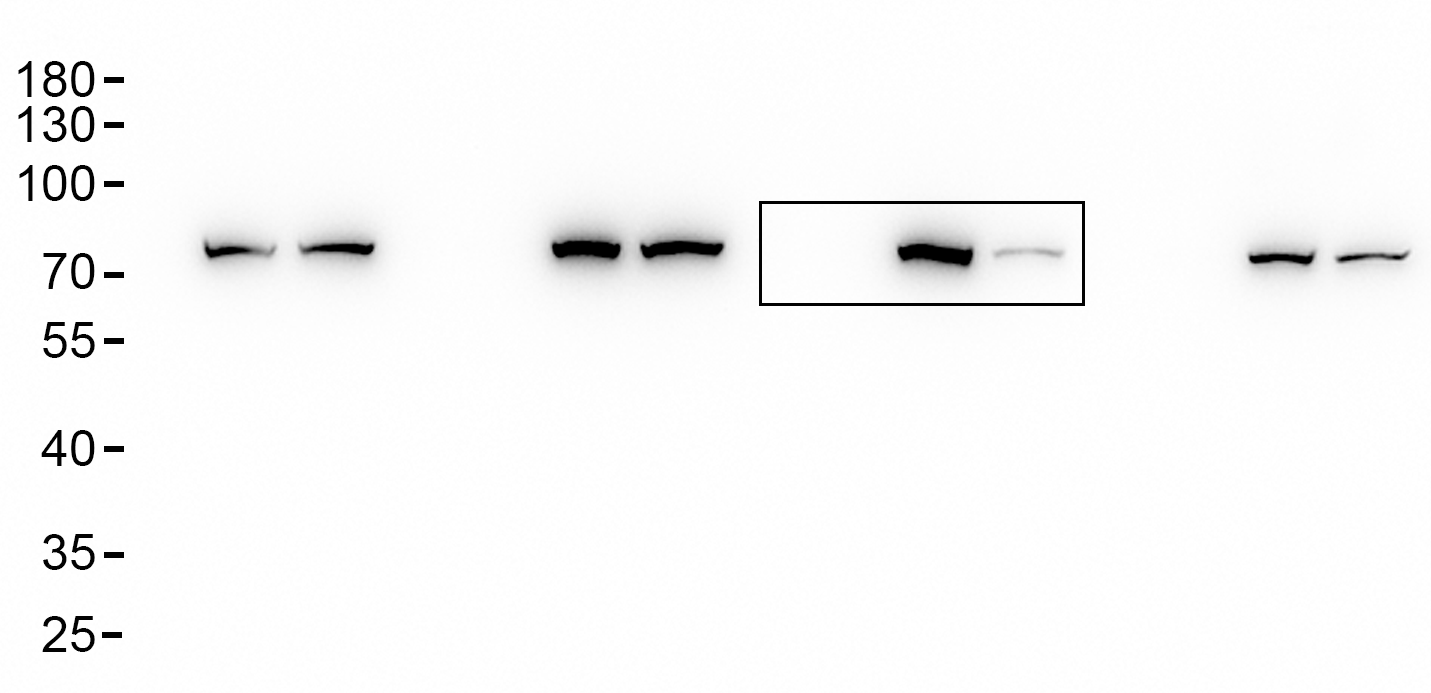

Supplement: Figure 2—source data 3. [file elife-97373-fig2-data3.zip › Figure 2-source data 2/Figure 2E/P-TAK1.tif]

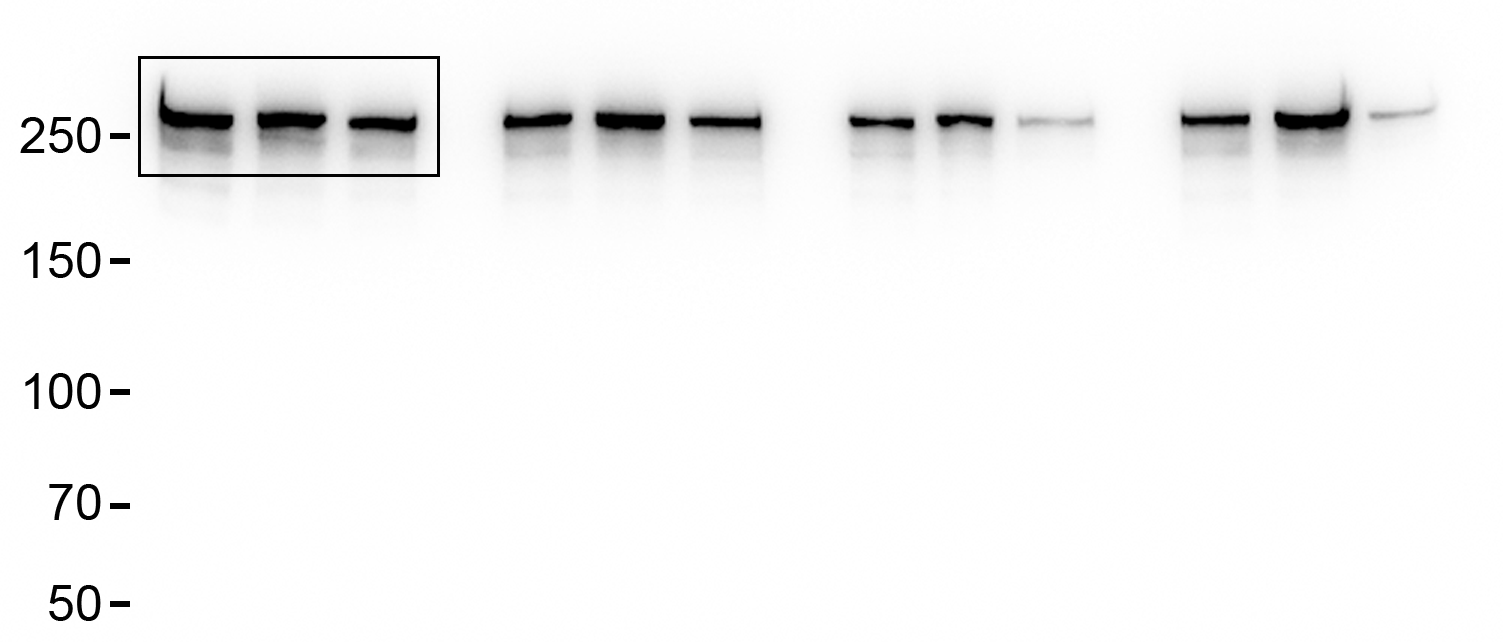

Supplement: Figure 2—source data 3. [file elife-97373-fig2-data3.zip › Figure 2-source data 2/Figure 2E/plce1.tif]

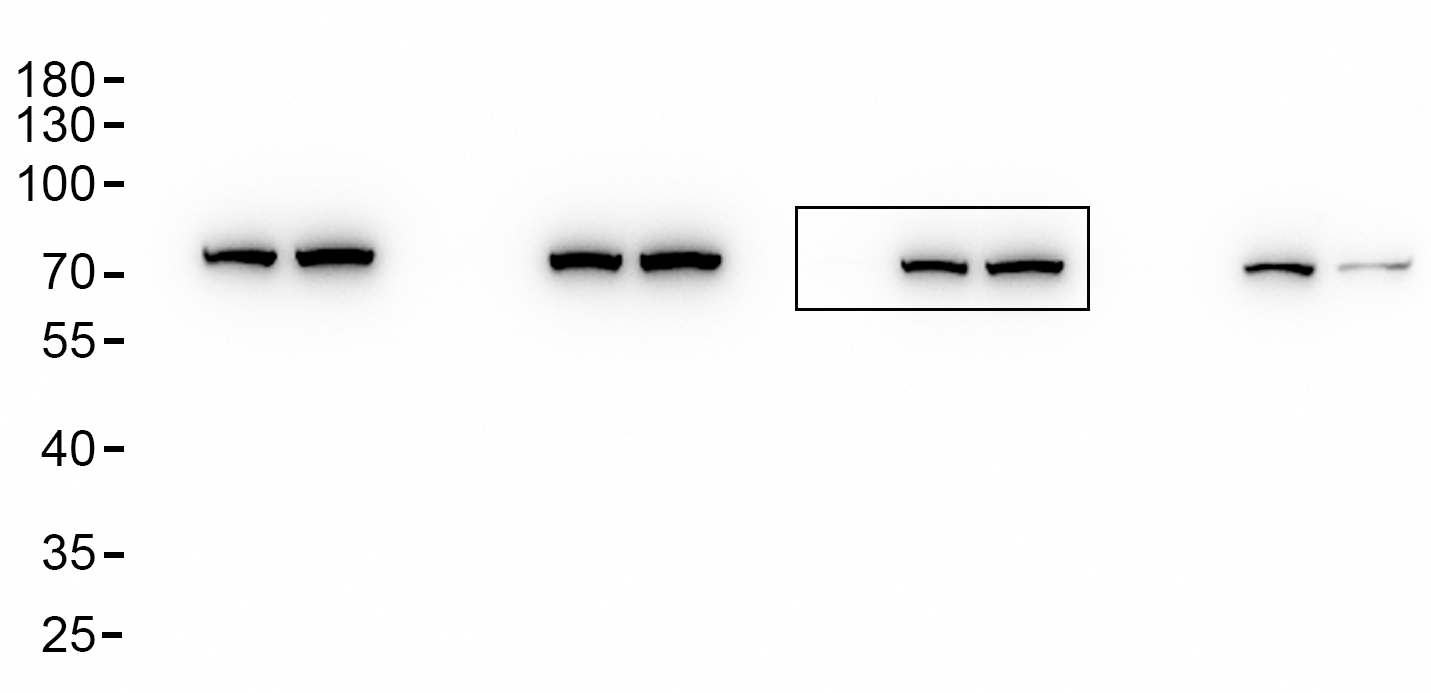

Supplement: Figure 2—source data 3. [file elife-97373-fig2-data3.zip › Figure 2-source data 2/Figure 2E/tak1.tif]

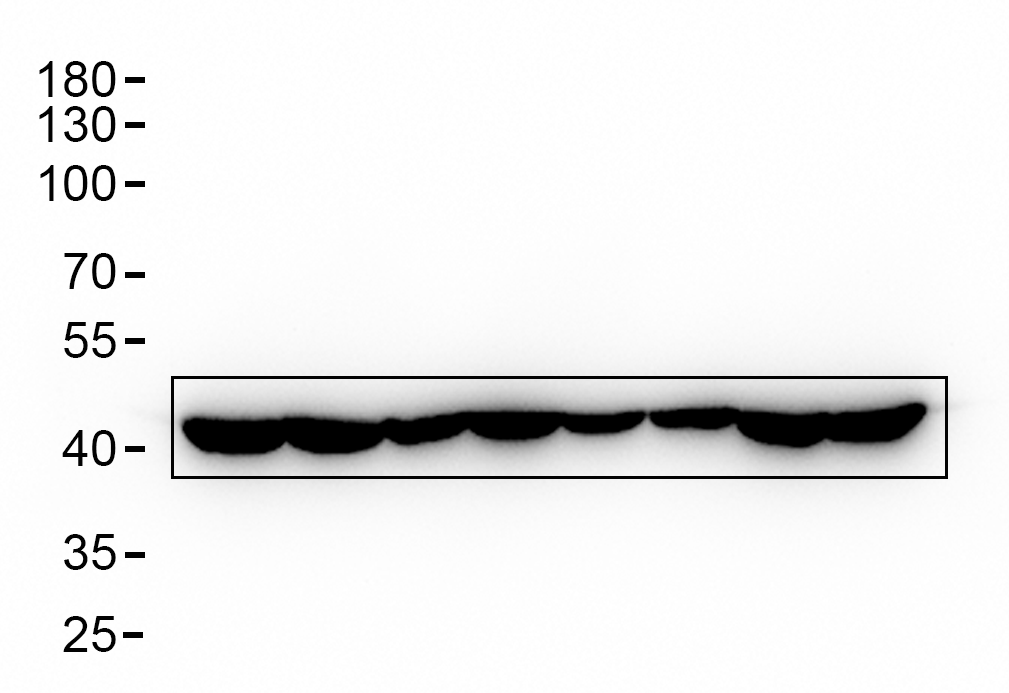

Supplement: Figure 2—source data 3. [file elife-97373-fig2-data3.zip › Figure 2-source data 2/Figure 2I/Actin.tif]

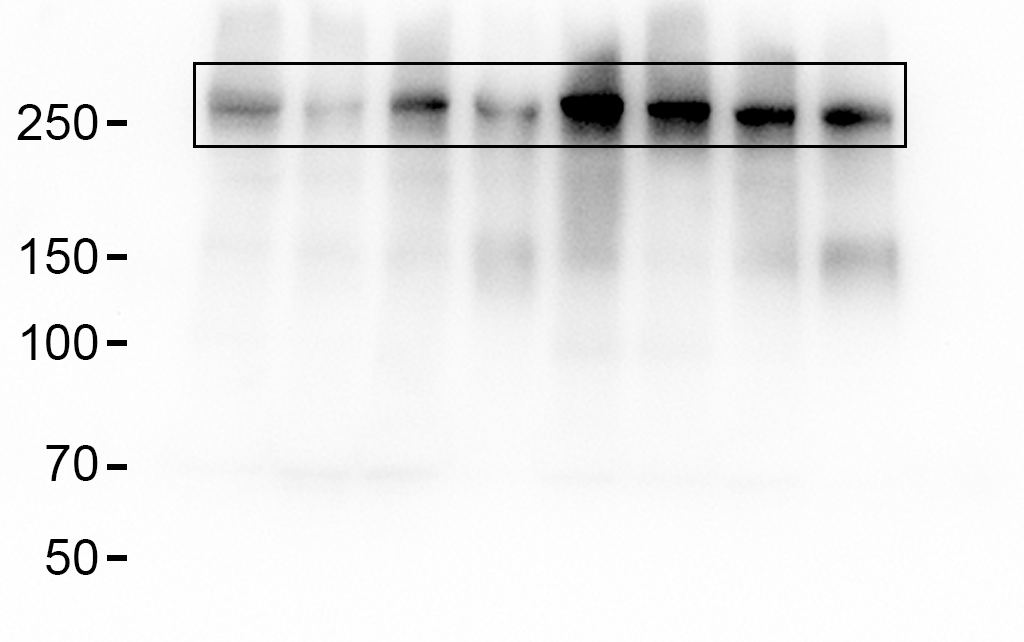

Supplement: Figure 2—source data 3. [file elife-97373-fig2-data3.zip › Figure 2-source data 2/Figure 2I/P-PLCE1.tif]

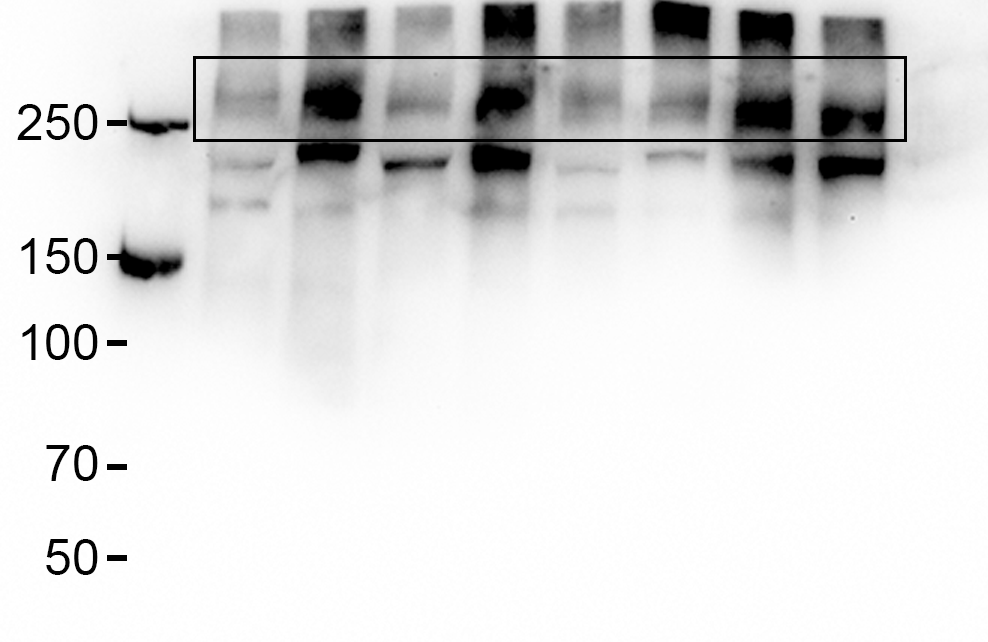

Supplement: Figure 2—source data 3. [file elife-97373-fig2-data3.zip › Figure 2-source data 2/Figure 2I/PLCE1.tif]

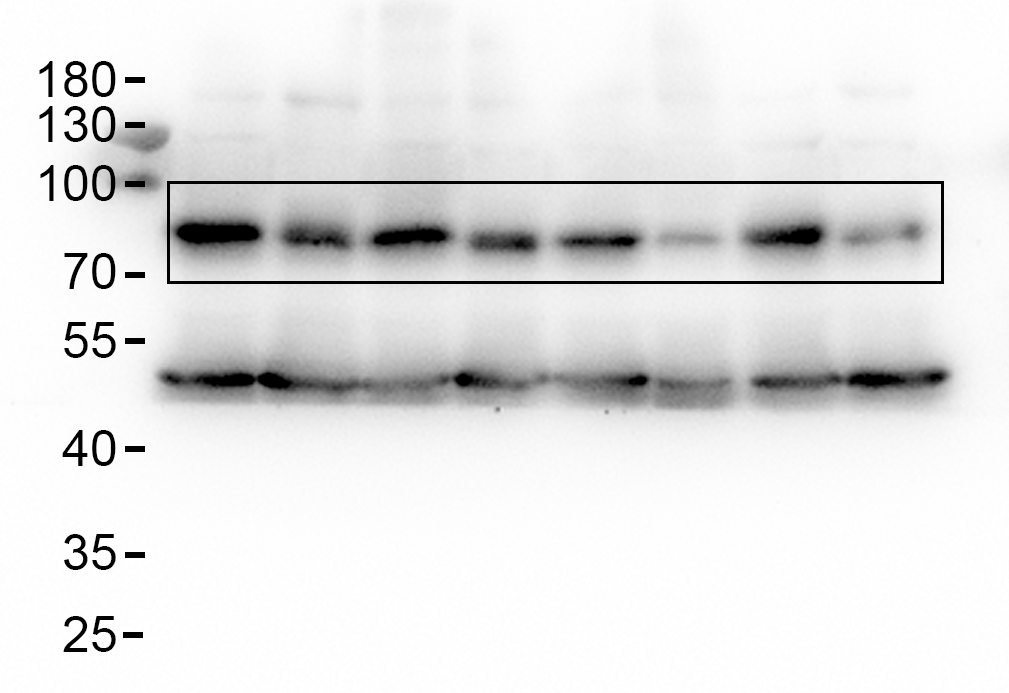

Supplement: Figure 2—source data 3. [file elife-97373-fig2-data3.zip › Figure 2-source data 2/Figure 2I/TAK1.tif]

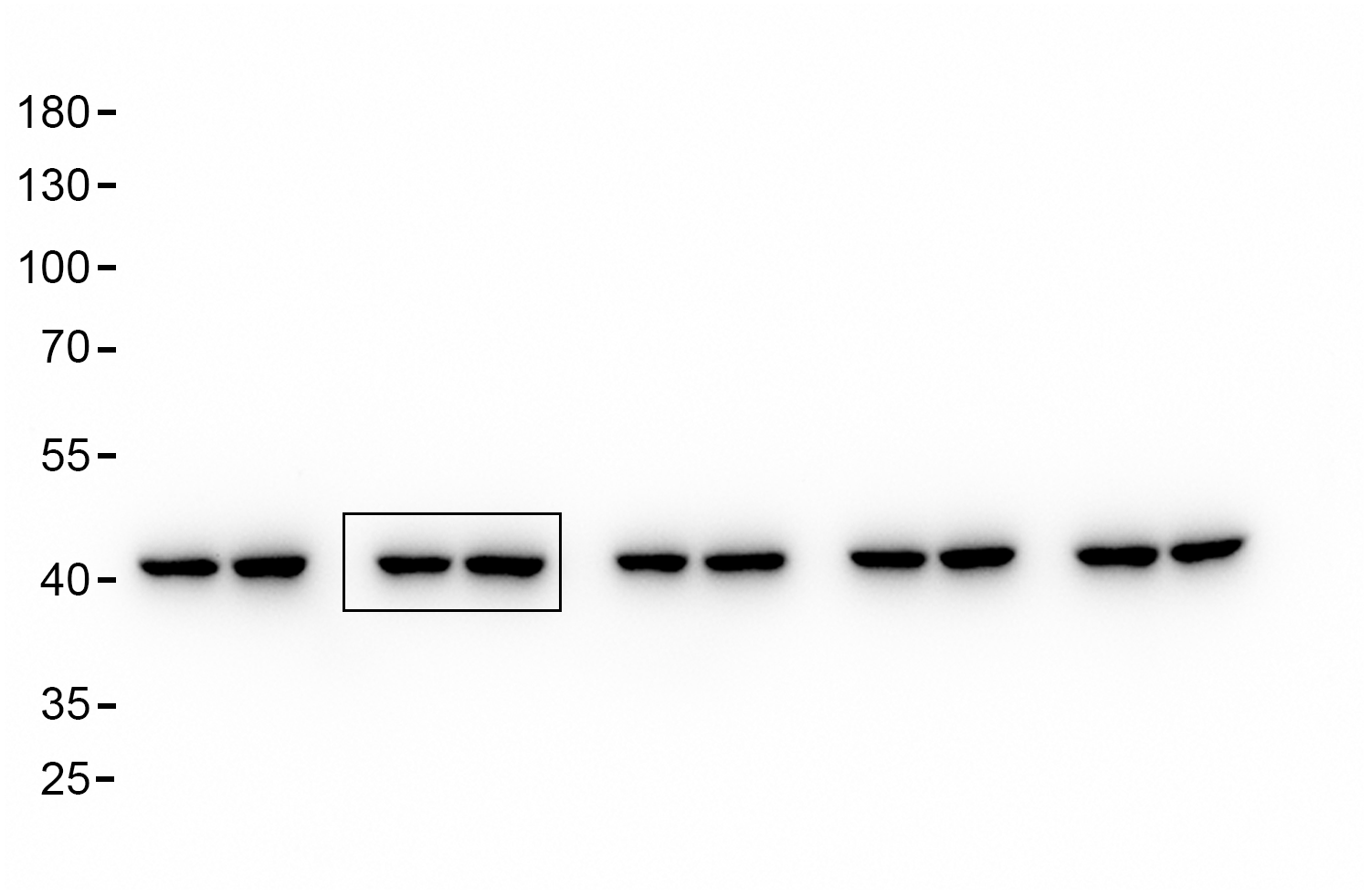

Supplement: Figure 2—figure supplement 1—source data 2. [file elife-97373-fig2-figsupp1-data2.zip › Figure 2-figure supplement 1-source data 2/Figure 2-figure supplement 1A/Actin.tif]

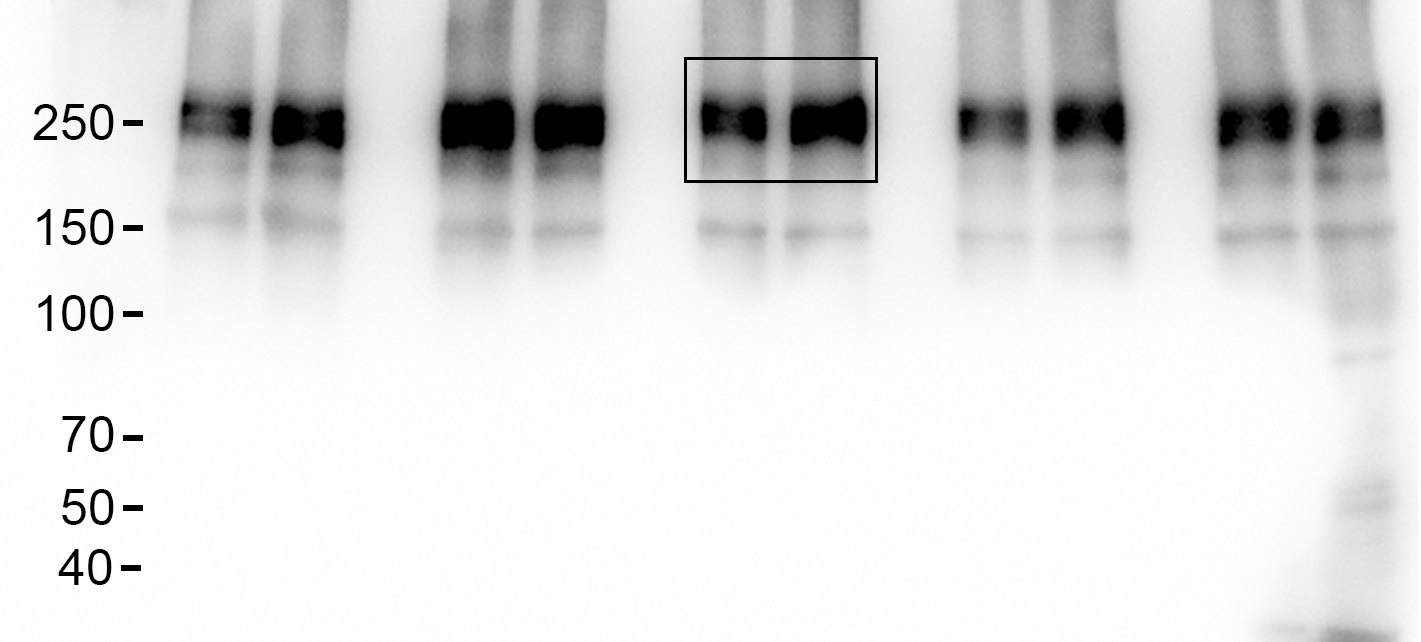

Supplement: Figure 2—figure supplement 1—source data 2. [file elife-97373-fig2-figsupp1-data2.zip › Figure 2-figure supplement 1-source data 2/Figure 2-figure supplement 1A/p-PLCE1.tif]

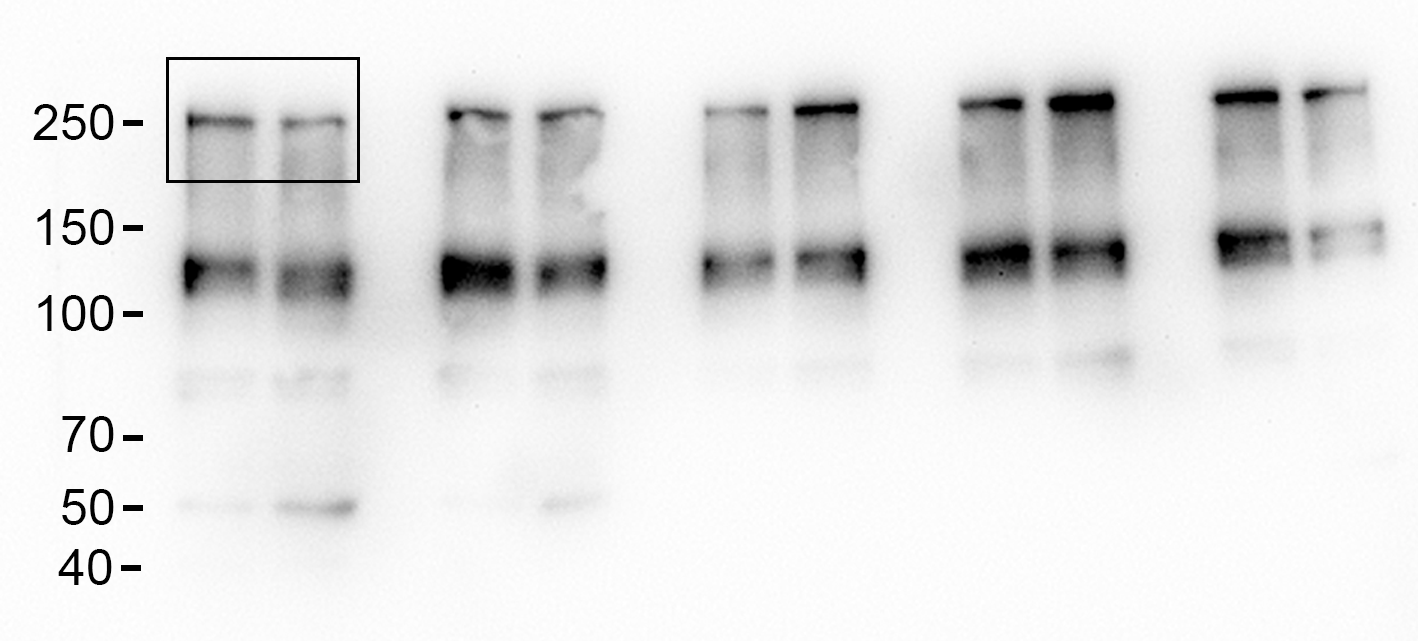

Supplement: Figure 2—figure supplement 1—source data 2. [file elife-97373-fig2-figsupp1-data2.zip › Figure 2-figure supplement 1-source data 2/Figure 2-figure supplement 1A/PLCE1.tif]

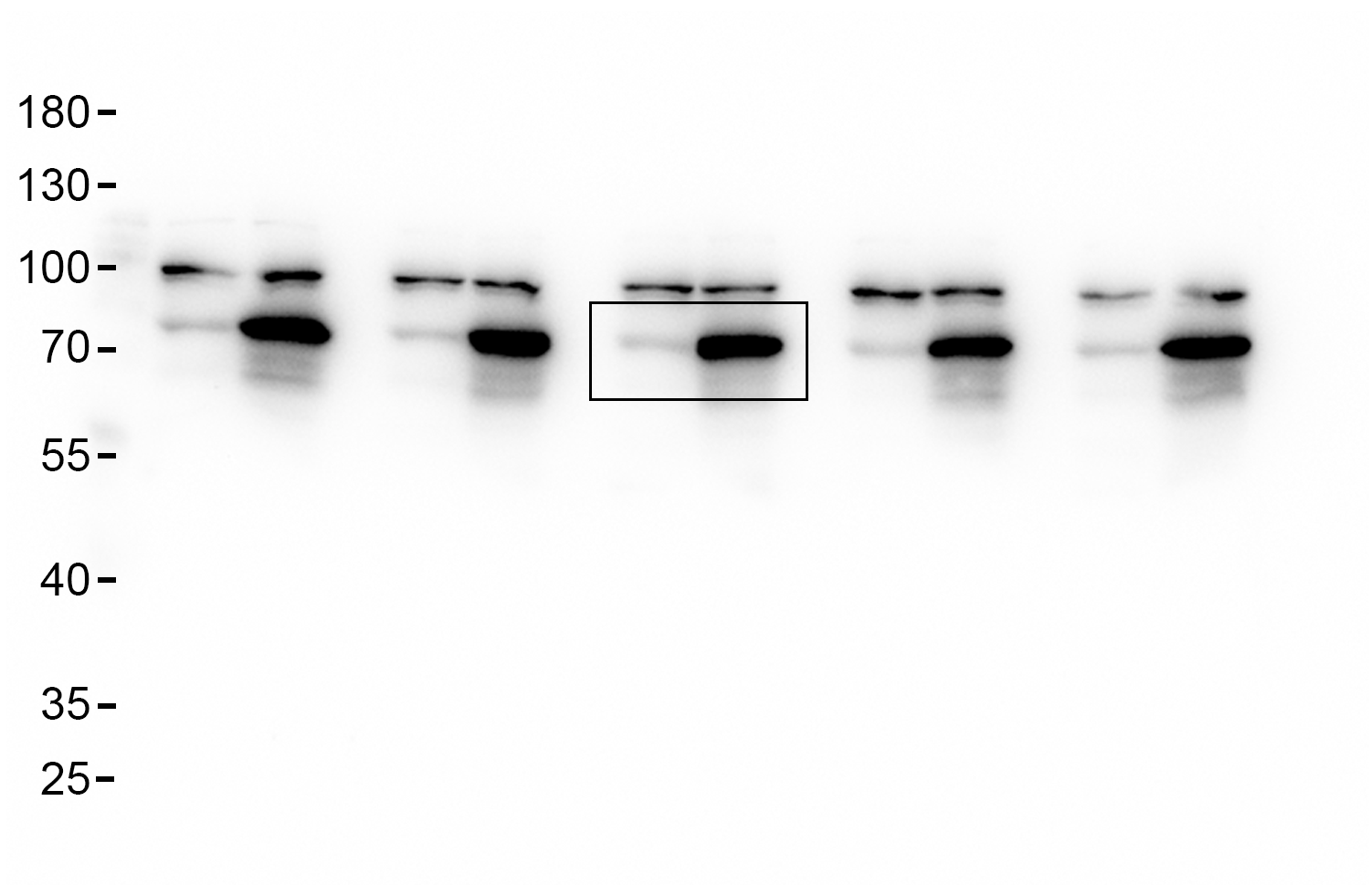

Supplement: Figure 2—figure supplement 1—source data 2. [file elife-97373-fig2-figsupp1-data2.zip › Figure 2-figure supplement 1-source data 2/Figure 2-figure supplement 1A/TAK1.tif]

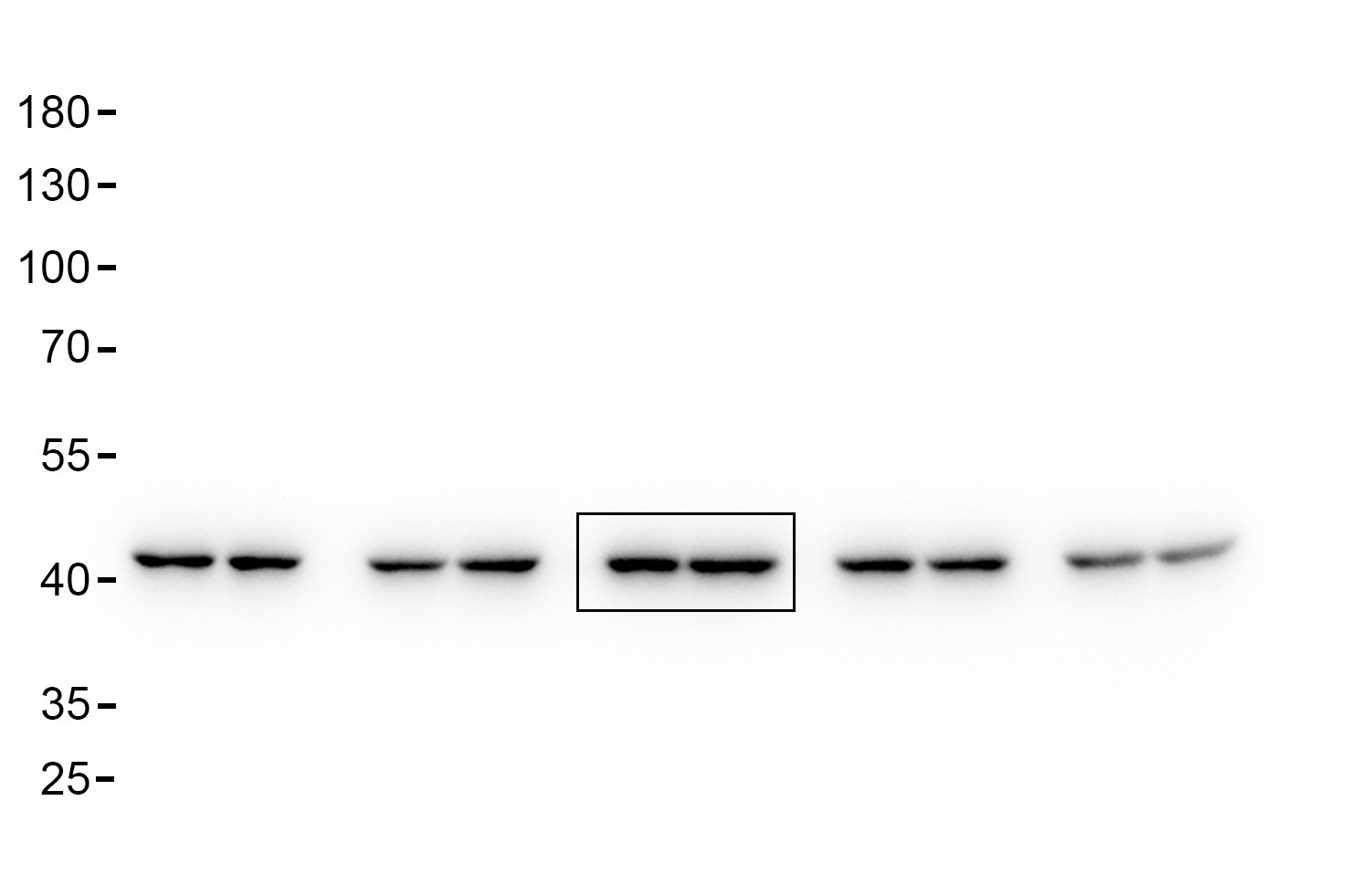

Supplement: Figure 2—figure supplement 1—source data 2. [file elife-97373-fig2-figsupp1-data2.zip › Figure 2-figure supplement 1-source data 2/Figure 2-figure supplement 1B/Actin.tif]

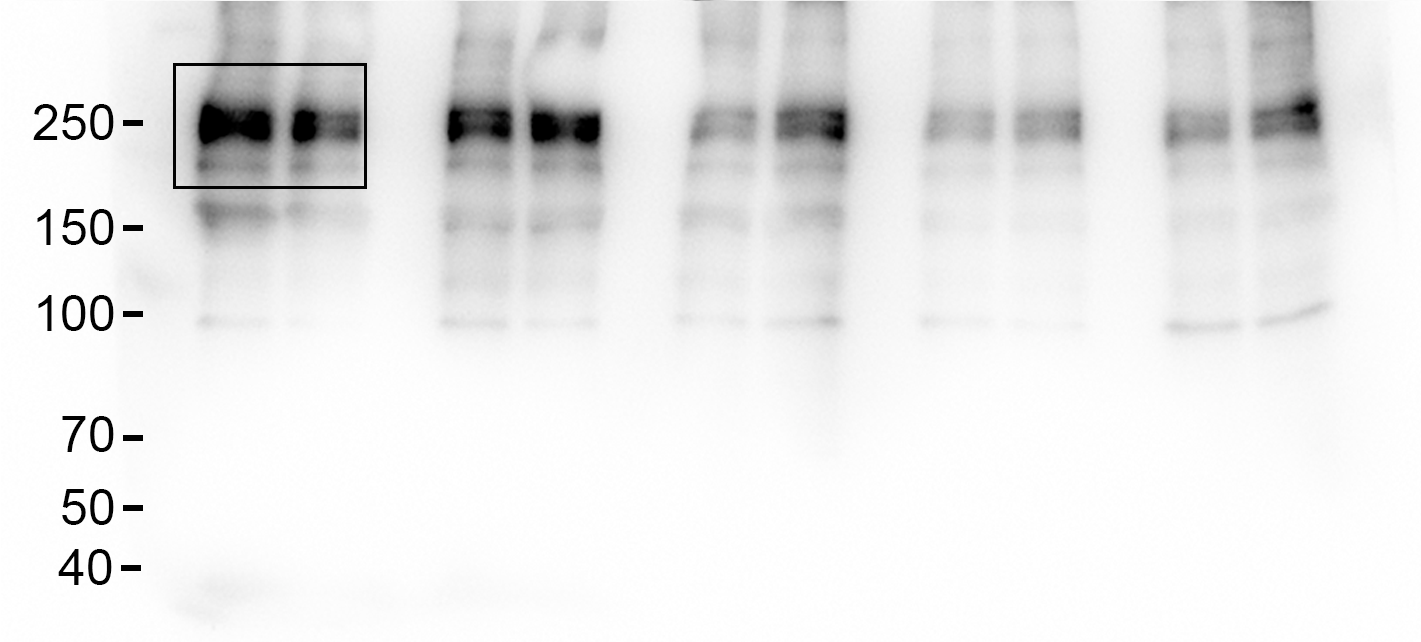

Supplement: Figure 2—figure supplement 1—source data 2. [file elife-97373-fig2-figsupp1-data2.zip › Figure 2-figure supplement 1-source data 2/Figure 2-figure supplement 1B/p-PLCE1.tif]

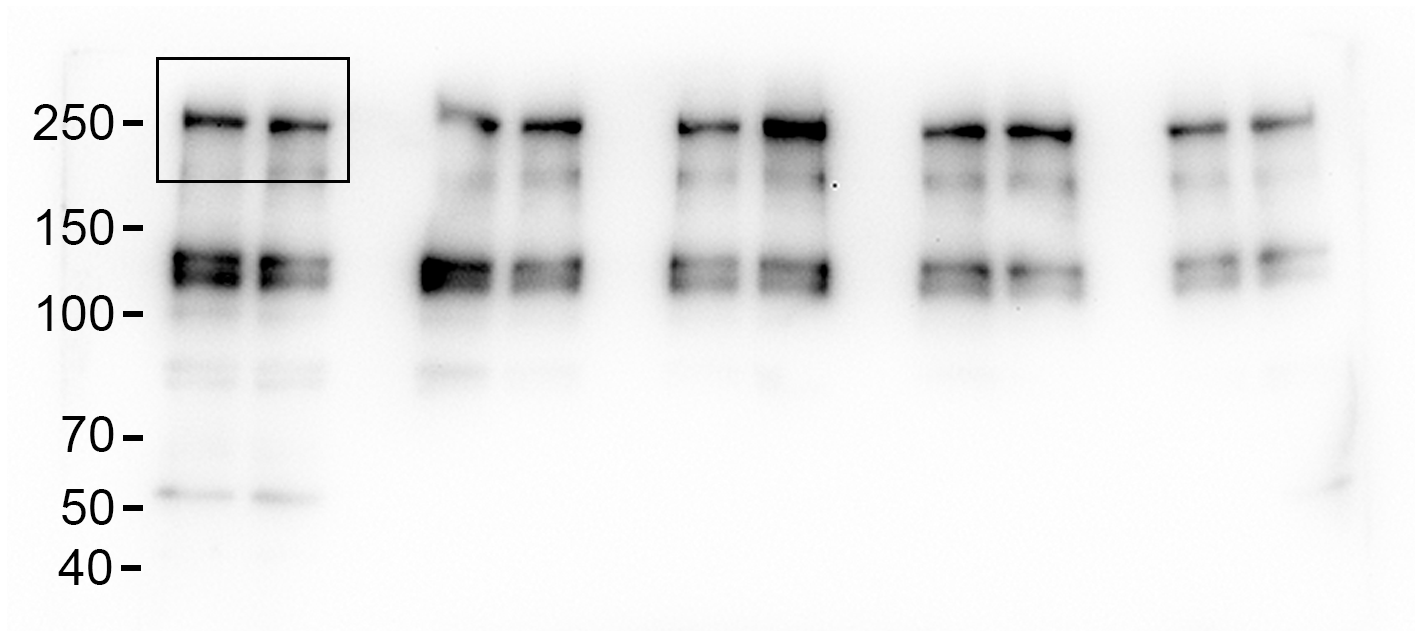

Supplement: Figure 2—figure supplement 1—source data 2. [file elife-97373-fig2-figsupp1-data2.zip › Figure 2-figure supplement 1-source data 2/Figure 2-figure supplement 1B/PLCE1.tif]

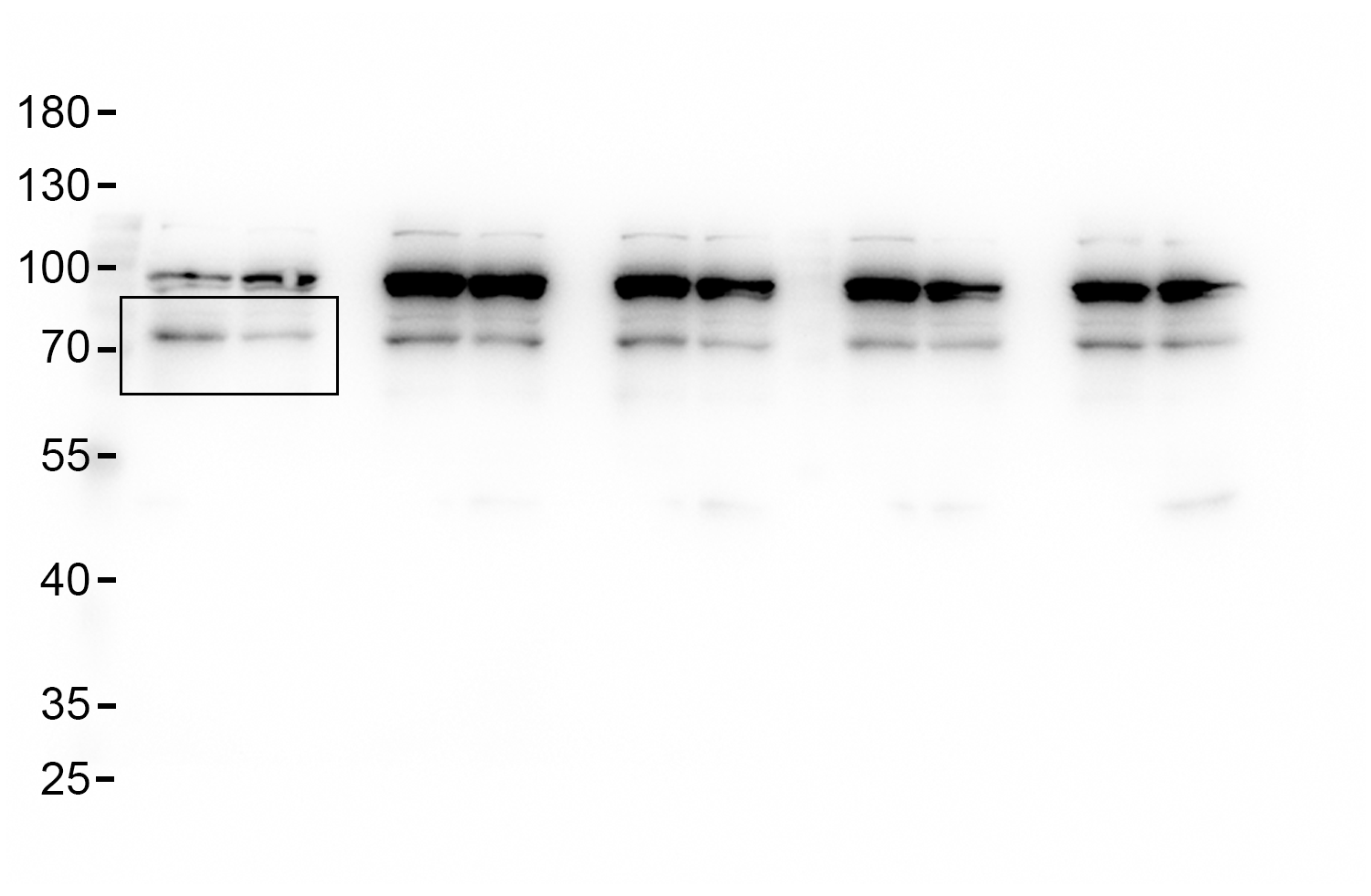

Supplement: Figure 2—figure supplement 1—source data 2. [file elife-97373-fig2-figsupp1-data2.zip › Figure 2-figure supplement 1-source data 2/Figure 2-figure supplement 1B/TAK1.tif]

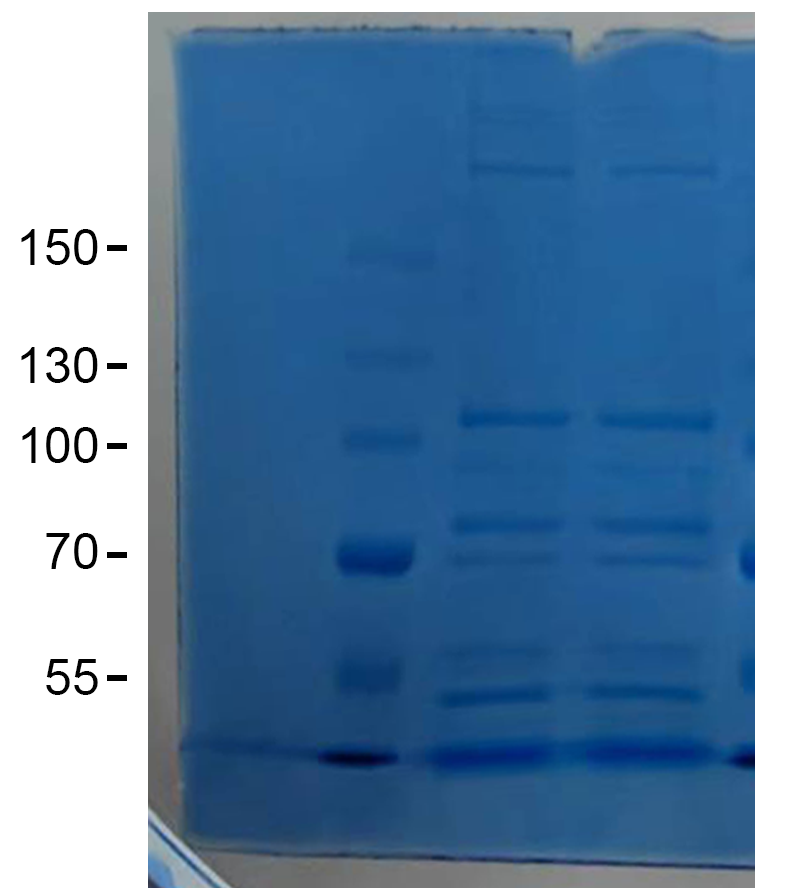

Supplement: Figure 2—figure supplement 1—source data 2. [file elife-97373-fig2-figsupp1-data2.zip › Figure 2-figure supplement 1-source data 2/Figure 2-figure supplement 1C/Coomassie.tif]

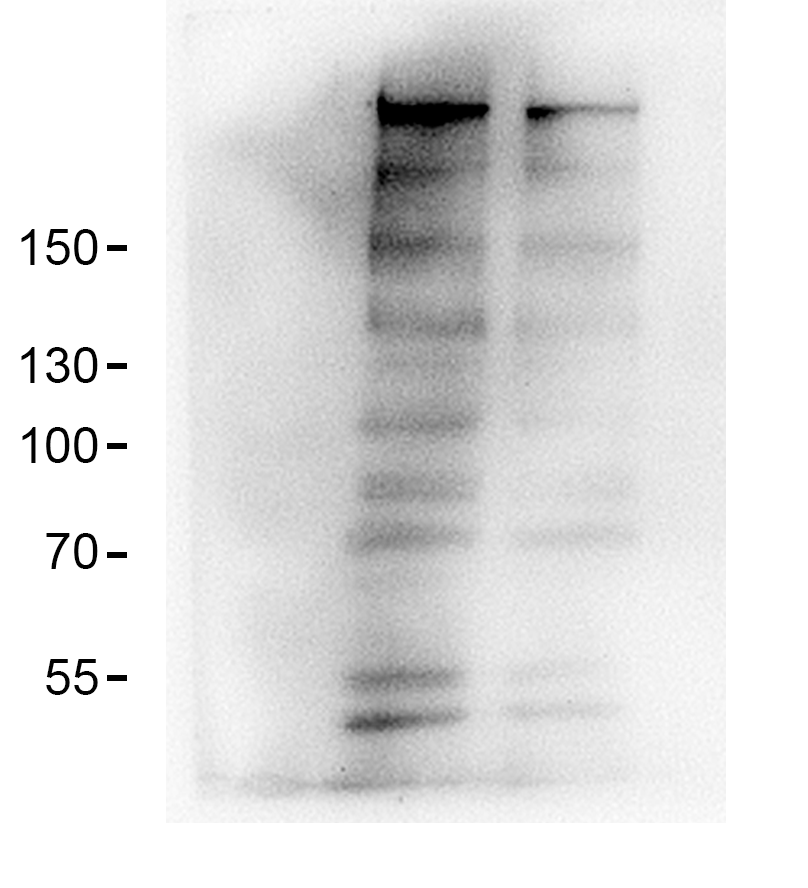

Supplement: Figure 2—figure supplement 1—source data 2. [file elife-97373-fig2-figsupp1-data2.zip › Figure 2-figure supplement 1-source data 2/Figure 2-figure supplement 1C/p-PLCE.tif]

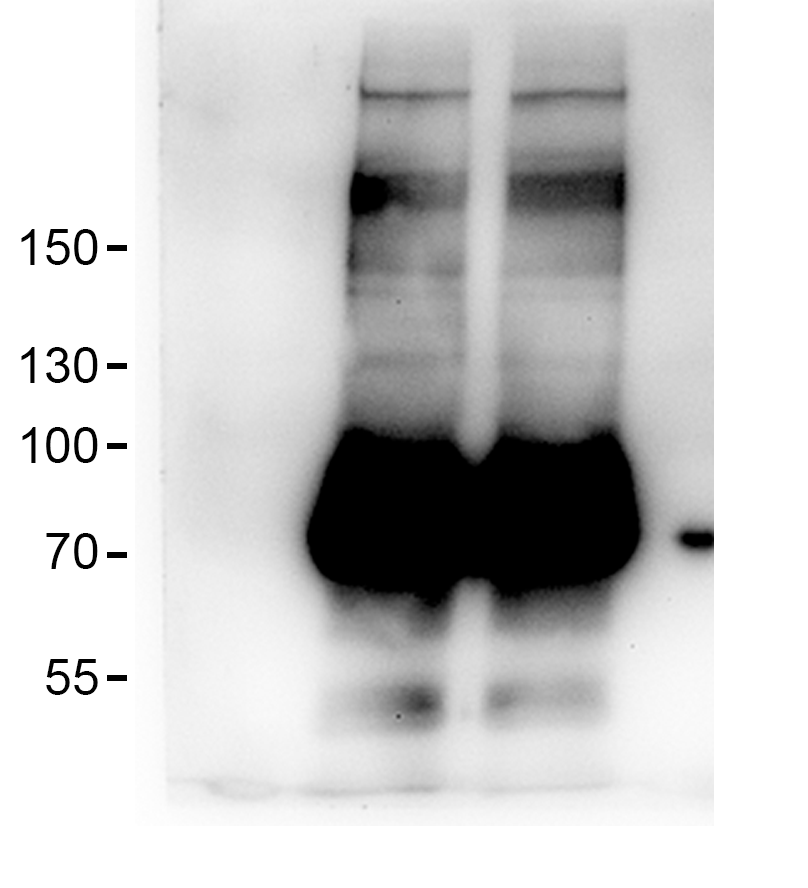

Supplement: Figure 2—figure supplement 1—source data 2. [file elife-97373-fig2-figsupp1-data2.zip › Figure 2-figure supplement 1-source data 2/Figure 2-figure supplement 1C/PLCE1+TAK1.tif]

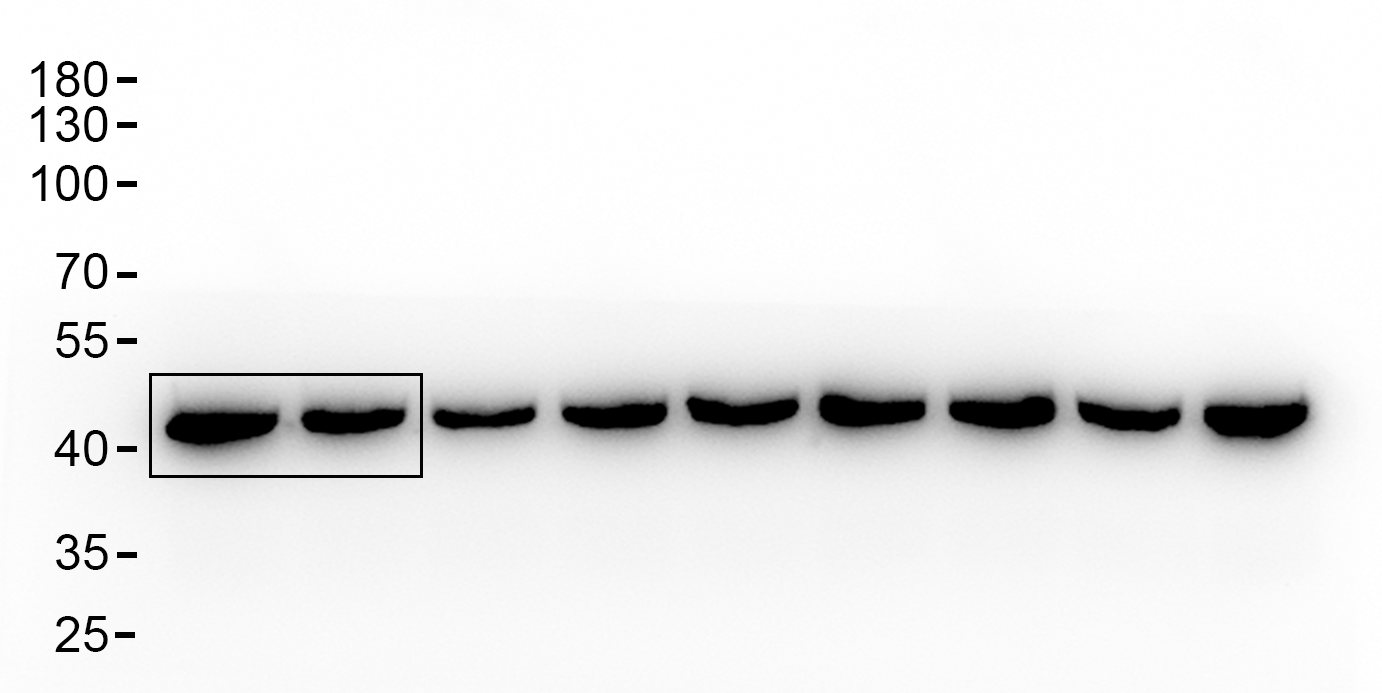

Supplement: Figure 3—source data 3. [file elife-97373-fig3-data3.zip › Figure 3-source data 2/Figure 3A/Actin.tif]

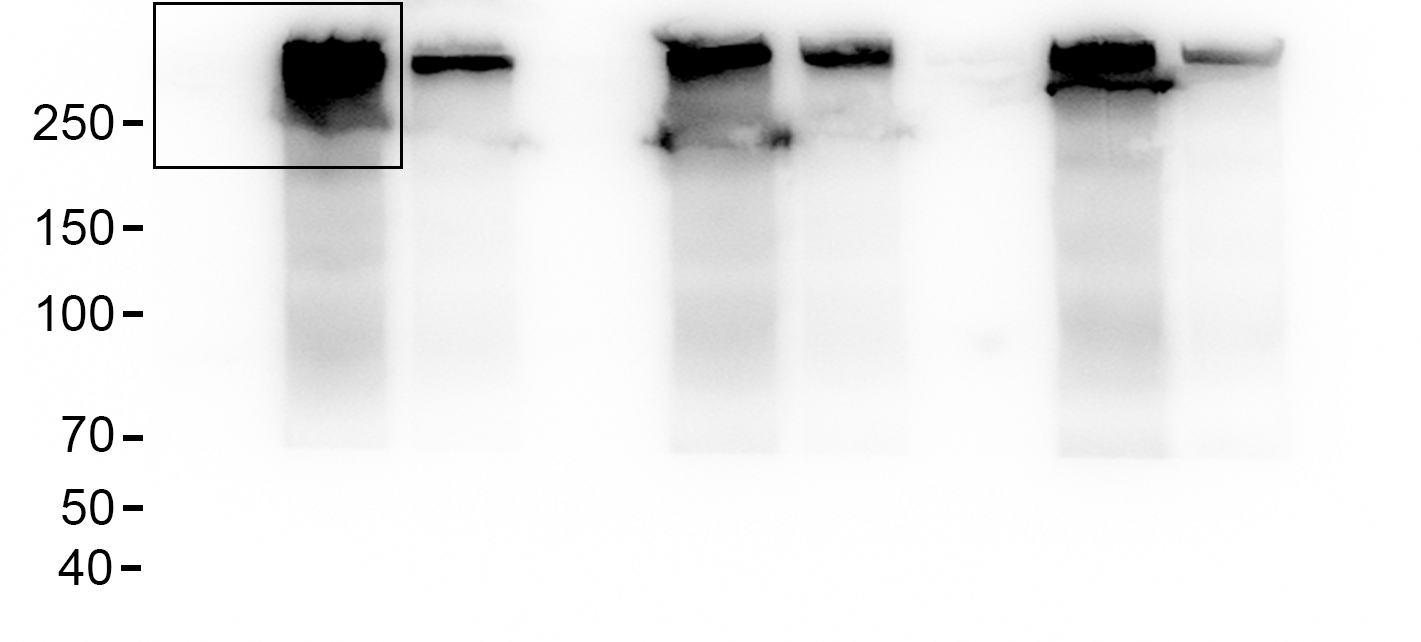

Supplement: Figure 3—source data 3. [file elife-97373-fig3-data3.zip › Figure 3-source data 2/Figure 3A/plce1.tif]

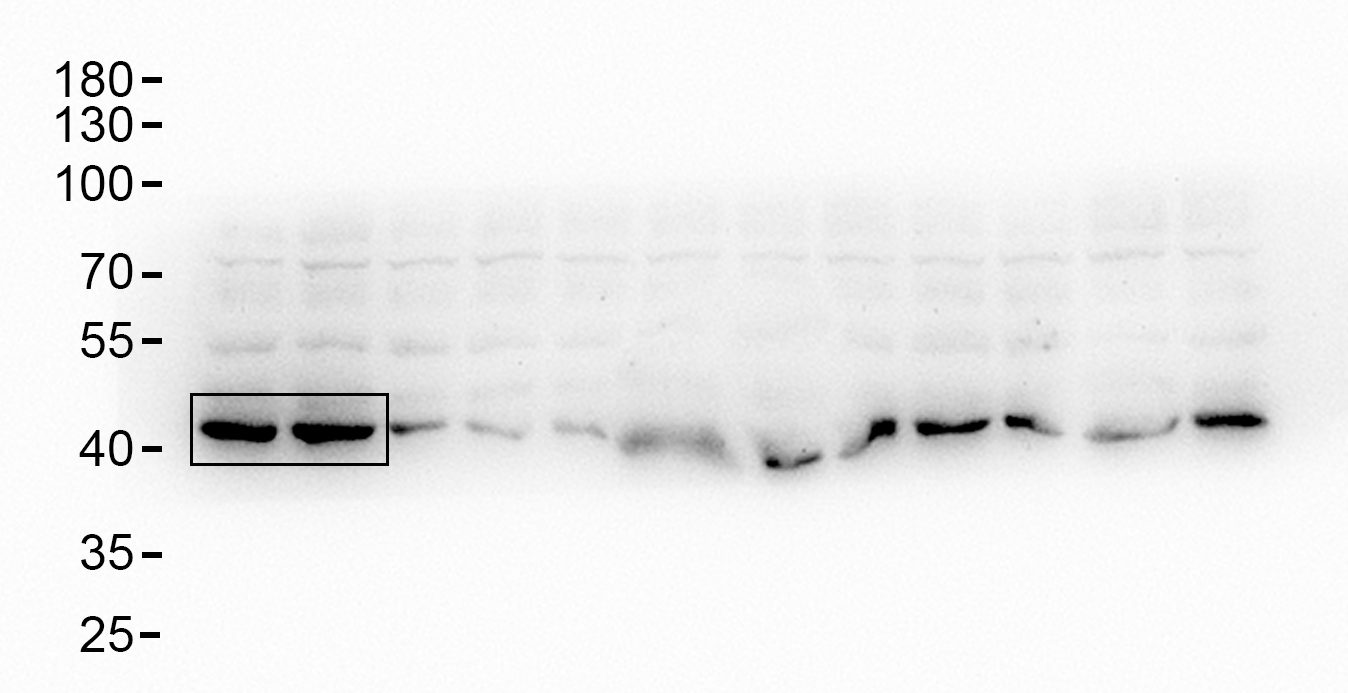

Supplement: Figure 3—source data 3. [file elife-97373-fig3-data3.zip › Figure 3-source data 2/Figure 3D/Actin.tif]

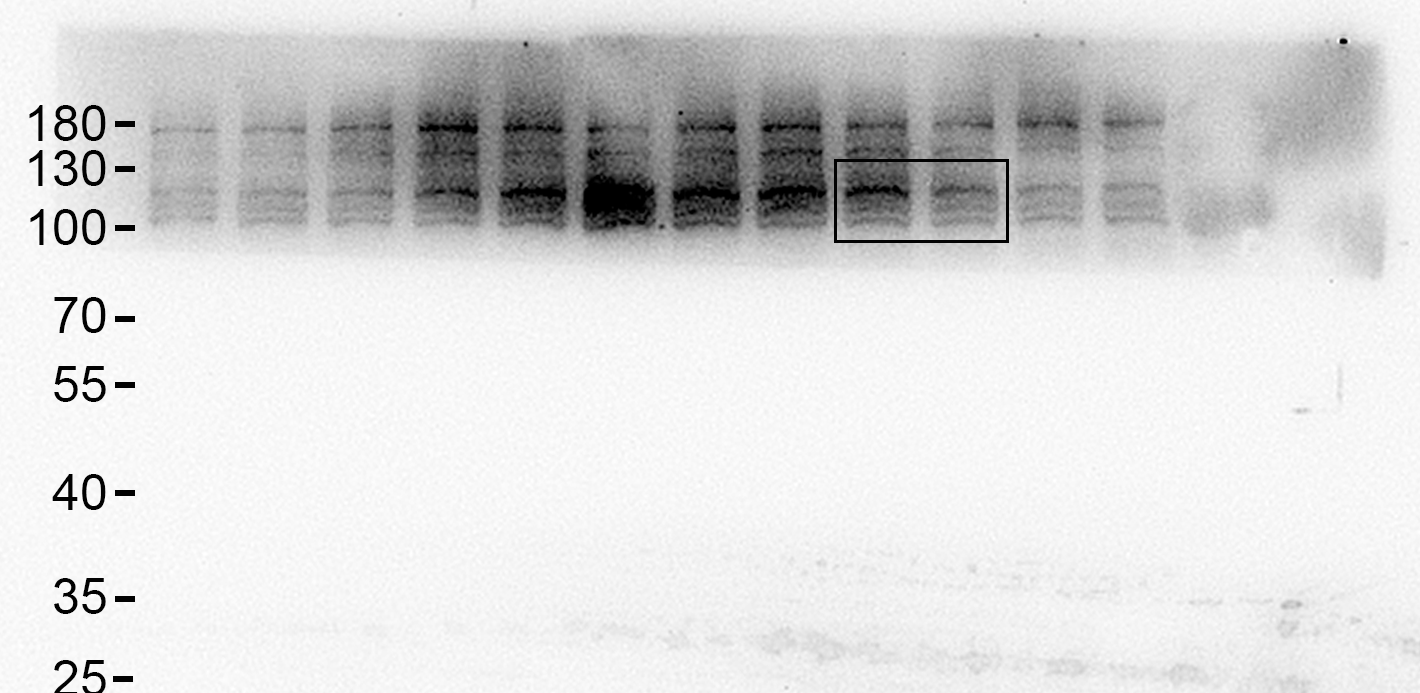

Supplement: Figure 3—source data 3. [file elife-97373-fig3-data3.zip › Figure 3-source data 2/Figure 3D/E-cadherin.tif]

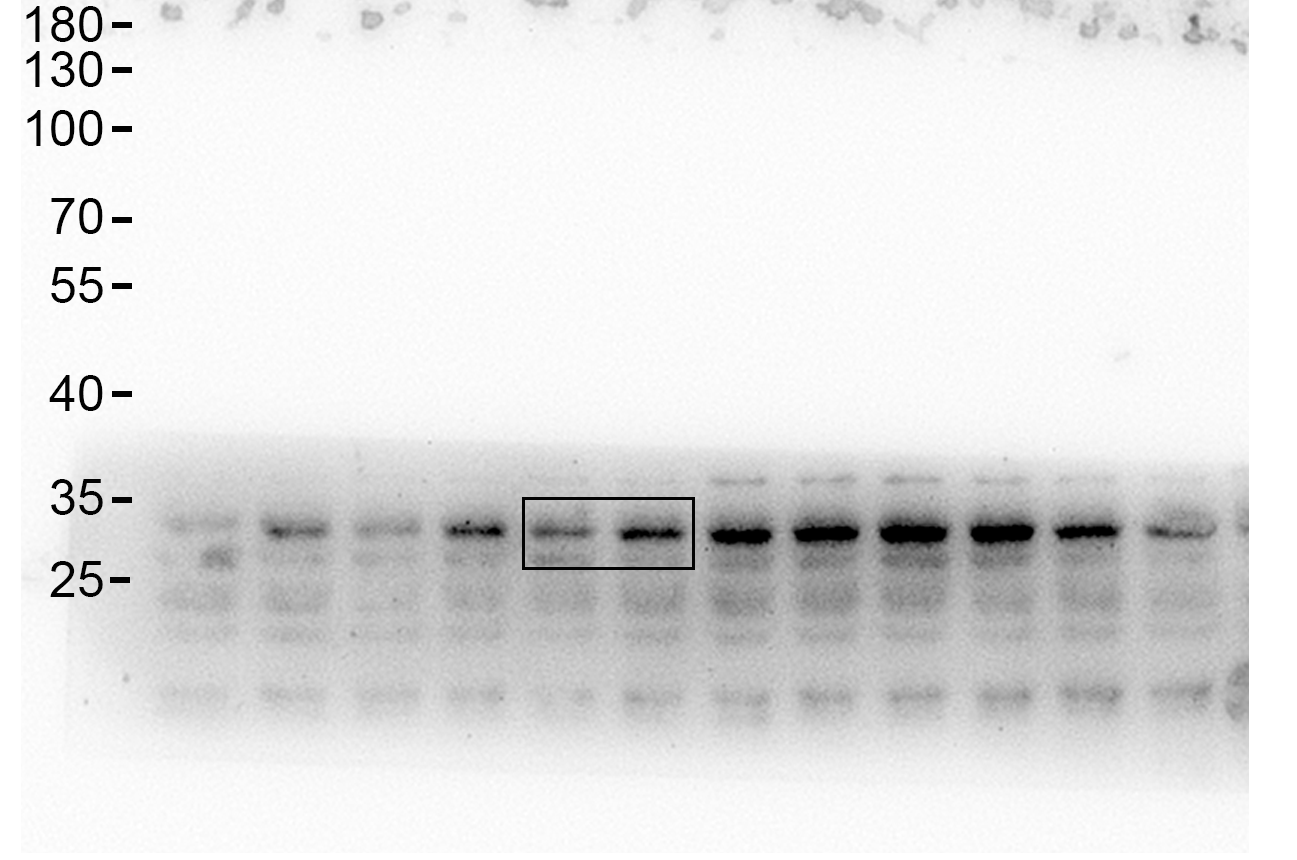

Supplement: Figure 3—source data 3. [file elife-97373-fig3-data3.zip › Figure 3-source data 2/Figure 3D/slug.tif]

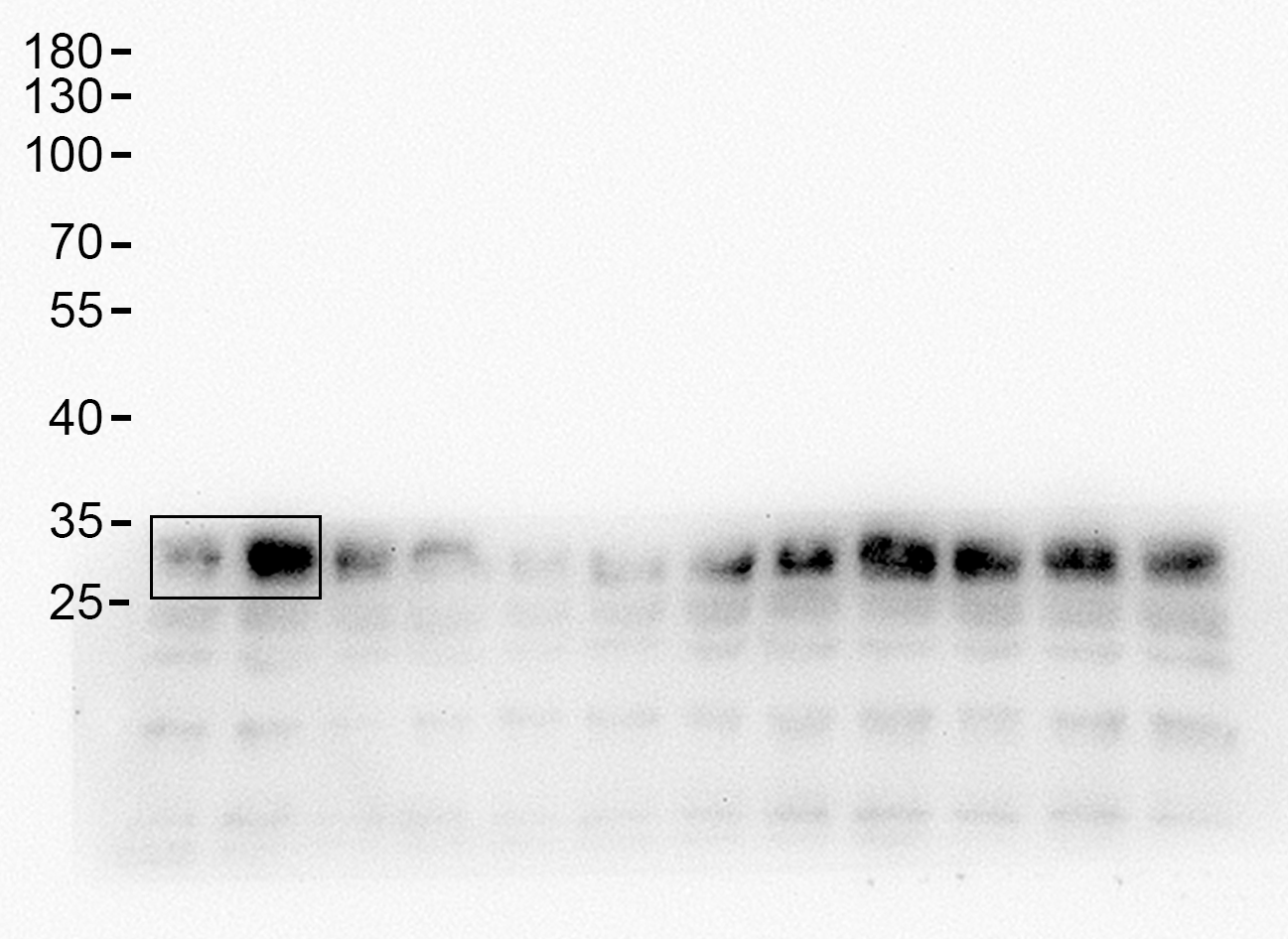

Supplement: Figure 3—source data 3. [file elife-97373-fig3-data3.zip › Figure 3-source data 2/Figure 3D/snail.tif]

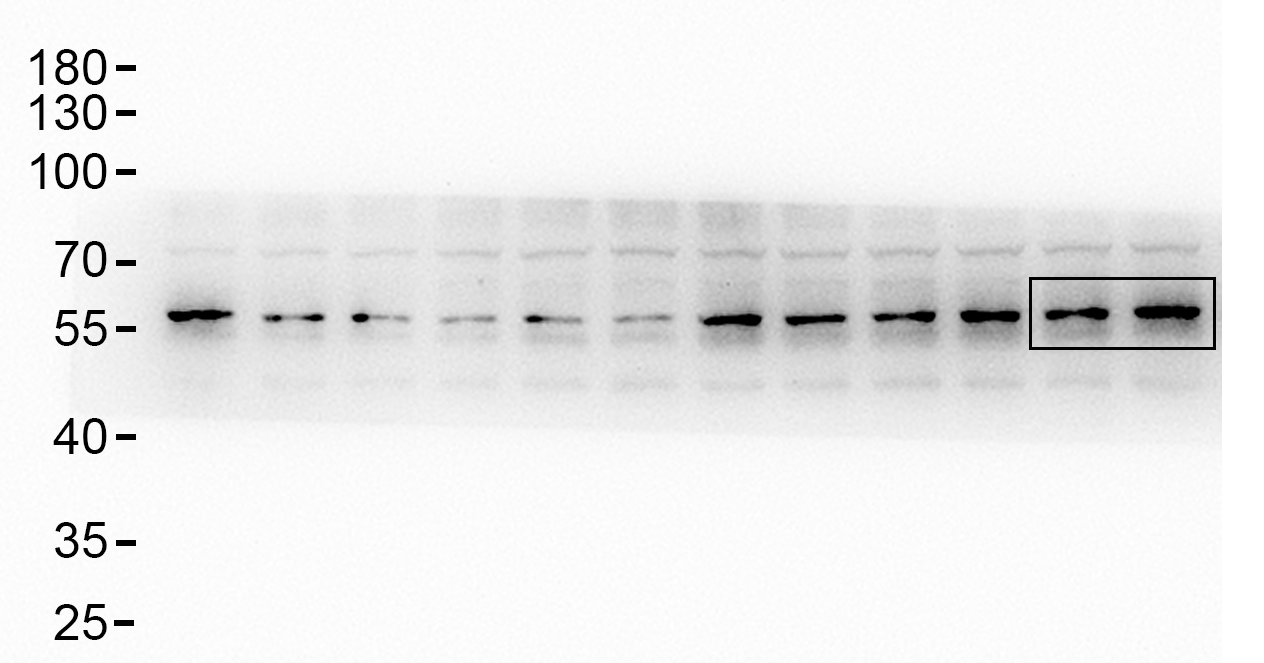

Supplement: Figure 3—source data 3. [file elife-97373-fig3-data3.zip › Figure 3-source data 2/Figure 3D/vimentin.tif]

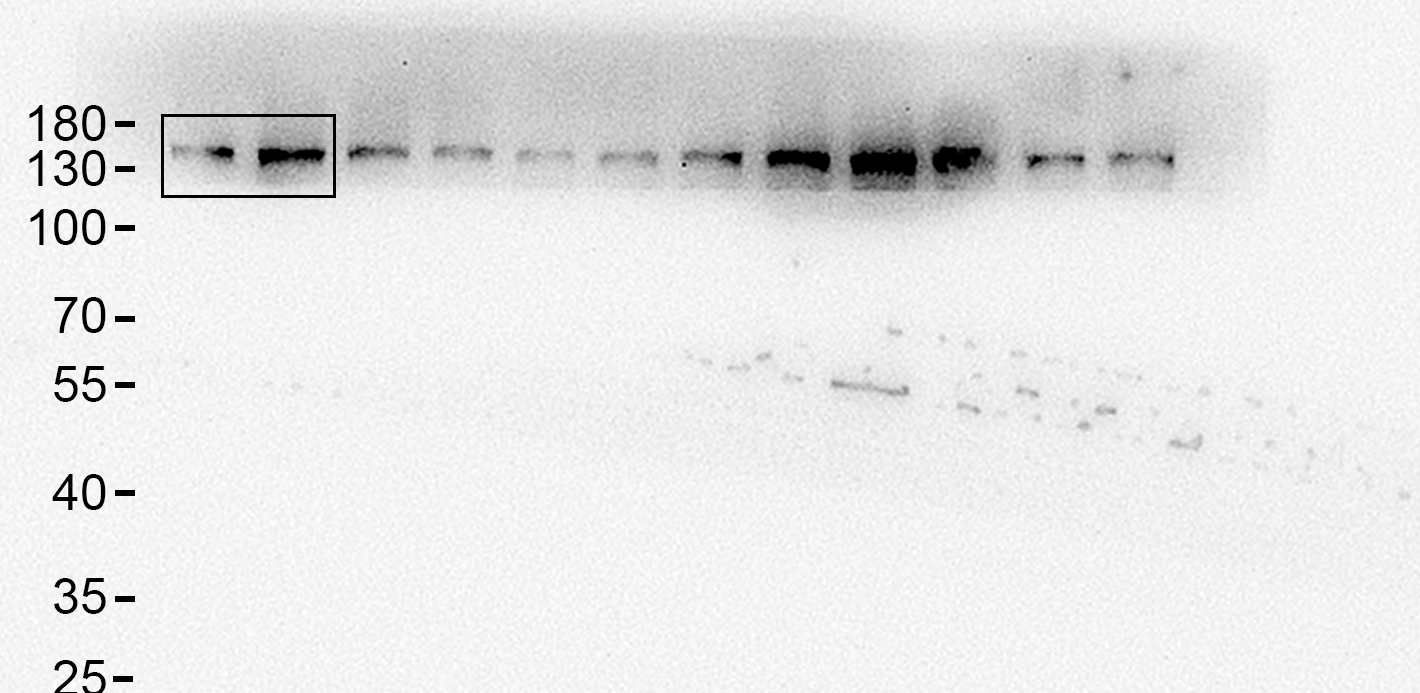

Supplement: Figure 3—source data 3. [file elife-97373-fig3-data3.zip › Figure 3-source data 2/Figure 3D/ZEB.tif]

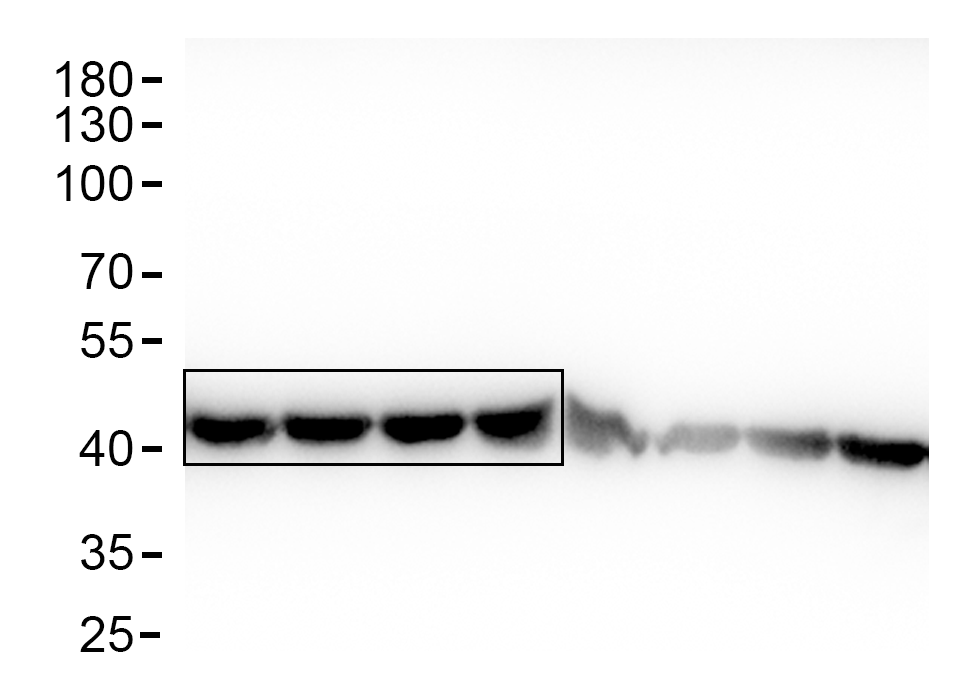

Supplement: Figure 3—source data 3. [file elife-97373-fig3-data3.zip › Figure 3-source data 2/Figure 3F/Actin.tif]

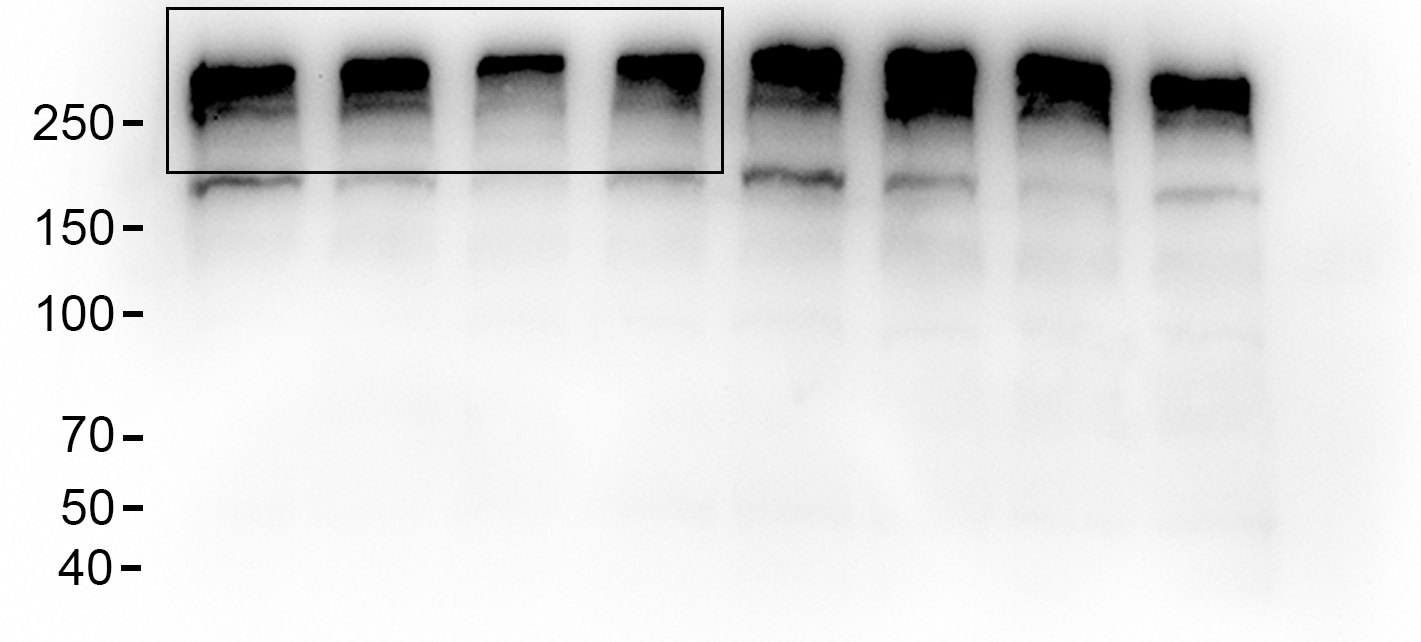

Supplement: Figure 3—source data 3. [file elife-97373-fig3-data3.zip › Figure 3-source data 2/Figure 3F/plce1.tif]

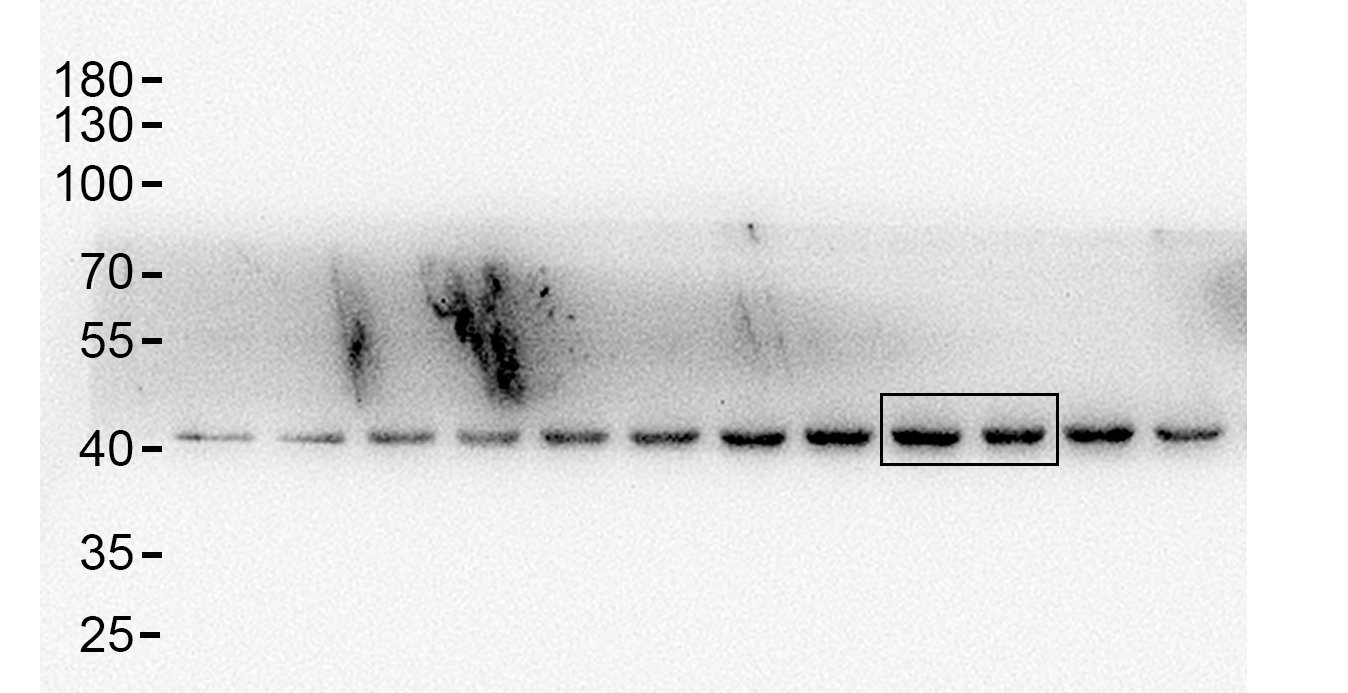

Supplement: Figure 3—source data 3. [file elife-97373-fig3-data3.zip › Figure 3-source data 2/Figure 3I/Actin.tif]

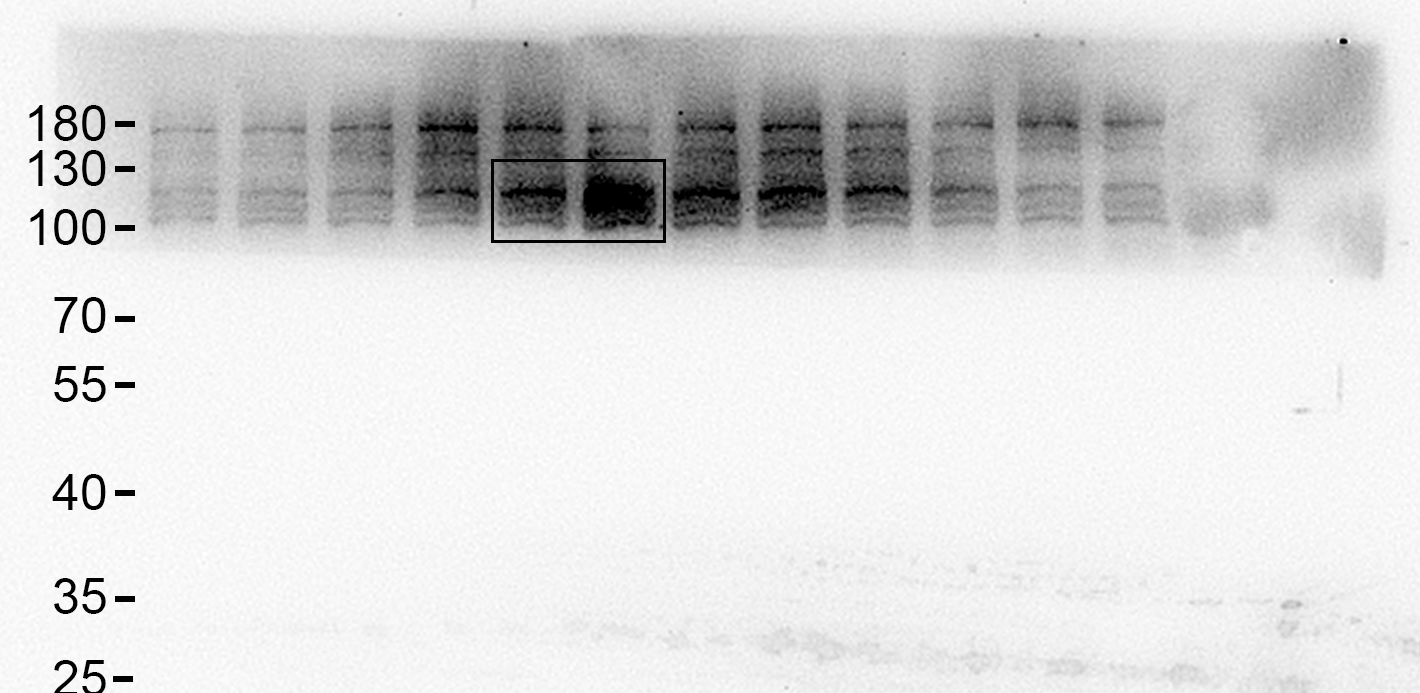

Supplement: Figure 3—source data 3. [file elife-97373-fig3-data3.zip › Figure 3-source data 2/Figure 3I/E-cadherin.tif]

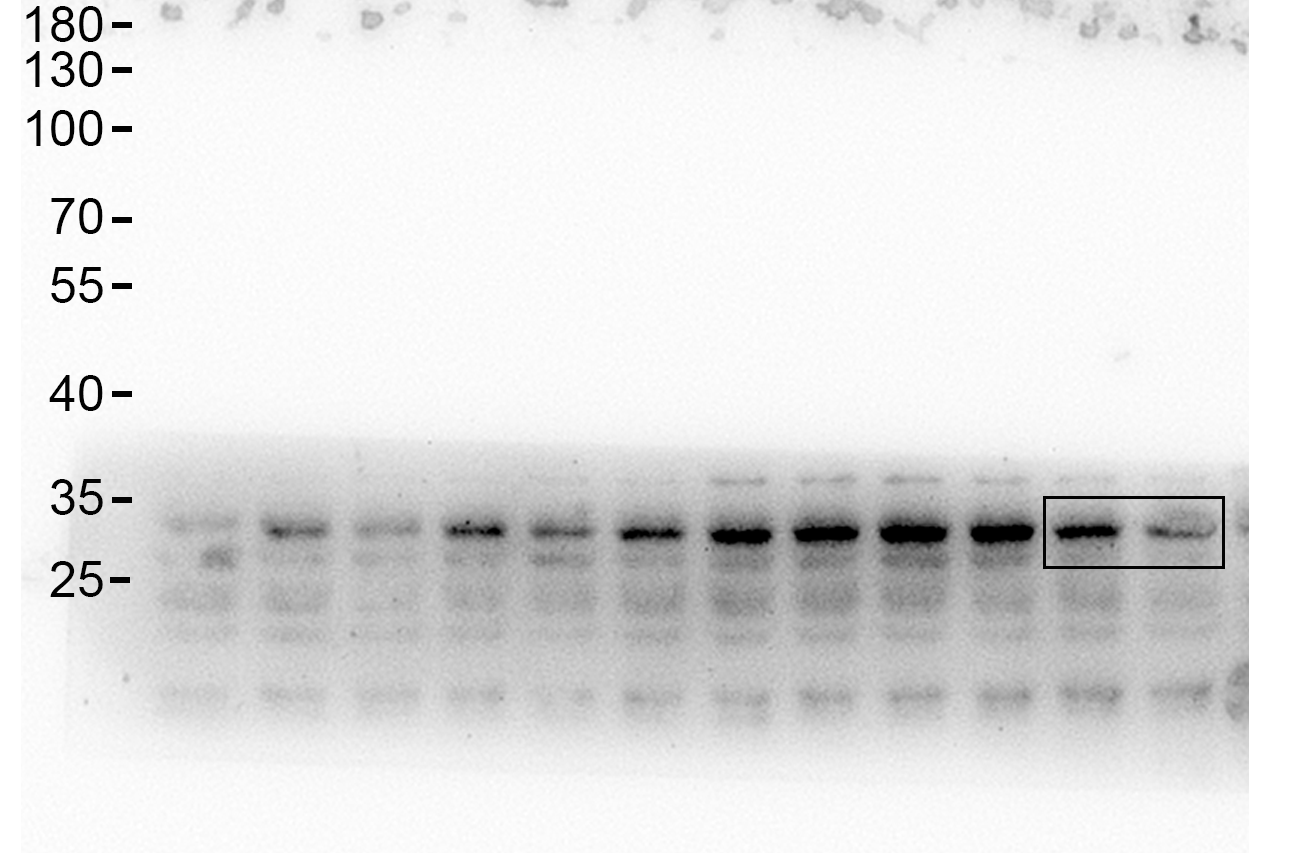

Supplement: Figure 3—source data 3. [file elife-97373-fig3-data3.zip › Figure 3-source data 2/Figure 3I/slug.tif]

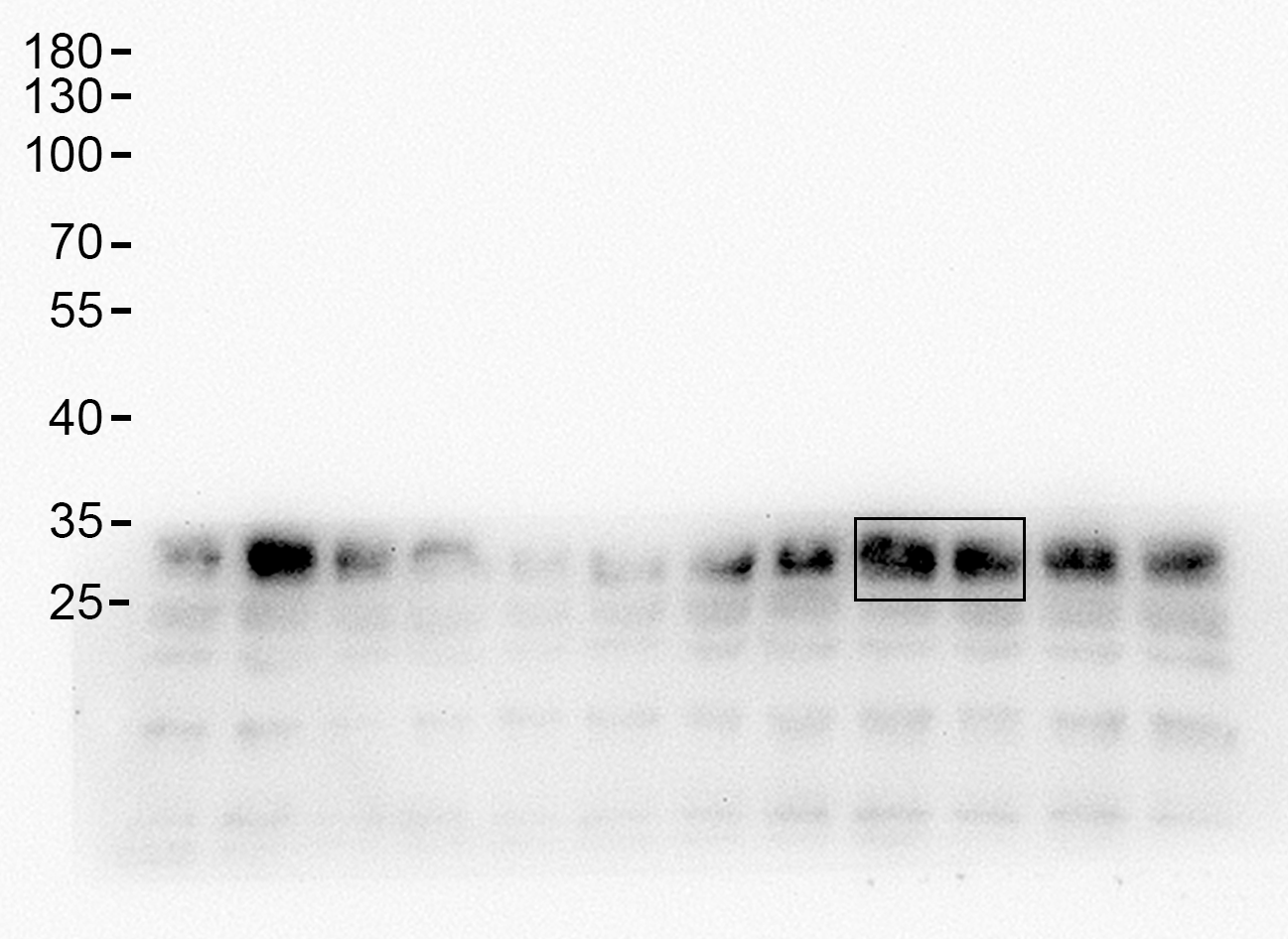

Supplement: Figure 3—source data 3. [file elife-97373-fig3-data3.zip › Figure 3-source data 2/Figure 3I/snail.tif]

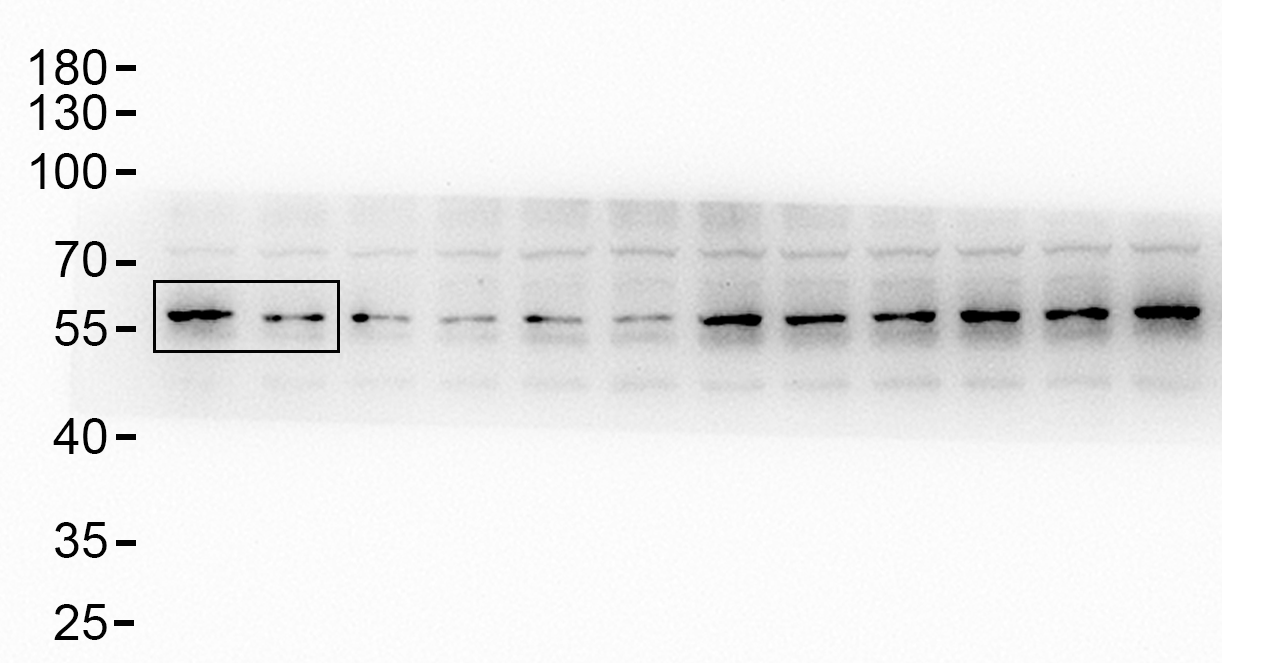

Supplement: Figure 3—source data 3. [file elife-97373-fig3-data3.zip › Figure 3-source data 2/Figure 3I/vimentin.tif]

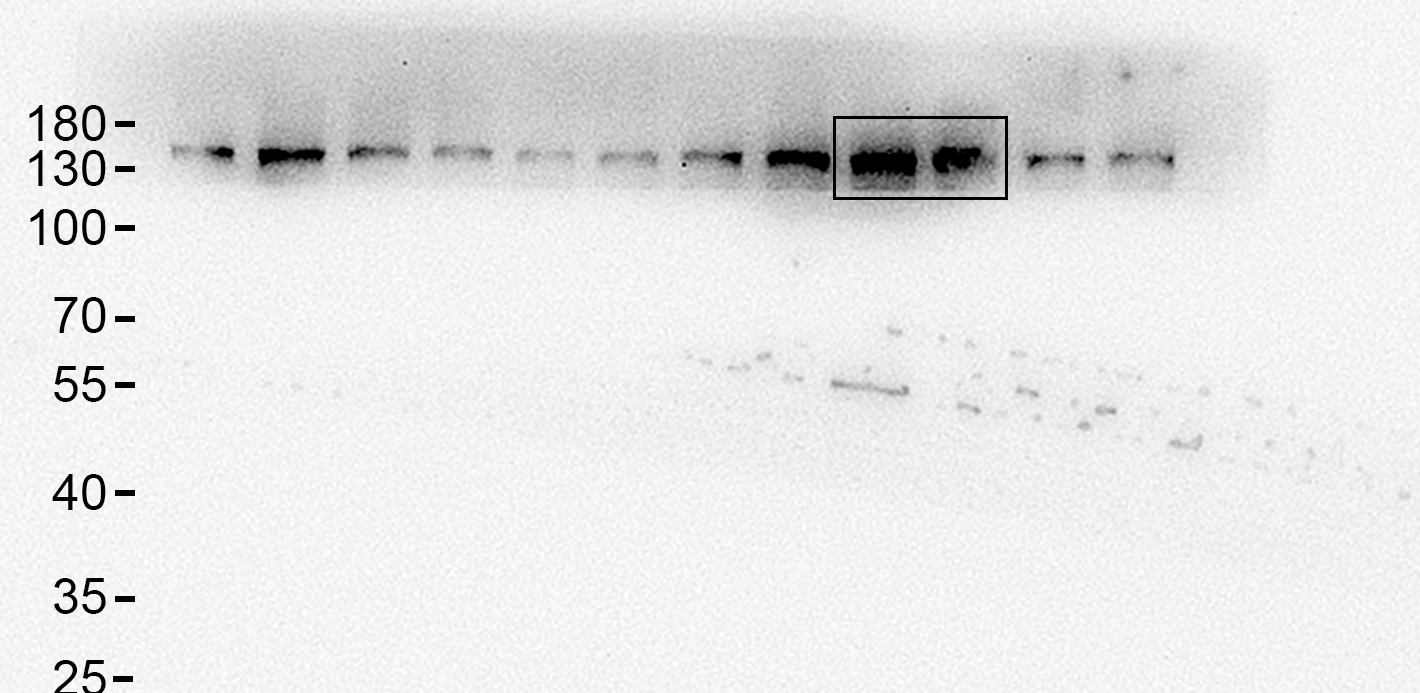

Supplement: Figure 3—source data 3. [file elife-97373-fig3-data3.zip › Figure 3-source data 2/Figure 3I/ZEB.tif]

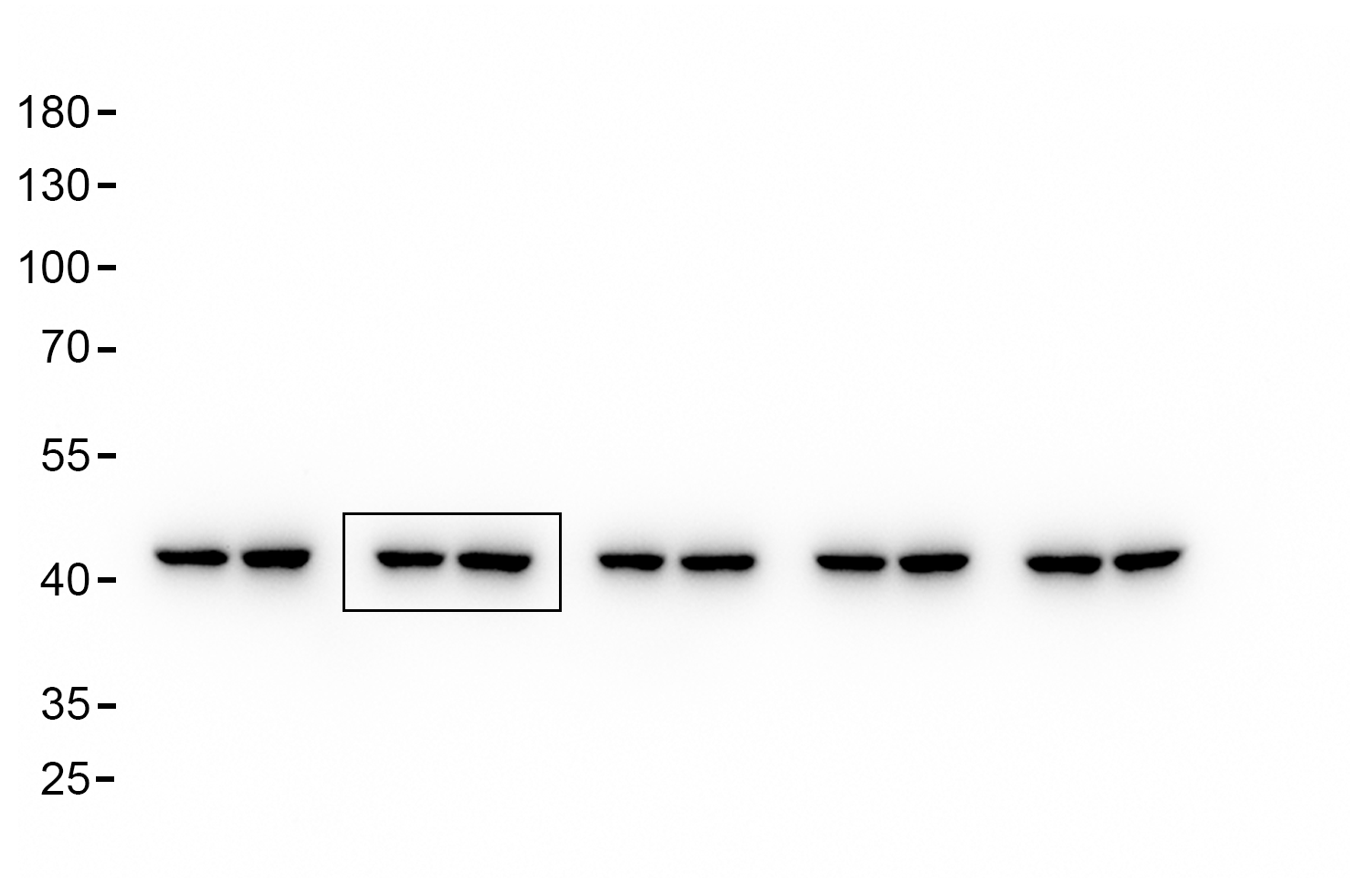

Supplement: Figure 4—figure supplement 2—source data 3. [file elife-97373-fig4-figsupp2-data3.zip › Figure 4-figure supplement 2-source data 2/Figure 4-figure supplement 2A/Actin.tif]

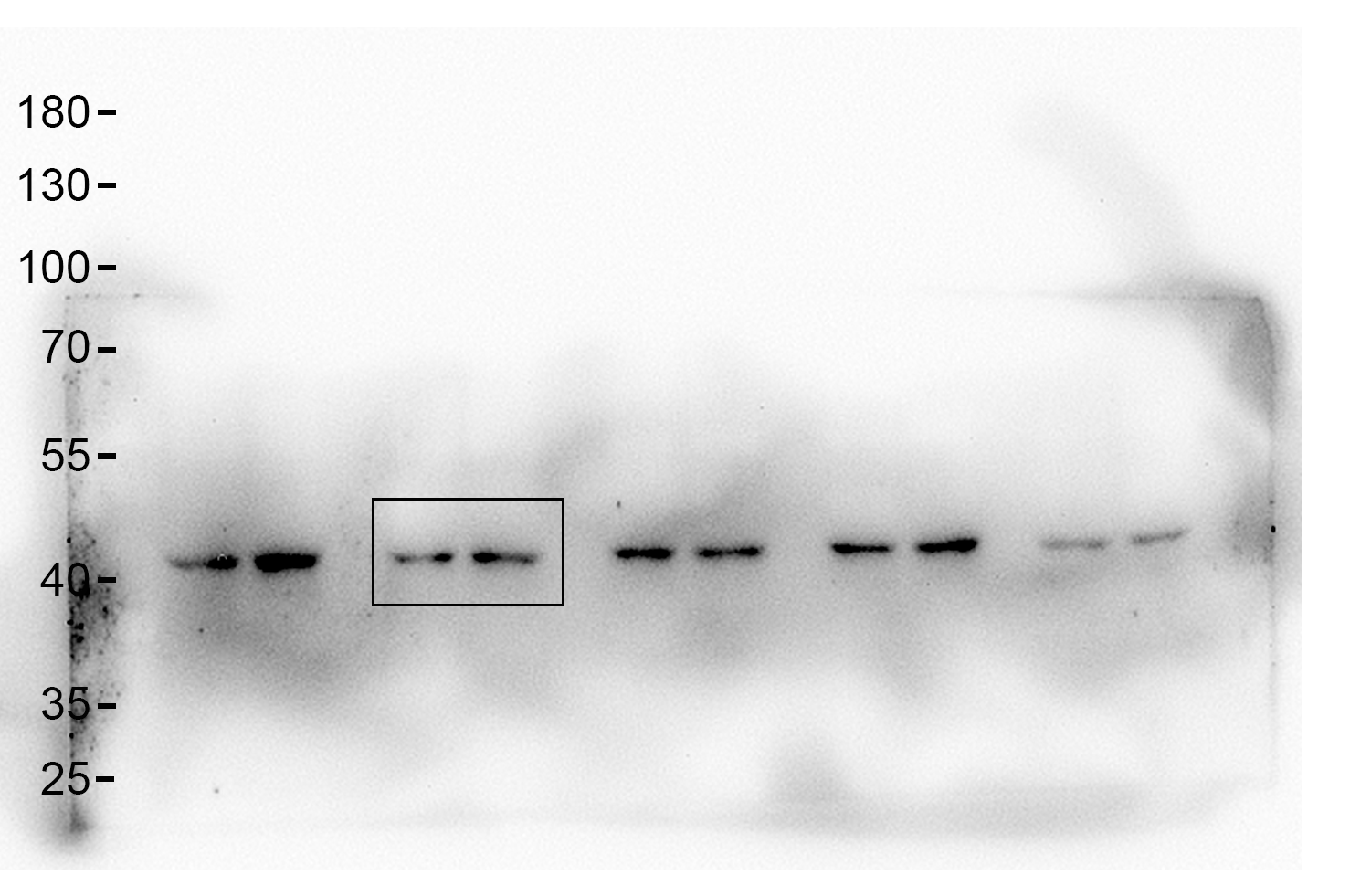

Supplement: Figure 4—figure supplement 2—source data 3. [file elife-97373-fig4-figsupp2-data3.zip › Figure 4-figure supplement 2-source data 2/Figure 4-figure supplement 2A/ERK.tif]

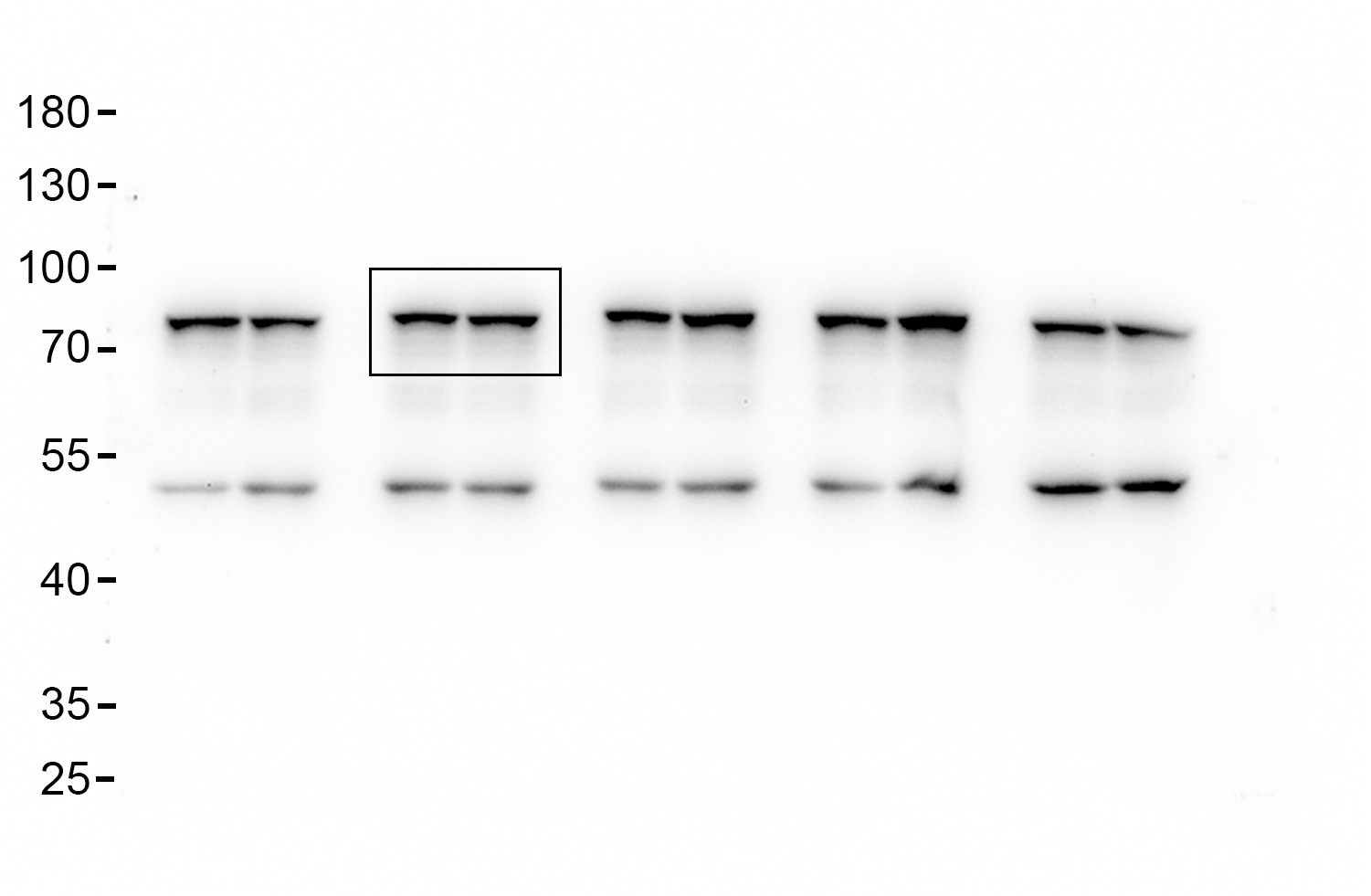

Supplement: Figure 4—figure supplement 2—source data 3. [file elife-97373-fig4-figsupp2-data3.zip › Figure 4-figure supplement 2-source data 2/Figure 4-figure supplement 2A/IKK.tif]

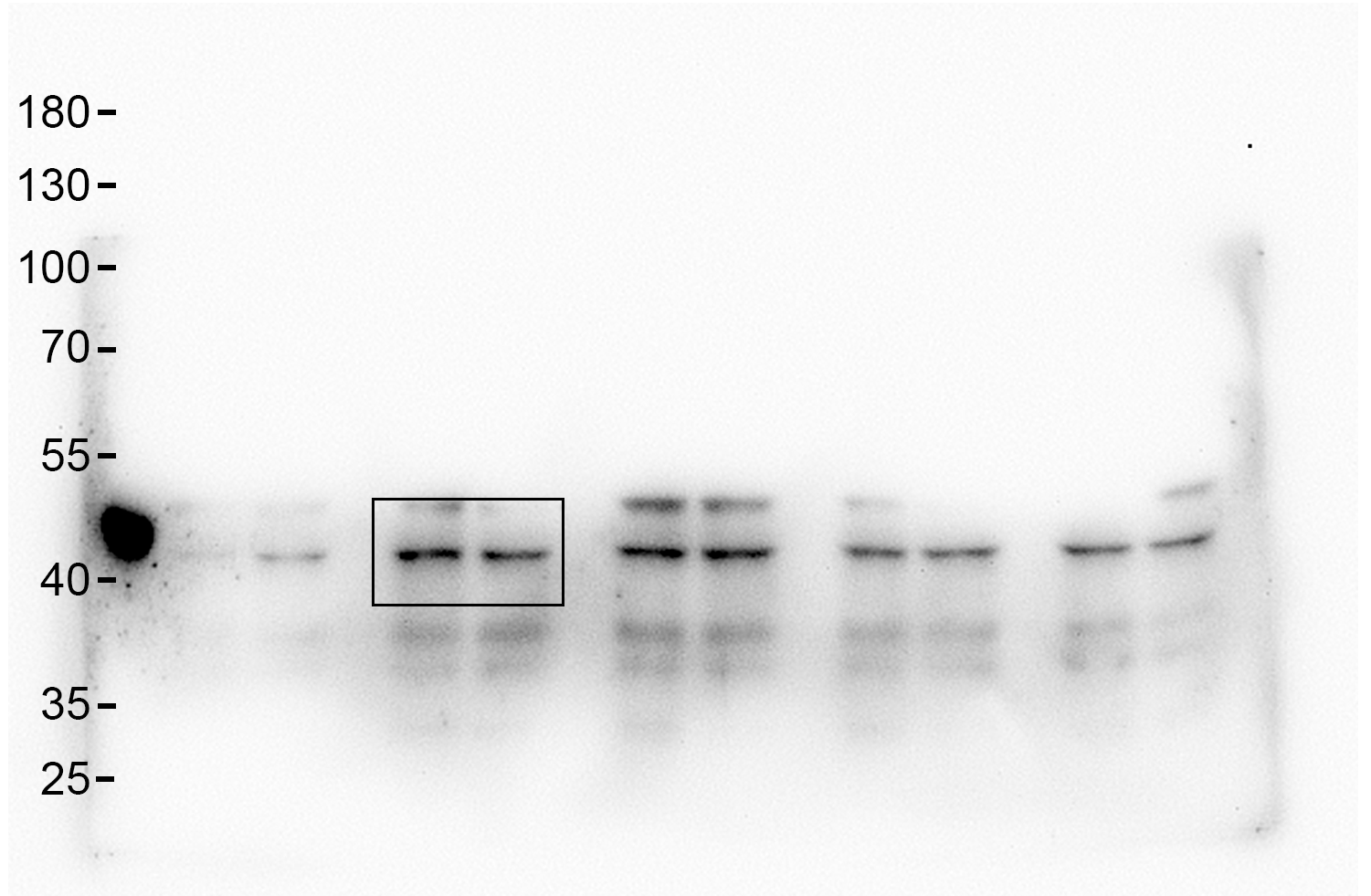

Supplement: Figure 4—figure supplement 2—source data 3. [file elife-97373-fig4-figsupp2-data3.zip › Figure 4-figure supplement 2-source data 2/Figure 4-figure supplement 2A/JNK.tif]

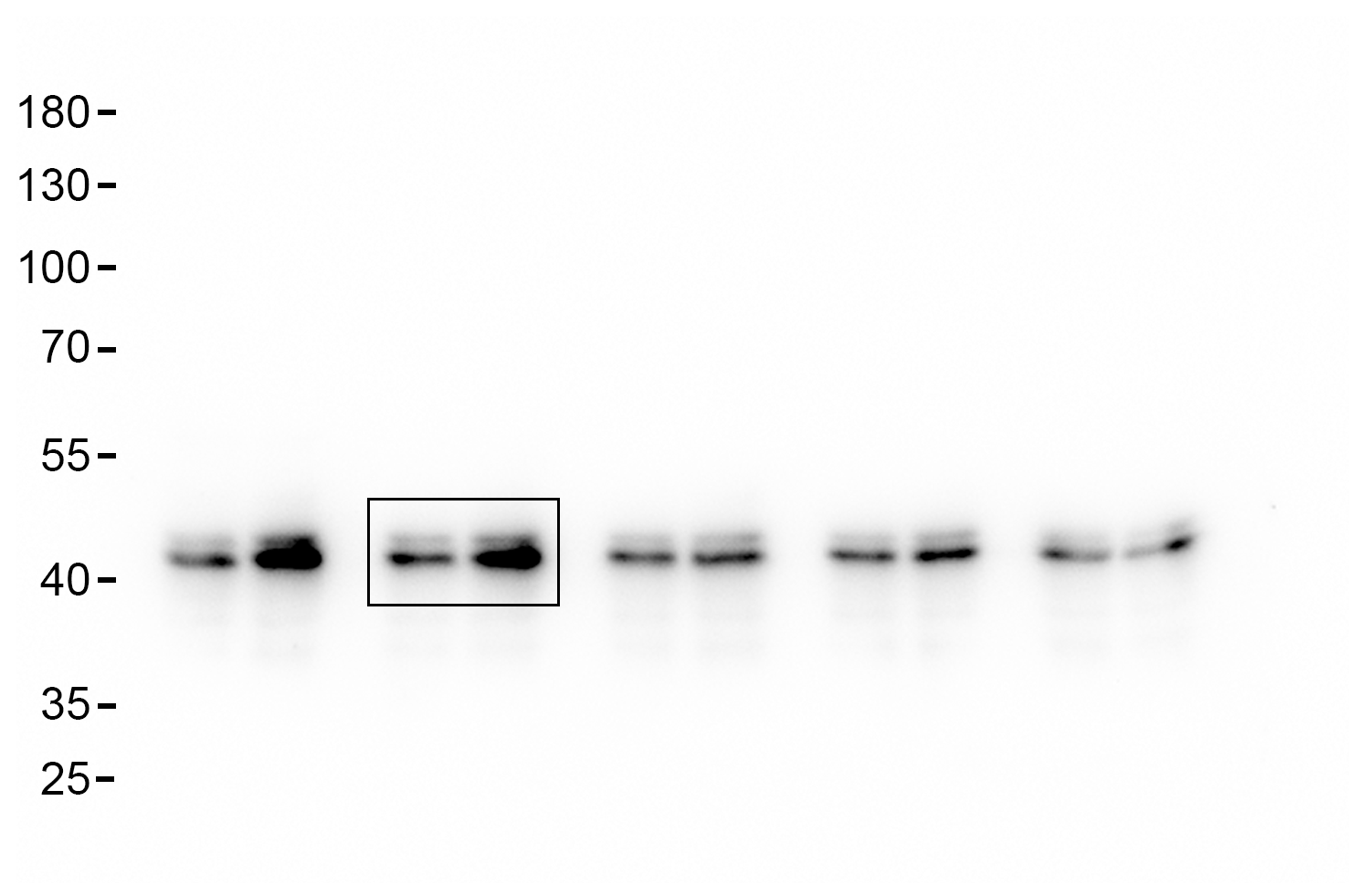

Supplement: Figure 4—figure supplement 2—source data 3. [file elife-97373-fig4-figsupp2-data3.zip › Figure 4-figure supplement 2-source data 2/Figure 4-figure supplement 2A/p-ERK.tif]

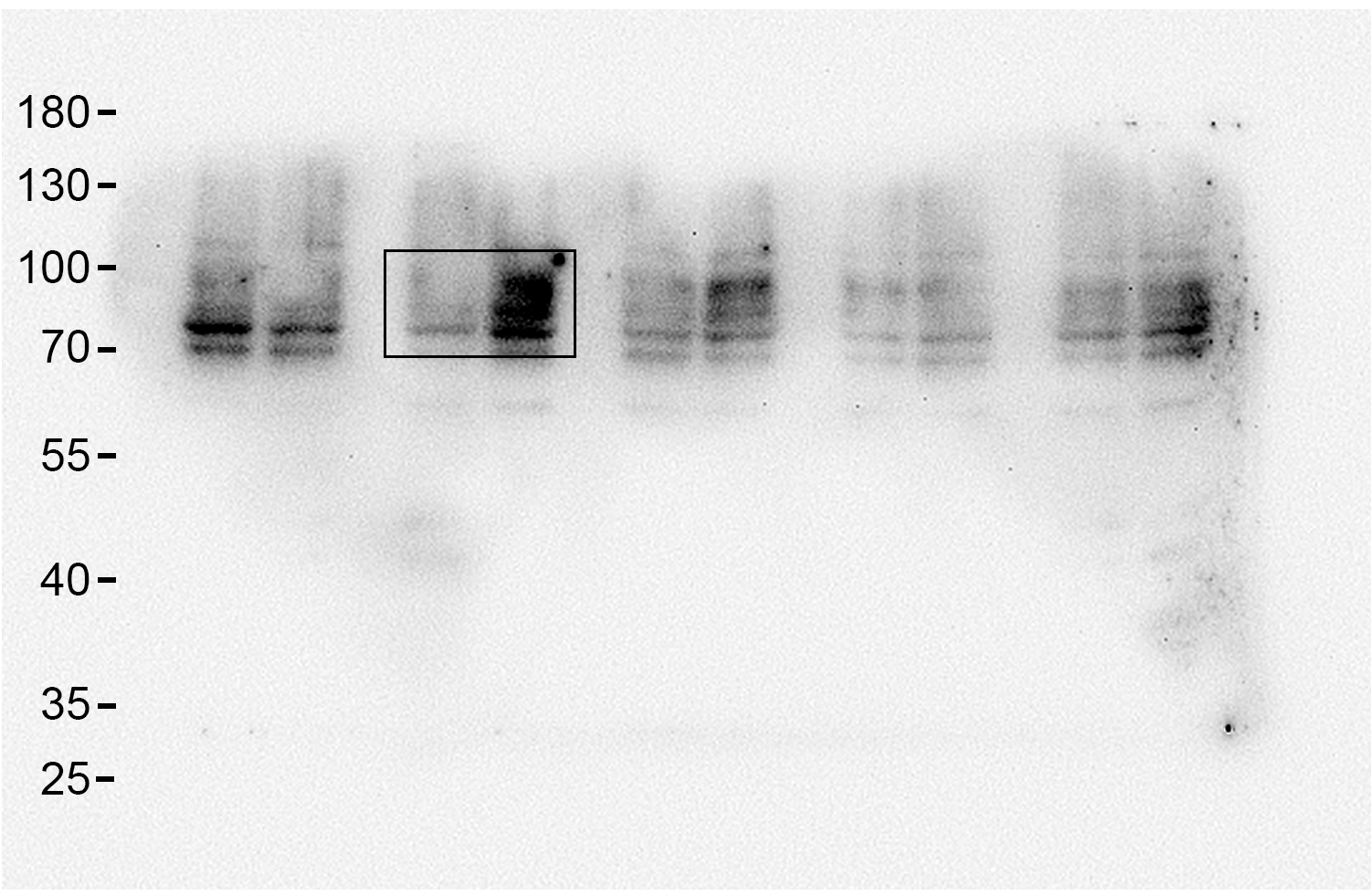

Supplement: Figure 4—figure supplement 2—source data 3. [file elife-97373-fig4-figsupp2-data3.zip › Figure 4-figure supplement 2-source data 2/Figure 4-figure supplement 2A/p-IKK.tif]

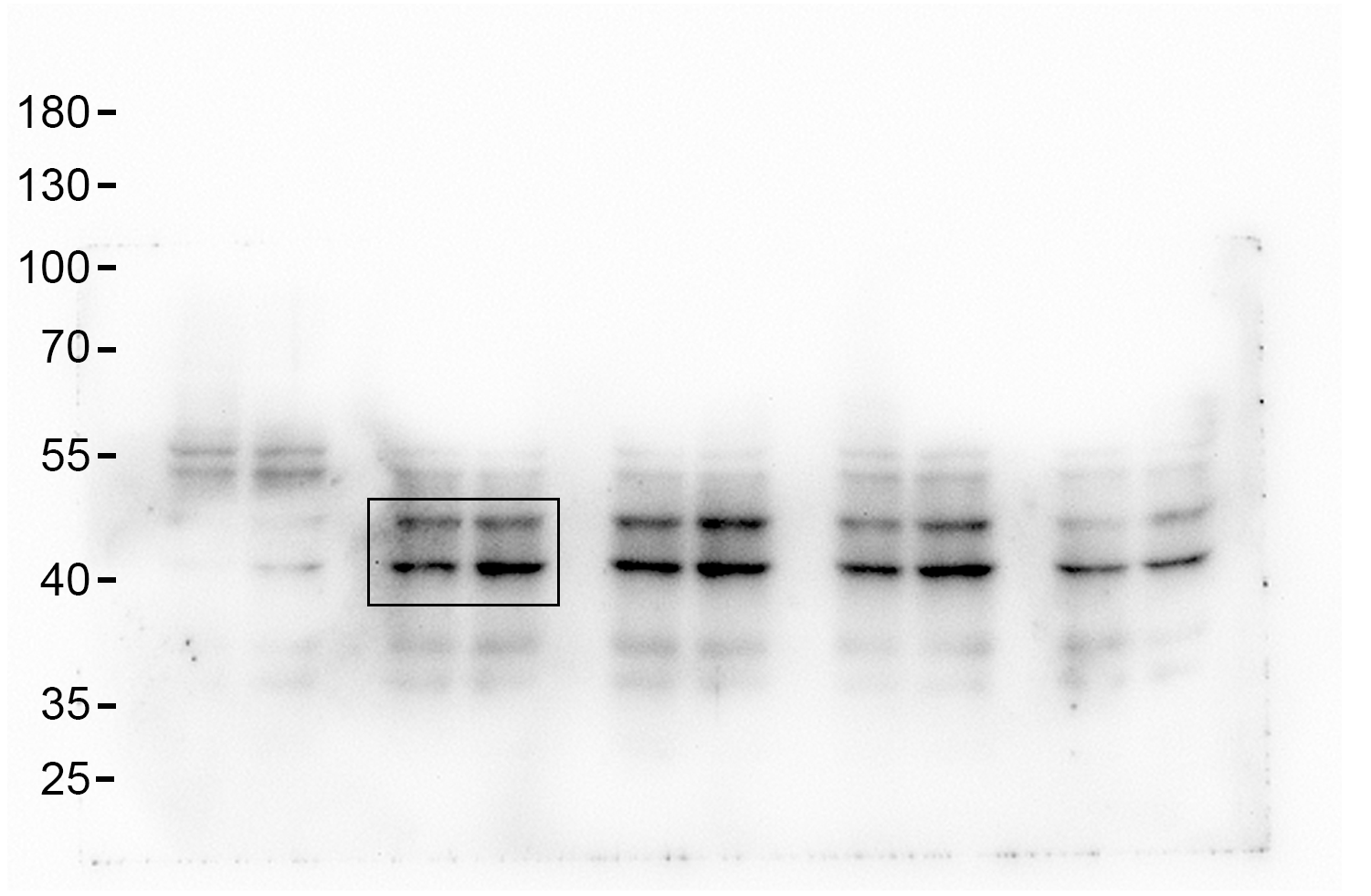

Supplement: Figure 4—figure supplement 2—source data 3. [file elife-97373-fig4-figsupp2-data3.zip › Figure 4-figure supplement 2-source data 2/Figure 4-figure supplement 2A/p-JNK.tif]

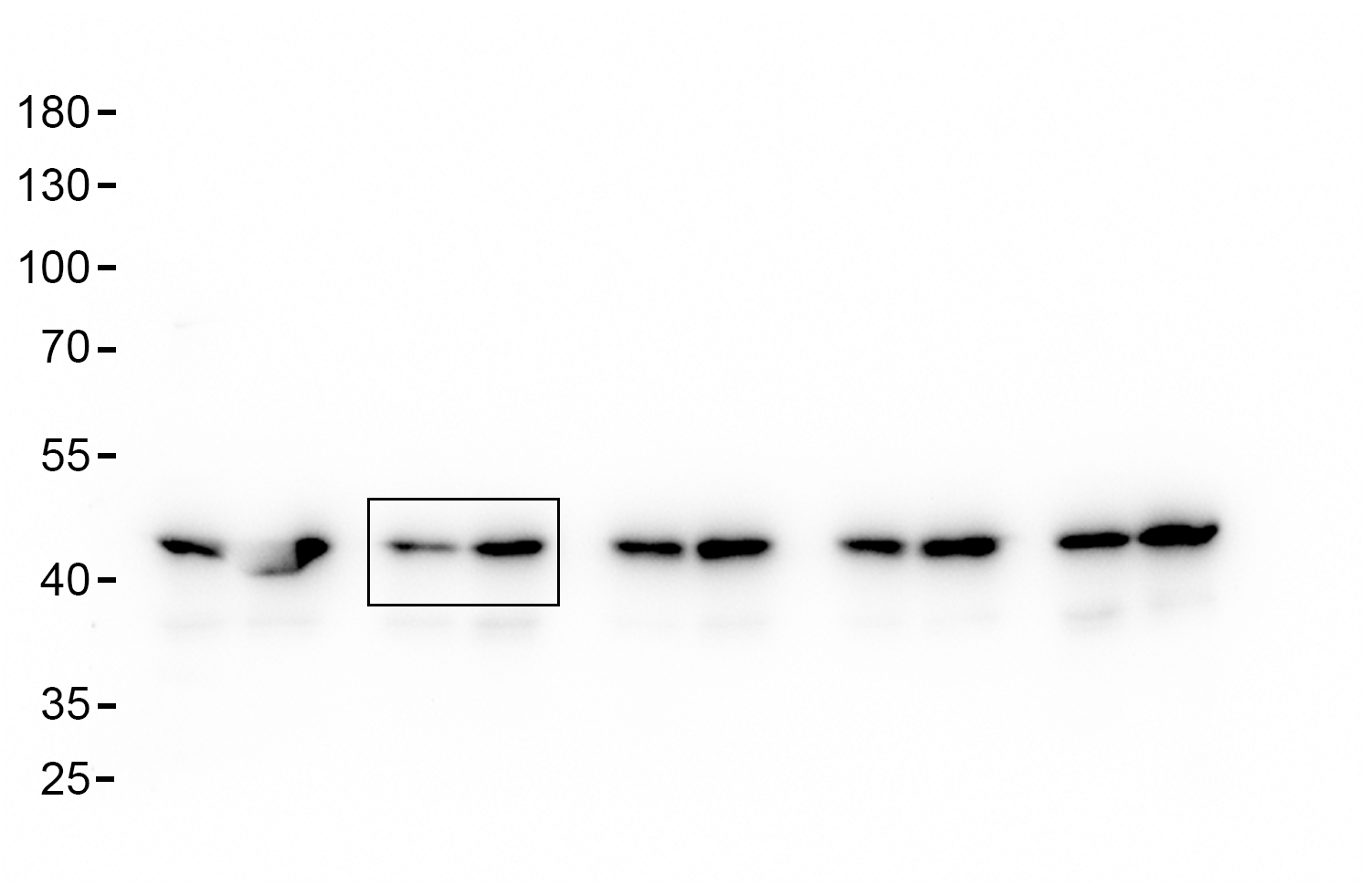

Supplement: Figure 4—figure supplement 2—source data 3. [file elife-97373-fig4-figsupp2-data3.zip › Figure 4-figure supplement 2-source data 2/Figure 4-figure supplement 2A/p-P38.tif]

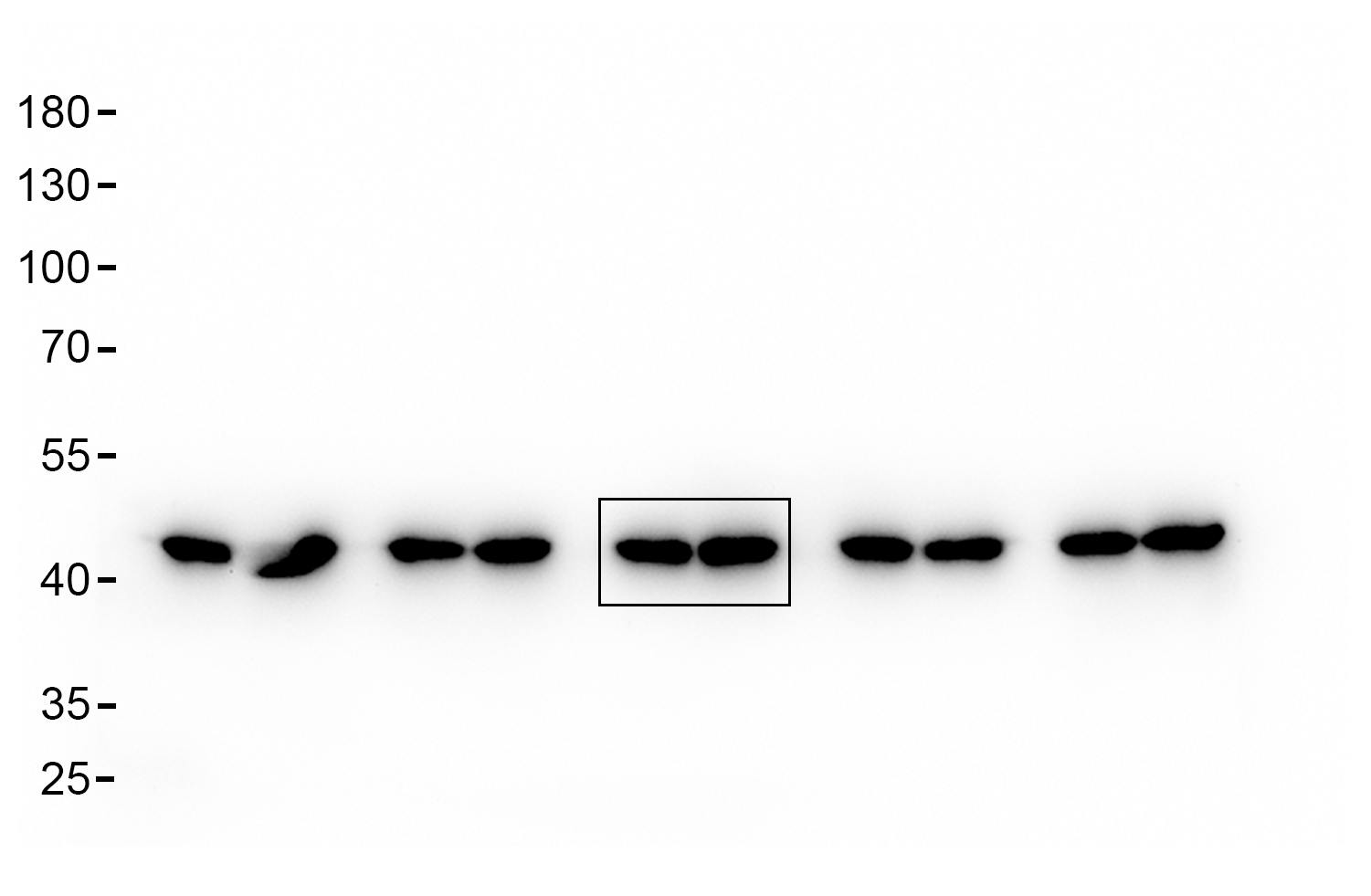

Supplement: Figure 4—figure supplement 2—source data 3. [file elife-97373-fig4-figsupp2-data3.zip › Figure 4-figure supplement 2-source data 2/Figure 4-figure supplement 2A/P38.tif]

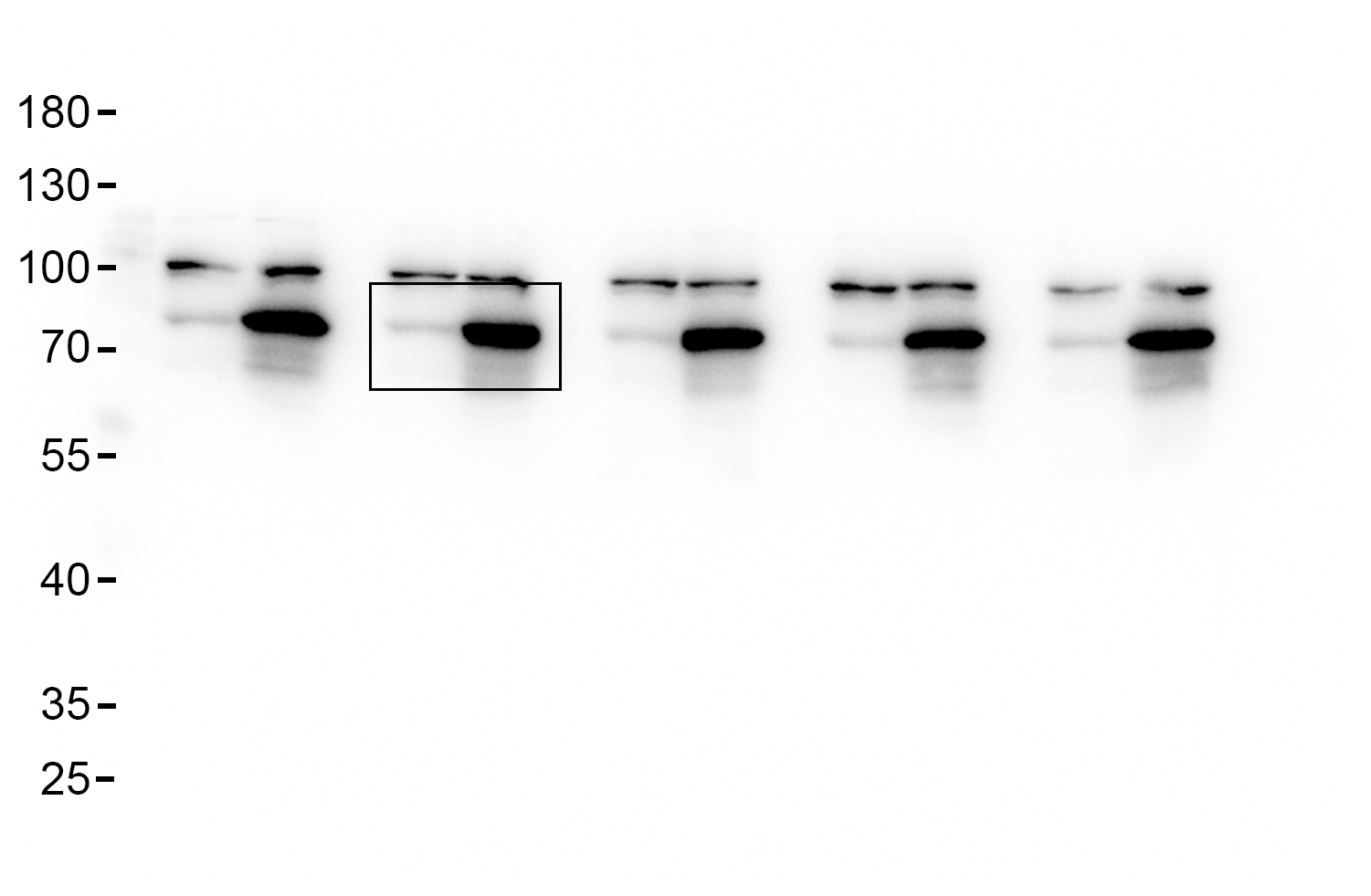

Supplement: Figure 4—figure supplement 2—source data 3. [file elife-97373-fig4-figsupp2-data3.zip › Figure 4-figure supplement 2-source data 2/Figure 4-figure supplement 2A/TAK1.tif]

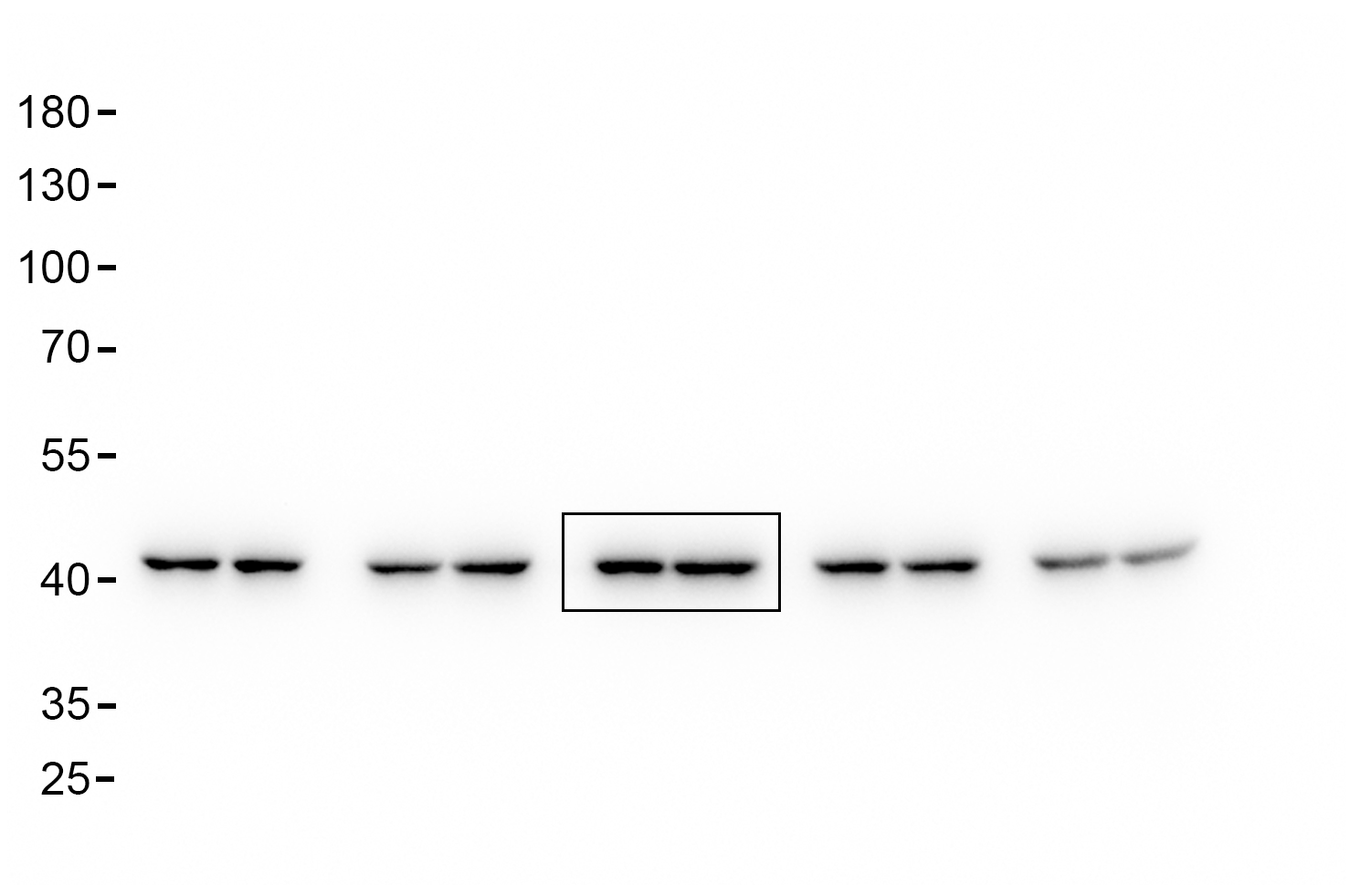

Supplement: Figure 4—figure supplement 2—source data 3. [file elife-97373-fig4-figsupp2-data3.zip › Figure 4-figure supplement 2-source data 2/Figure 4-figure supplement 2C/Actin.tif]

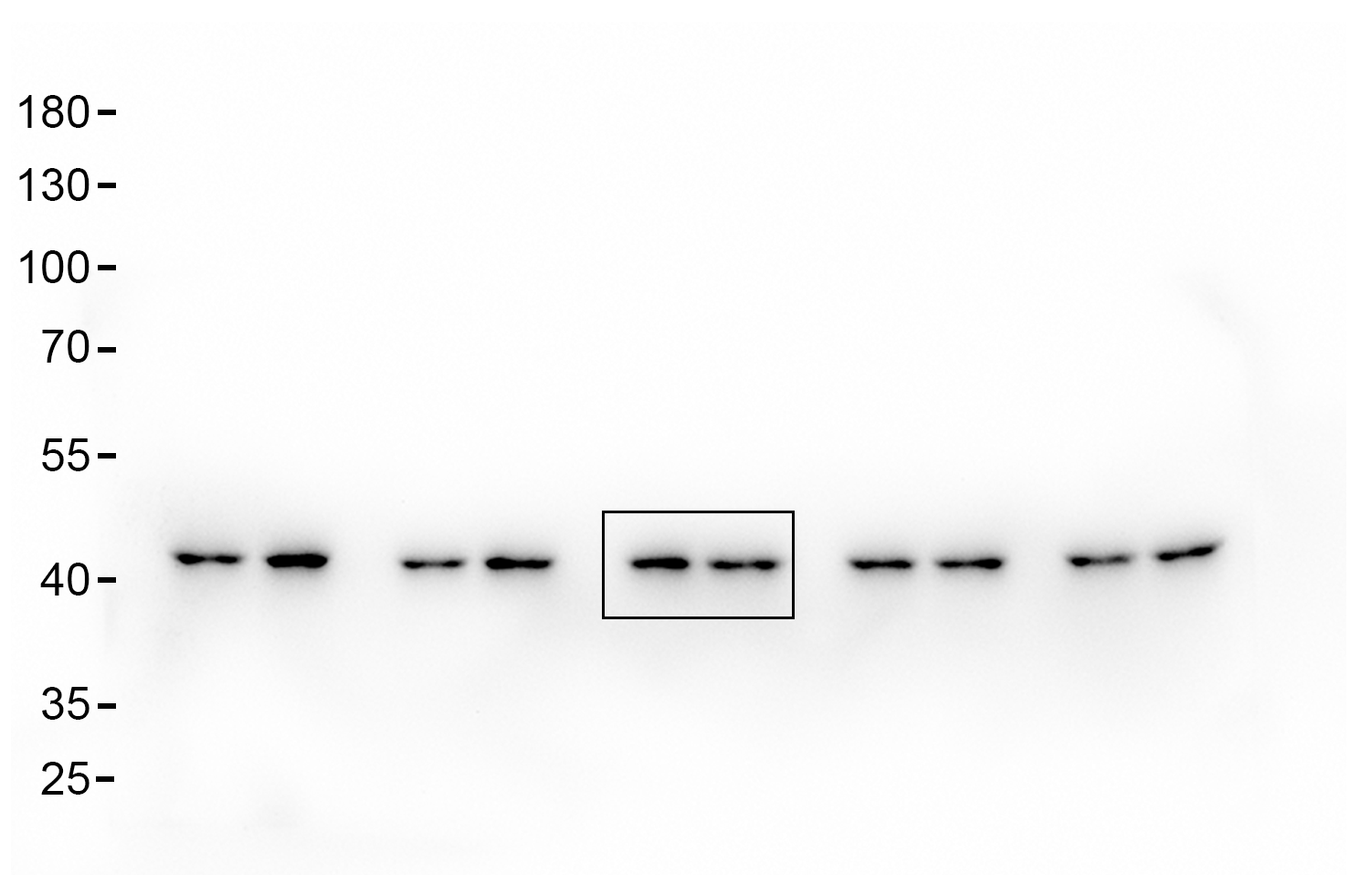

Supplement: Figure 4—figure supplement 2—source data 3. [file elife-97373-fig4-figsupp2-data3.zip › Figure 4-figure supplement 2-source data 2/Figure 4-figure supplement 2C/ERK.tif]

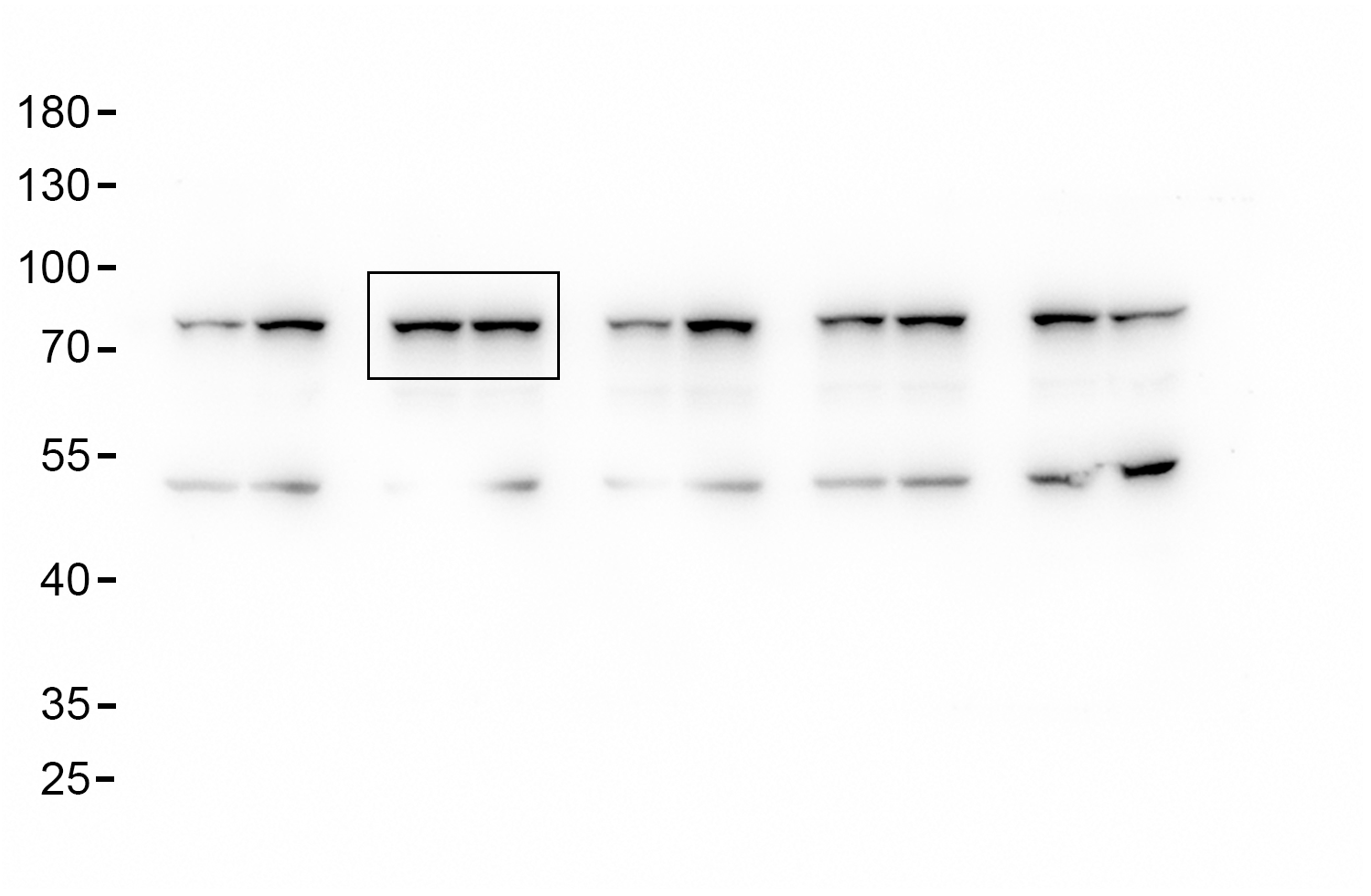

Supplement: Figure 4—figure supplement 2—source data 3. [file elife-97373-fig4-figsupp2-data3.zip › Figure 4-figure supplement 2-source data 2/Figure 4-figure supplement 2C/IKK.tif]

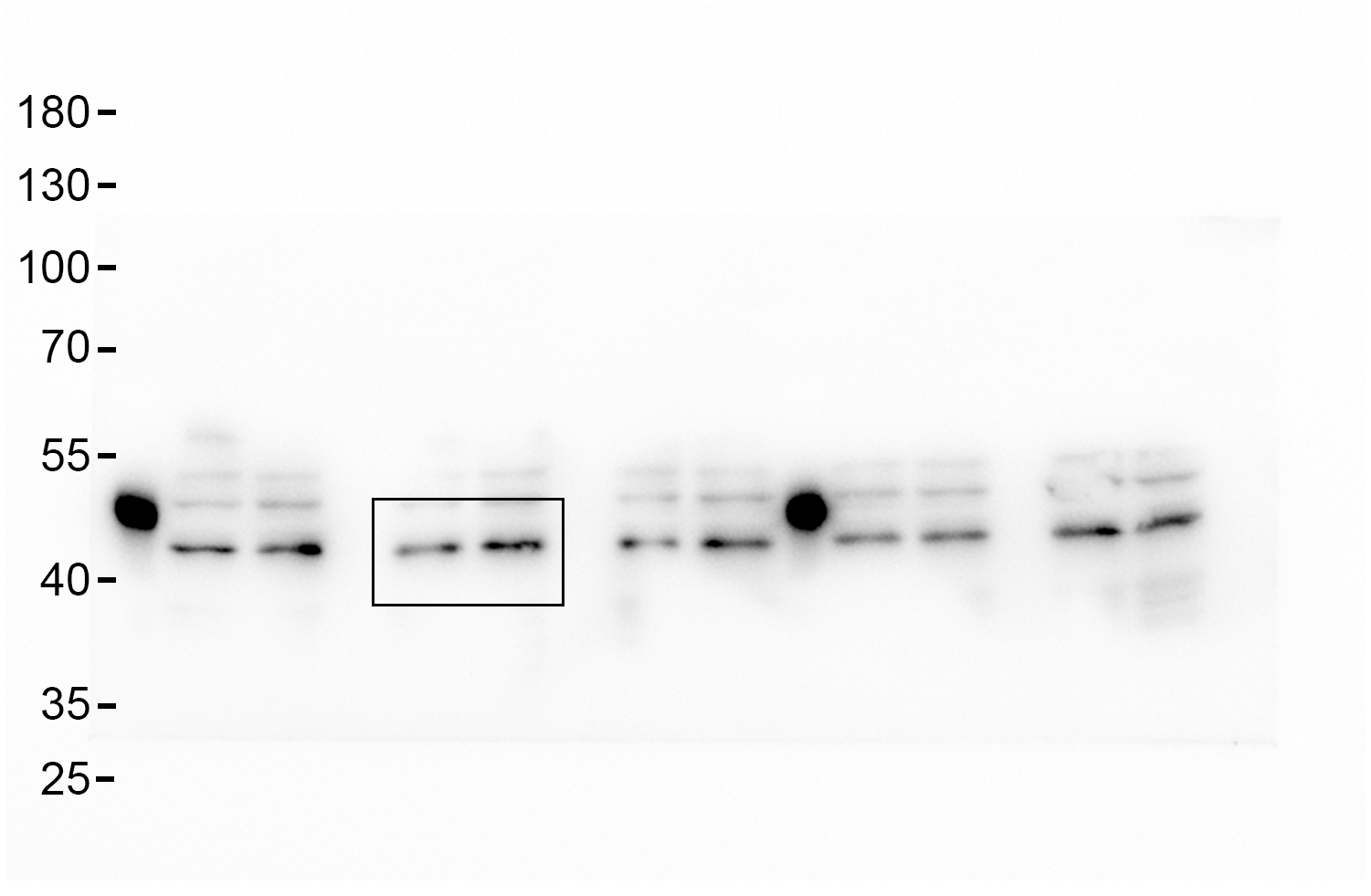

Supplement: Figure 4—figure supplement 2—source data 3. [file elife-97373-fig4-figsupp2-data3.zip › Figure 4-figure supplement 2-source data 2/Figure 4-figure supplement 2C/JNK.tif]

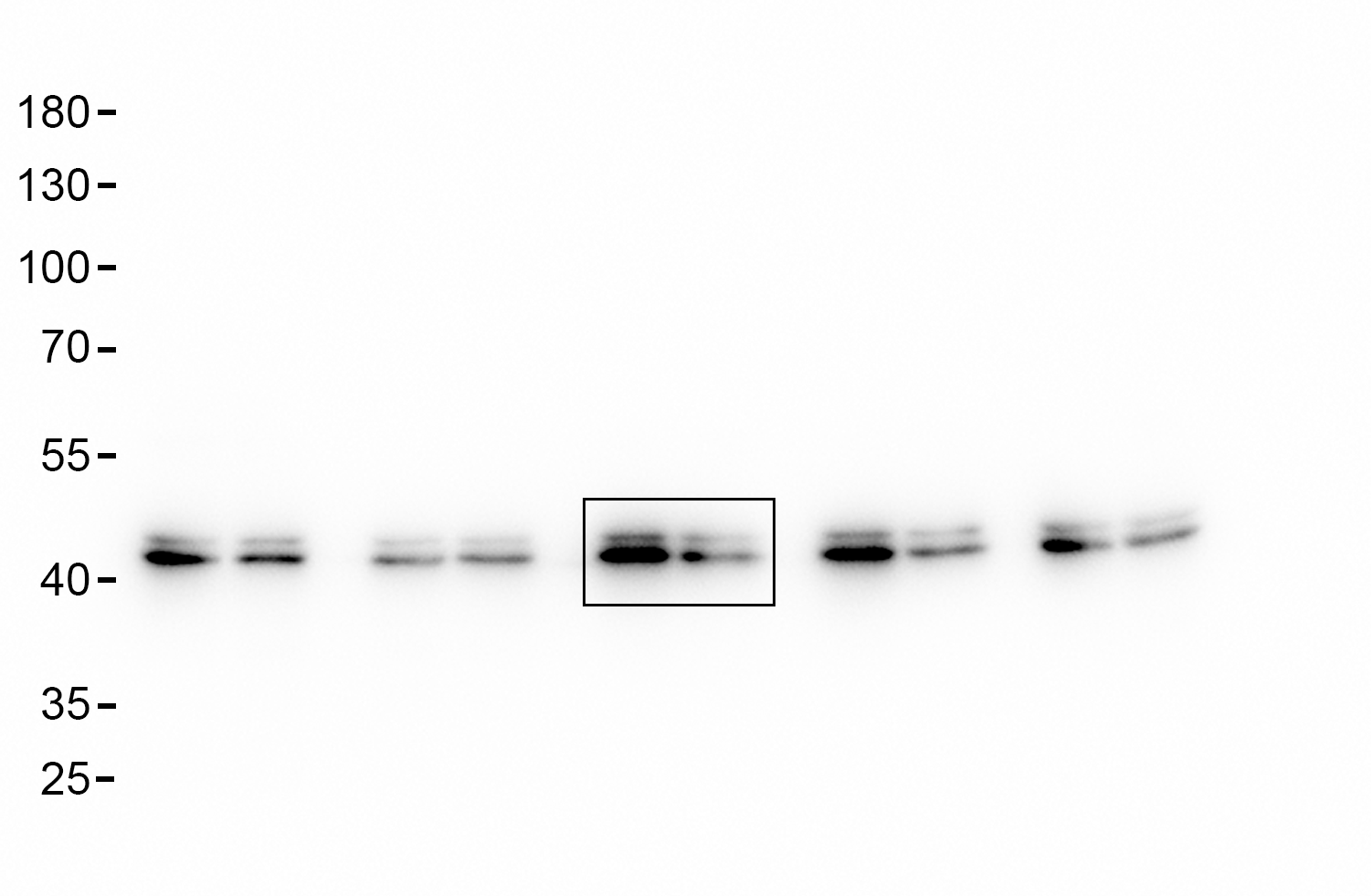

Supplement: Figure 4—figure supplement 2—source data 3. [file elife-97373-fig4-figsupp2-data3.zip › Figure 4-figure supplement 2-source data 2/Figure 4-figure supplement 2C/p-ERK.tif]

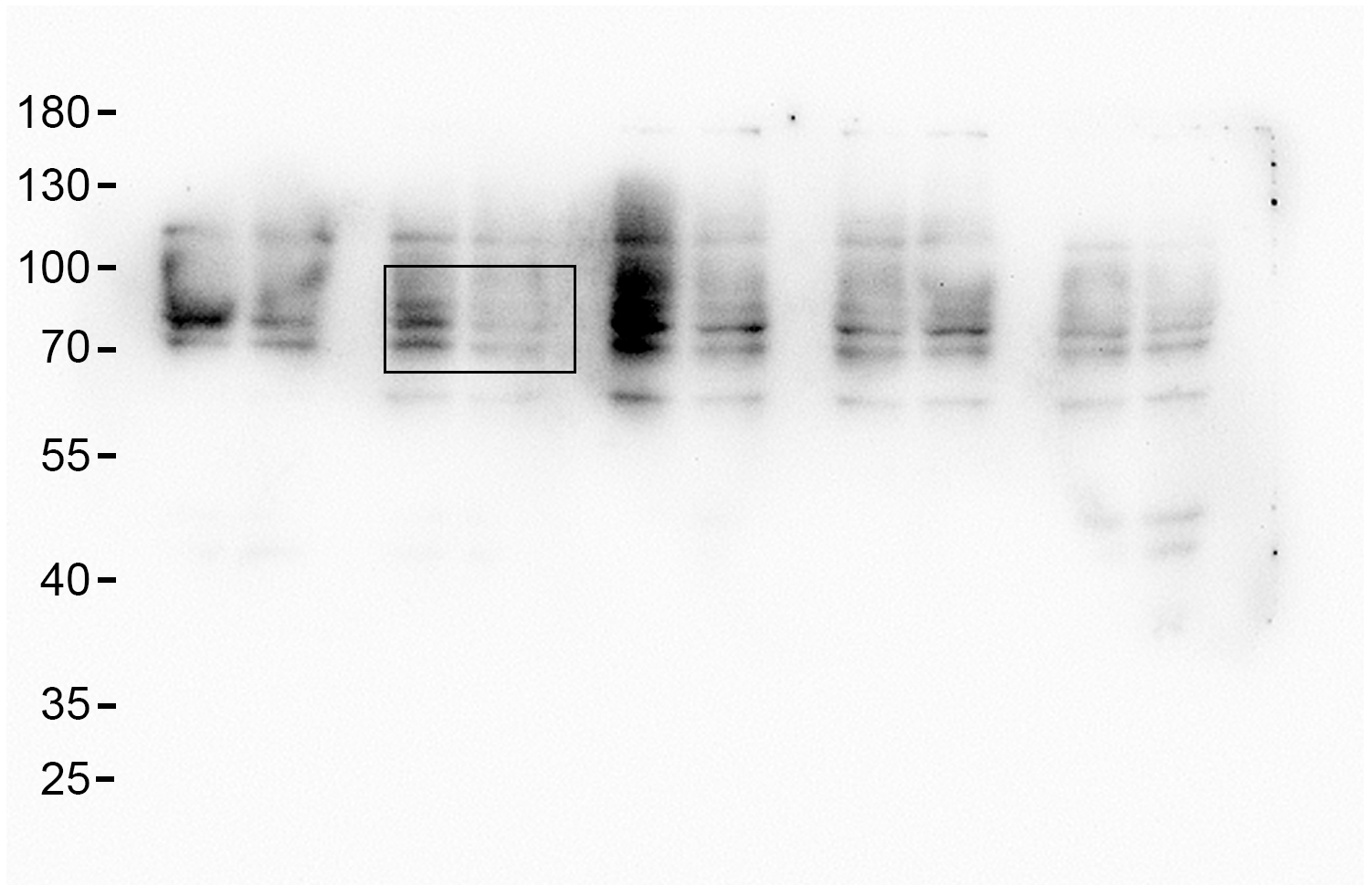

Supplement: Figure 4—figure supplement 2—source data 3. [file elife-97373-fig4-figsupp2-data3.zip › Figure 4-figure supplement 2-source data 2/Figure 4-figure supplement 2C/p-IKK.tif]
