# Supplementary material for: TAK1-mediated phosphorylation of PLCE1 represses PIP2 hydrolysis to impede esophageal squamous cancer metastasis
Source: eLife. 2025 Apr 23;13:RP97373. doi: 10.7554/eLife.97373 (PMC12017773; doi:10.7554/eLife.97373)
Supplement: Figure 1—figure supplement 4—source data 2. [file elife-97373-fig1-figsupp4-data2.zip › Figure 1-figure supplement 4-source data 1/Figure 1-figure supplement 4-source data 1.pdf]

Figure 1–Figure Supplement 4A

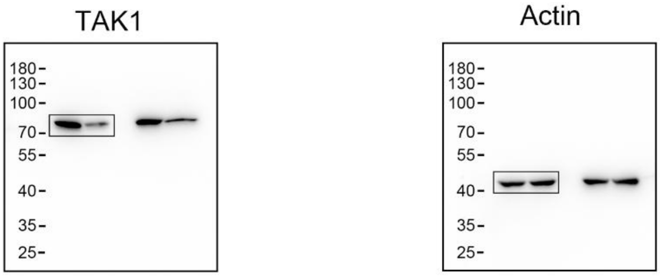

Figure 1–Figure Supplement 4E

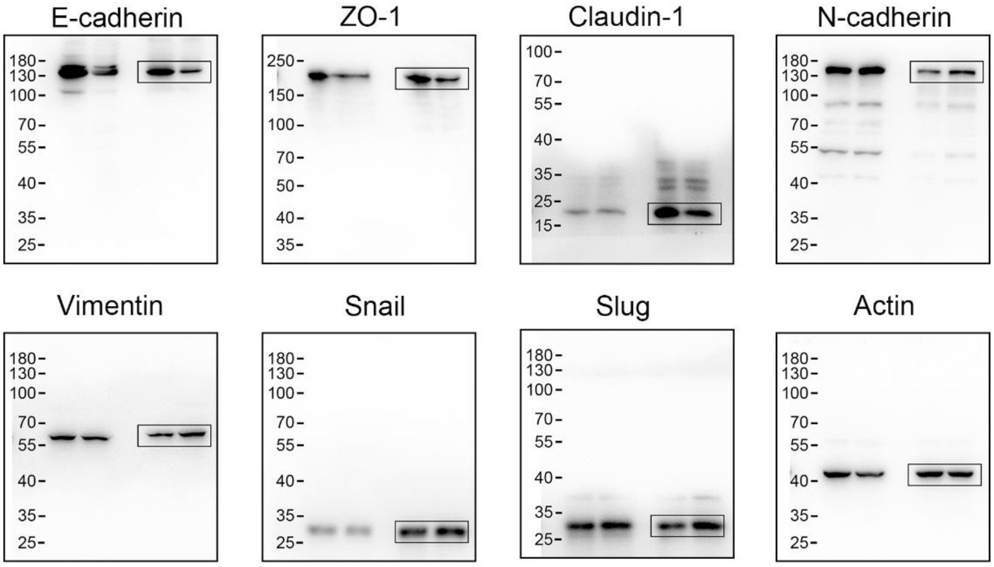

Figure 1-figure supplement 4, Source Data 1. Original membranes corresponding to Figure 1-figure supplement 4, panel A and E, indicating the relevant bands.
