# Supplementary material for: TAK1-mediated phosphorylation of PLCE1 represses PIP2 hydrolysis to impede esophageal squamous cancer metastasis
Source: eLife. 2025 Apr 23;13:RP97373. doi: 10.7554/eLife.97373 (PMC12017773; doi:10.7554/eLife.97373)
Supplement: Figure 3—source data 2. [file elife-97373-fig3-data2.zip › Figure 3-source data 1/Figure 3-source data 1.pdf]

Figure 3A

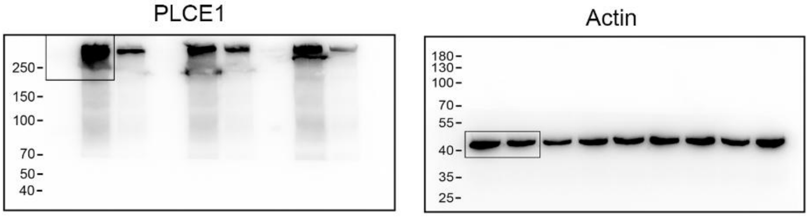

Figure 3D

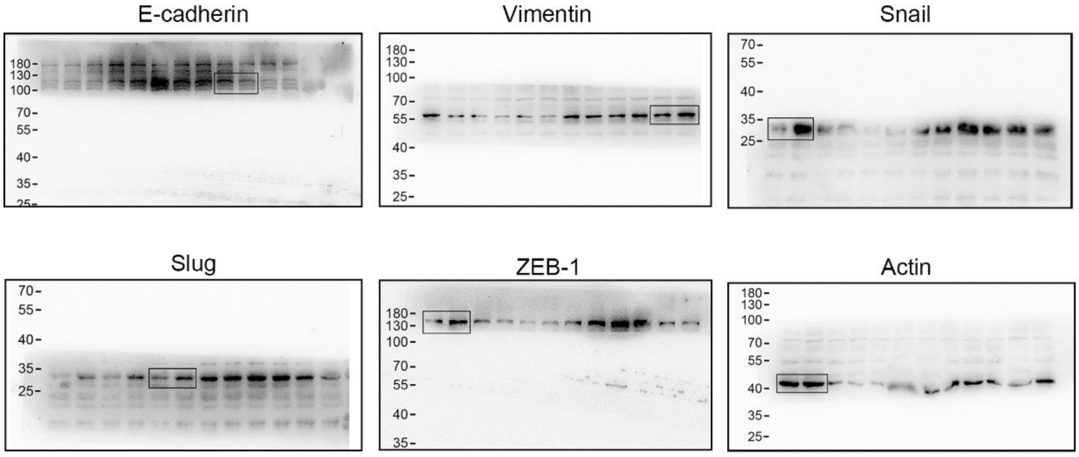

Figure 3F

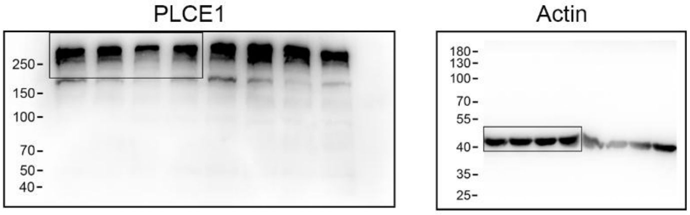

Figure 3I

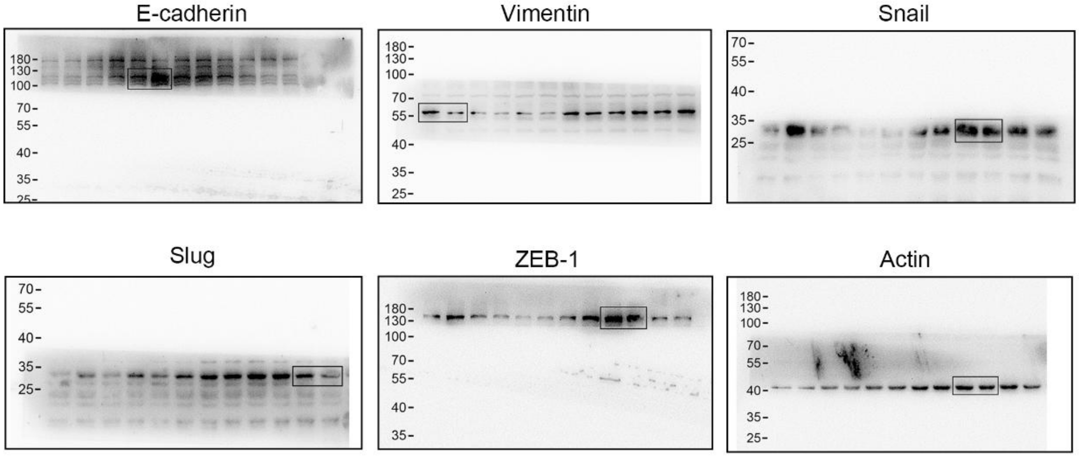

Figure 3, Source Data 1. Original membranes corresponding to Figure 3, panel A, D, F and I, indicating the relevant bands.
