# Supplementary material for: TAK1-mediated phosphorylation of PLCE1 represses PIP2 hydrolysis to impede esophageal squamous cancer metastasis
Source: eLife. 2025 Apr 23;13:RP97373. doi: 10.7554/eLife.97373 (PMC12017773; doi:10.7554/eLife.97373)
Supplement: Figure 4—figure supplement 2—source data 2. [file elife-97373-fig4-figsupp2-data2.zip › Figure 4-figure supplement 2-source data 1/Figure 4-figure supplement 2-source data 1.pdf]

Figure 4–Figure Supplement 2A

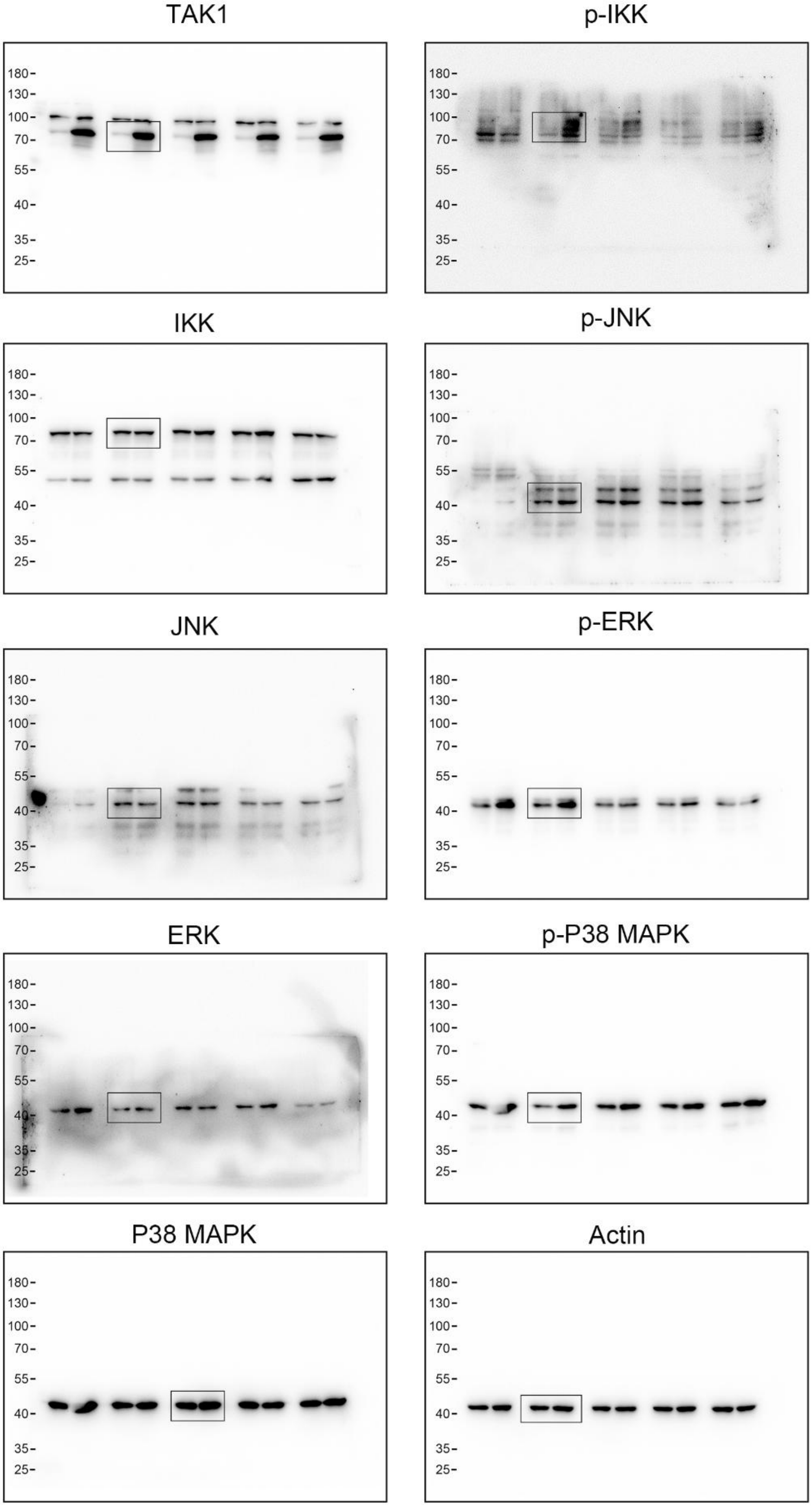

Figure 4–Figure Supplement 2C

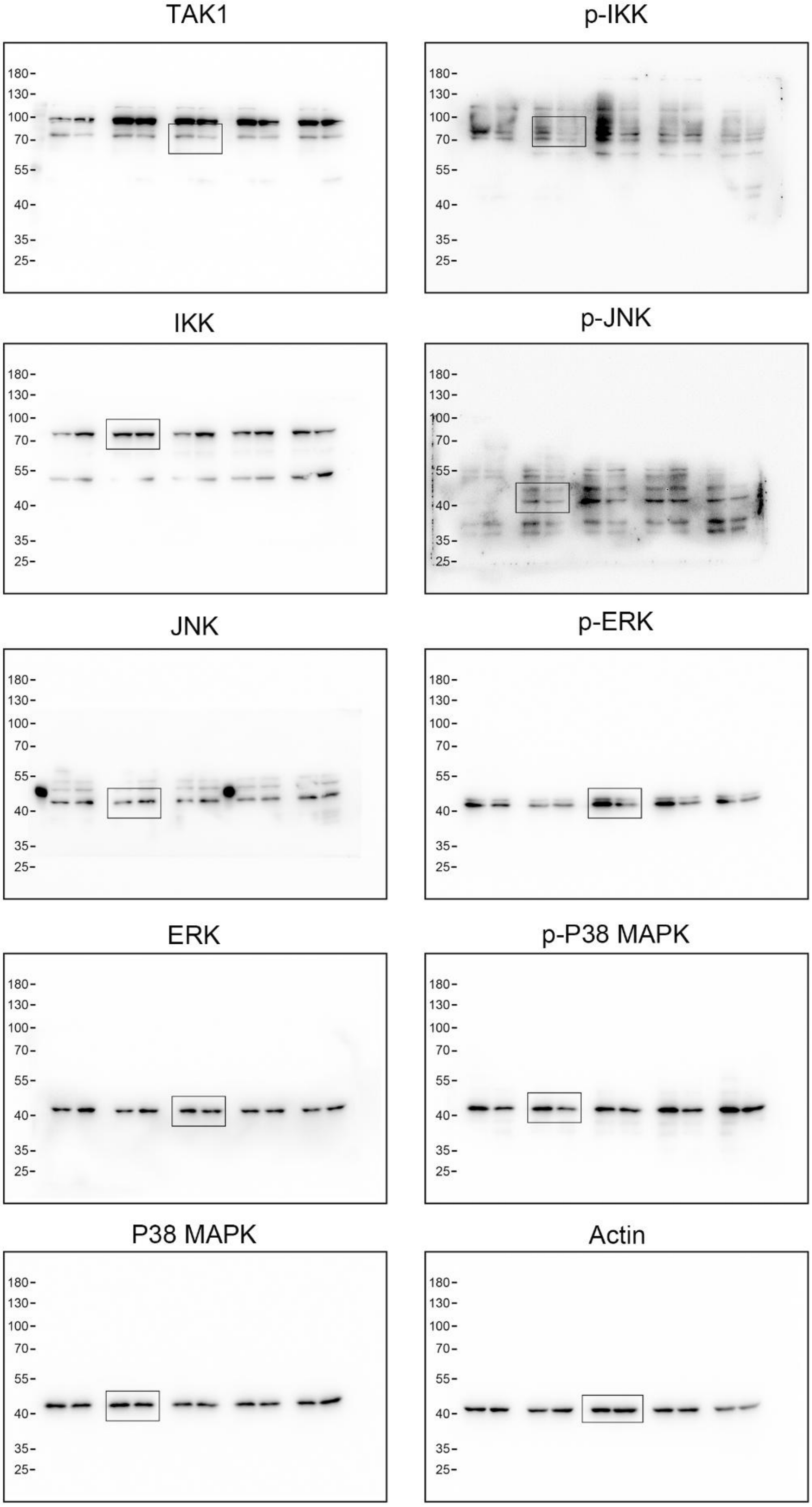

Figure 4-figure supplement 2, Source Data 1. Original membranes corresponding to Figure 4-figure supplement 2, panel A and C, indicating the relevant bands.
