# Supplementary figures and images for: TAK1-mediated phosphorylation of PLCE1 represses PIP2 hydrolysis to impede esophageal squamous cancer metastasis (part 2 of 2)
Source: eLife. 2025 Apr 23;13:RP97373. doi: 10.7554/eLife.97373 (PMC12017773; doi:10.7554/eLife.97373)

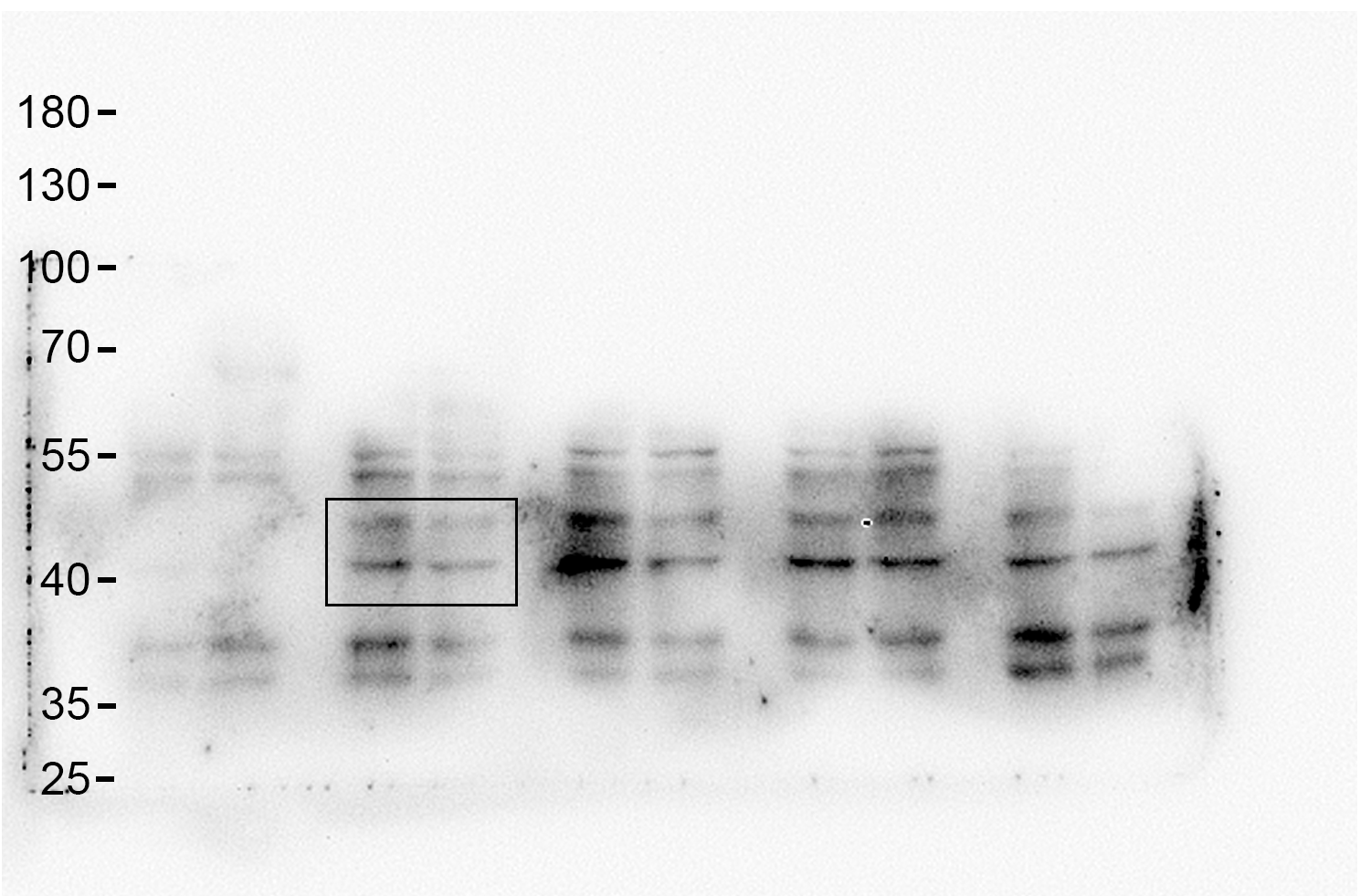

Supplement: Figure 4—figure supplement 2—source data 3. [file elife-97373-fig4-figsupp2-data3.zip › Figure 4-figure supplement 2-source data 2/Figure 4-figure supplement 2C/p-JNK.tif]

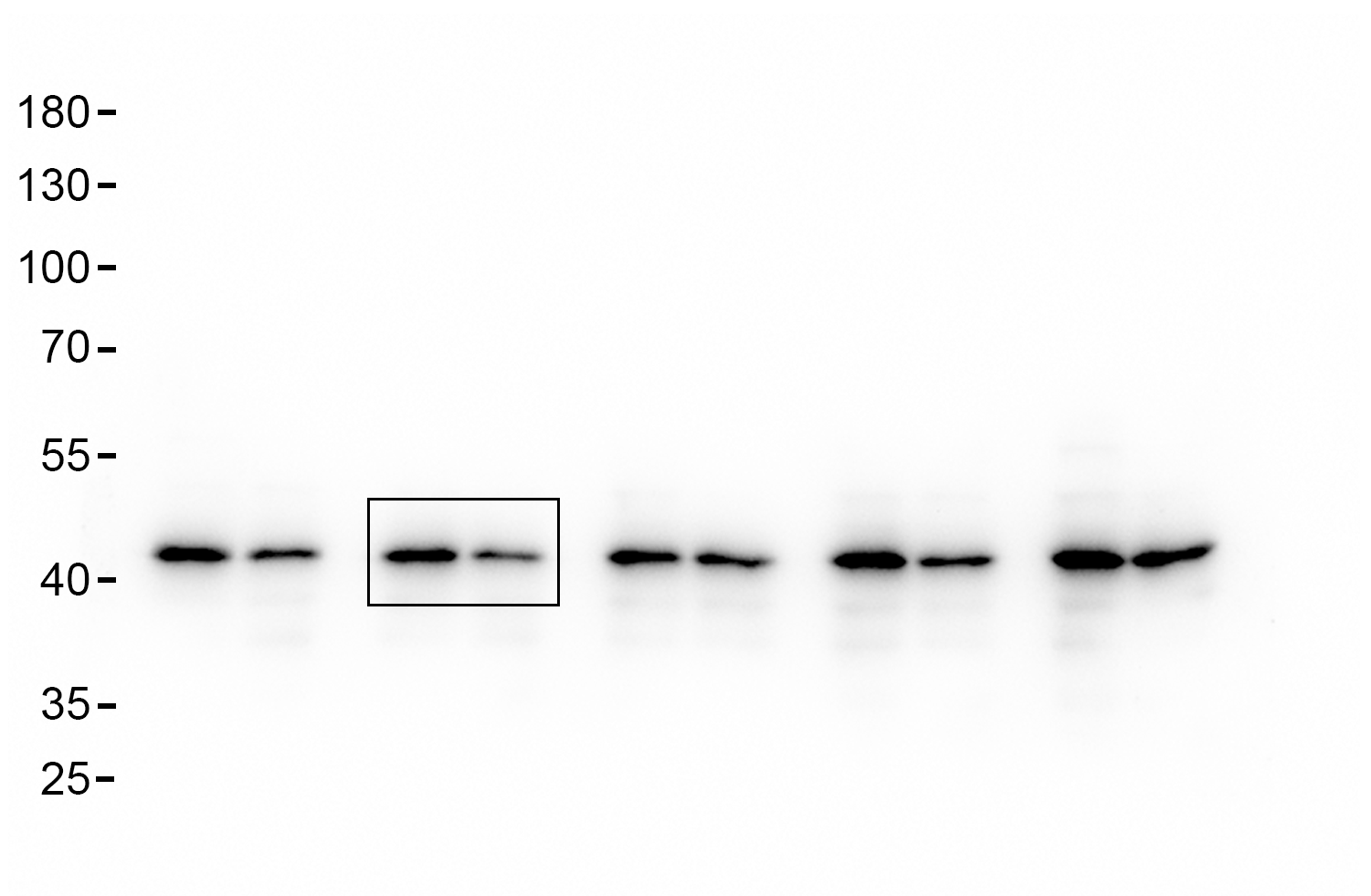

Supplement: Figure 4—figure supplement 2—source data 3. [file elife-97373-fig4-figsupp2-data3.zip › Figure 4-figure supplement 2-source data 2/Figure 4-figure supplement 2C/p-P38.tif]

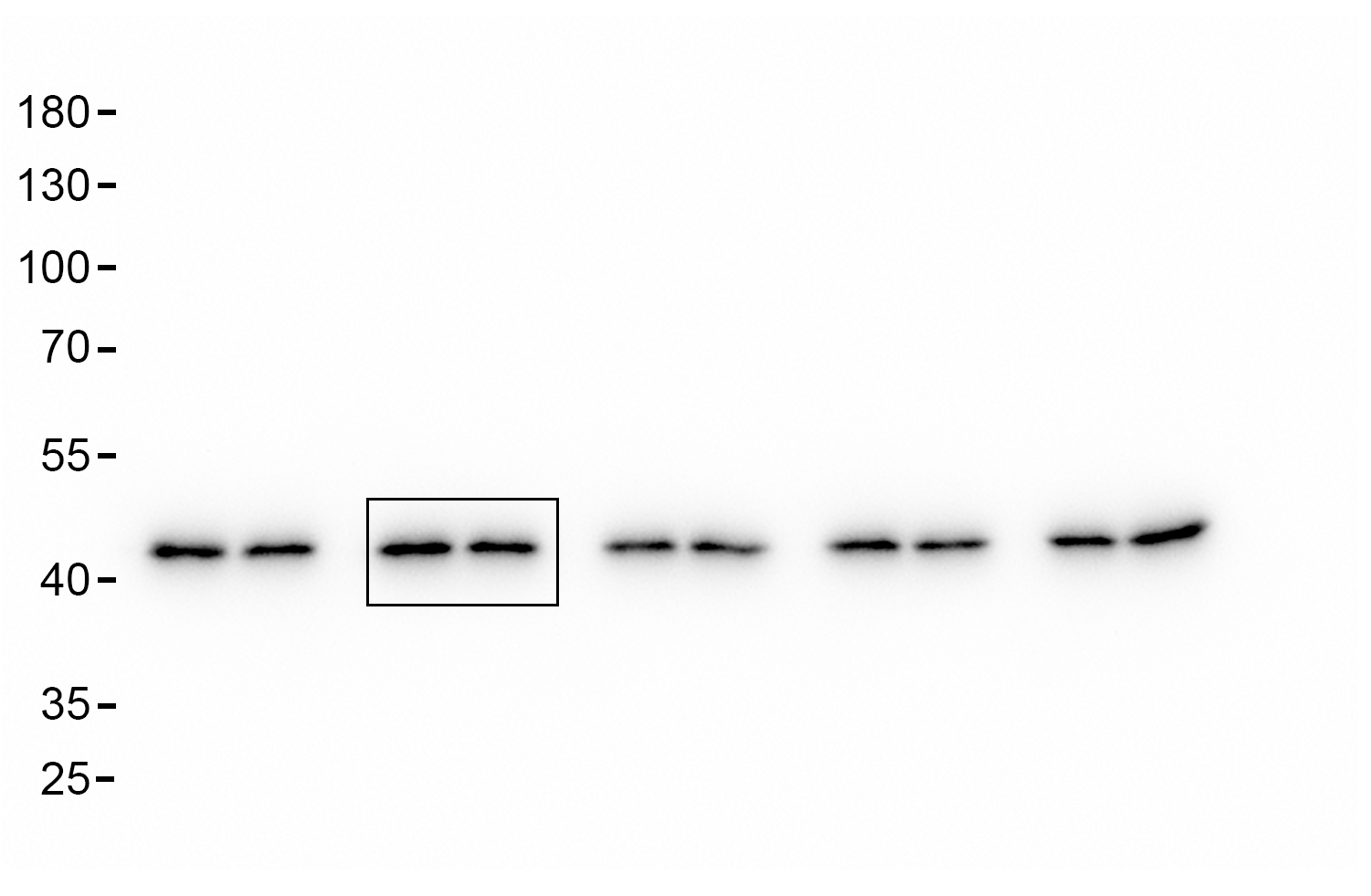

Supplement: Figure 4—figure supplement 2—source data 3. [file elife-97373-fig4-figsupp2-data3.zip › Figure 4-figure supplement 2-source data 2/Figure 4-figure supplement 2C/P38.tif]

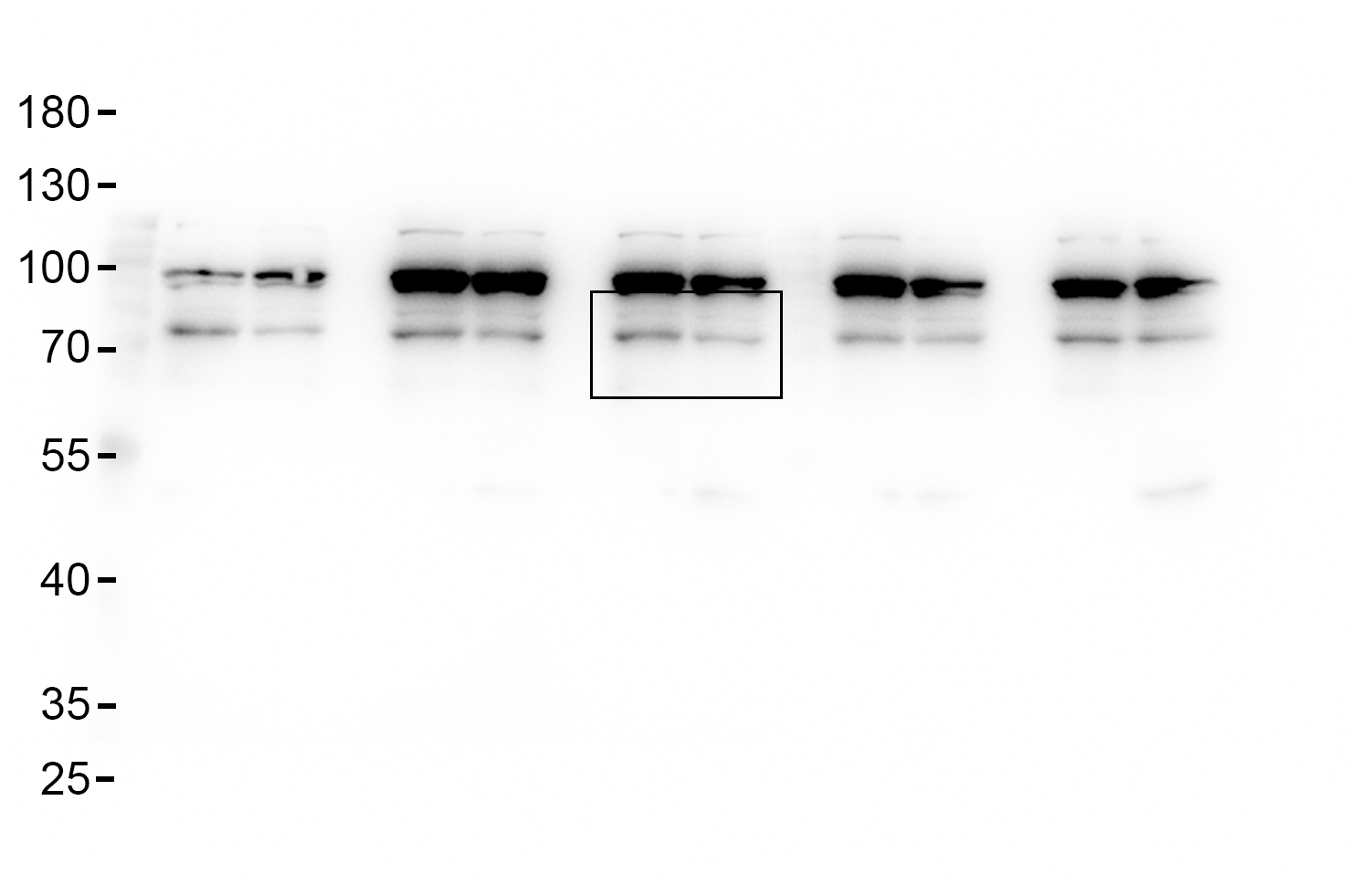

Supplement: Figure 4—figure supplement 2—source data 3. [file elife-97373-fig4-figsupp2-data3.zip › Figure 4-figure supplement 2-source data 2/Figure 4-figure supplement 2C/TAK1.tif]

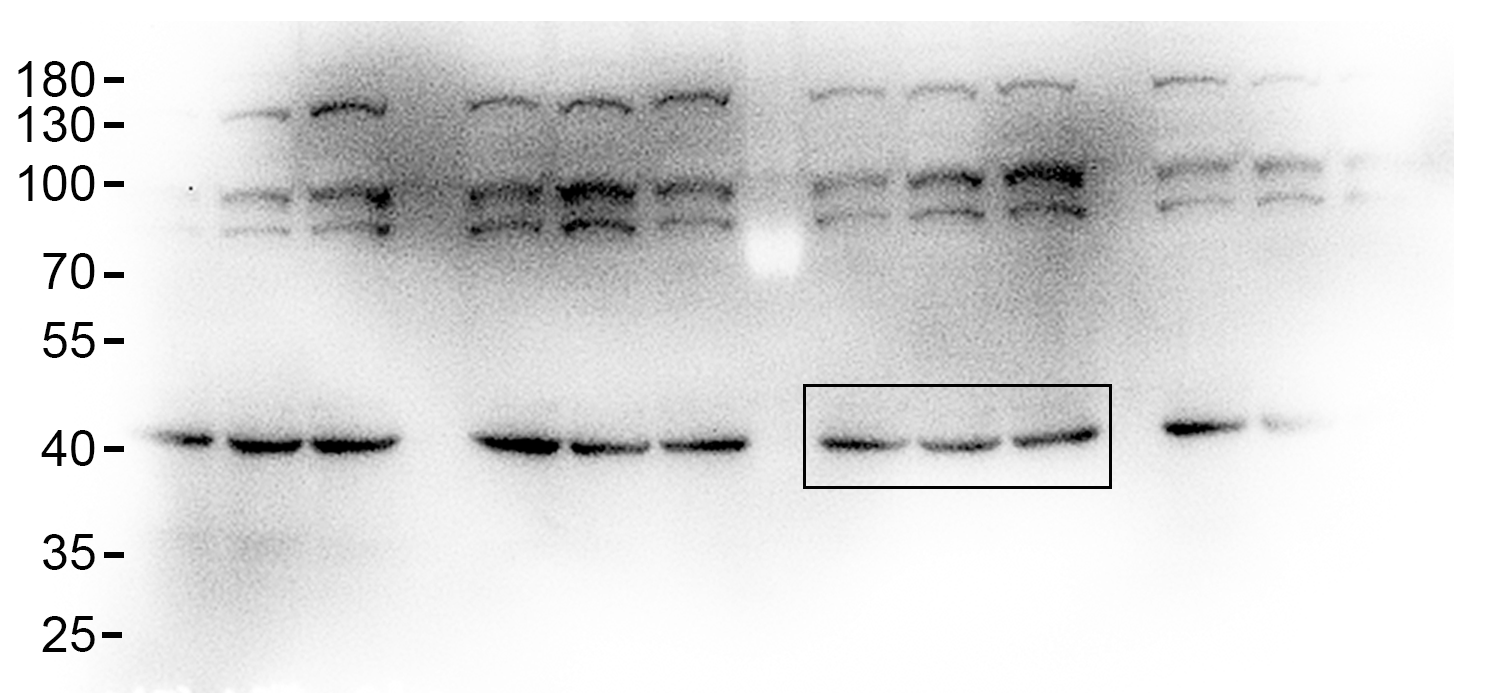

Supplement: Figure 5—source data 2. [file elife-97373-fig5-data2.zip › Figure 5-source data 2/Figure 5A/Actin.tif]

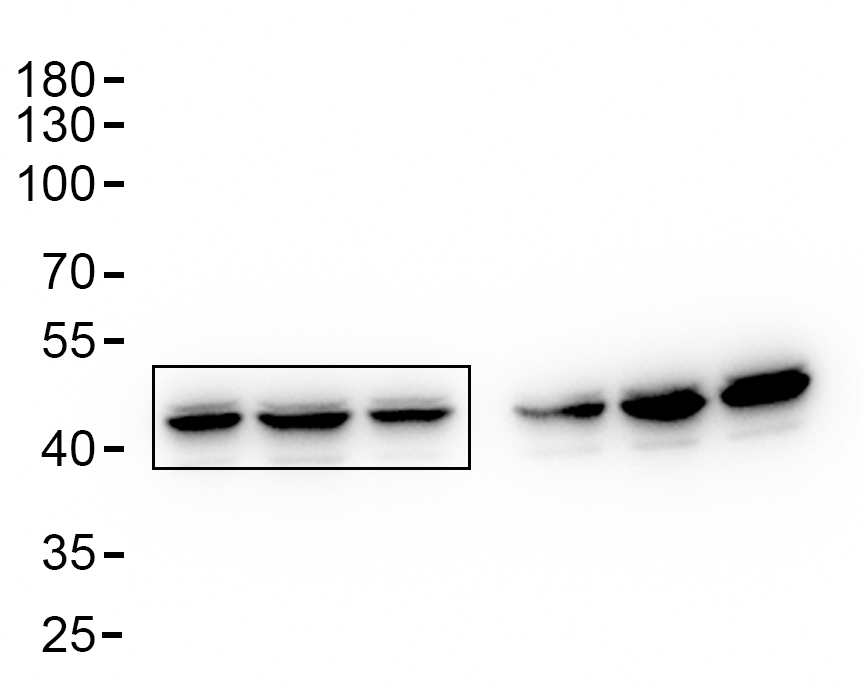

Supplement: Figure 5—source data 2. [file elife-97373-fig5-data2.zip › Figure 5-source data 2/Figure 5A/gsk-3b.tif]

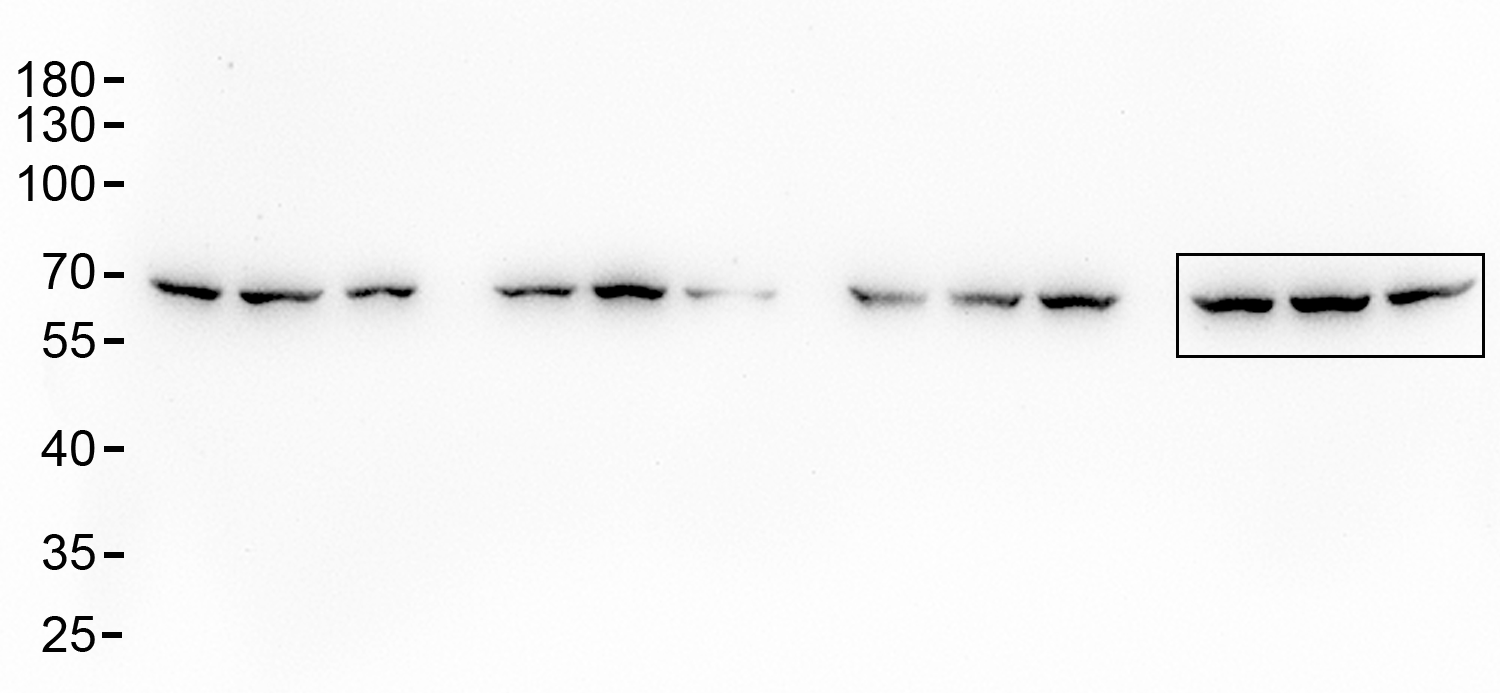

Supplement: Figure 5—source data 2. [file elife-97373-fig5-data2.zip › Figure 5-source data 2/Figure 5A/MMP2.tif]

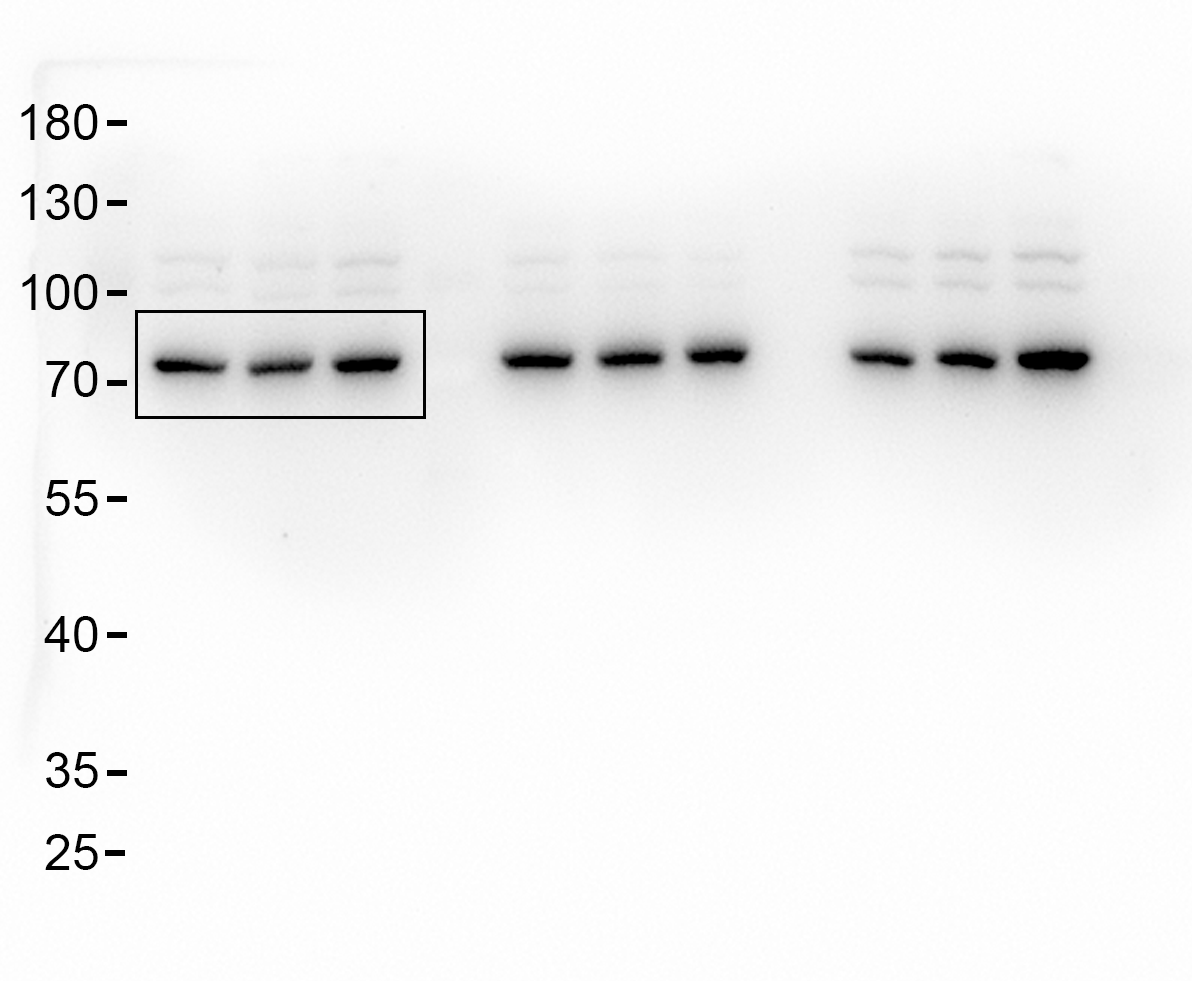

Supplement: Figure 5—source data 2. [file elife-97373-fig5-data2.zip › Figure 5-source data 2/Figure 5A/p-b-catenin.tif]

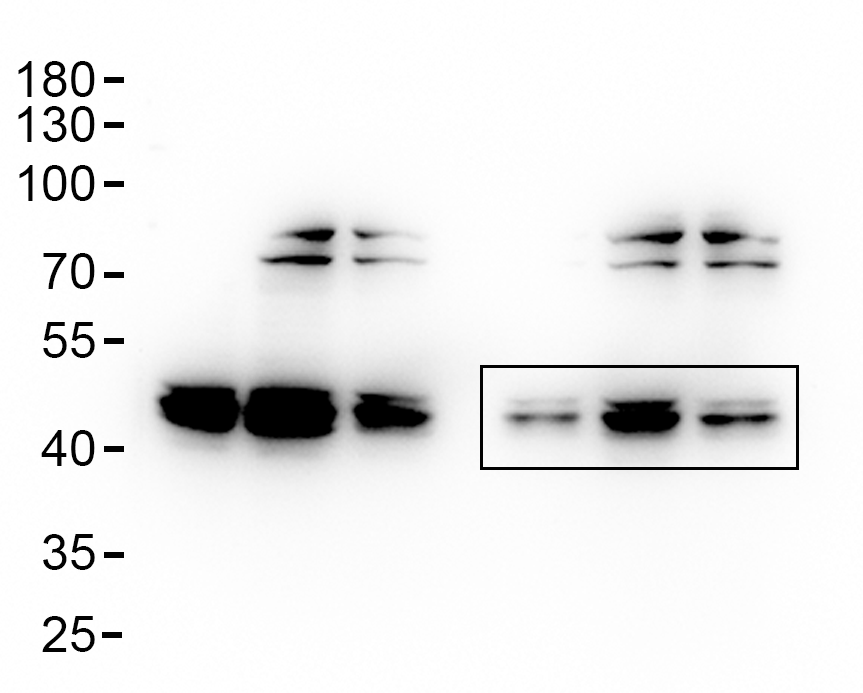

Supplement: Figure 5—source data 2. [file elife-97373-fig5-data2.zip › Figure 5-source data 2/Figure 5A/p-gsk-3b.tif]

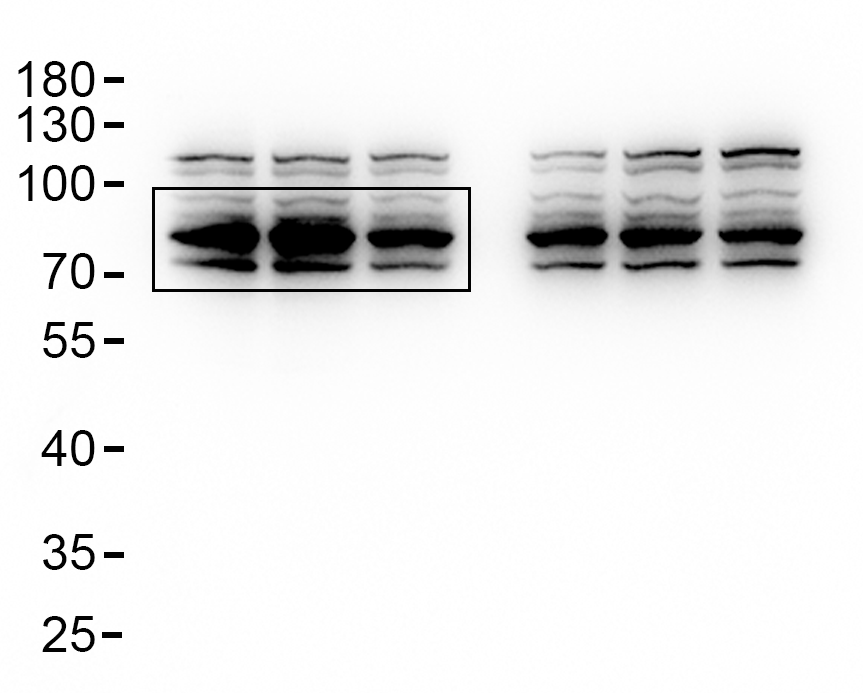

Supplement: Figure 5—source data 2. [file elife-97373-fig5-data2.zip › Figure 5-source data 2/Figure 5A/p-pkc.tif]

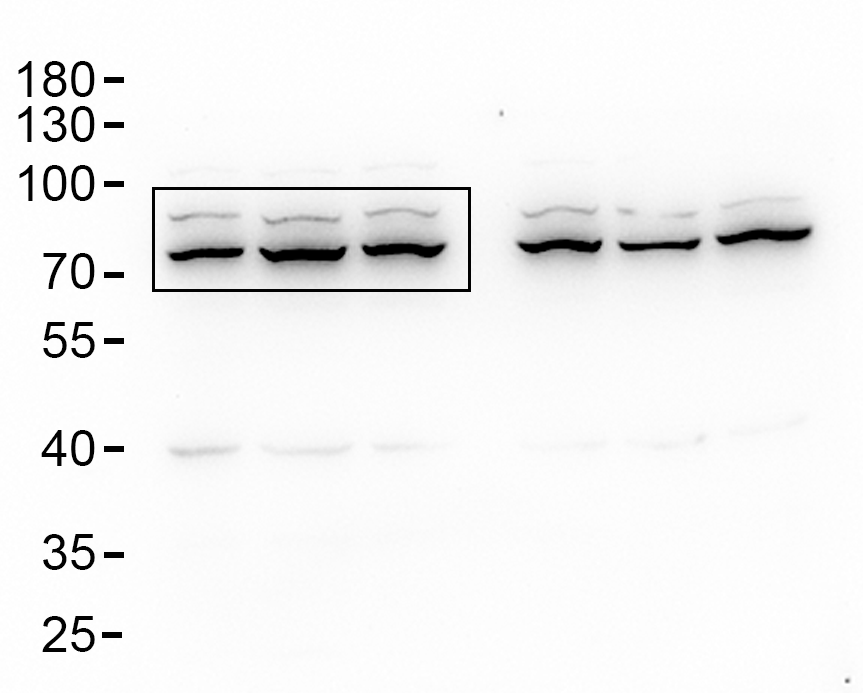

Supplement: Figure 5—source data 2. [file elife-97373-fig5-data2.zip › Figure 5-source data 2/Figure 5A/pkc.tif]

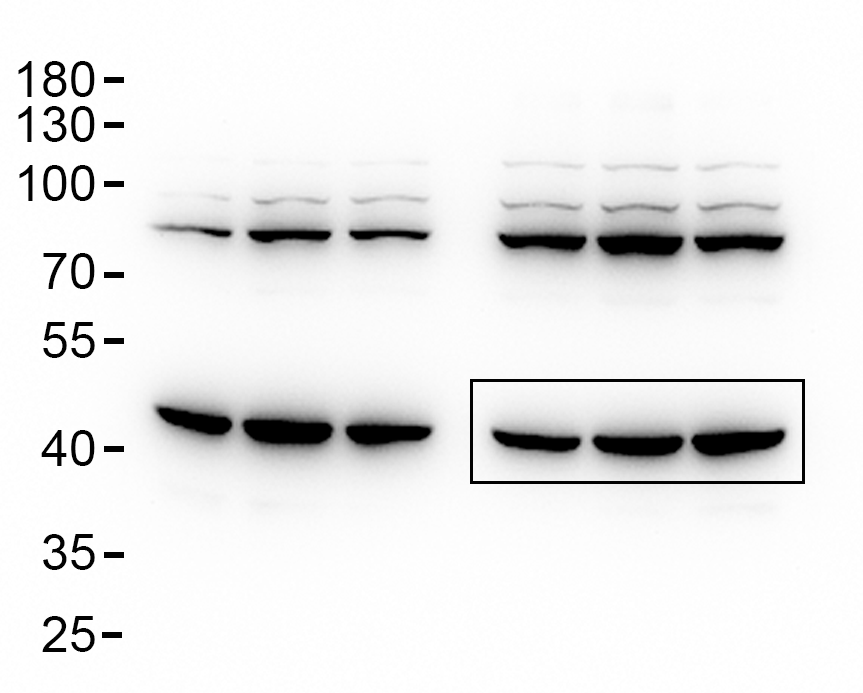

Supplement: Figure 5—source data 2. [file elife-97373-fig5-data2.zip › Figure 5-source data 2/Figure 5B/Actin.tif]

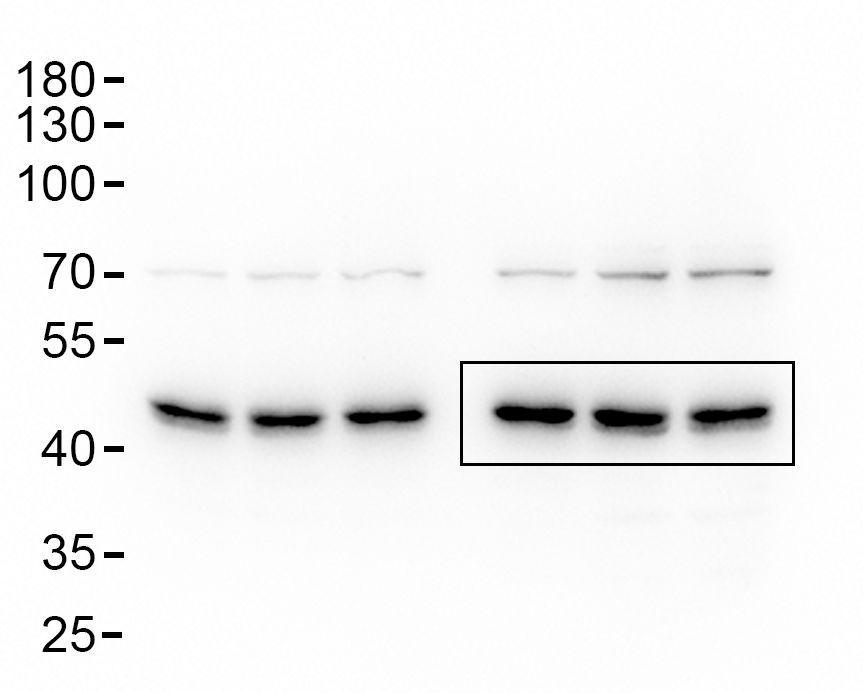

Supplement: Figure 5—source data 2. [file elife-97373-fig5-data2.zip › Figure 5-source data 2/Figure 5B/gsk-3b.tif]

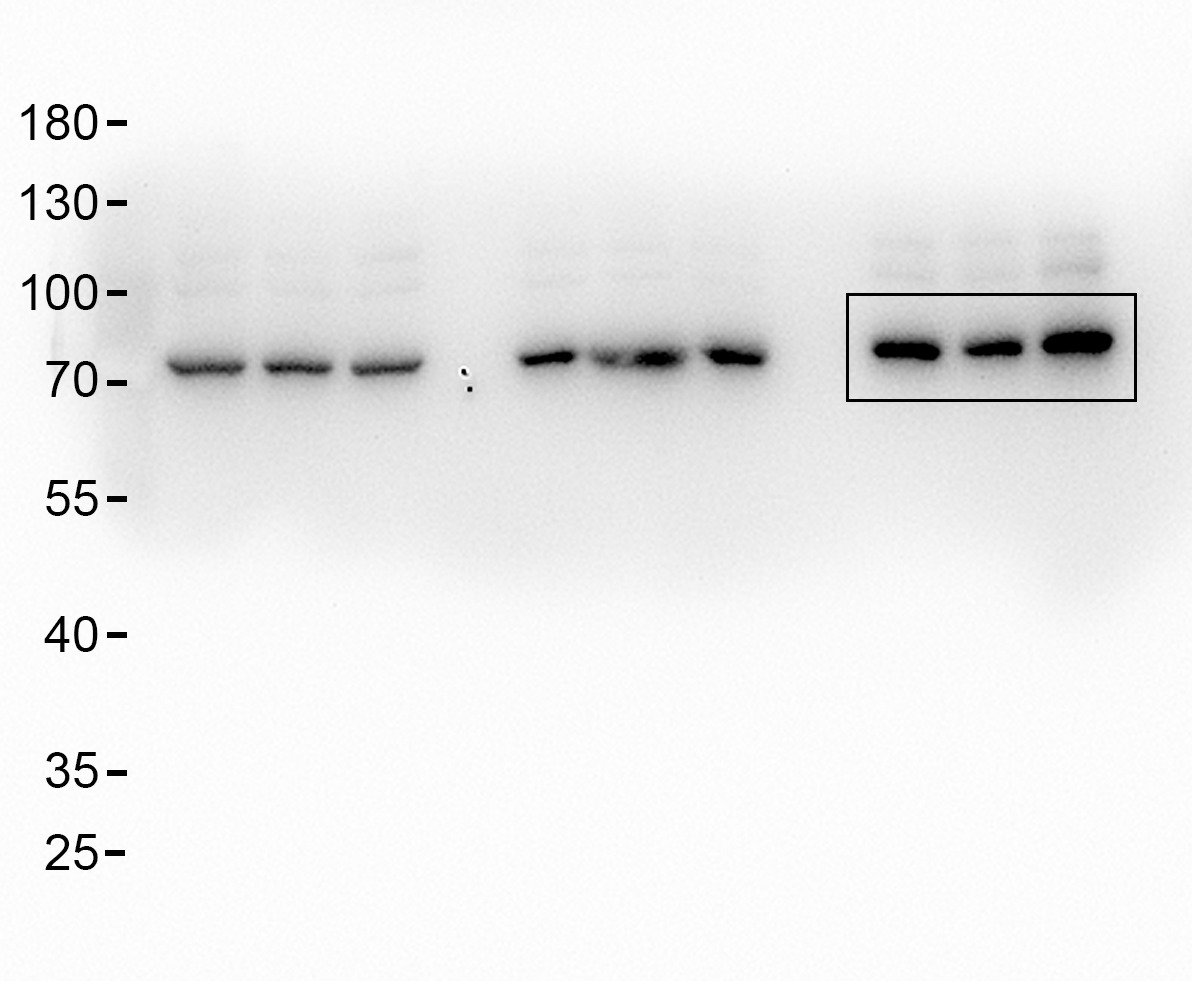

Supplement: Figure 5—source data 2. [file elife-97373-fig5-data2.zip › Figure 5-source data 2/Figure 5B/p-b-catenin.tif]

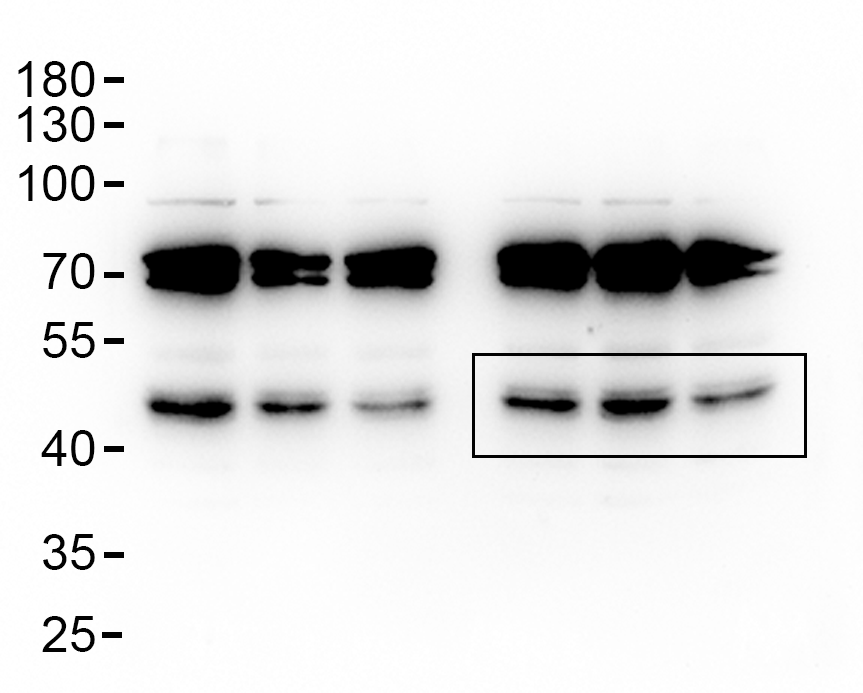

Supplement: Figure 5—source data 2. [file elife-97373-fig5-data2.zip › Figure 5-source data 2/Figure 5B/p-gsk-3b.tif]

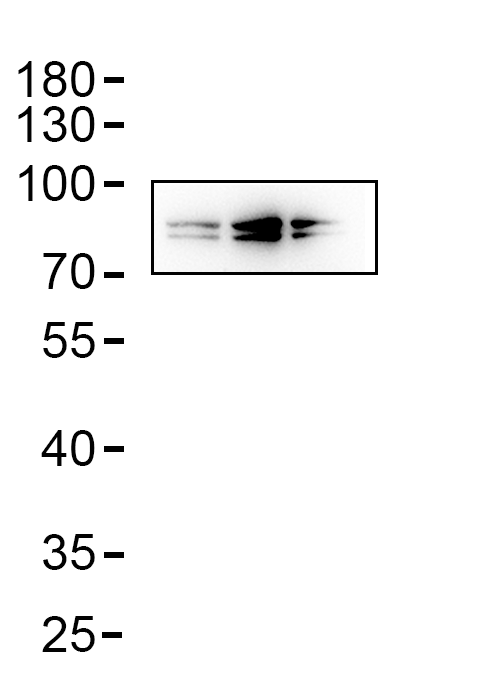

Supplement: Figure 5—source data 2. [file elife-97373-fig5-data2.zip › Figure 5-source data 2/Figure 5B/p-pkc.tif]

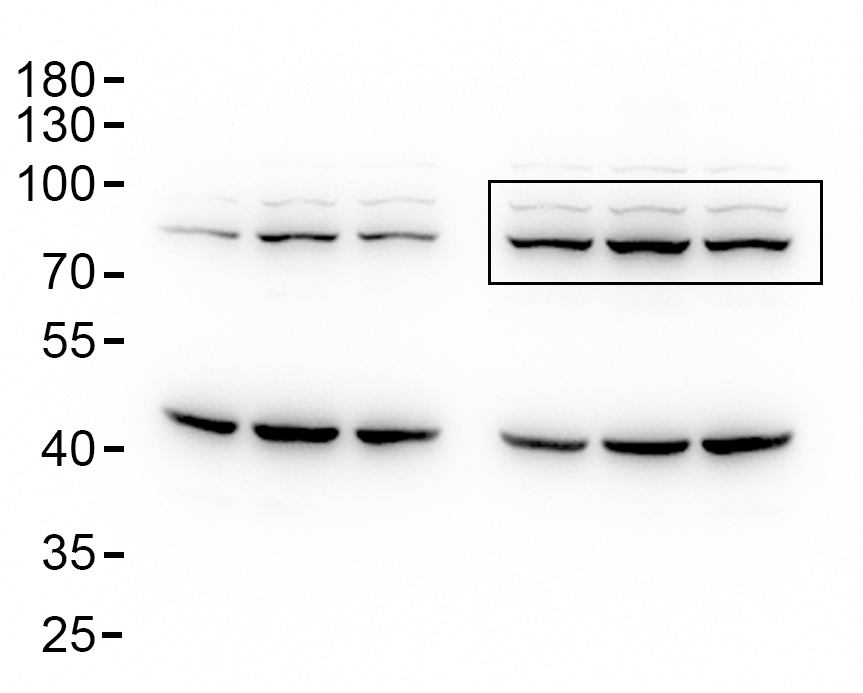

Supplement: Figure 5—source data 2. [file elife-97373-fig5-data2.zip › Figure 5-source data 2/Figure 5B/pkc.tif]

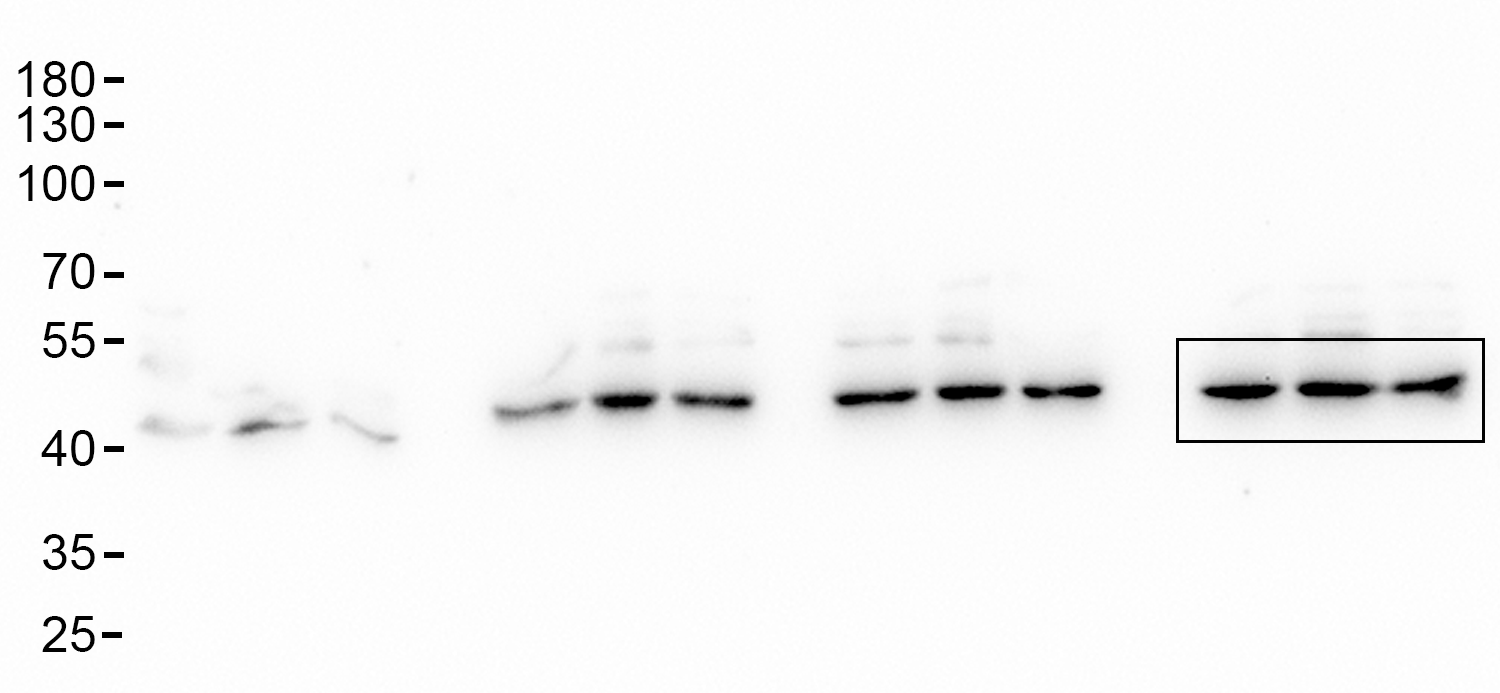

Supplement: Figure 5—source data 2. [file elife-97373-fig5-data2.zip › Figure 5-source data 2/Figure 5C/Actin.tif]

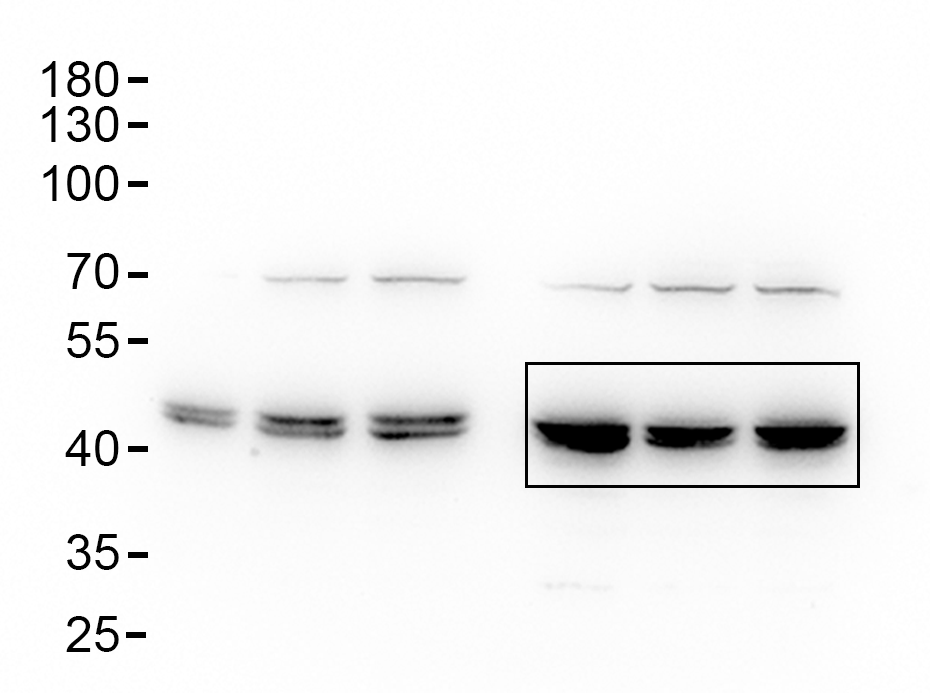

Supplement: Figure 5—source data 2. [file elife-97373-fig5-data2.zip › Figure 5-source data 2/Figure 5C/gsk-3b.tif]

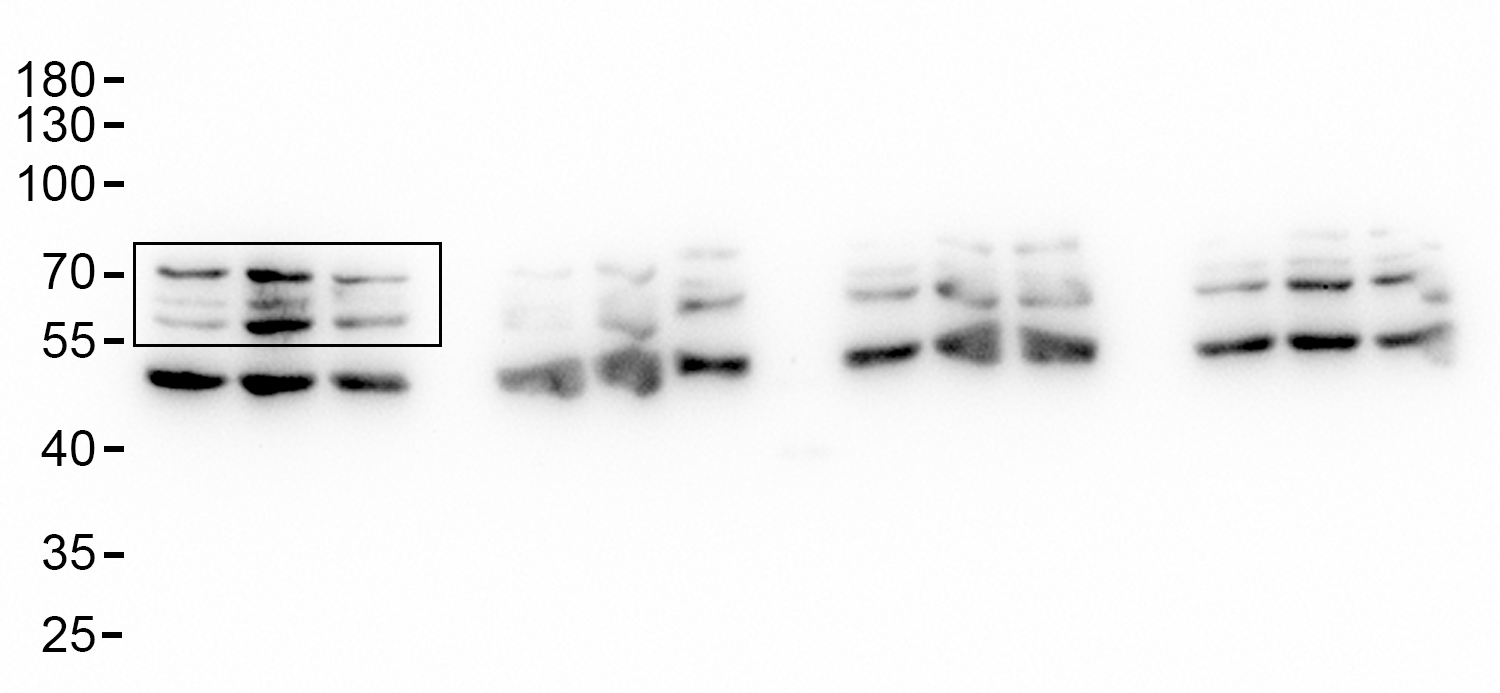

Supplement: Figure 5—source data 2. [file elife-97373-fig5-data2.zip › Figure 5-source data 2/Figure 5C/MMP2.tif]

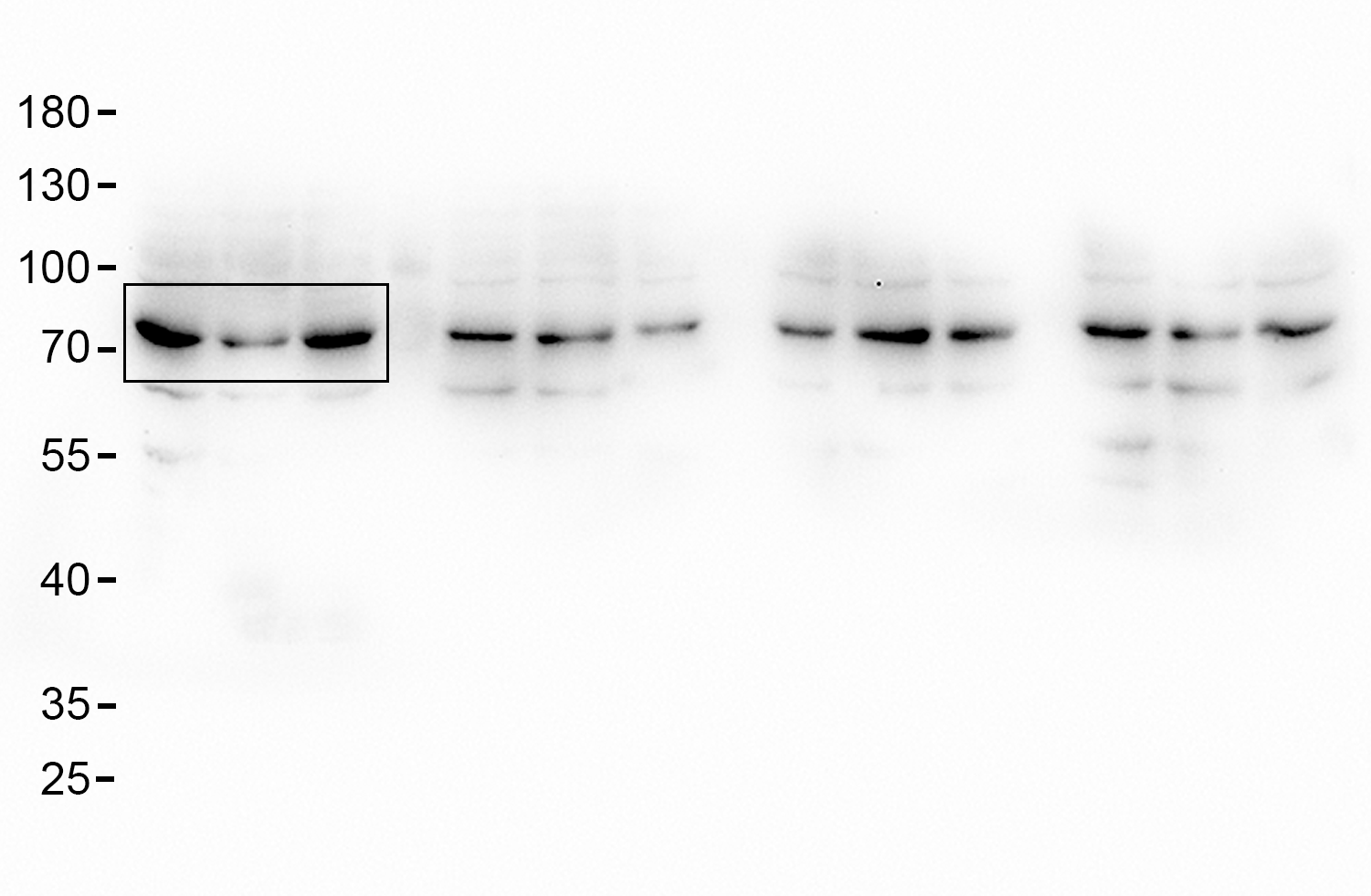

Supplement: Figure 5—source data 2. [file elife-97373-fig5-data2.zip › Figure 5-source data 2/Figure 5C/p-b-catenin.tif]

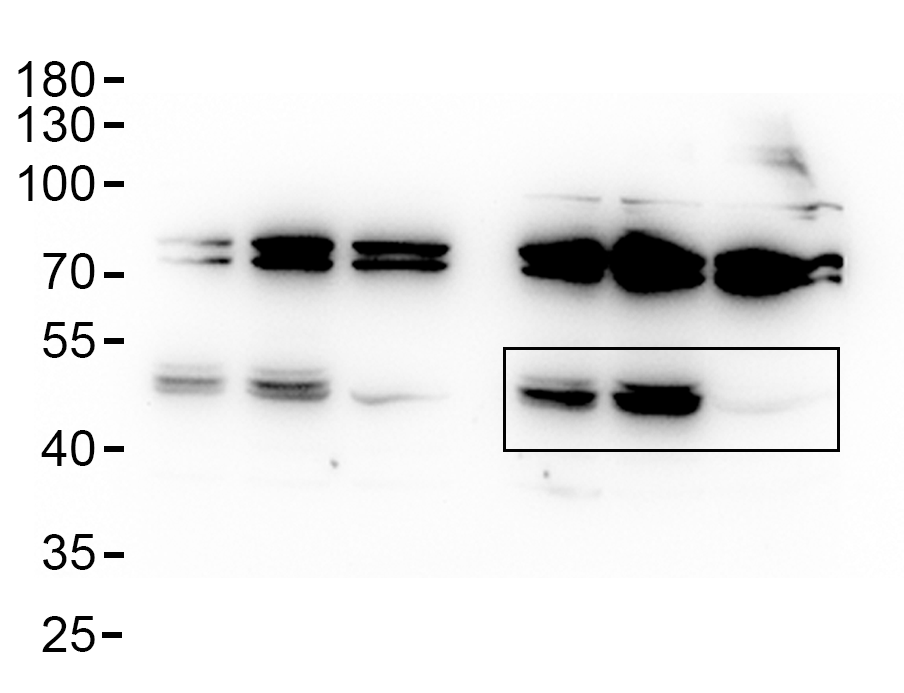

Supplement: Figure 5—source data 2. [file elife-97373-fig5-data2.zip › Figure 5-source data 2/Figure 5C/p-gsk-3b.tif]

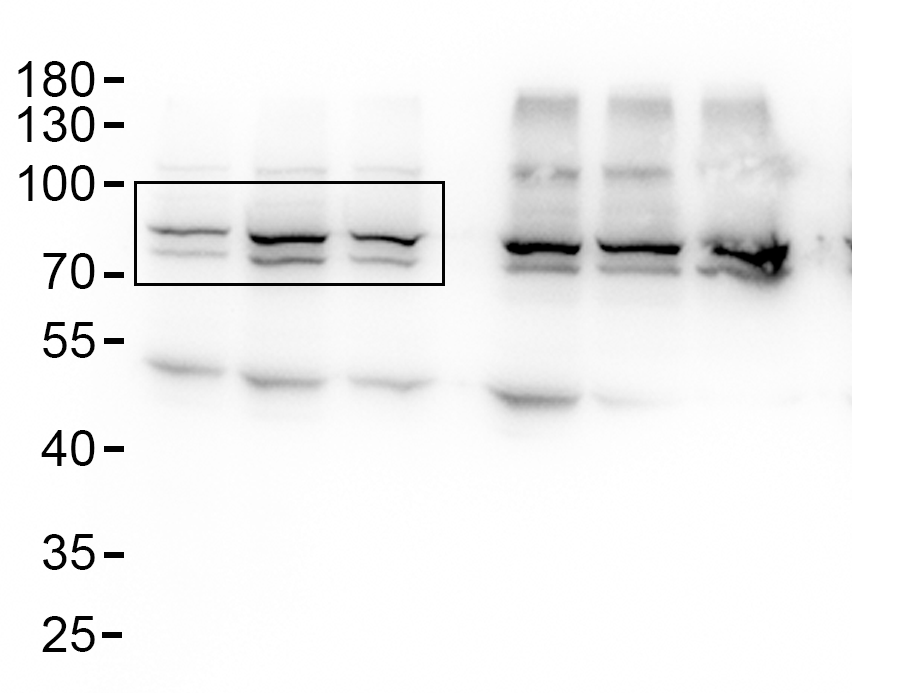

Supplement: Figure 5—source data 2. [file elife-97373-fig5-data2.zip › Figure 5-source data 2/Figure 5C/p-pkc.tif]

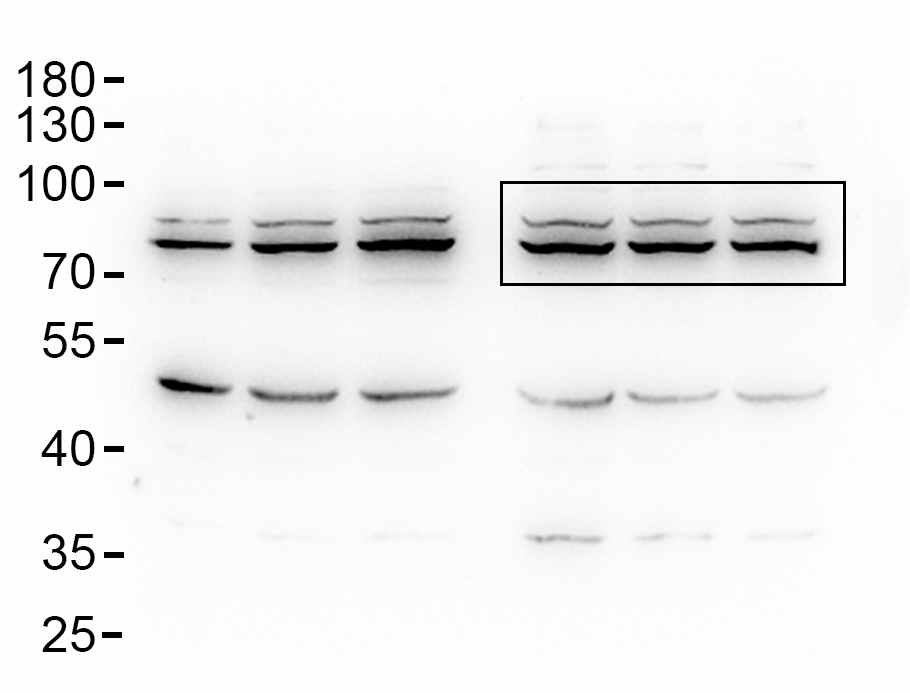

Supplement: Figure 5—source data 2. [file elife-97373-fig5-data2.zip › Figure 5-source data 2/Figure 5C/pkc.tif]

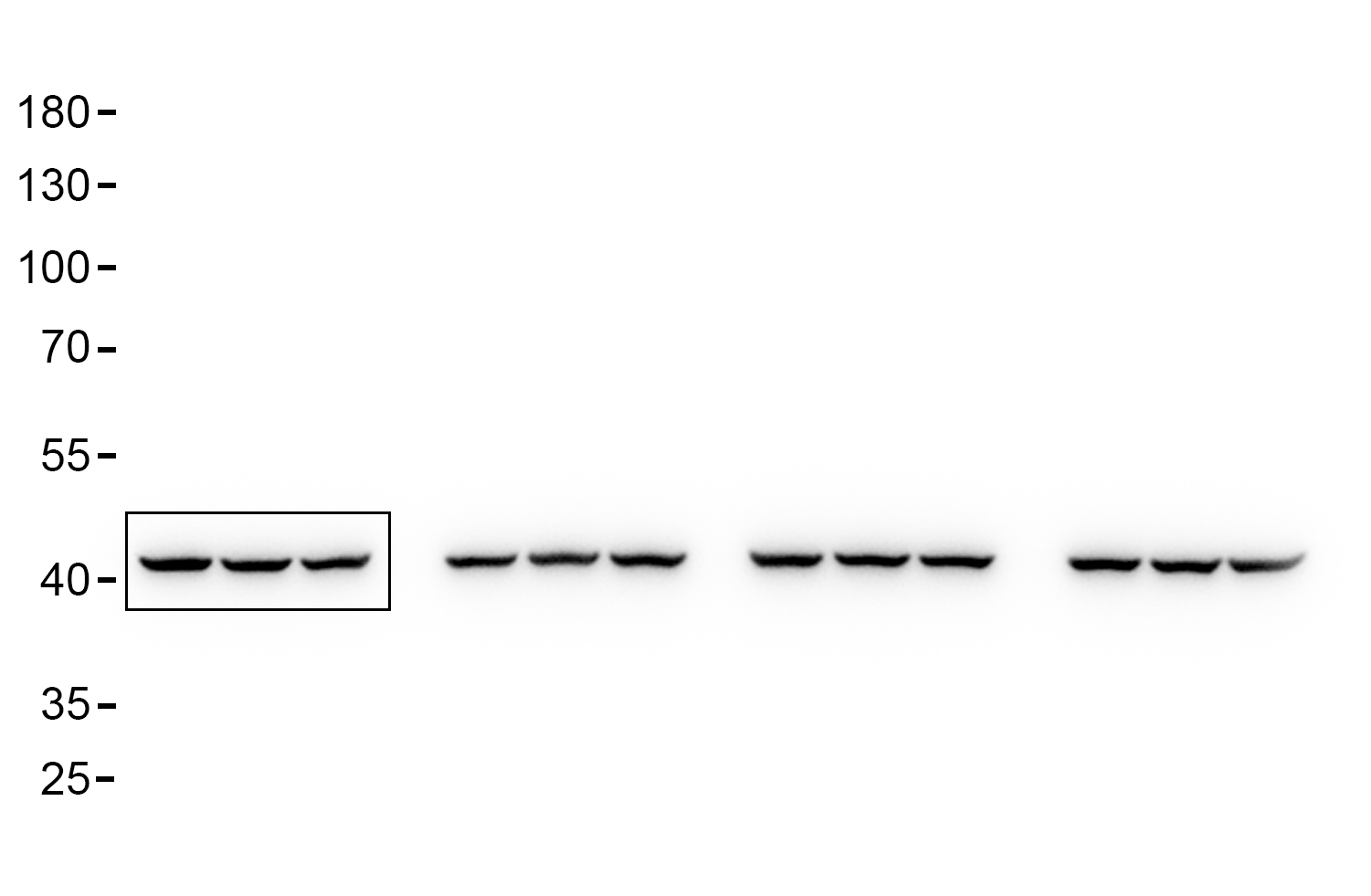

Supplement: Figure 5—source data 2. [file elife-97373-fig5-data2.zip › Figure 5-source data 2/Figure 5E/Actin.tif]

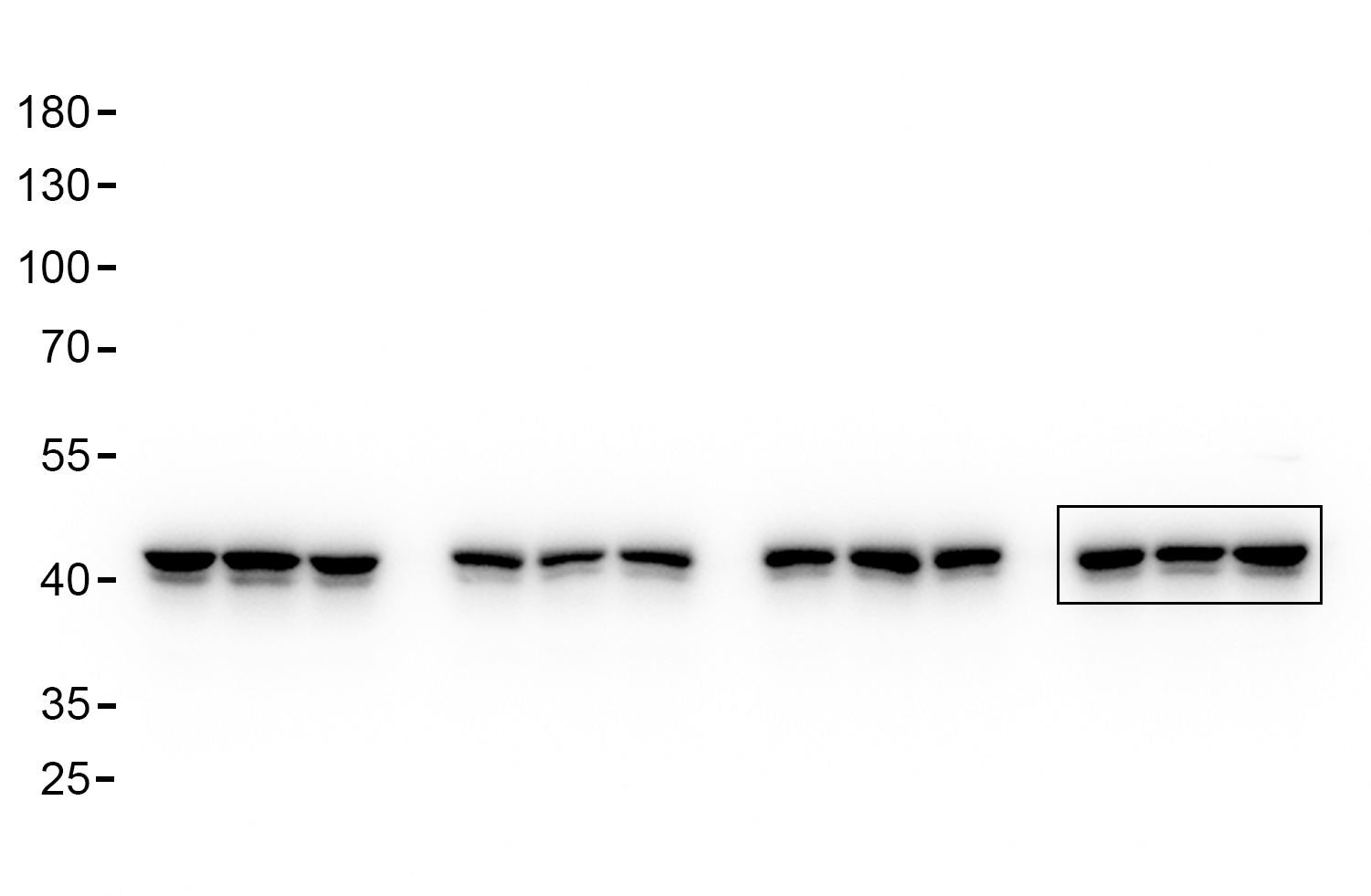

Supplement: Figure 5—source data 2. [file elife-97373-fig5-data2.zip › Figure 5-source data 2/Figure 5E/gsk-3b.tif]

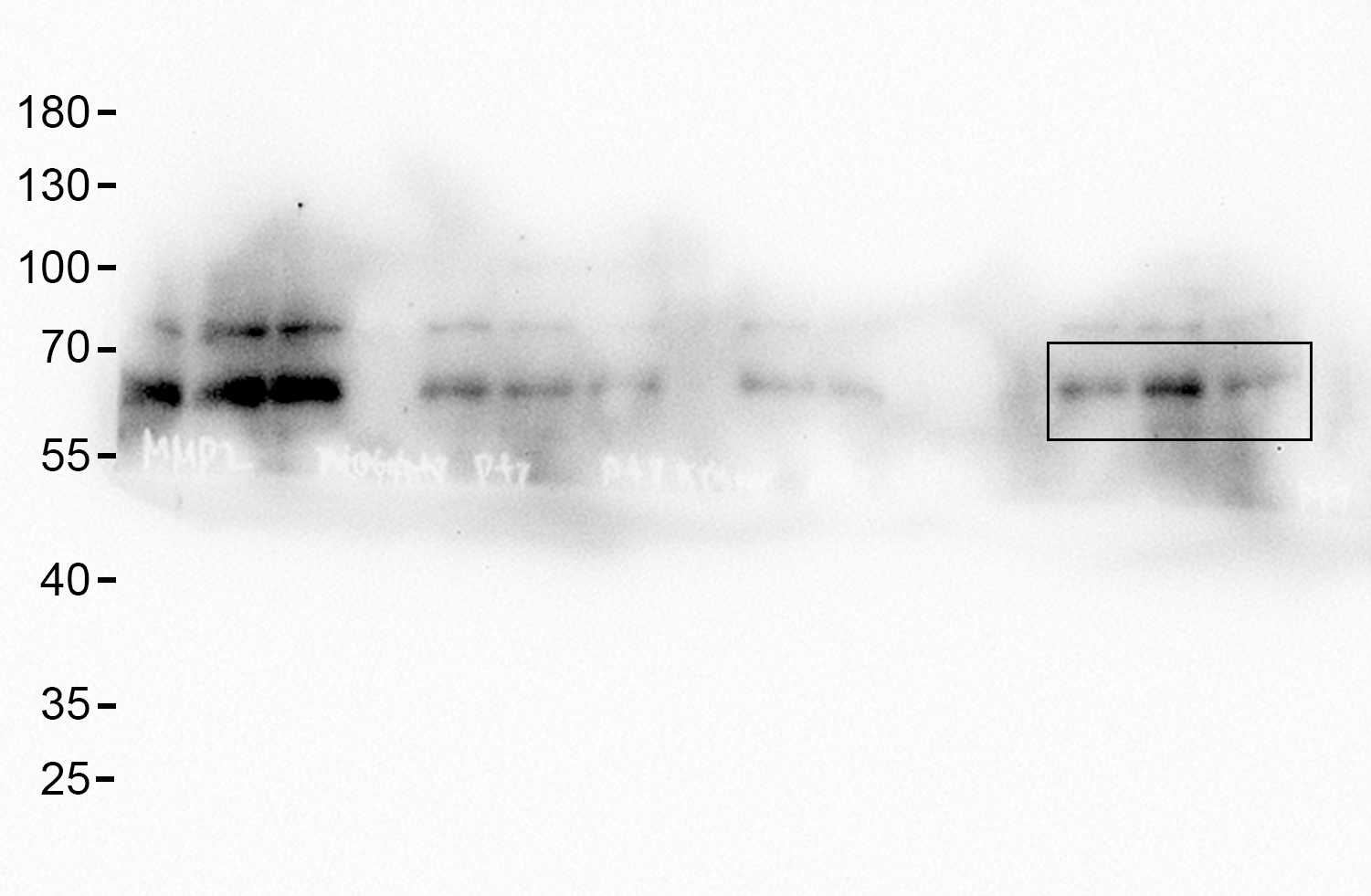

Supplement: Figure 5—source data 2. [file elife-97373-fig5-data2.zip › Figure 5-source data 2/Figure 5E/mmp2.tif]

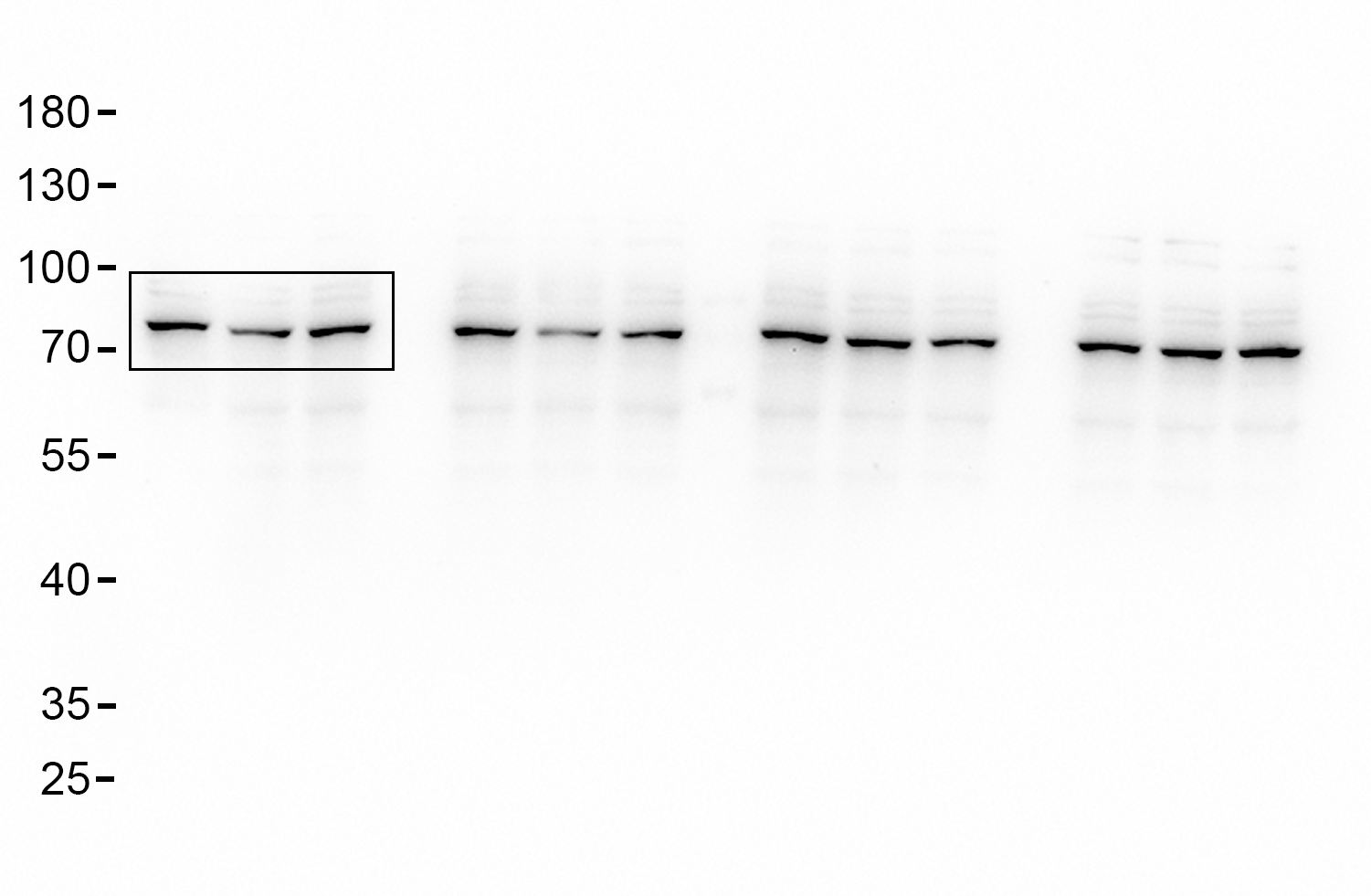

Supplement: Figure 5—source data 2. [file elife-97373-fig5-data2.zip › Figure 5-source data 2/Figure 5E/p-b-catenin.tif]

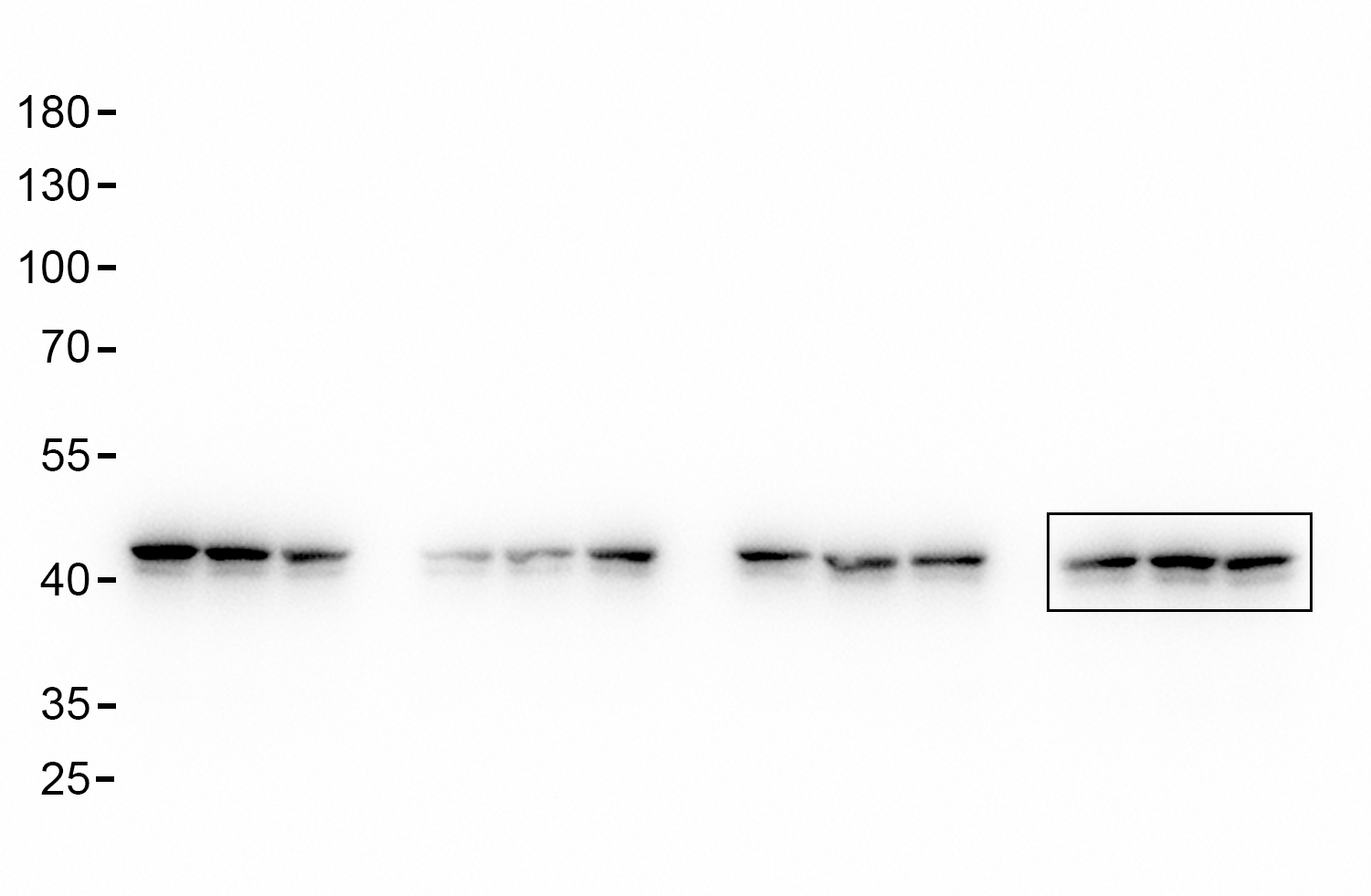

Supplement: Figure 5—source data 2. [file elife-97373-fig5-data2.zip › Figure 5-source data 2/Figure 5E/p-gsk-3b.tif]

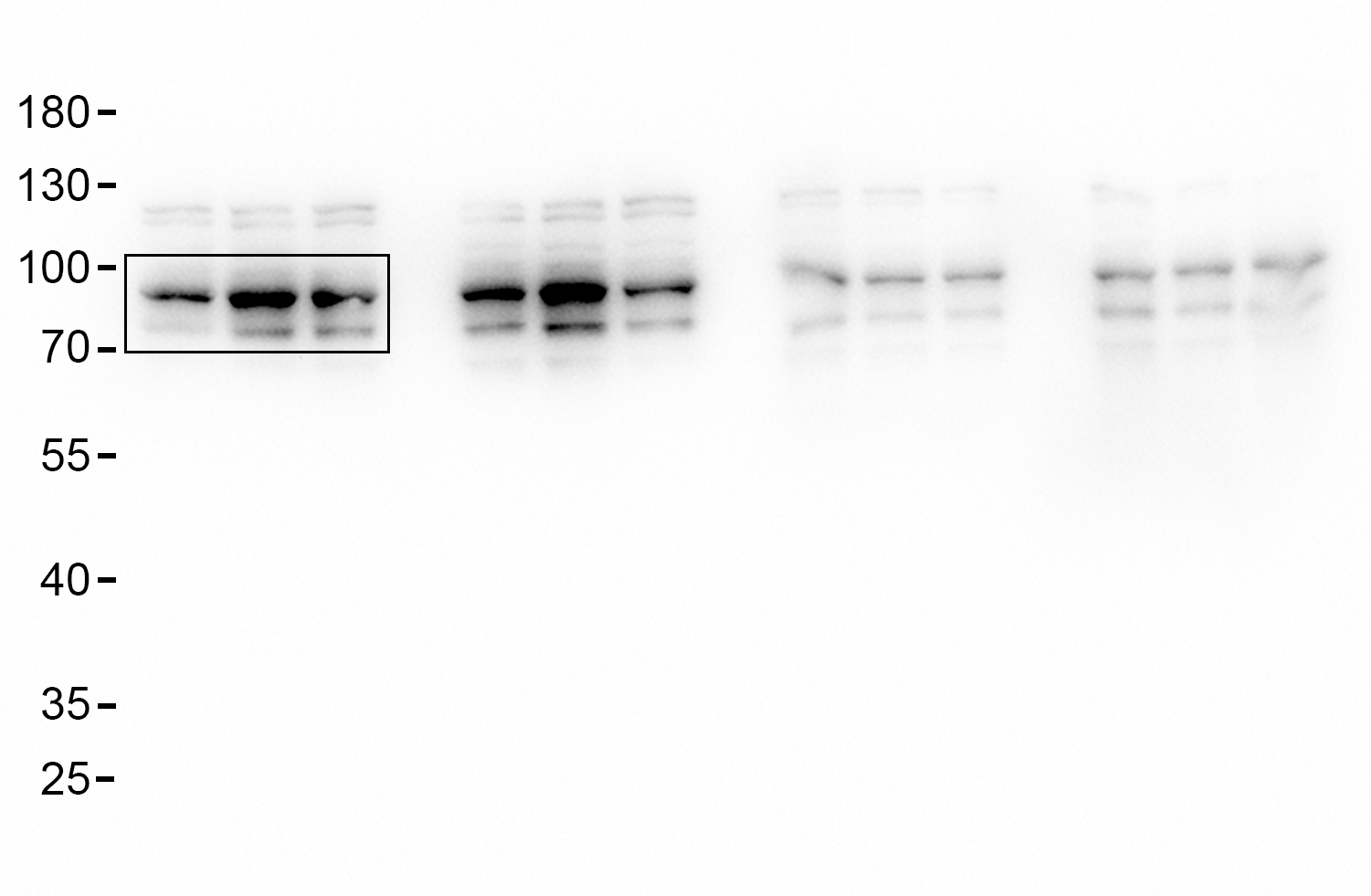

Supplement: Figure 5—source data 2. [file elife-97373-fig5-data2.zip › Figure 5-source data 2/Figure 5E/p-pkc.tif]

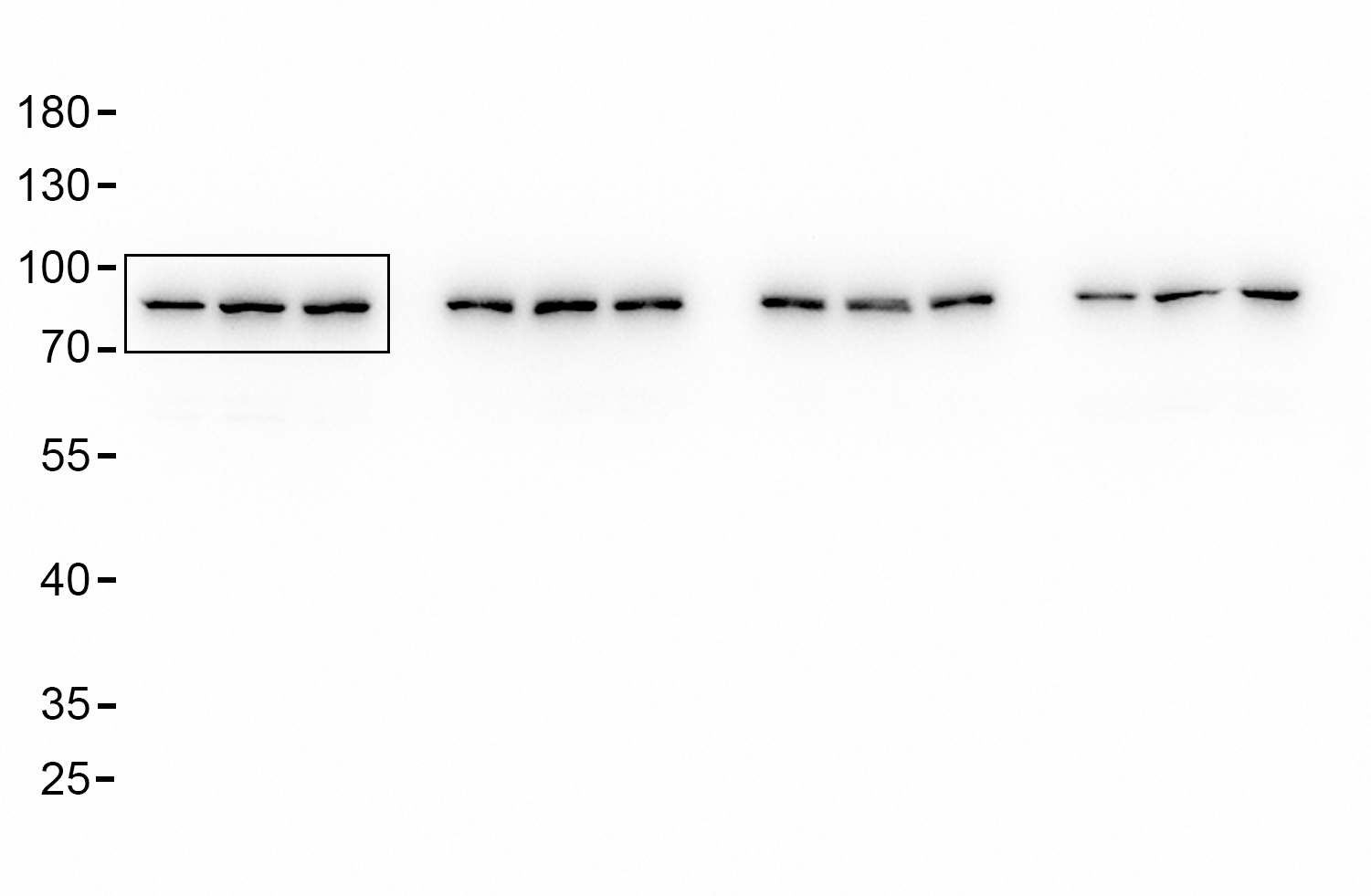

Supplement: Figure 5—source data 2. [file elife-97373-fig5-data2.zip › Figure 5-source data 2/Figure 5E/pkc.tif]

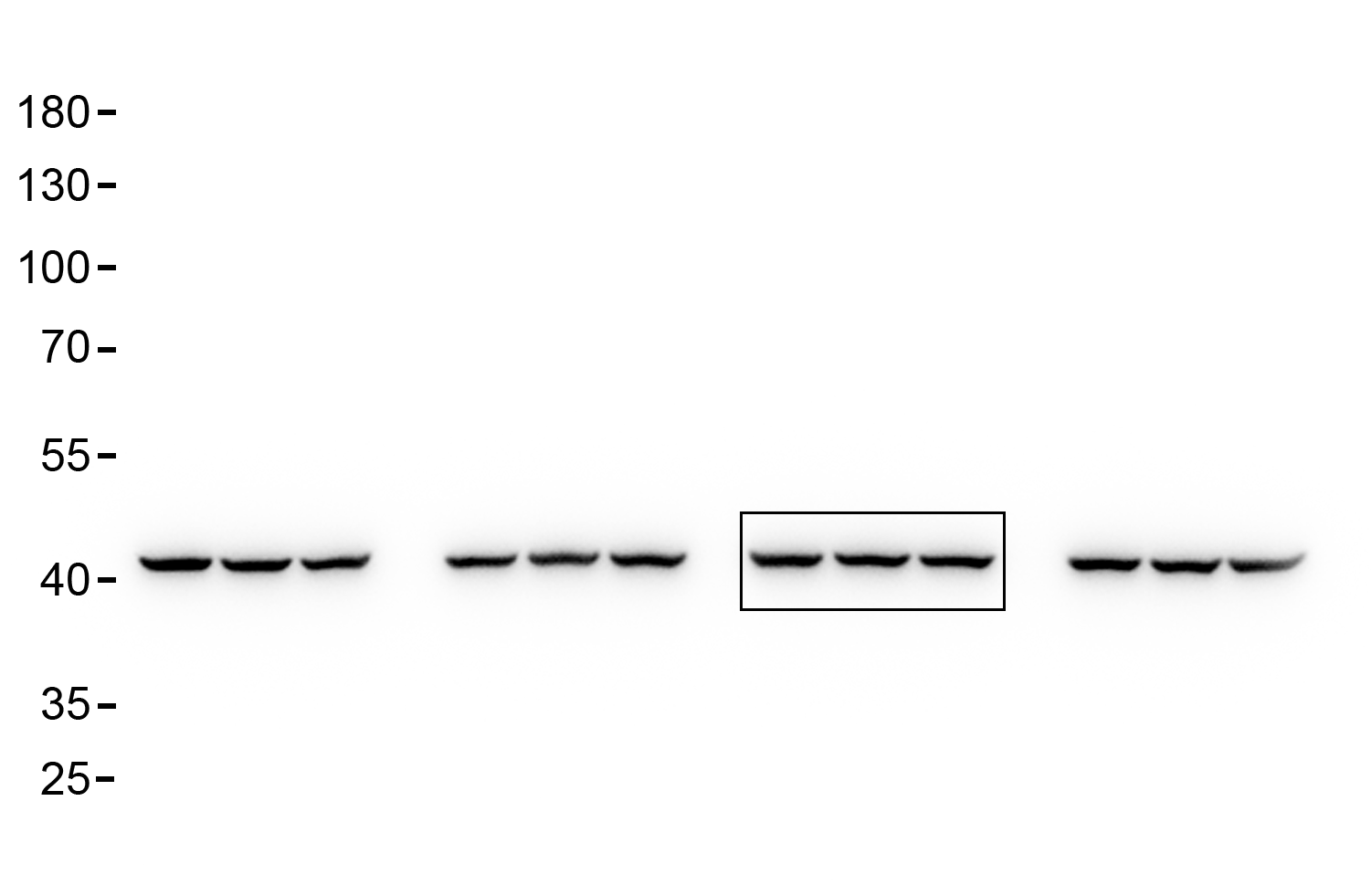

Supplement: Figure 5—source data 2. [file elife-97373-fig5-data2.zip › Figure 5-source data 2/Figure 5G/Actin.tif]

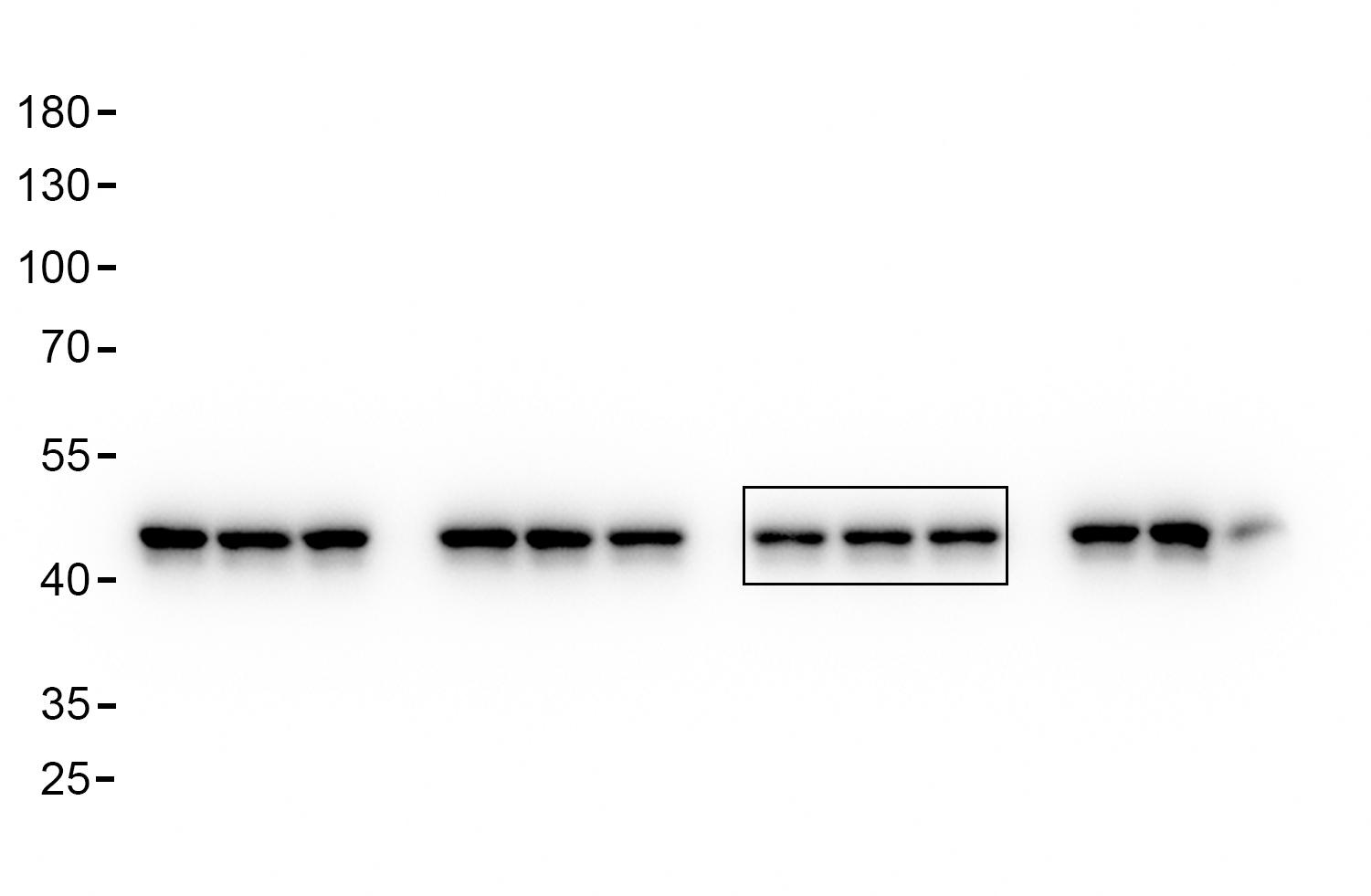

Supplement: Figure 5—source data 2. [file elife-97373-fig5-data2.zip › Figure 5-source data 2/Figure 5G/gsk-3b.tif]

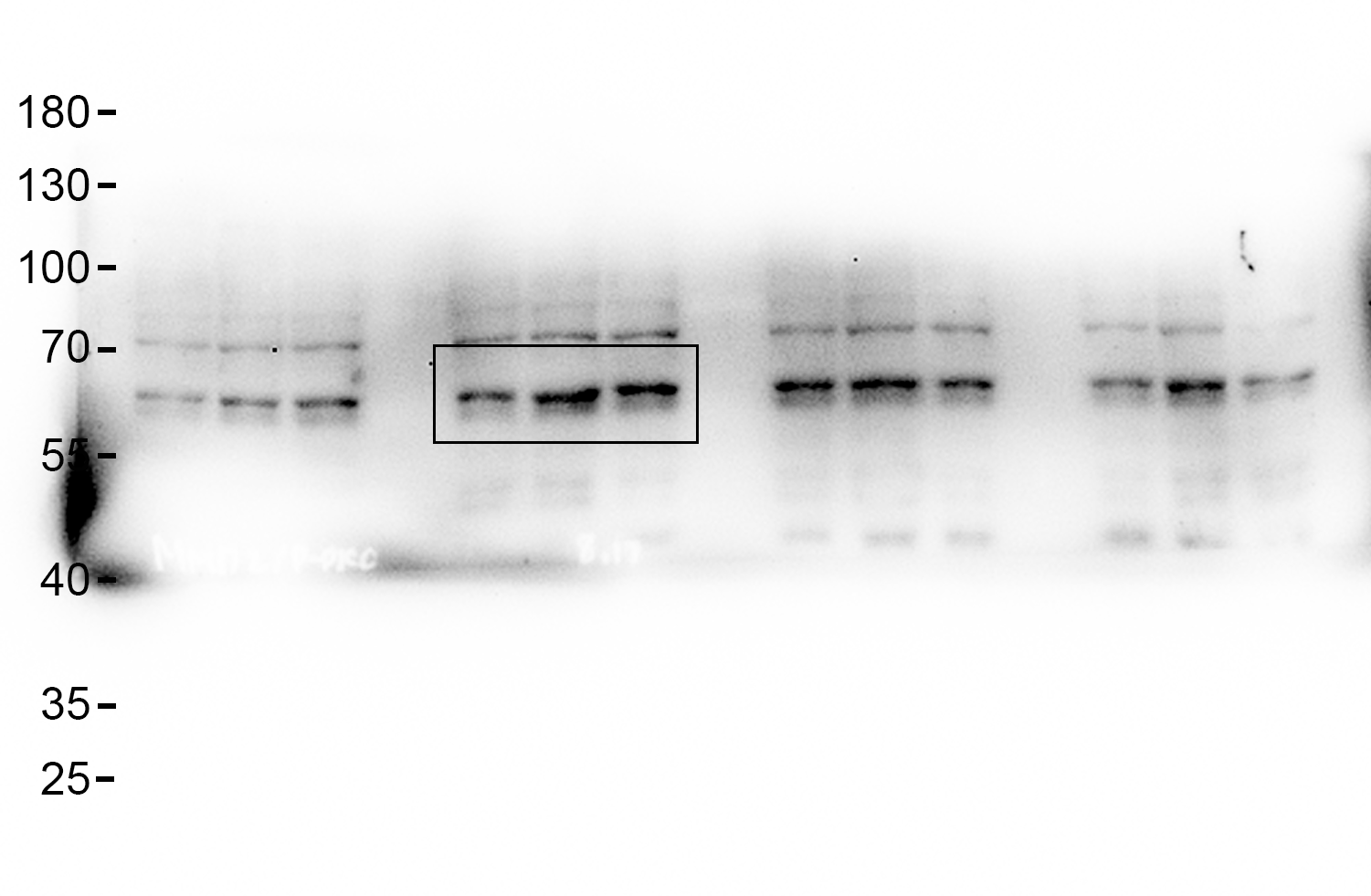

Supplement: Figure 5—source data 2. [file elife-97373-fig5-data2.zip › Figure 5-source data 2/Figure 5G/mmp2.tif]

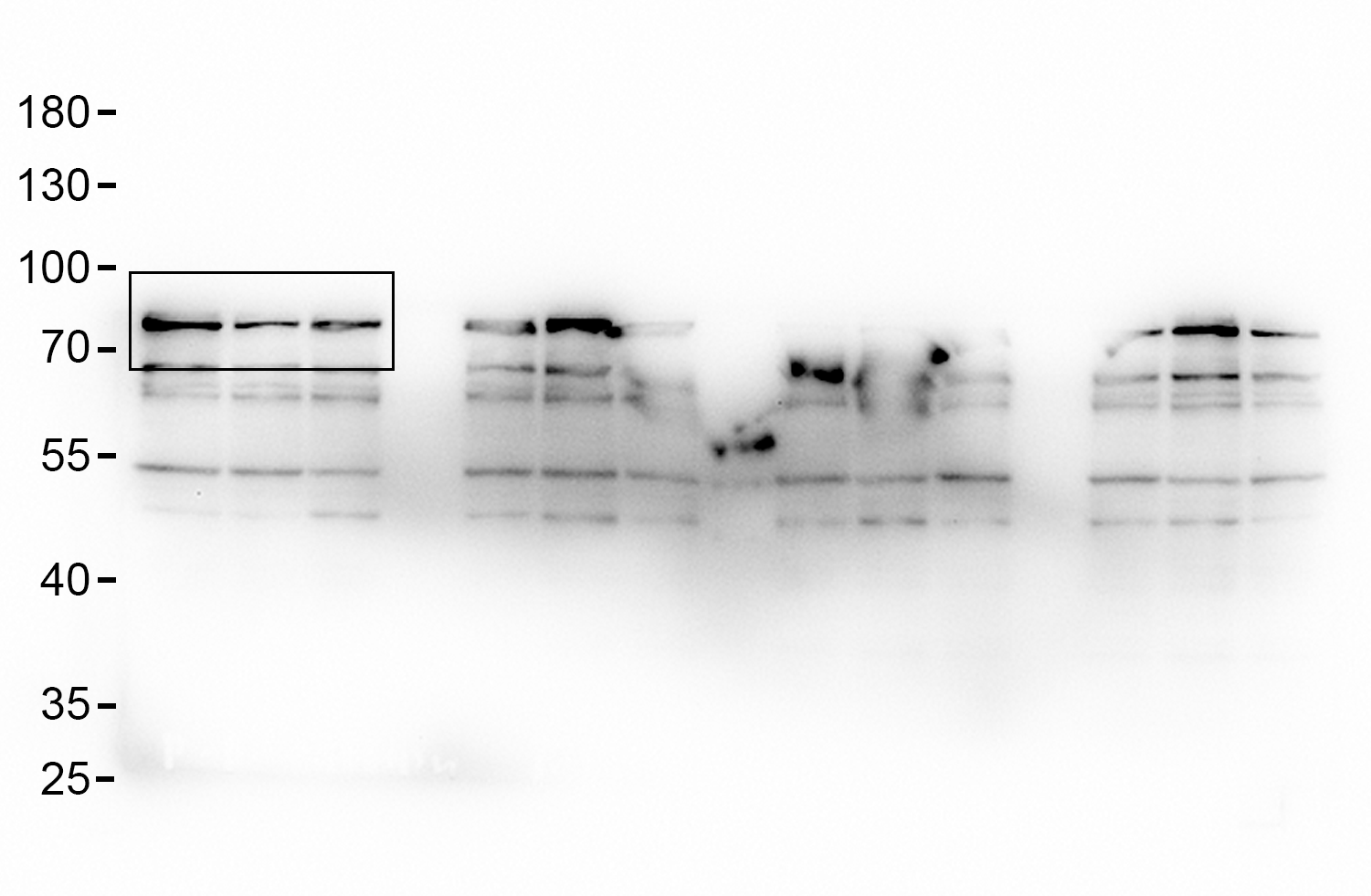

Supplement: Figure 5—source data 2. [file elife-97373-fig5-data2.zip › Figure 5-source data 2/Figure 5G/p-b-catenin.tif]

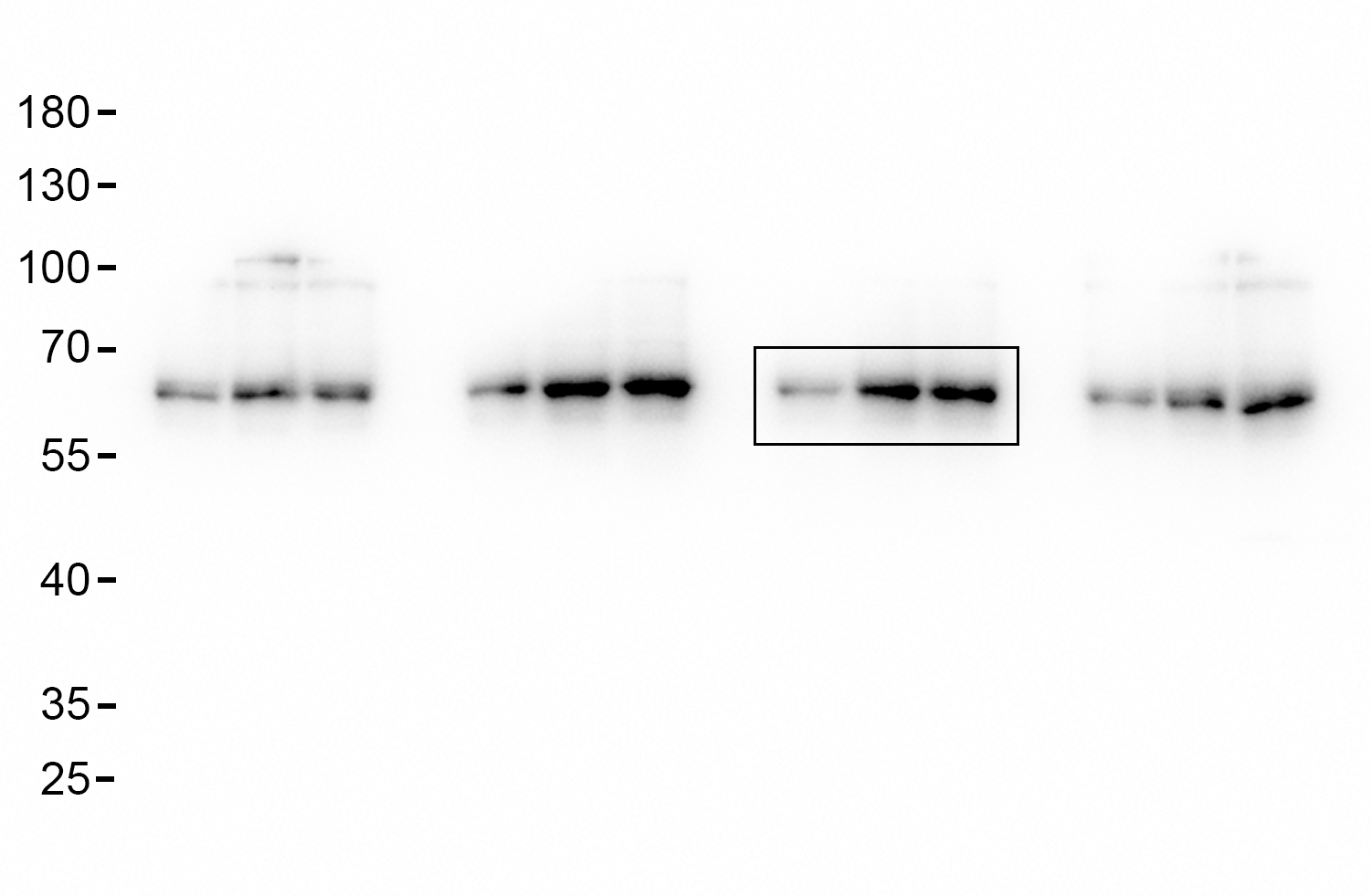

Supplement: Figure 5—source data 2. [file elife-97373-fig5-data2.zip › Figure 5-source data 2/Figure 5G/p-gsk-3b.tif]

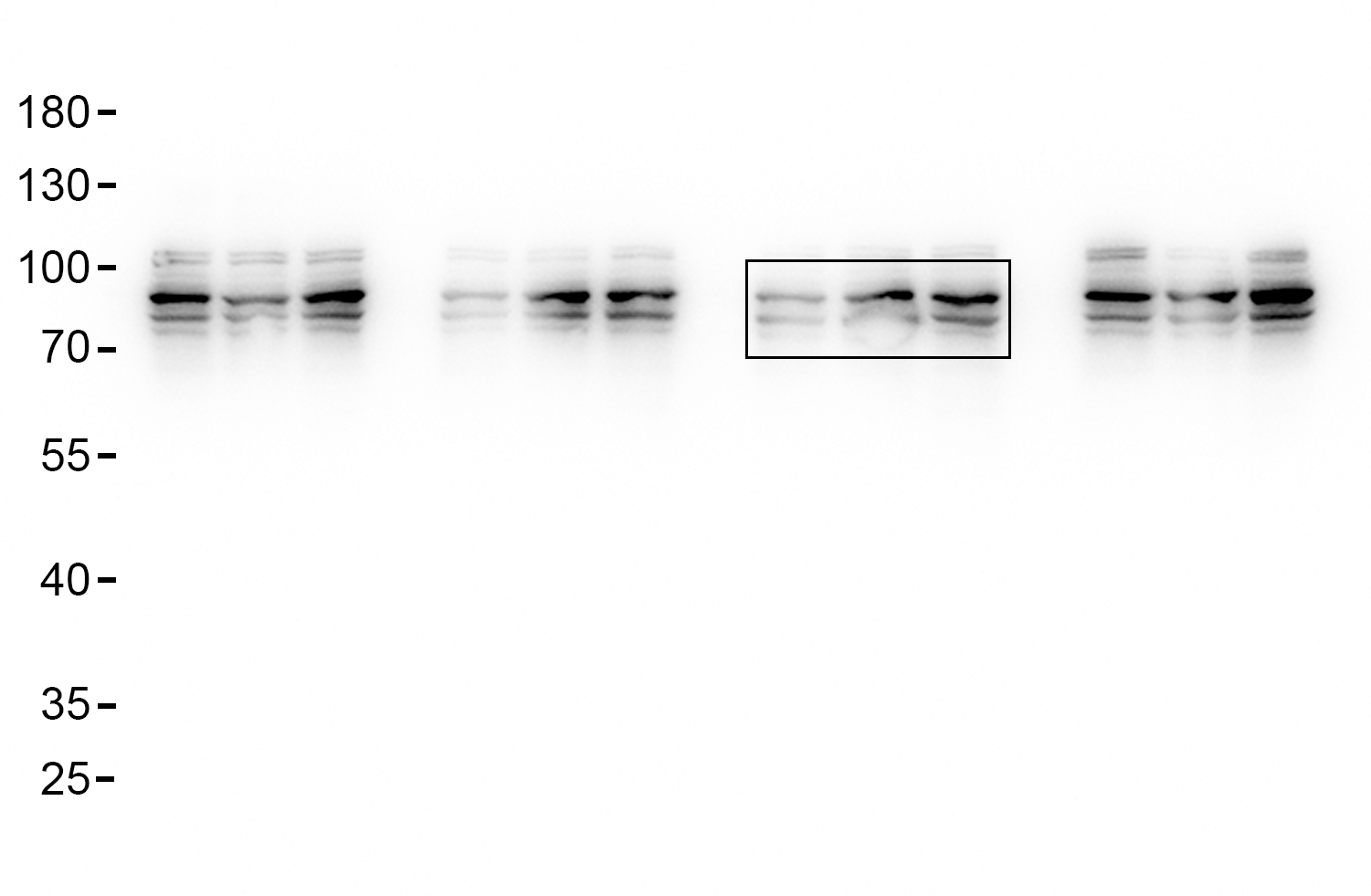

Supplement: Figure 5—source data 2. [file elife-97373-fig5-data2.zip › Figure 5-source data 2/Figure 5G/p-pkc.tif]

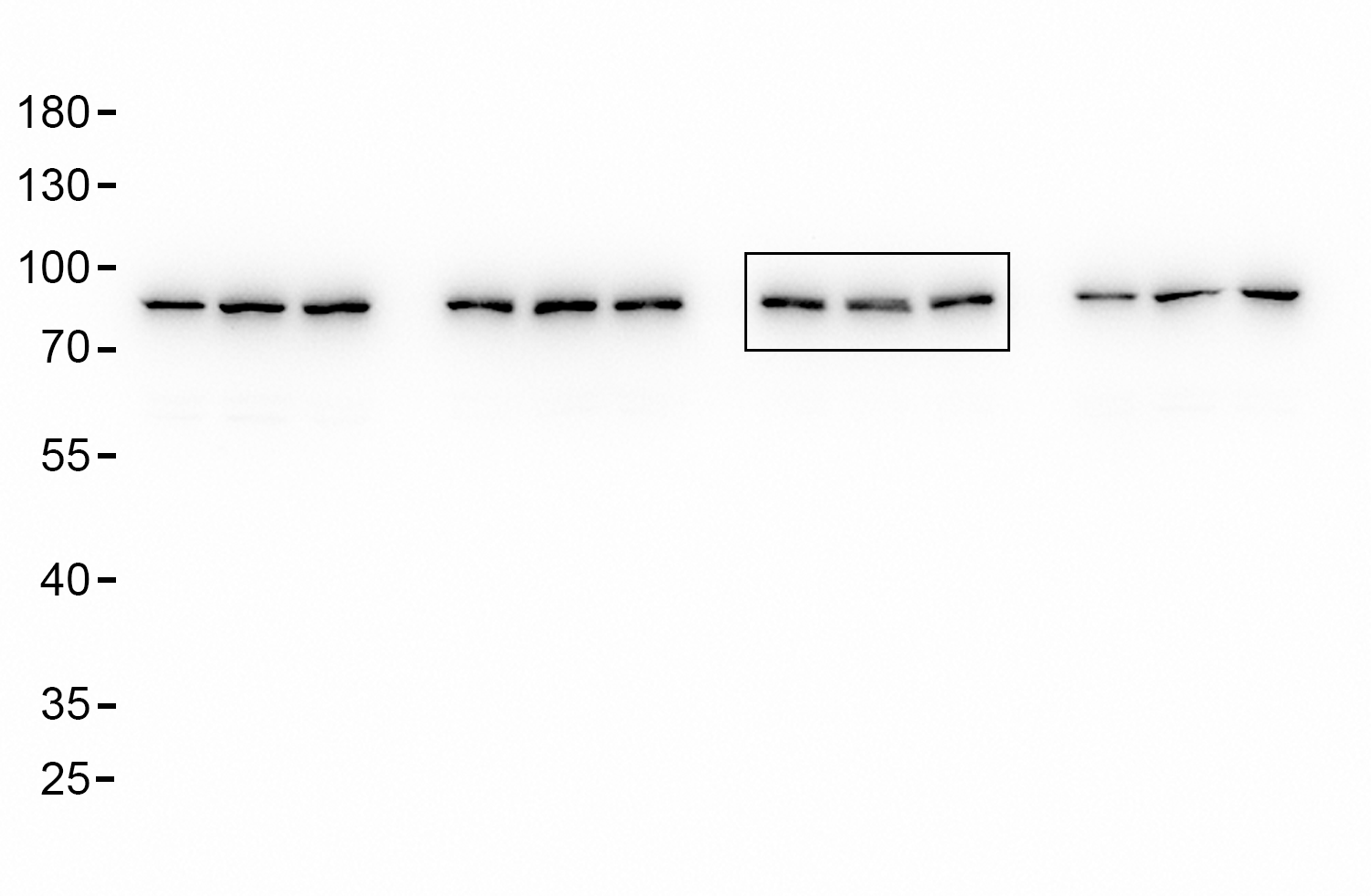

Supplement: Figure 5—source data 2. [file elife-97373-fig5-data2.zip › Figure 5-source data 2/Figure 5G/pkc.tif]

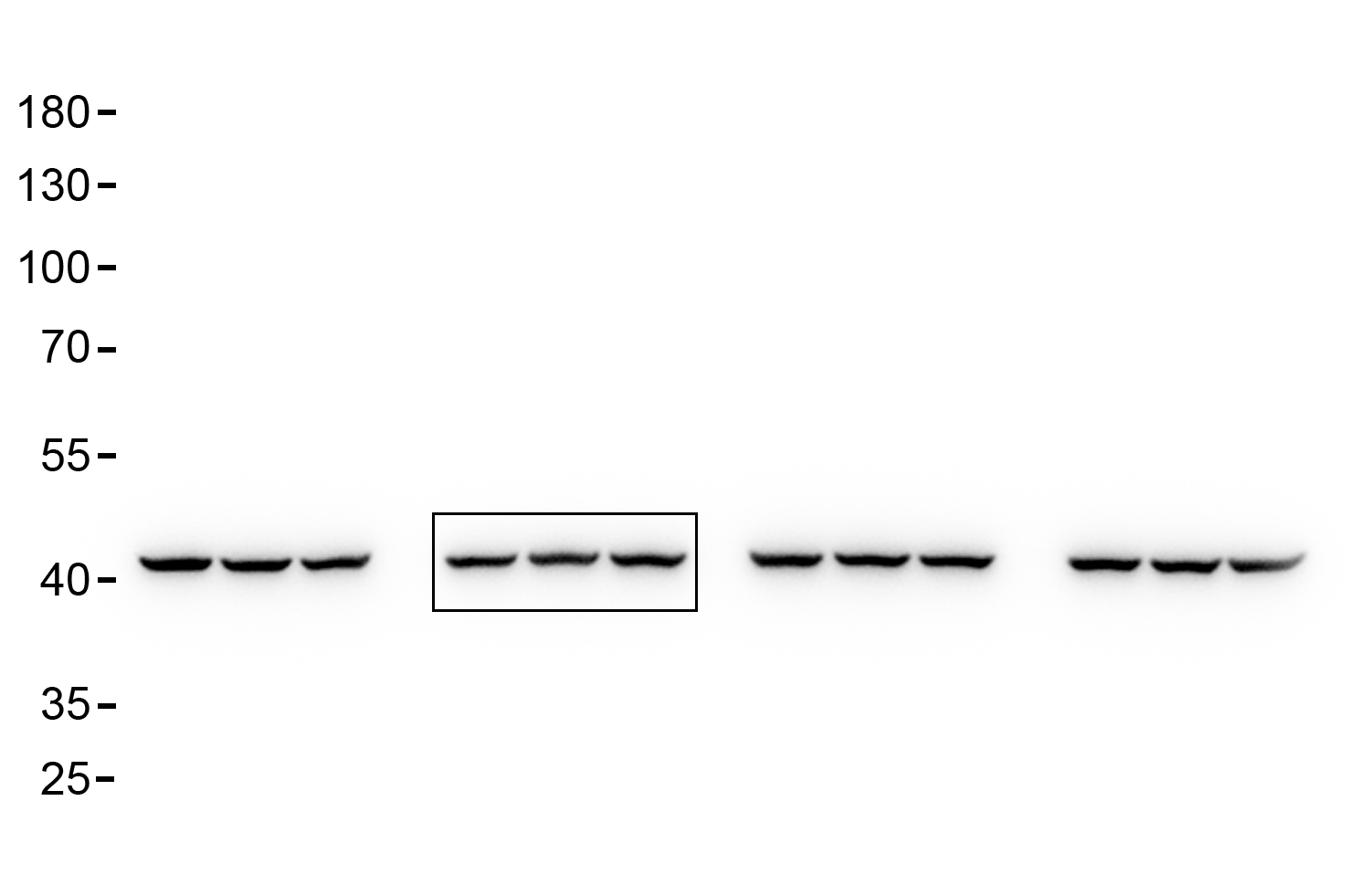

Supplement: Figure 5—source data 2. [file elife-97373-fig5-data2.zip › Figure 5-source data 2/Figure 5H/Actin.tif]

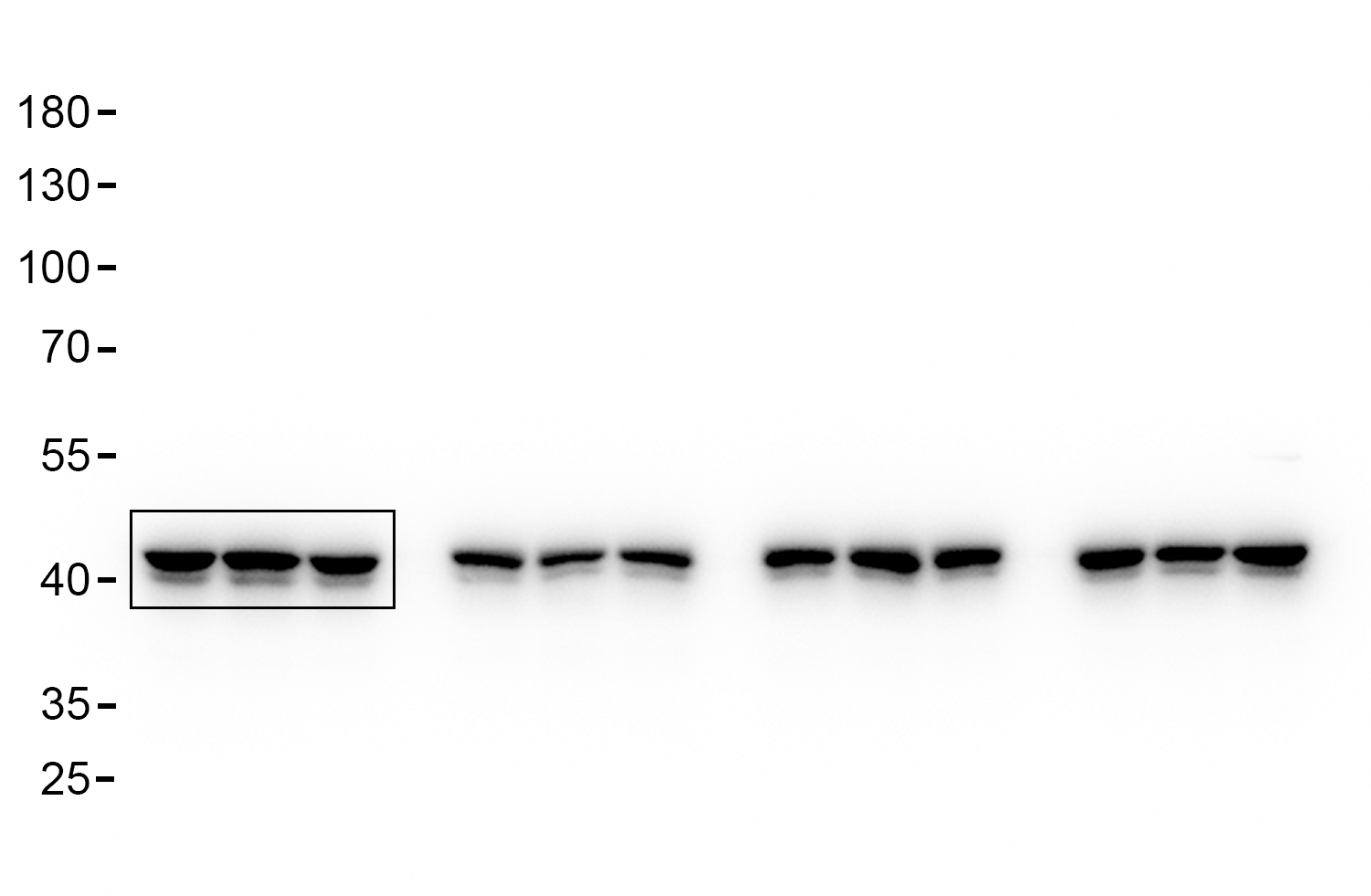

Supplement: Figure 5—source data 2. [file elife-97373-fig5-data2.zip › Figure 5-source data 2/Figure 5H/gsk-3b.tif]

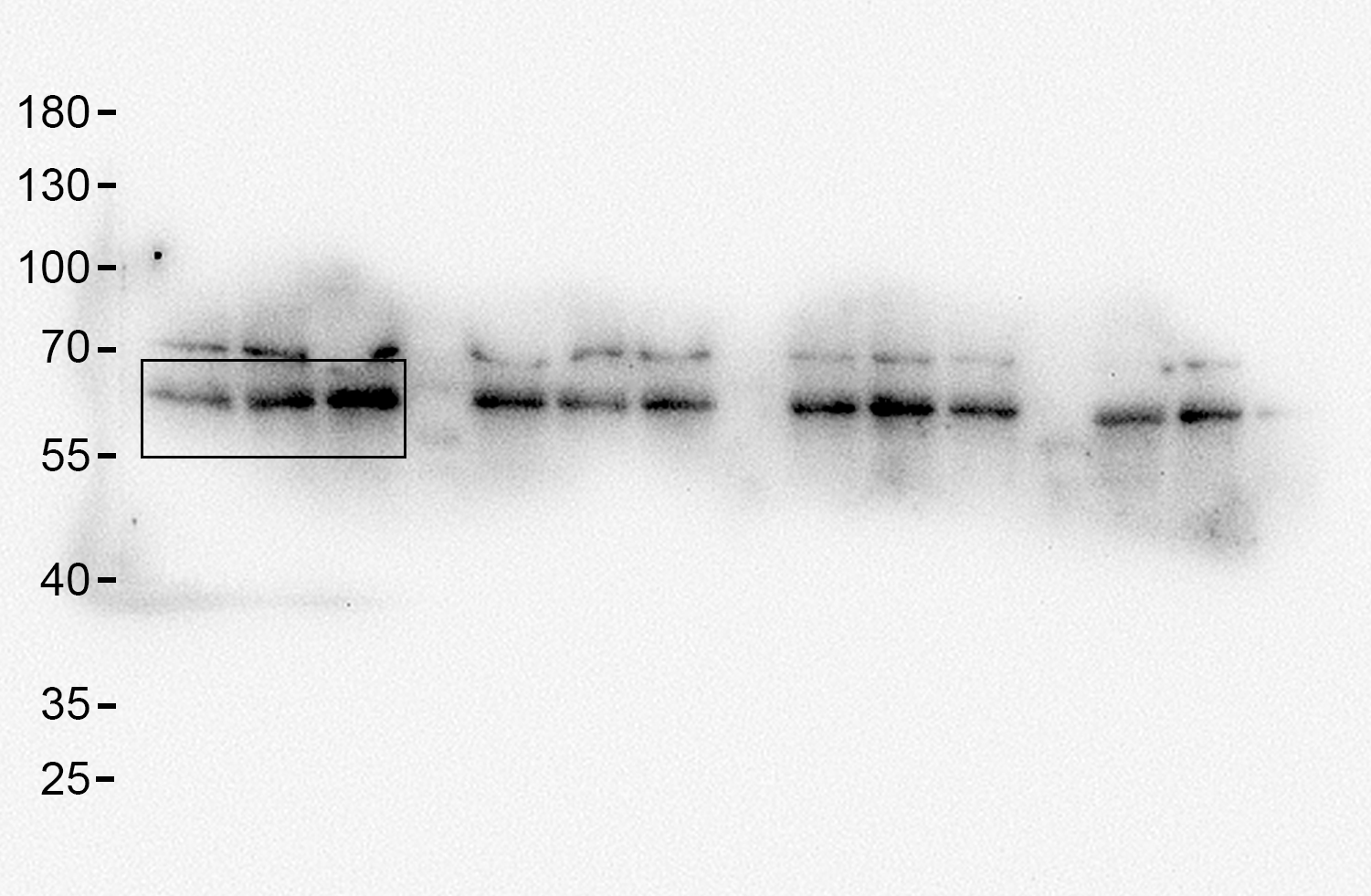

Supplement: Figure 5—source data 2. [file elife-97373-fig5-data2.zip › Figure 5-source data 2/Figure 5H/mmp2.tif]

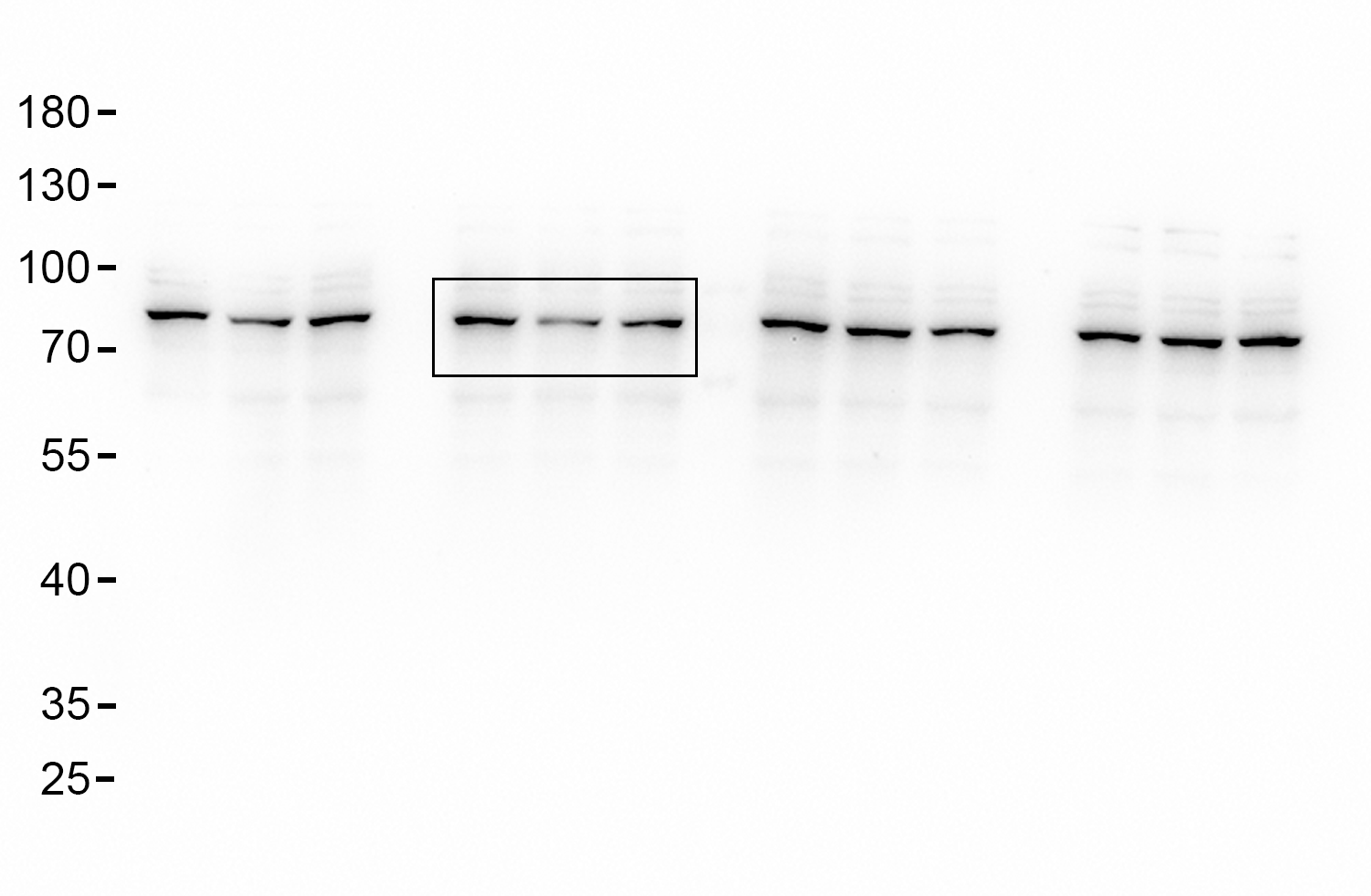

Supplement: Figure 5—source data 2. [file elife-97373-fig5-data2.zip › Figure 5-source data 2/Figure 5H/p-b-catenin.tif]

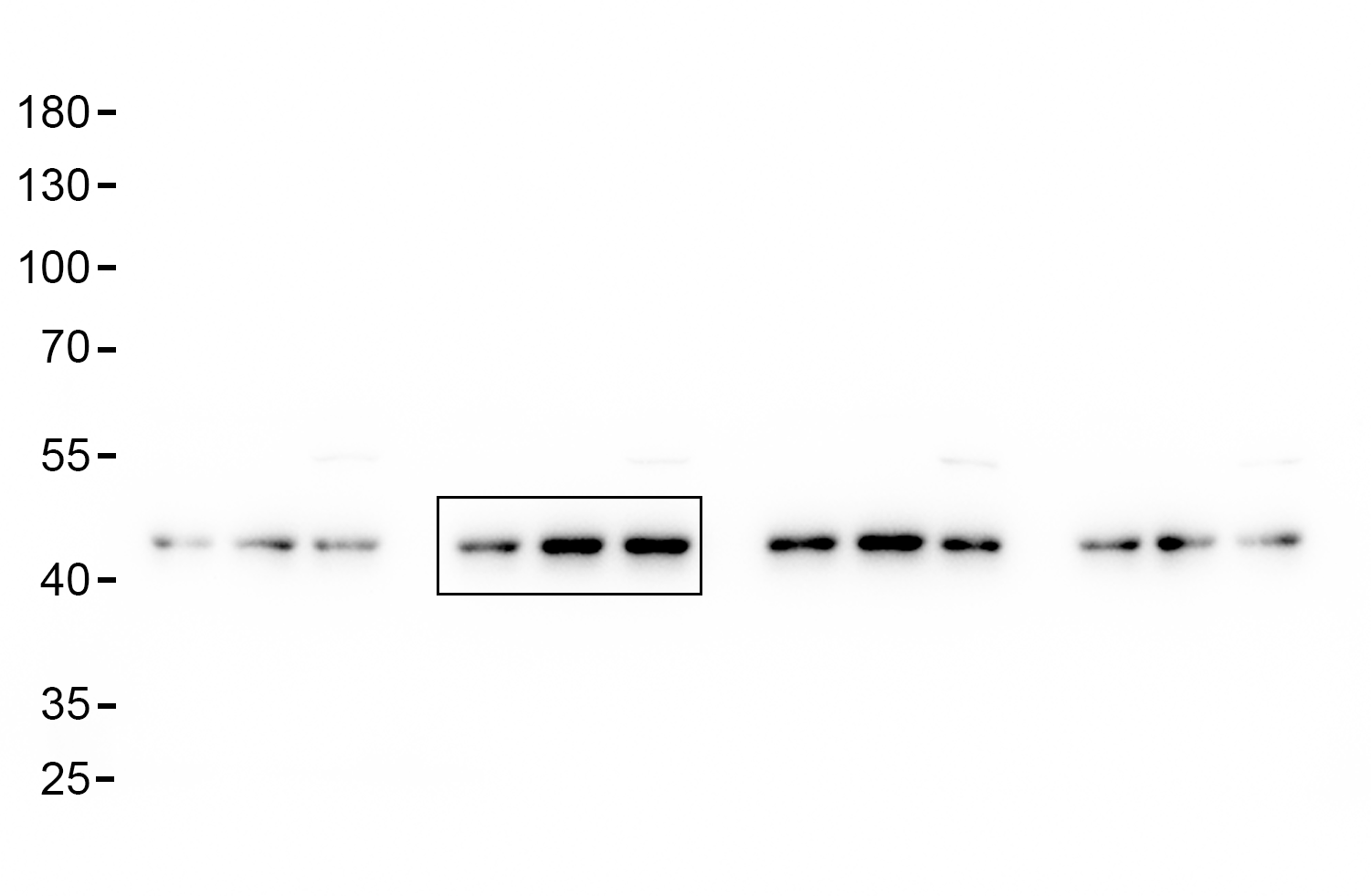

Supplement: Figure 5—source data 2. [file elife-97373-fig5-data2.zip › Figure 5-source data 2/Figure 5H/p-gsk-3b.tif]

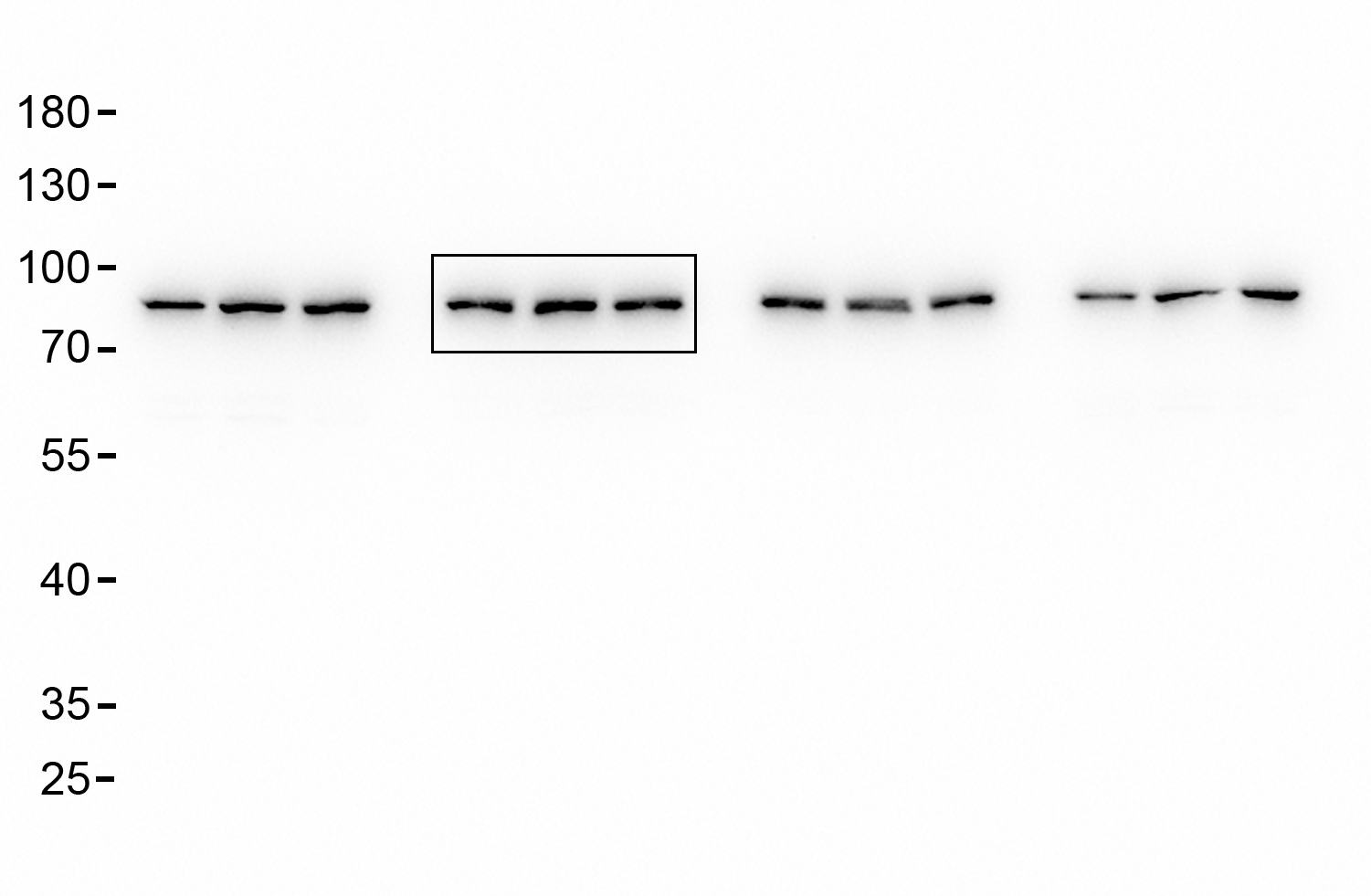

Supplement: Figure 5—source data 2. [file elife-97373-fig5-data2.zip › Figure 5-source data 2/Figure 5H/pkc.tif]

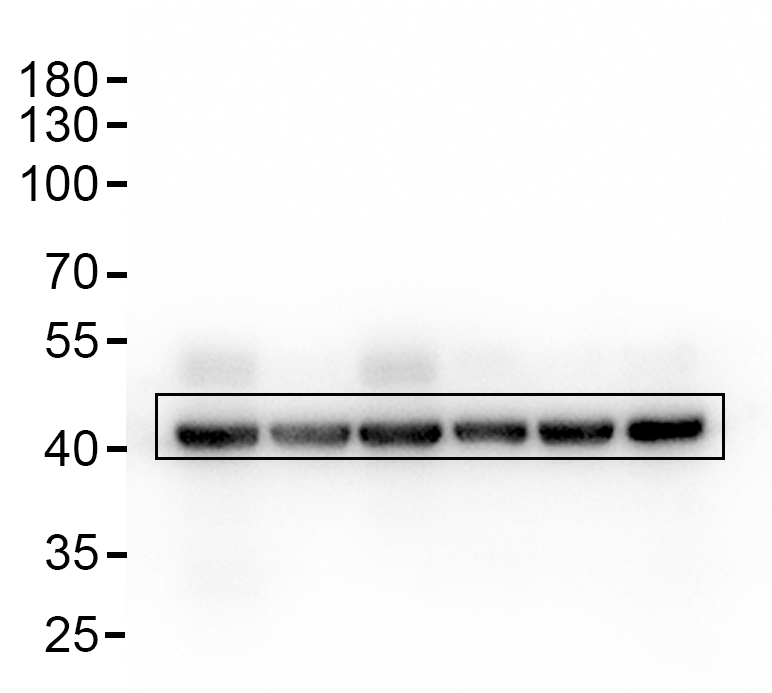

Supplement: Figure 6—source data 3. [file elife-97373-fig6-data3.zip › Figure 6-source data 2/Actin.tif]

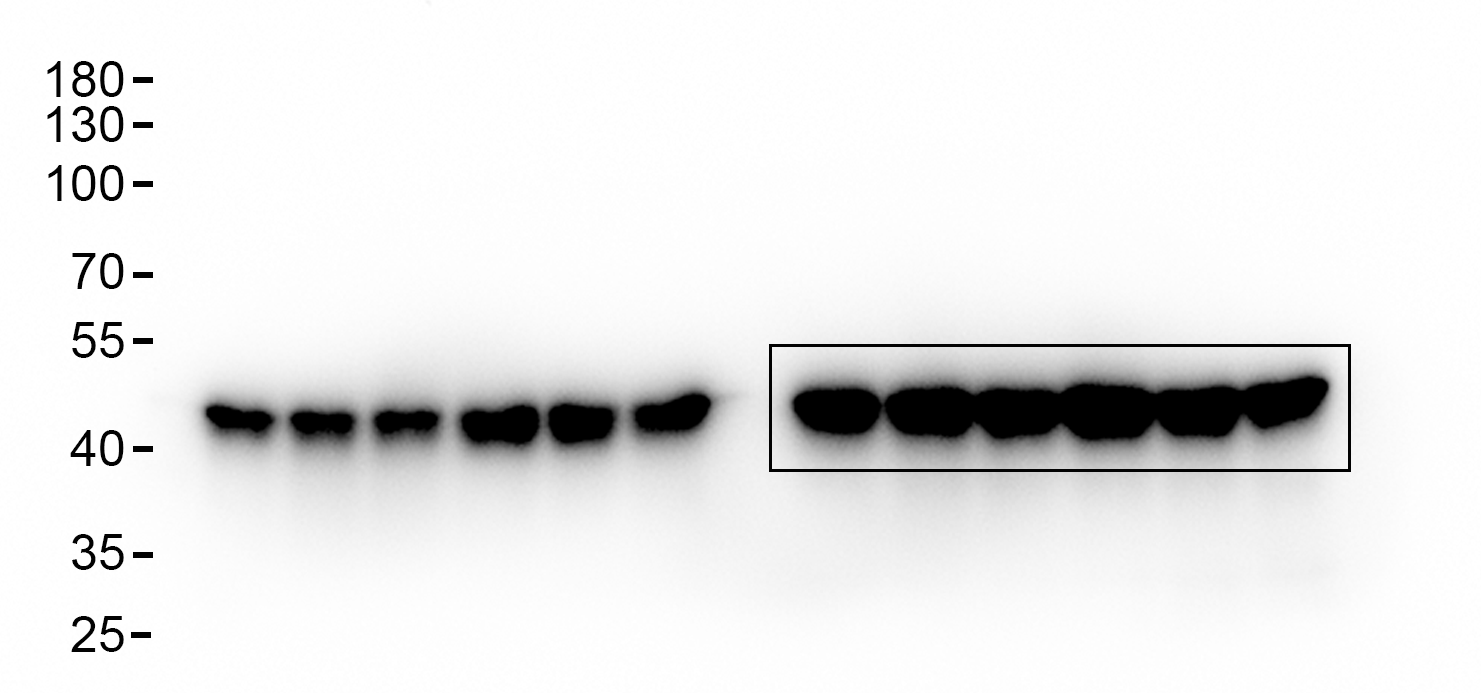

Supplement: Figure 6—source data 3. [file elife-97373-fig6-data3.zip › Figure 6-source data 2/gsk-3b.tif]

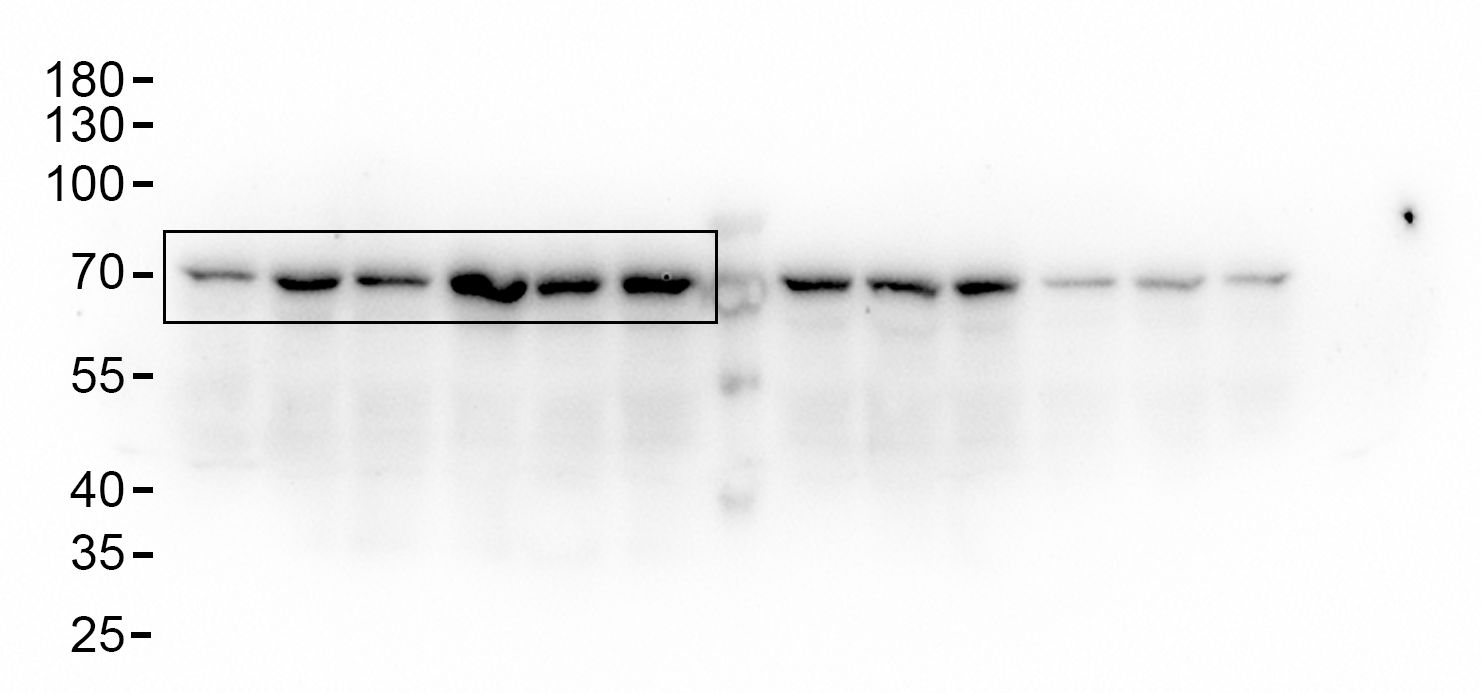

Supplement: Figure 6—source data 3. [file elife-97373-fig6-data3.zip › Figure 6-source data 2/mmp2.tif]

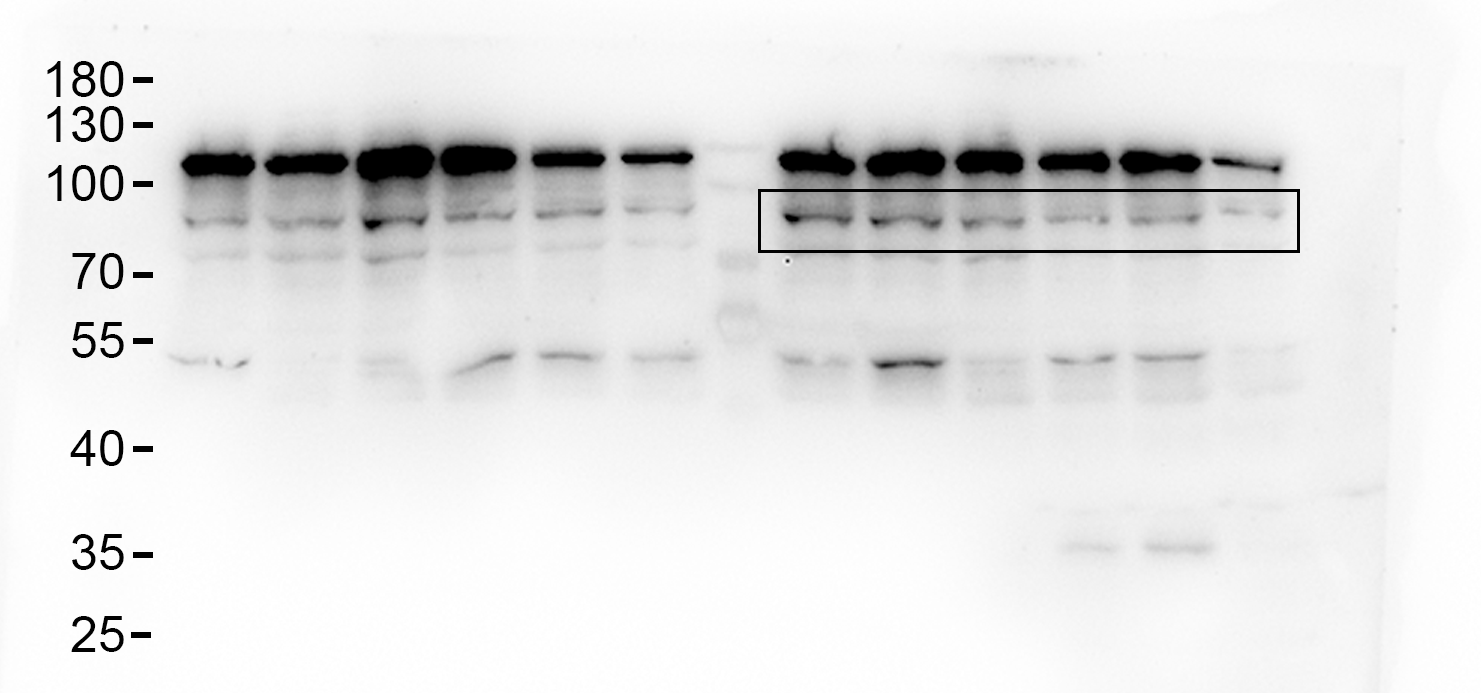

Supplement: Figure 6—source data 3. [file elife-97373-fig6-data3.zip › Figure 6-source data 2/p-b-catenin.tif]

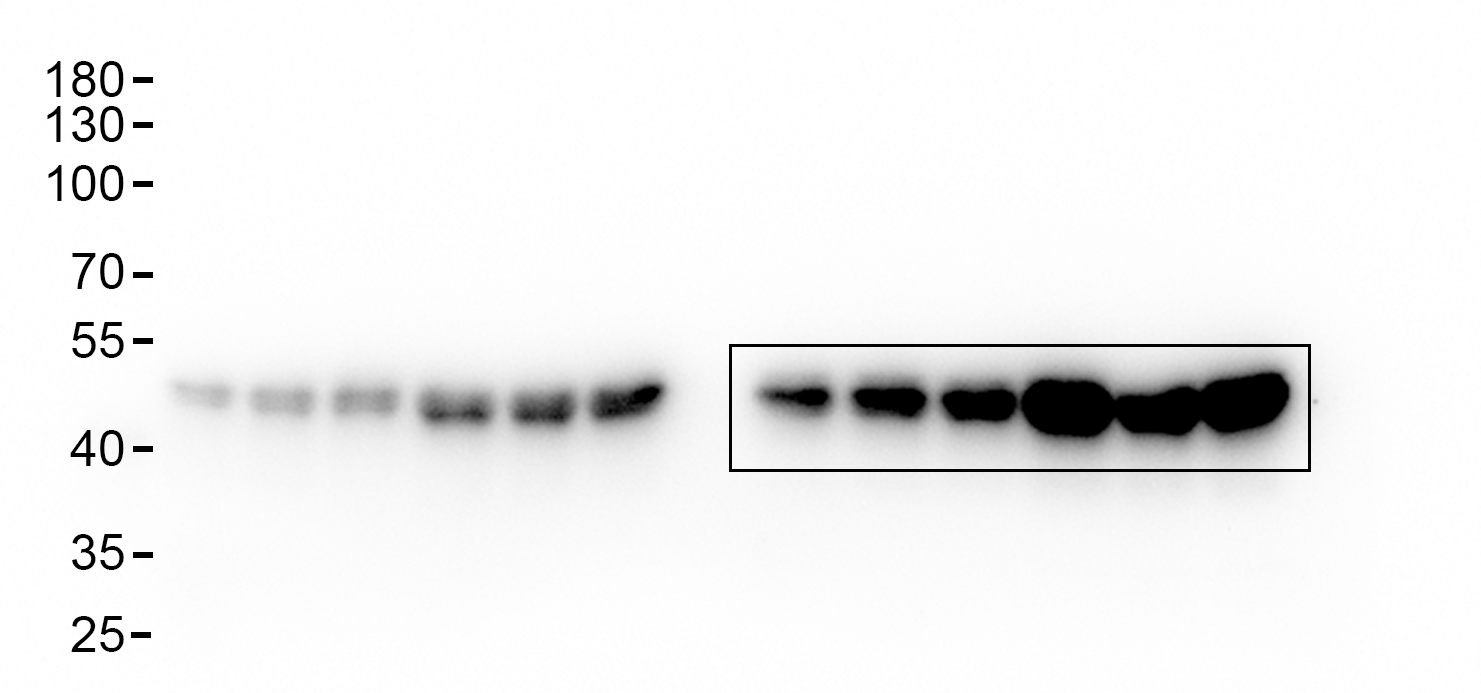

Supplement: Figure 6—source data 3. [file elife-97373-fig6-data3.zip › Figure 6-source data 2/p-gsk-3b.tif]

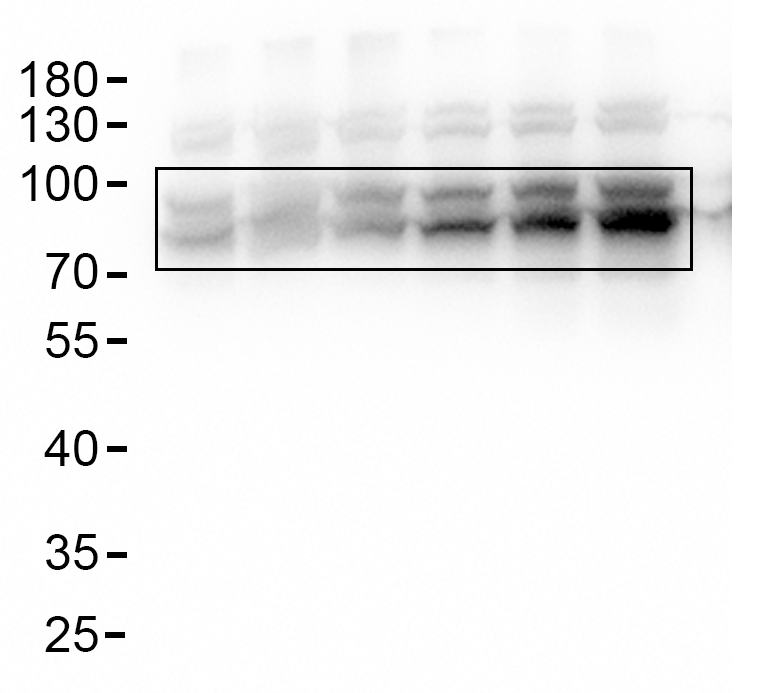

Supplement: Figure 6—source data 3. [file elife-97373-fig6-data3.zip › Figure 6-source data 2/p-pkc.tif]

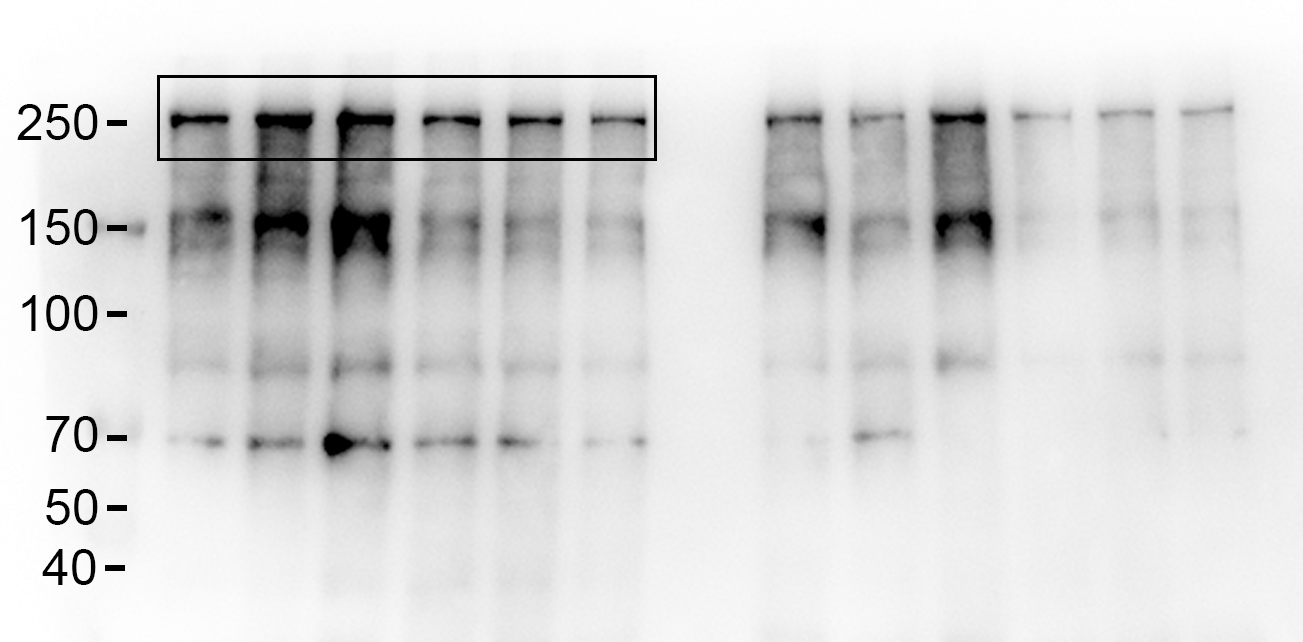

Supplement: Figure 6—source data 3. [file elife-97373-fig6-data3.zip › Figure 6-source data 2/p-plce1.tif]

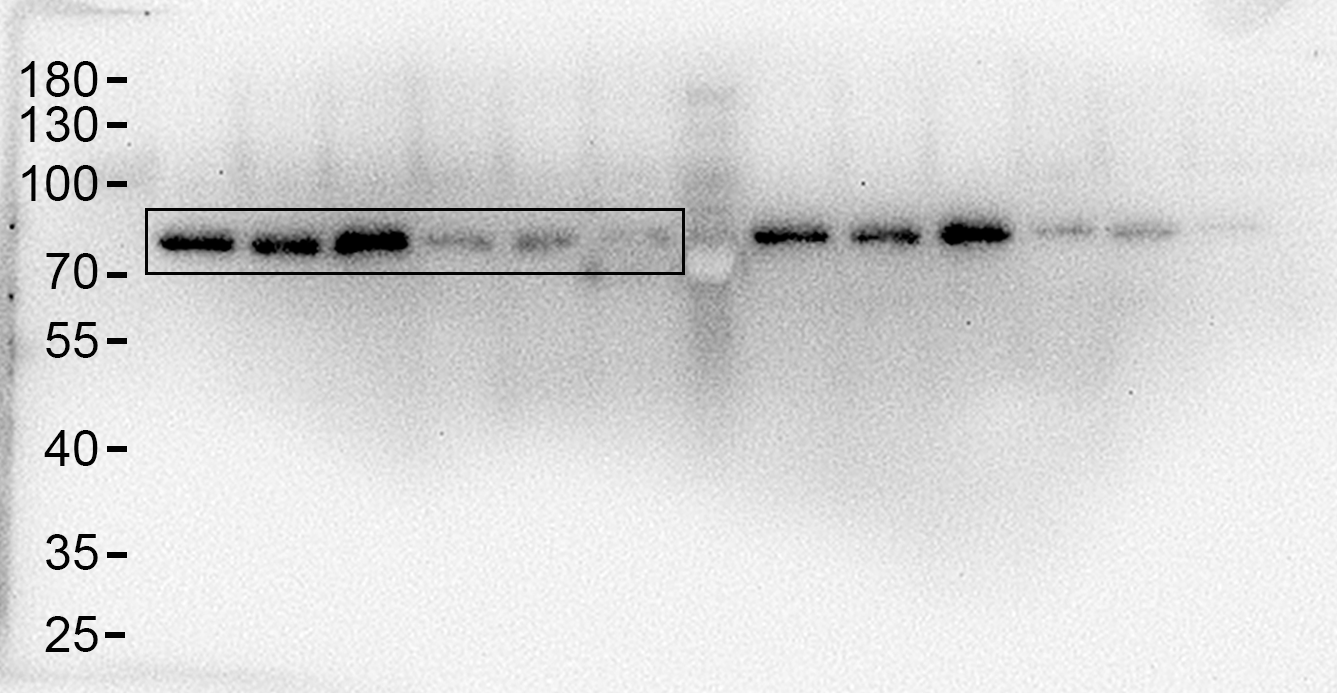

Supplement: Figure 6—source data 3. [file elife-97373-fig6-data3.zip › Figure 6-source data 2/p-TAK1.tif]

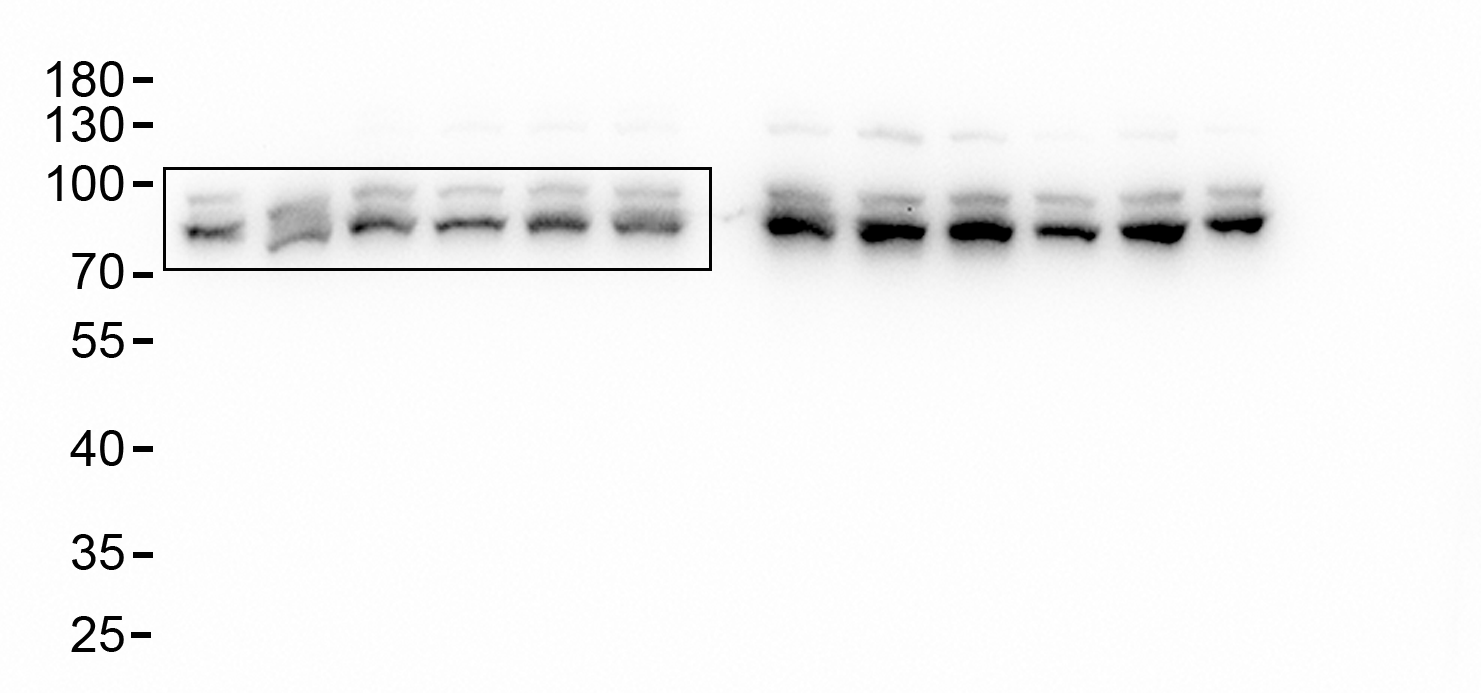

Supplement: Figure 6—source data 3. [file elife-97373-fig6-data3.zip › Figure 6-source data 2/pkc.tif]

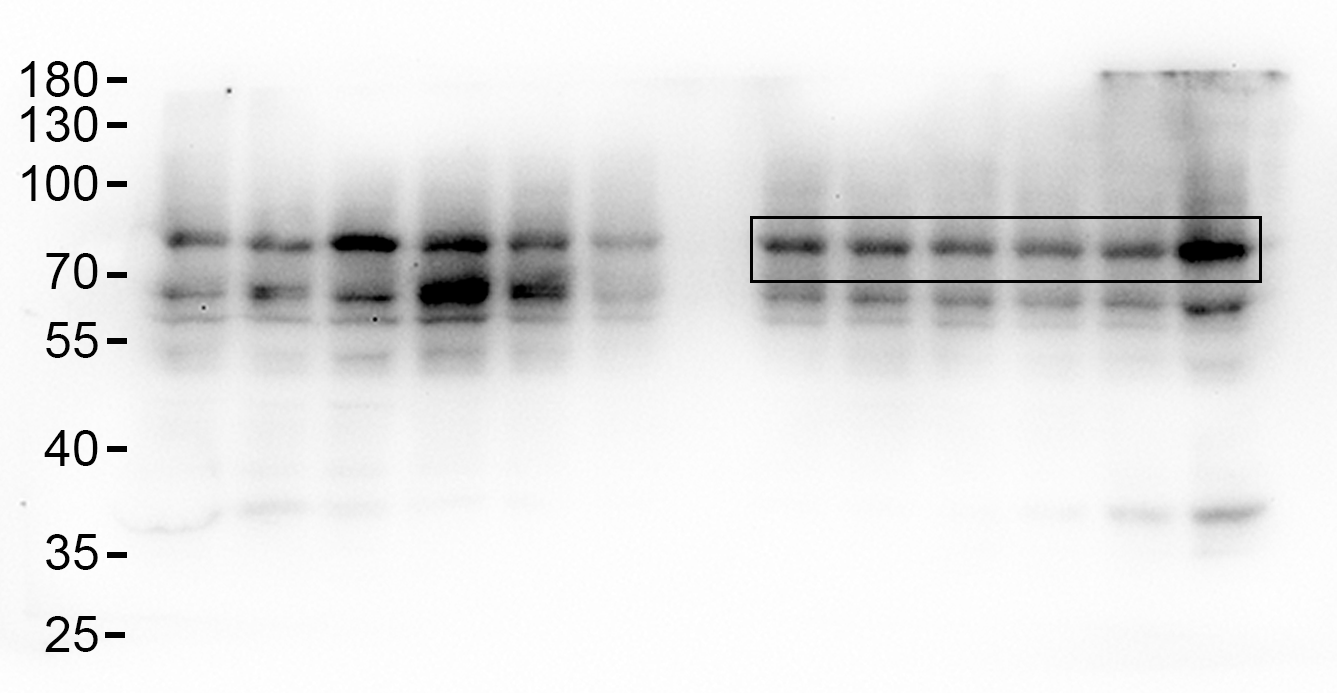

Supplement: Figure 6—source data 3. [file elife-97373-fig6-data3.zip › Figure 6-source data 2/TAK1.tif]

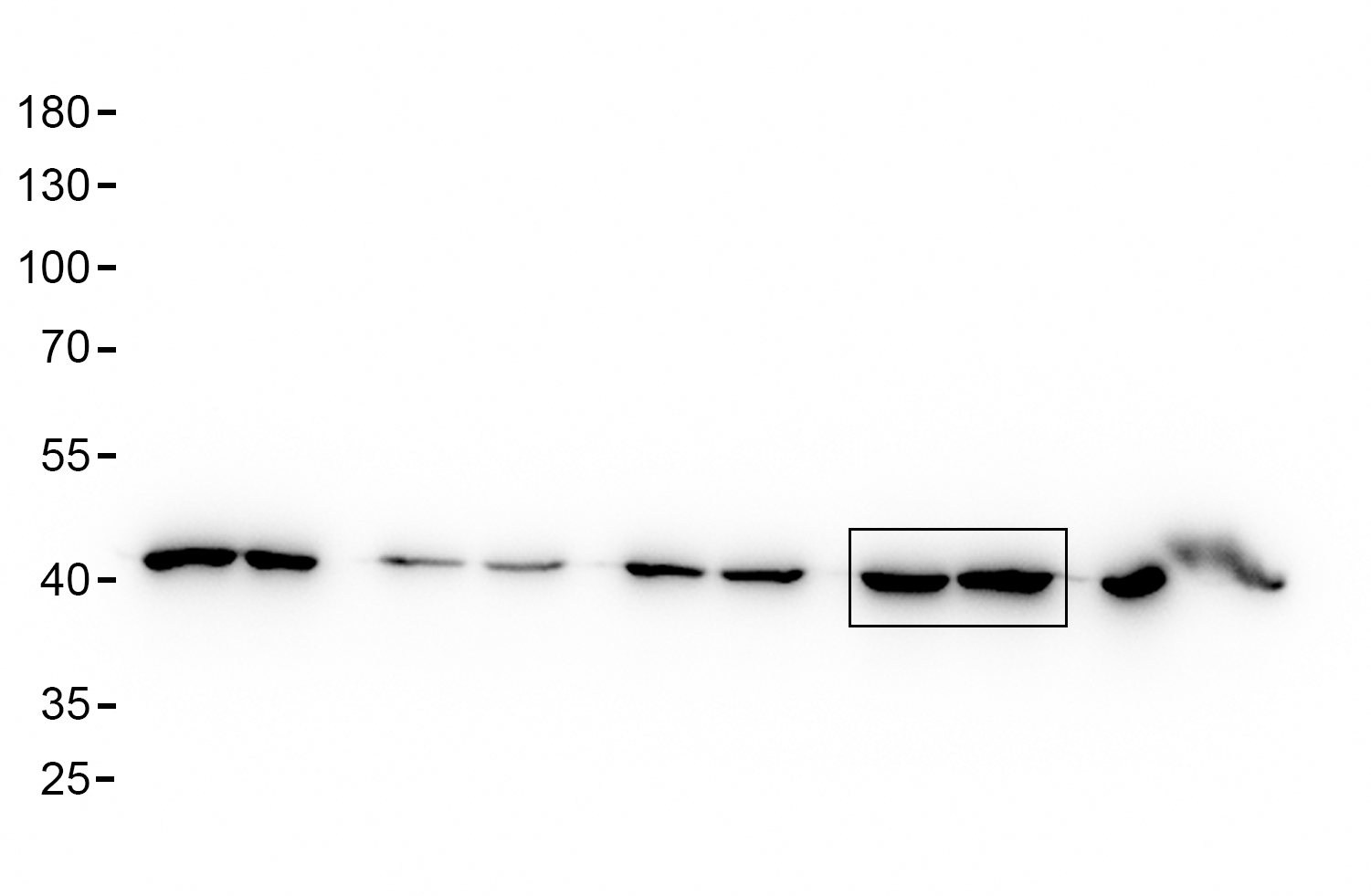

Supplement: Figure 6—figure supplement 1—source data 3. [file elife-97373-fig6-figsupp1-data3.zip › Figure 6-figure supplement 1-source data 2/Actin.tif]

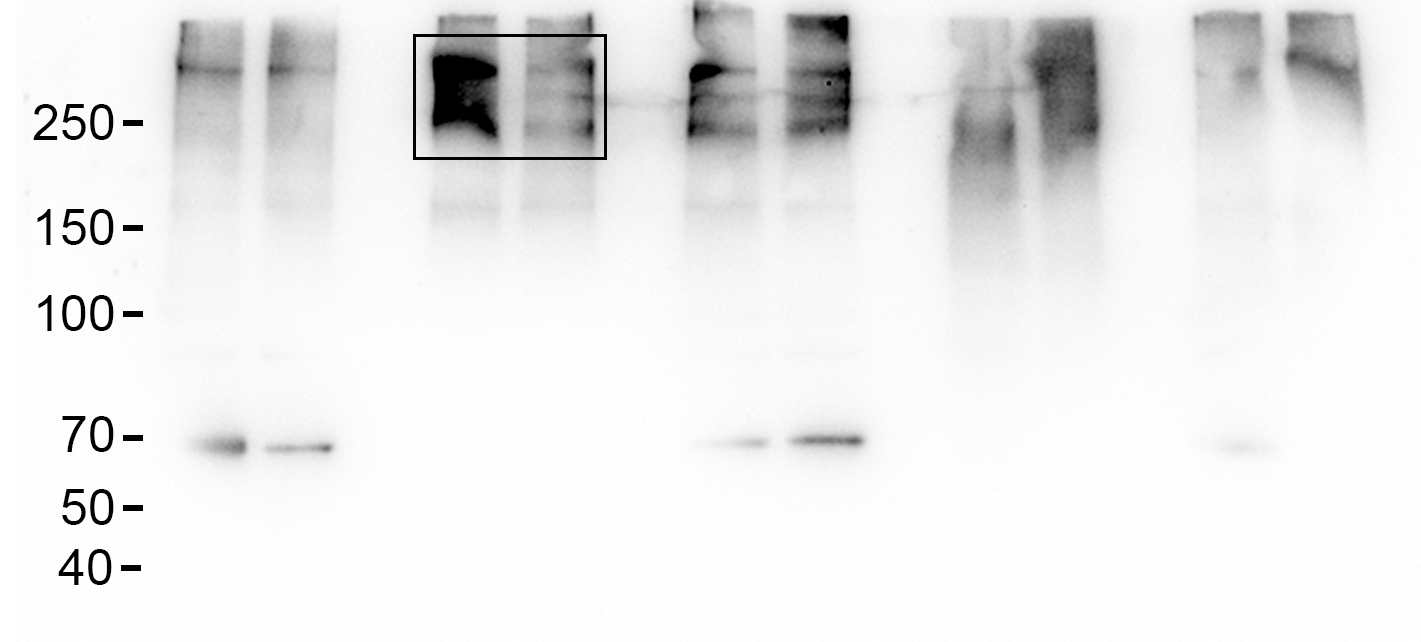

Supplement: Figure 6—figure supplement 1—source data 3. [file elife-97373-fig6-figsupp1-data3.zip › Figure 6-figure supplement 1-source data 2/plce1.tif]

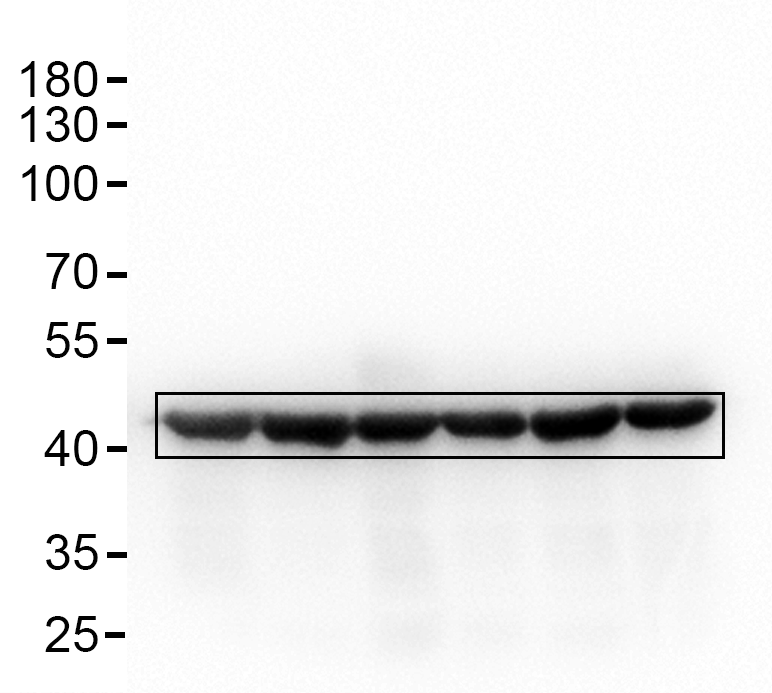

Supplement: Figure 7—source data 3. [file elife-97373-fig7-data3.zip › Figure 7-source data 2/Actin.tif]

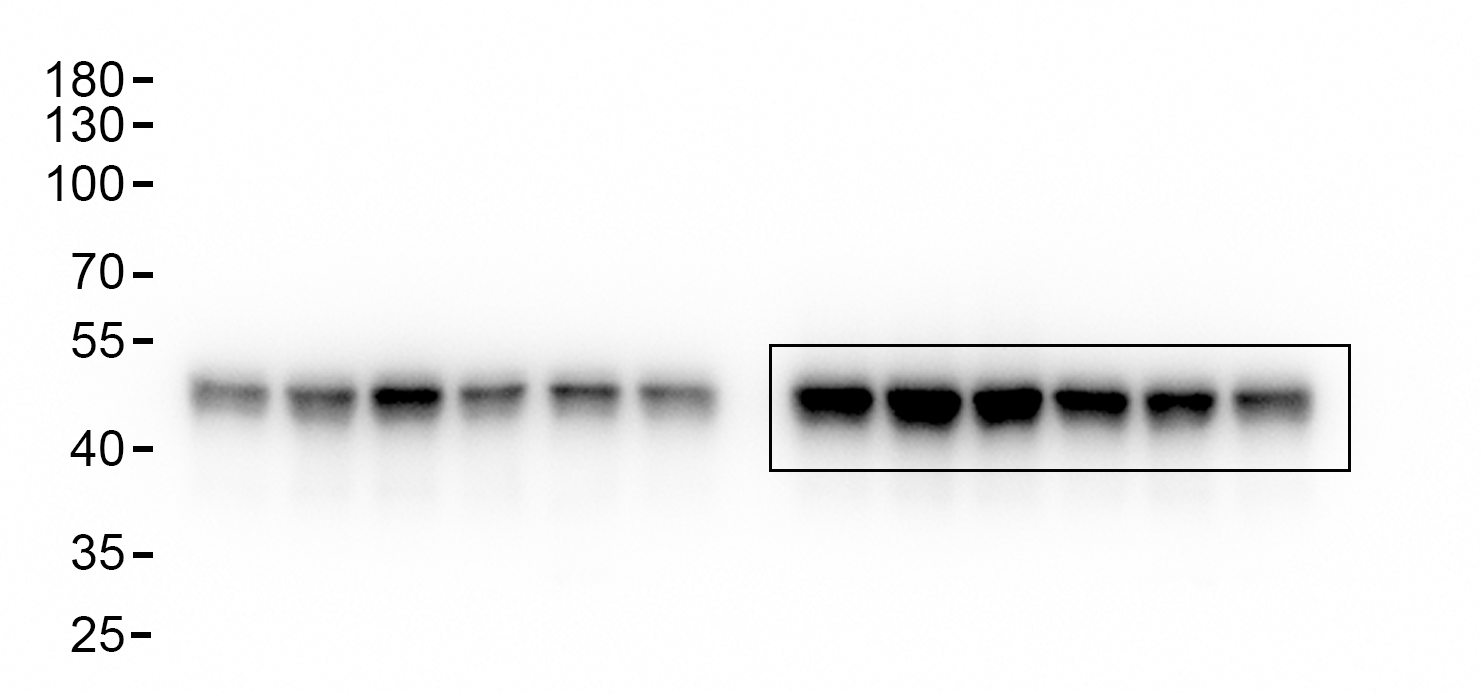

Supplement: Figure 7—source data 3. [file elife-97373-fig7-data3.zip › Figure 7-source data 2/gsk-3b.tif]

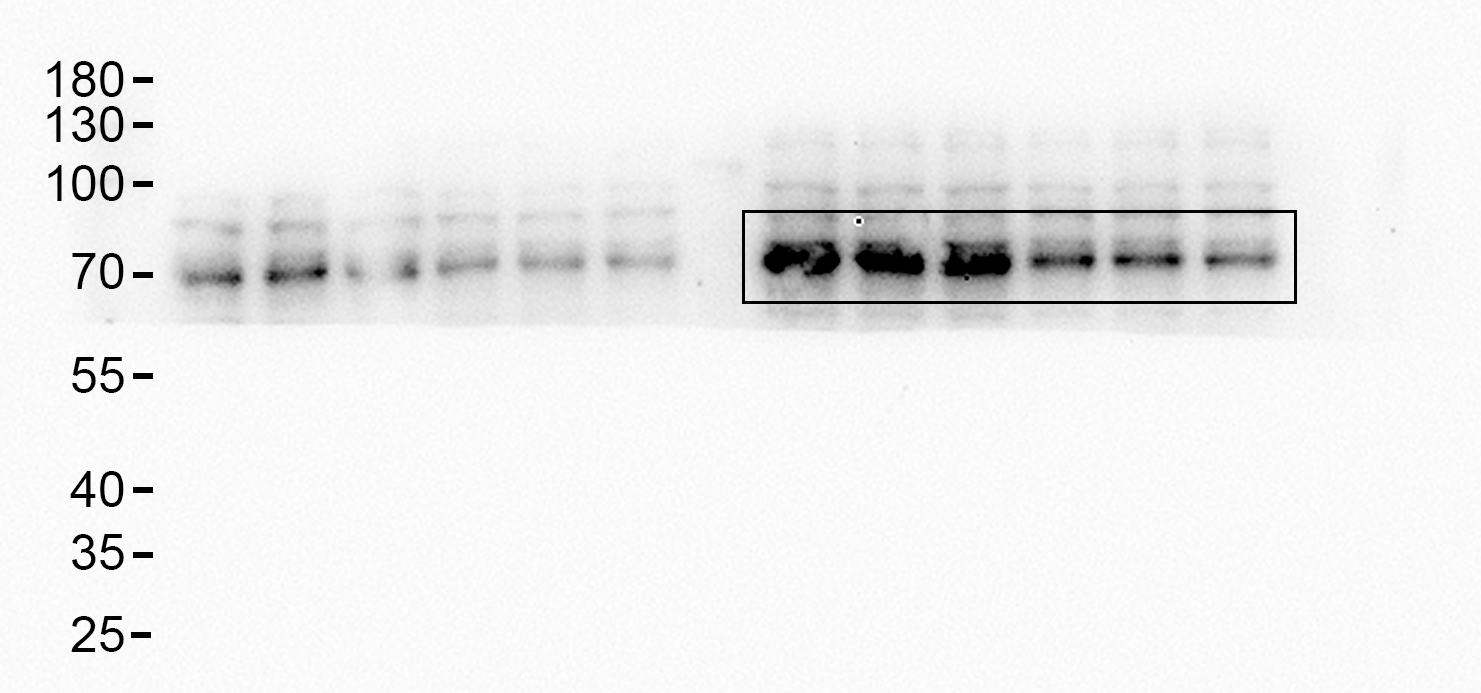

Supplement: Figure 7—source data 3. [file elife-97373-fig7-data3.zip › Figure 7-source data 2/mmp2.tif]

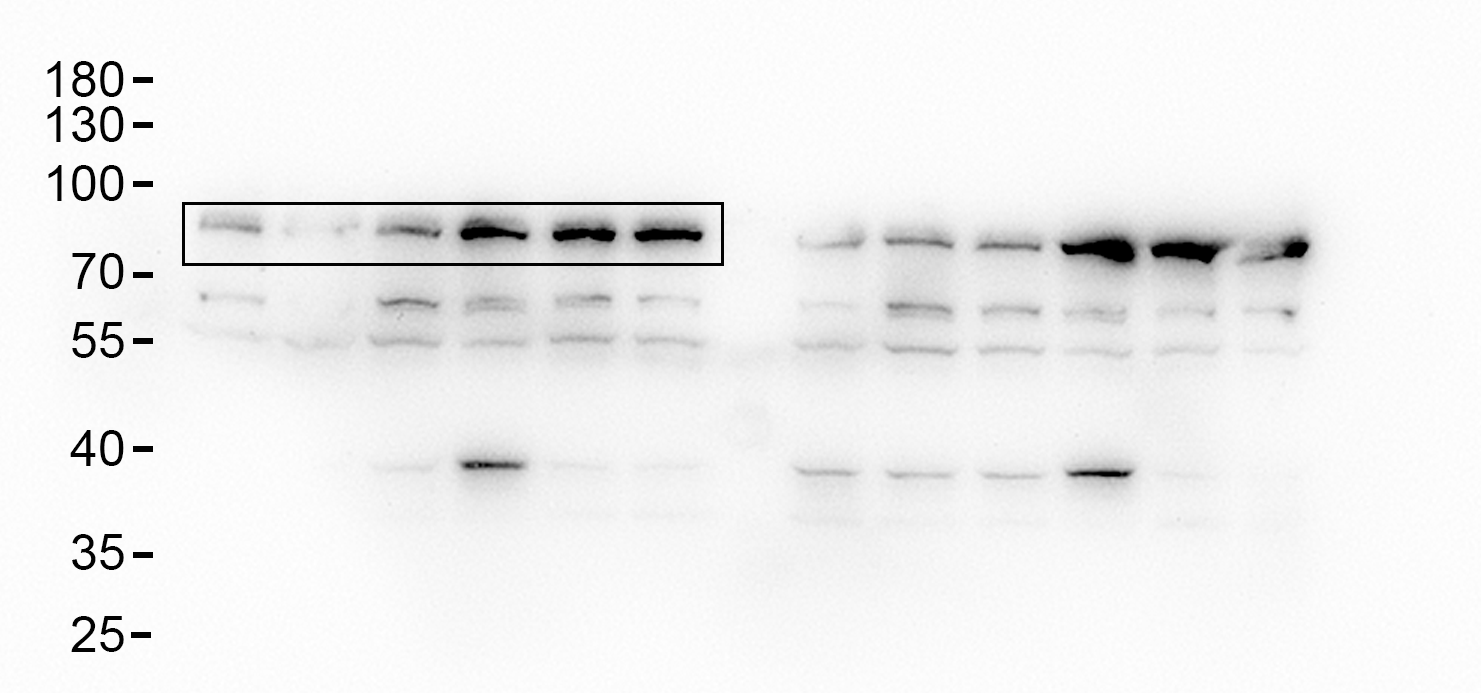

Supplement: Figure 7—source data 3. [file elife-97373-fig7-data3.zip › Figure 7-source data 2/p-b-catenin.tif]

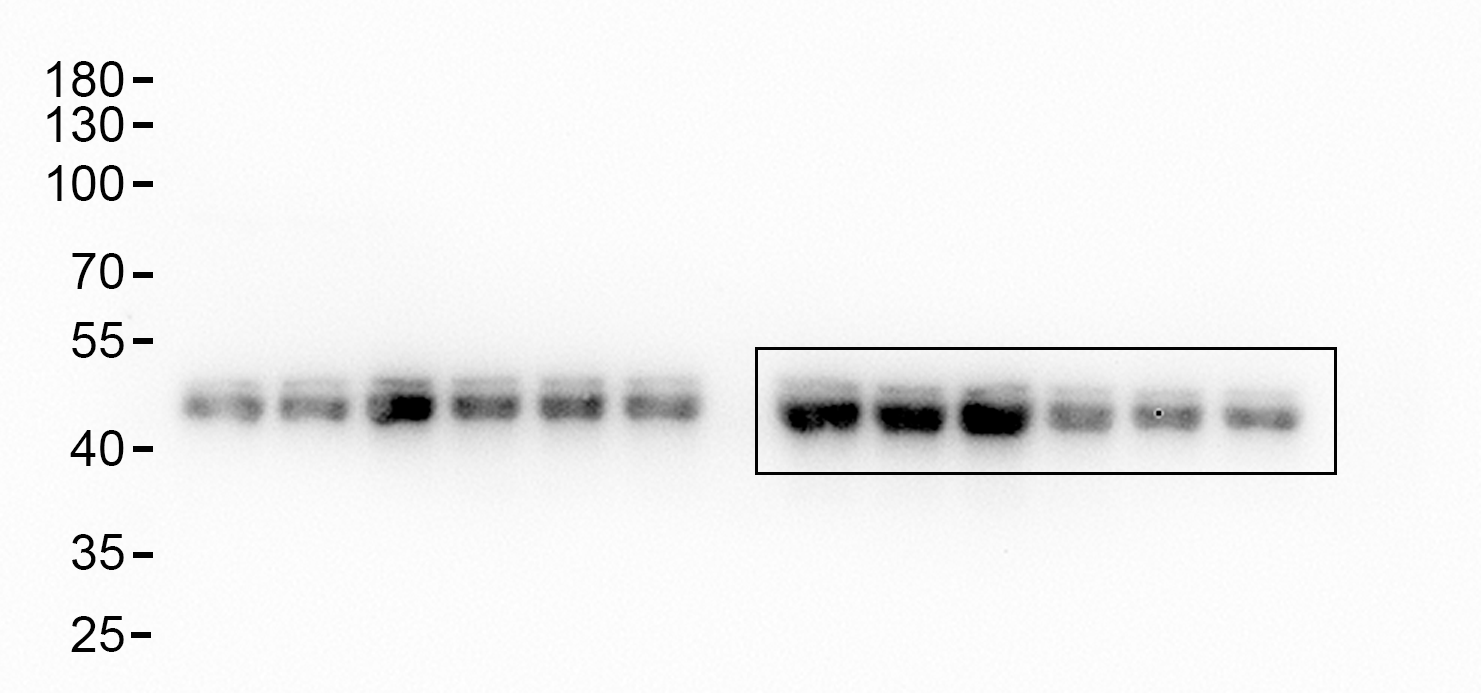

Supplement: Figure 7—source data 3. [file elife-97373-fig7-data3.zip › Figure 7-source data 2/p-gsk-3b.tif]

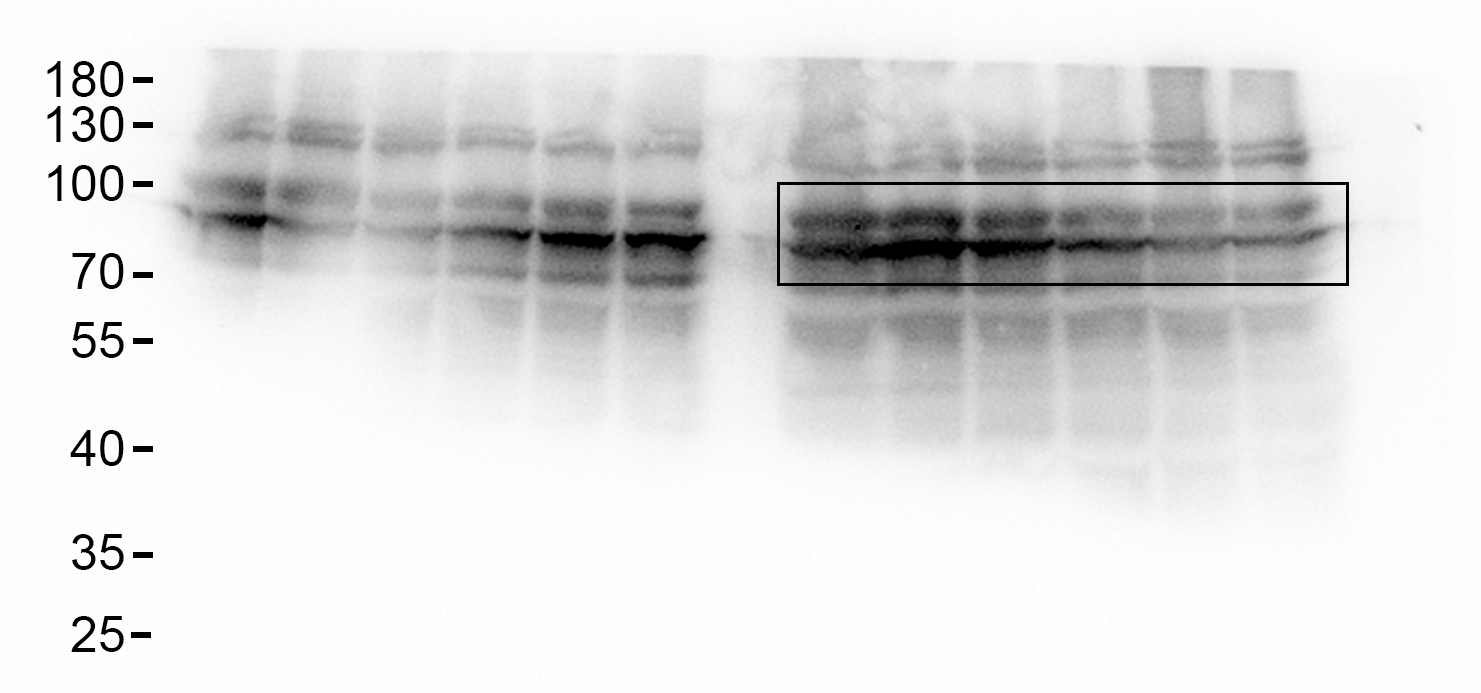

Supplement: Figure 7—source data 3. [file elife-97373-fig7-data3.zip › Figure 7-source data 2/p-pkc.tif]

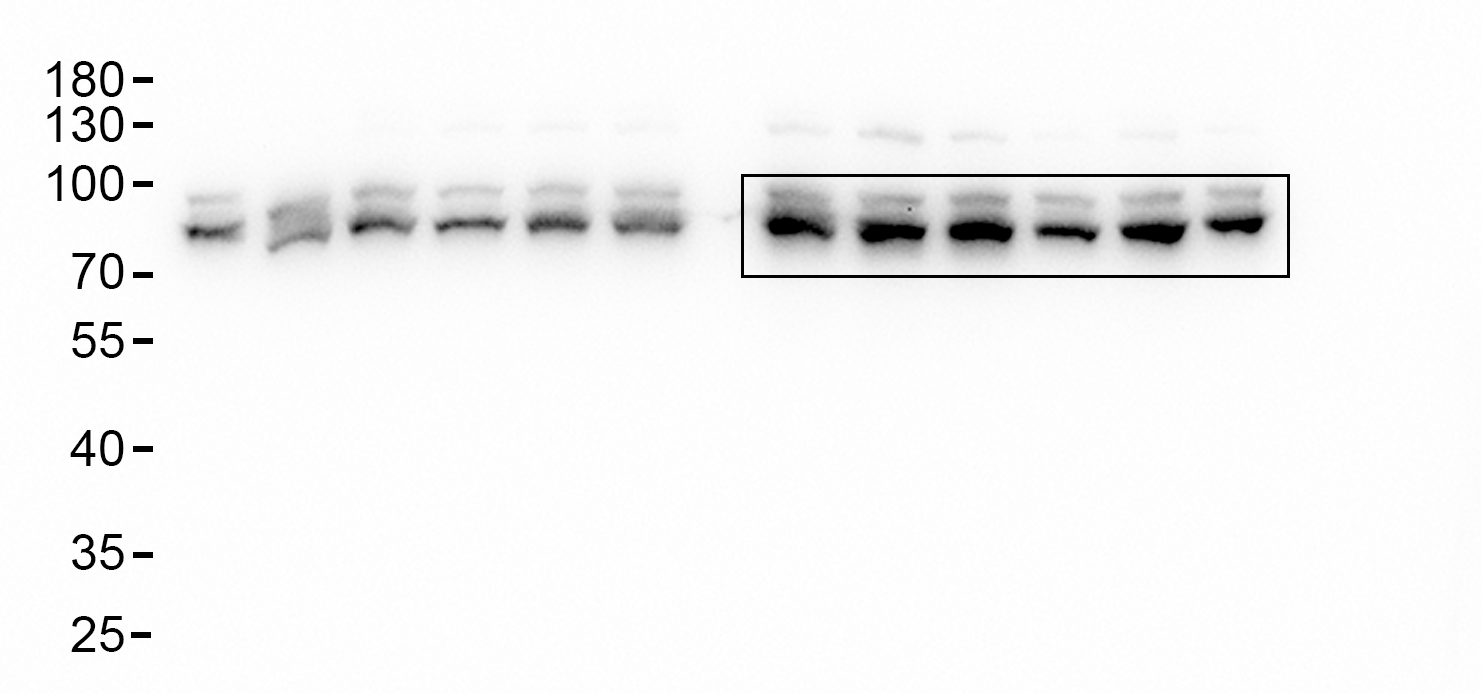

Supplement: Figure 7—source data 3. [file elife-97373-fig7-data3.zip › Figure 7-source data 2/pkc.tif]

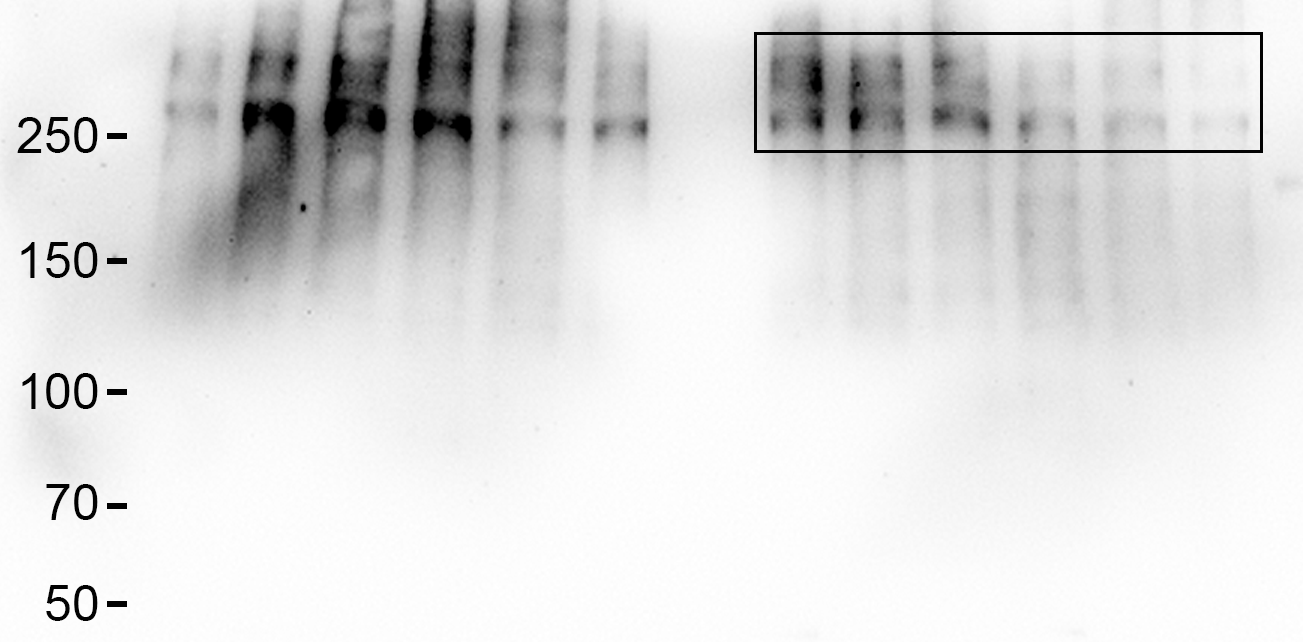

Supplement: Figure 7—source data 3. [file elife-97373-fig7-data3.zip › Figure 7-source data 2/plce1.tif]
