# Supplementary material for: TAK1-mediated phosphorylation of PLCE1 represses PIP2 hydrolysis to impede esophageal squamous cancer metastasis
Source: eLife. 2025 Apr 23;13:RP97373. doi: 10.7554/eLife.97373 (PMC12017773; doi:10.7554/eLife.97373)
Supplement: Figure 5—source data 1. [file elife-97373-fig5-data1.zip › Figure 5-source data 1/Figure 5-source data 1.pdf]

## Figure 5A

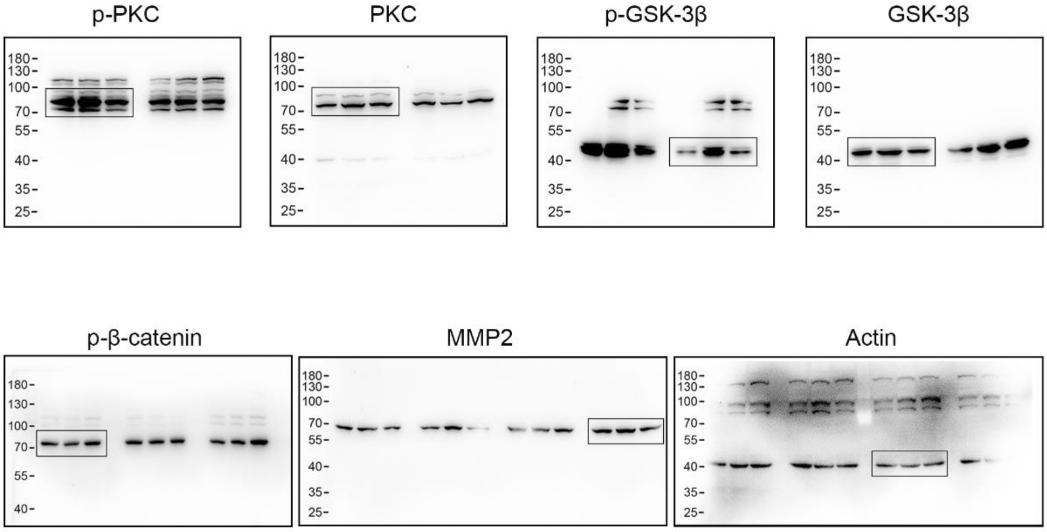


## Figure 5B

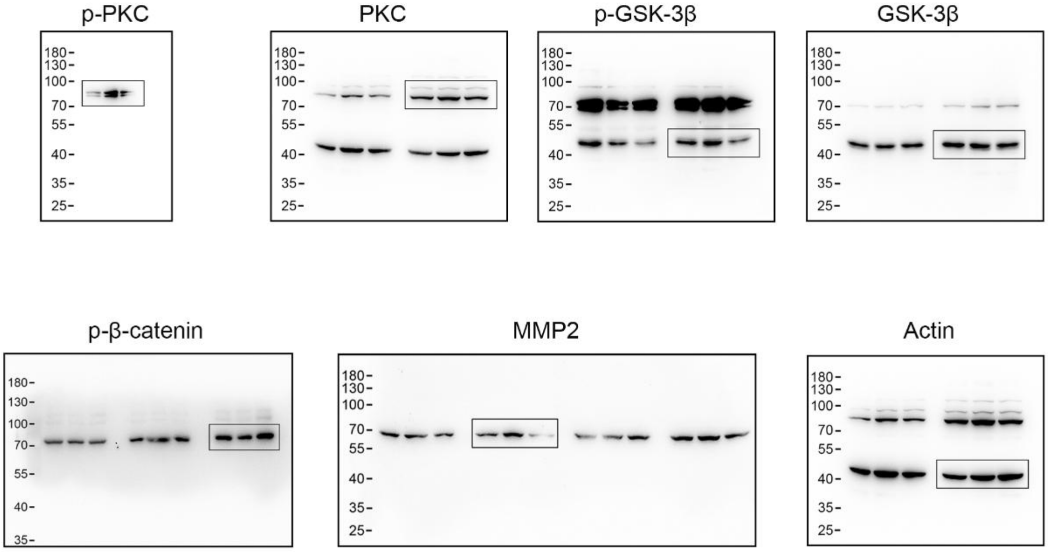

## Figure 5C

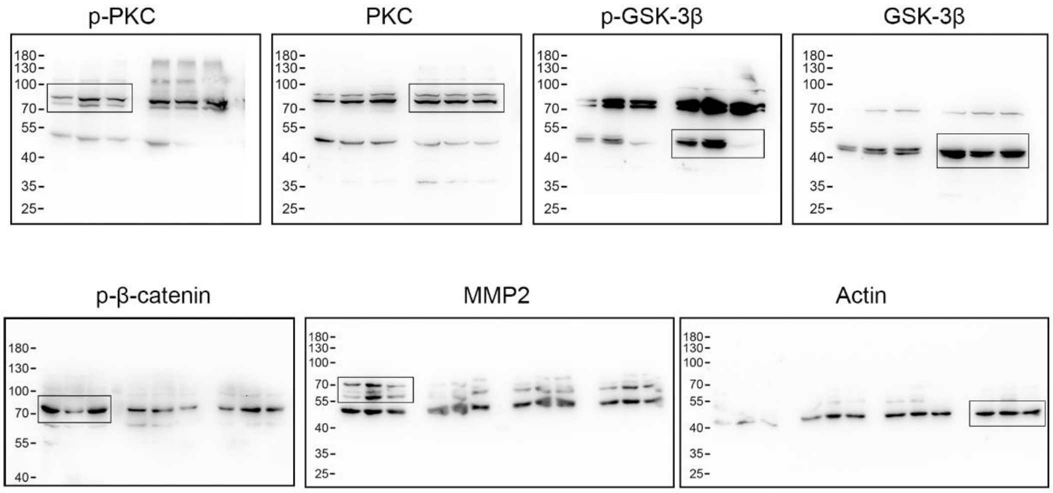


## Figure 5E

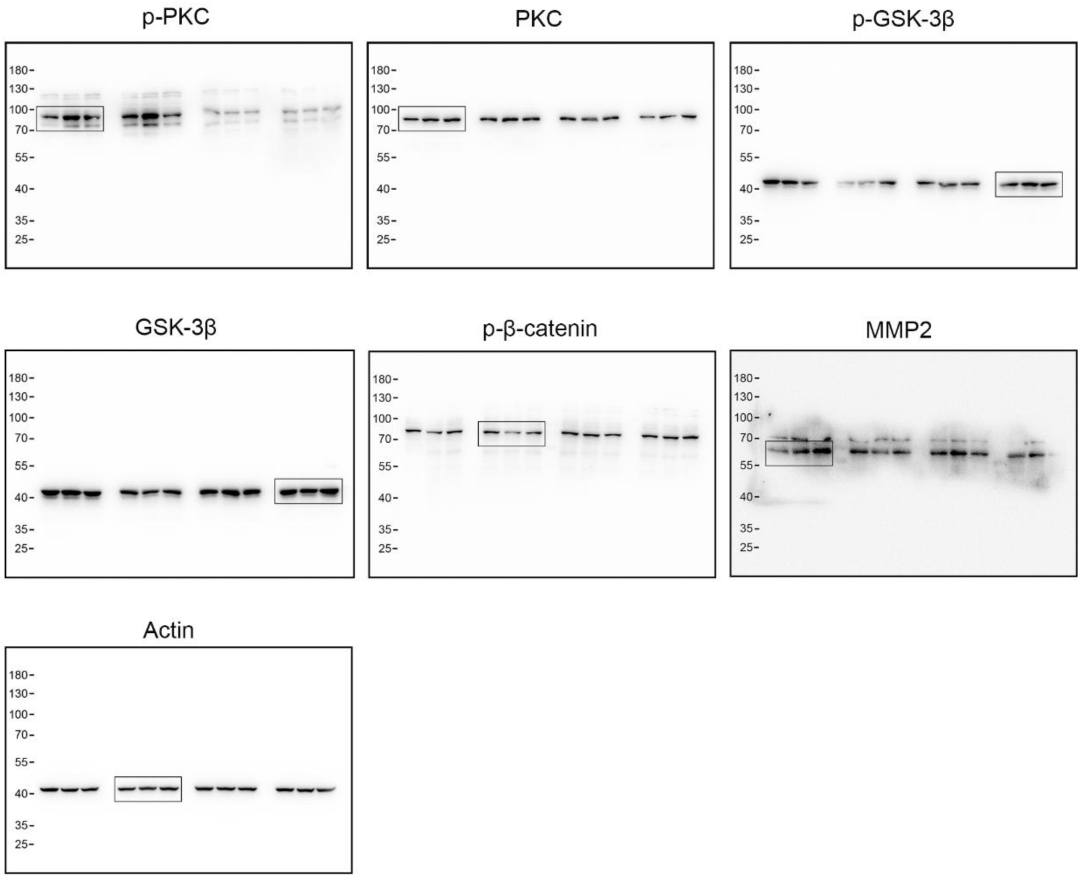

**Figure 5G**

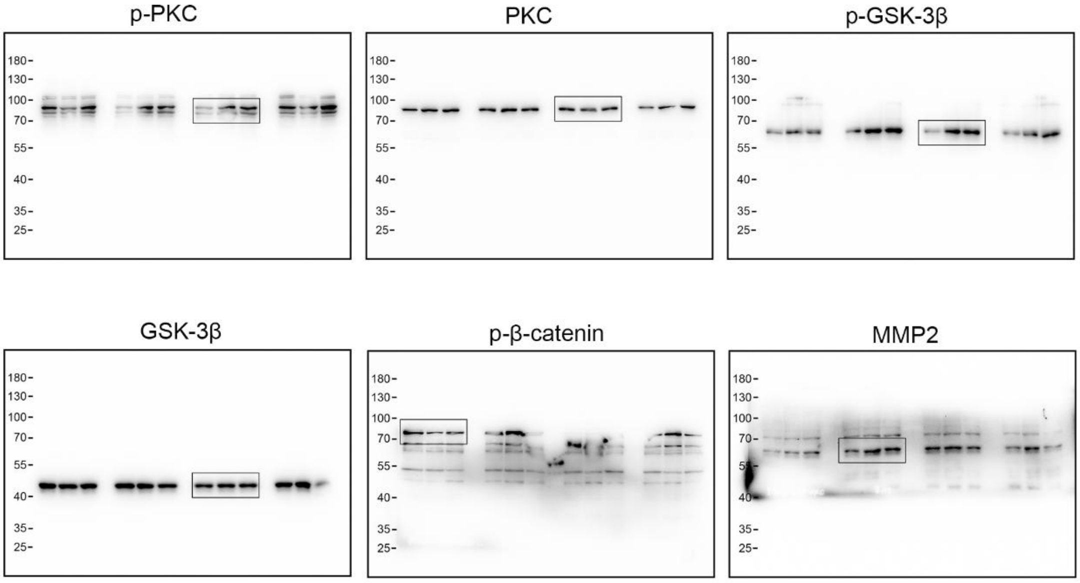


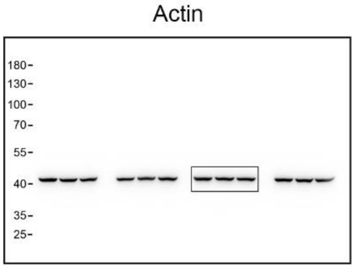


**Figure 5H**

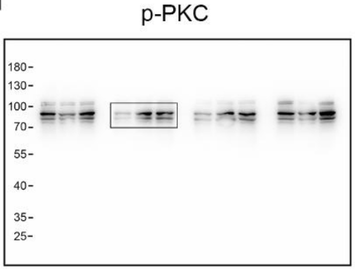


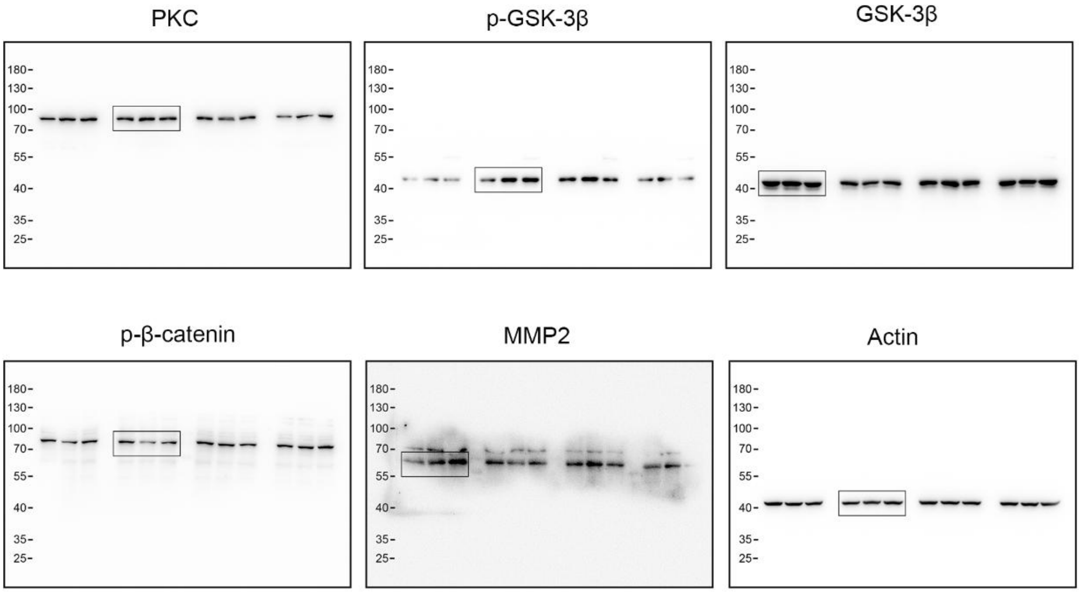


Figure 5, Source Data 1. Original membranes corresponding to Figure 5, panel A, B, C, E, G and H, indicating the relevant bands.
