# Supplementary material for: TAK1-mediated phosphorylation of PLCE1 represses PIP2 hydrolysis to impede esophageal squamous cancer metastasis
Source: eLife. 2025 Apr 23;13:RP97373. doi: 10.7554/eLife.97373 (PMC12017773; doi:10.7554/eLife.97373)
Supplement: Figure 6—figure supplement 1—source data 2. [file elife-97373-fig6-figsupp1-data2.zip › Figure 6-figure supplement 1-source data 1/Figure 6-figure supplement 1-source data 1.pdf]

Figure 6-figure supplement 1C

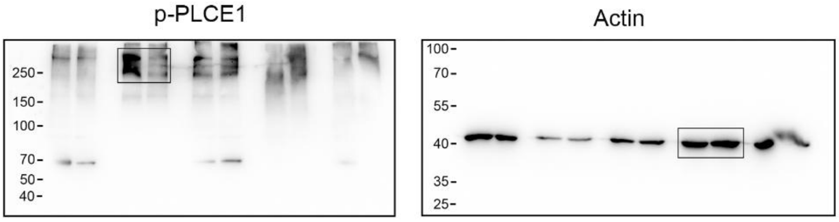

Figure 6-figure supplement 1, Source Data 1. Original membranes corresponding to Figure 6-figure supplement 1, panel C, indicating the relevant bands.
