# Supplementary material for: TAK1-mediated phosphorylation of PLCE1 represses PIP2 hydrolysis to impede esophageal squamous cancer metastasis
Source: eLife. 2025 Apr 23;13:RP97373. doi: 10.7554/eLife.97373 (PMC12017773; doi:10.7554/eLife.97373)
Supplement: Figure 7—source data 2. [file elife-97373-fig7-data2.zip › Figure 7-source data 1/Figure 7-source data 1.pdf]

Figure 7D

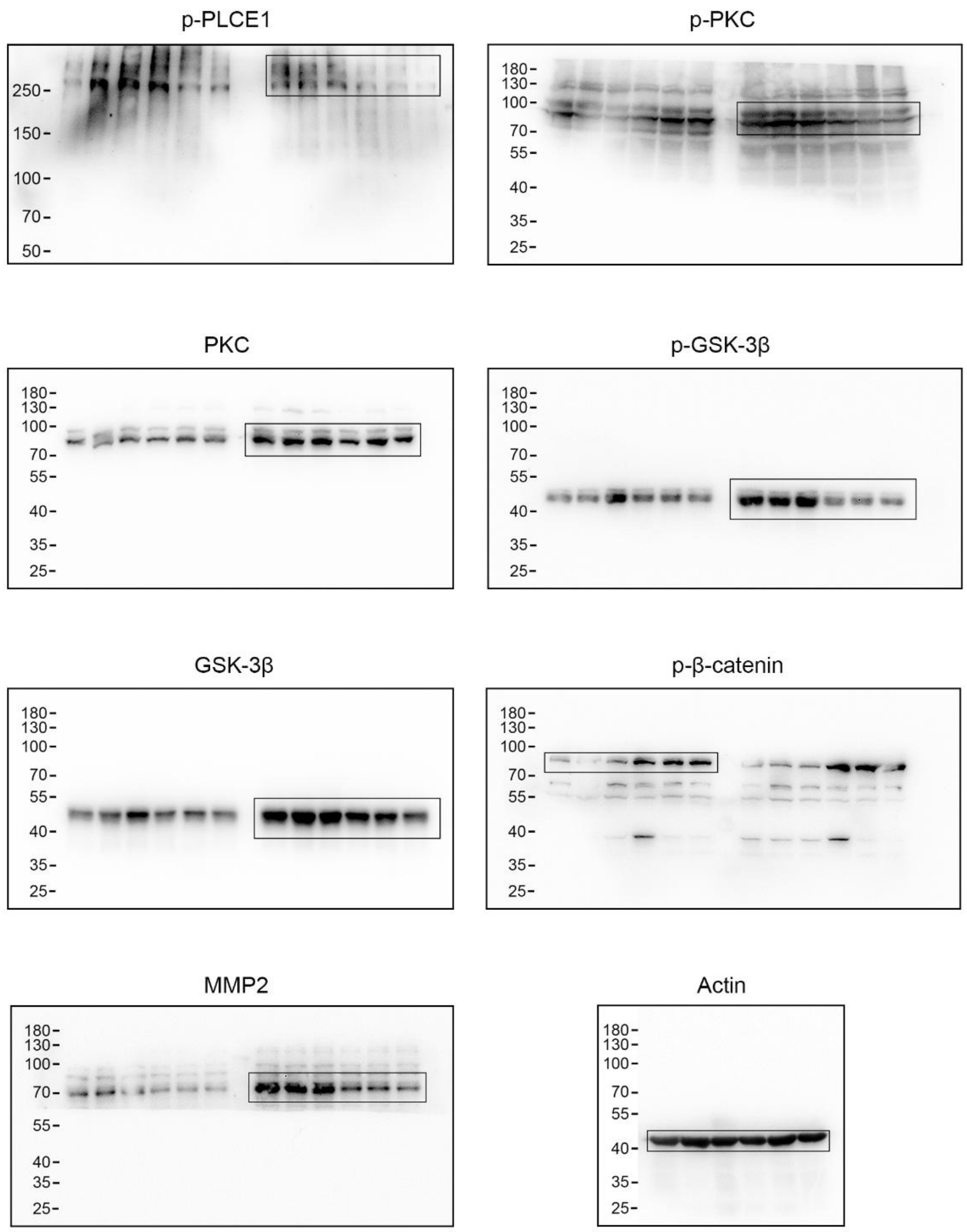

Figure 7, Source Data 1. Original membranes corresponding to Figure 7, panel D, indicating the relevant bands.
