# Supplementary material for: TAK1-mediated phosphorylation of PLCE1 represses PIP2 hydrolysis to impede esophageal squamous cancer metastasis
Source: eLife. 2025 Apr 23;13:RP97373. doi: 10.7554/eLife.97373 (PMC12017773; doi:10.7554/eLife.97373)
Supplement: Supplementary file 1. [file elife-97373-supp1.docx]

**Supplementary file 1**

**Table S1. The sequences used in gene knockdown and mutation.**

| Genes | Sequences (5’-3’) |
| --- | --- |
| *Map3k7* siRNA | GGAGTTGTTTGCAAAGCTA |
| *Plce1* siRNA-1 | GGACTTCAATATCGCAGTA |
| *Plce1* siRNA-2 | GTCGAAGTGTAGAATTGGA |
| *Plce1* siRNA-3 | CAATCATCATATCGATTGA |
| *Map3k7* gRNA | F: ccgAGGGGCTTCGATCATCTCAC |
|  | R: aacGTGAGATGATCGAAGCCCCT |
| *Plce1* (S1060A) | F: TGGAGTGCTCGAAACCCCGCACCCGGAACATCAGCAAA |
|  | R: GGGGTTTCGAGCACTCCACCGTCTGCCACCAAACAA |

F: forward; R: reverse.
