# Supplementary material for: TAK1-mediated phosphorylation of PLCE1 represses PIP2 hydrolysis to impede esophageal squamous cancer metastasis
Source: eLife. 2025 Apr 23;13:RP97373. doi: 10.7554/eLife.97373 (PMC12017773; doi:10.7554/eLife.97373)
Supplement: Supplementary file 2. [file elife-97373-supp2.docx]

**Supplementary file 2**

**Table S2. Primer sequences used in qRT-PCR.**

| *Genes* | Primer sequences (5’-3’) |
| --- | --- |
| *Map3k7* | F: ATTGTAGAGCTTCGGCAGTTATC |
|  | R: CTGTAAACACCAACTCATTGCG |
| *Plce1* | F: GGGTGACATGGCTGATCCTC |
|  | R: GACAGCGTTGTAGTTGCCCA |
| *Cdh1* | F: GCTTTACTGTTTCTCAAGTGT |
|  | R: AATACACAATTATCAGCACCC |
| *Vim* | F: AACTTCTCAGCATCACGAT |
|  | R: GTAGGAGTGTCGGTTGTT |
| *Ctnnb1* | F: AGAATTGAGTAATGGTGTAGAAC |
|  | R: TACCCATACATATCCCAAATAGT |
| *Cldn1* | F: TGTATAGTCCTCTTGGGTTG |
|  | R: AATTGTCAGTGGAGTCAGT |
| *Cdh2* | F: AAAAGGAAAGGAAAGAAAGGG |
|  | R: GTCAGAGGTGTATCATTTATATTCT |
| *Zeb1* | F: GTTGCTCCTTCTTCCTGA |
|  | R: ATGTGGTTCCTGTTCCTAG |
| *Tjp1* | F: TGTGGACATCCTACTTACTTAA |
|  | R: GAGAAGATAAAGAAACTGTTGTATG |
| *Snai1* | F: AGCTATTTCAGCCTCCTG |
|  | R: TGTAAACATCTTCCTCCCAG |
| *Snai2* | F: CTGTATGAAACTGAGATGTTGT |
|  | R: GAAGCAAGTAAAGTCTCTGAAA |
| *Gapdh* | F: AAGAGCACAAGAGGAAGAG |
|  | R: TAACTGGTTGAGCACAGG |

F: forward; R: reverse.
